# Needle extracts of *Pinus squamata* X.W. Li ameliorate obesity and modulate cecal microbiota in high-fat diet-induced obese mice

**DOI:** 10.3389/fphar.2026.1906430

**Published:** 2026-09-22

**Authors:** Xun Zhang, Tianshuang Wang, Hong Chen, Yanping Hu, Lei Tang, Shuai Chang

**Affiliations:** 1 State Key Laboratory of Discovery and Utilization of Functional Components in Traditional Chinese Medicine, School of Pharmaceutical Sciences, Guizhou Medical University, Guian New Area, Guizhou, China; 2 School of Traditional Chinese Pharmacy, China Pharmaceutical University, Nanjing, Jiangsu, China; 3 Research Institute, Management Bureau of Yaoshan National Nature Reserve, Zhaotong, Yunnan, China

**Keywords:** cecal microbiota, high-fat diet, metabolic regulation, obesity, pinus squamata

## Abstract

Obesity is closely associated with excessive adipose accumulation, chronic low-grade inflammation, and gut microbiota dysbiosis. Although needle extracts from several *Pinus* species have been reported to attenuate body weight gain and modulate the gut microbiota, the pharmacological properties of *Pinus squamata* X.W. Li—an endangered coniferous species endemic to northeastern Yunnan, China—remain largely unexplored. This study evaluated the effects of *Pinus squamata* needle extract (PSNE) on high-fat diet (HFD)-induced obesity in mice. Our results demonstrated that PSNE significantly mitigated HFD-induced body weight gain, adipose accumulation, hepatic steatosis and injury, dyslipidaemia, oxidative stress, and systemic low-grade inflammation, while concomitantly improving glucose homeostasis. Furthermore, 16S rRNA amplicon sequencing of cecal contents revealed that PSNE increased amplicon sequence variant (ASV) richness and α-diversity, remodelled the cecal microbiota, and reversed HFD-associated dysbiosis. These beneficial alterations were characterised by significant enrichment of taxa with reported metabolic benefits, such as *Bacteroides*, Muribaculaceae and Ruminococcaceae, together with a concomitant reduction in obesity-associated lineages. Collectively, these findings establish PSNE as a promising preclinical candidate for alleviating obesity and related metabolic disorders, potentially through targeted remodelling of the cecal microbiota.

## 1 Introduction

The rapid expansion of the global economy, together with substantial improvements in living standards, has driven a pronounced global shift in dietary patterns toward the widespread consumption of high-fat, high-sugar foods. These energy-dense yet nutrient-poor diets are now recognised as major environmental contributors to the pathogenesis of obesity and a spectrum of related metabolic disorders. Given that billions of individuals are affected by obesity, type 2 diabetes, and their associated complications, addressing these conditions has become an urgent global public health priority (Romieu et al., 2017).

It is well established that HFD consumption not only promotes excessive weight gain but also disrupts systemic metabolic homeostasis, thereby substantially increasing the risk of chronic disease development (Chen et al., 2023). Excessive sugar intake induces aberrant insulin secretion, leading to insulin resistance, whereas chronic HFD feeding triggers persistent low-grade inflammation (Krishnan et al., 2026). Nevertheless, the prevalence of HFD-based dietary patterns remains largely unchecked, and the global expansion of fast-food culture, together with the widespread availability of ultra-processed and refined foods, has further intensified this public health crisis (Popkin and Hawkes, 2016). Therefore, comprehensive investigation of the adverse health effects of HFD and the development of effective interventions are critical for improving population health and reducing the burden of chronic disease.

The gut microbiota plays a critical role in maintaining intestinal barrier integrity and mucosal immunity, thereby supporting host health (Gieryńska et al., 2022). Long-term HFD consumption disrupts microbial homeostasis and impairs liver function (Terzo et al., 2020). Such dysbiosis directly contributes to metabolic dysregulation, including disturbances in glucose, lipid, and amino acid metabolism (Hu et al., 2022; Su et al., 2022). Notably, alterations in microbiota composition and metabolite profiles are well-established hallmarks of obesity and metabolic diseases, as demonstrated in animal studies (Ling et al., 2021; Sheykhsaran et al., 2023; Ye et al., 2021). In particular, an increased Firmicutes/Bacteroidota (F/B) ratio is strongly associated with these disorders (Porras et al., 2017). Importantly, natural bioactive compounds can modulate gut microbial communities by enriching beneficial commensal bacteria, inhibiting opportunistic pathogens, and promoting the restoration of a healthy gut environment (Gong et al., 2023; Lan et al., 2024; Li et al., 2023). Thus, profiling gut microbiota composition not only refines the prediction of obesity risk but also facilitates the identification of novel therapeutic targets for metabolic disorders.

Pine needles, the principal medicinal organs of *Pinus* species, constitute sustainable and renewable resources enriched with diverse nutrients and bioactive metabolites. They have well-documented nutritional and medicinal value in both traditional applications and modern pharmacology. Pharmacological investigations have demonstrated that pine needles exert a broad spectrum of bioactivities, including anticancer, antioxidant, anti-obesity, anti-inflammatory, antihypertensive, and antibacterial effects (Khatamian et al., 2022; Kim et al., 2021; Kuo et al., 2021; Liang et al., 2025; Popescu et al., 2023). Pine needle essential oil, in particular, exhibits potent free-radical-scavenging and antibacterial activities (Aloui et al., 2021). Needles of *Cedrus deodara* (Roxb.) G. Don have been reported to display anti-obesity activity, with phenolic acids identified as the predominant active components (Wang et al., 2024). An ethanol extract derived from *Pinus koraiensis* Siebold and Zucc. needles attenuates hypertriglyceridemia and reduces hepatic triglyceride (TG) accumulation by suppressing the expression of genes involved in *de novo* lipogenesis and pro-inflammatory signalling (Lee et al., 2022). Pine needle extracts have also been shown to enhance hypothalamic pro-opiomelanocortin neuronal activity, an effect abrogated by pharmacological blockade of melanocortin 3/4 receptors. In HFD-fed mice, administration of pine needle extract significantly reduced body weight, fat mass, and plasma leptin levels, while improving glucose metabolism following diet-induced obesity. Notably, however, these extracts did not significantly alter the expression of thermogenic markers in brown adipose tissue of HFD-fed mice, but selectively reduced the expression of lipogenic markers in white adipose tissue (Kim et al., 2021). Collectively, these well-characterised bioactivities underscore the potential of pine needles as valuable natural resources for the development of functional foods and nutraceuticals.

*Pinus squamata* X.W. Li is currently designated as a Plant Species with Extremely Small Populations (PSESP) in China, necessitating urgent conservation measures. Despite its intrinsically limited natural population, artificial propagation and cultivation of this species have expanded markedly in recent years. To date, however, research on *Pinus squamata* has focused predominantly on its rhizosphere microbiome, chloroplast genome, and the interspecific associations of dominant woody species (Li et al., 2024; Ling and Zhang, 2019; Zhang et al., 2003). However, no traditional medicinal use of *Pinus squamata* has been documented, and its pharmacological activity remains completely unexplored. Given the well-established traditional use of other *Pinus* species for metabolic disorders, and the recognised ethnopharmacological relevance of the genus, PSNE warrants investigation as a potential source of bioactive principles. Confirming these properties would provide a scientific foundation for its conservation and sustainable utilisation. Accordingly, this study aimed to evaluate the preventive effects of PSNE against HFD-induced obesity in mice and to systematically assess its modulatory influence on cecal microbiota composition.

## 2 Materials and methods

### 2.1 Collection and extraction of pine needles

Fresh needles of artificially propagated *Pinus squamata* X.W. Li were collected on 12 June 2024 from an artificial reintroduction site within the species’ native range at Yangjiawan Village, Baihetan Town, Qiaojia County, Yunnan Province, China (26.87°N, 103.01°E). A voucher specimen was deposited in the Herbarium of Guizhou Medical University under the number GMC530622A001. All experiments were performed using a single batch of plant material and extracts to ensure internal consistency of the pharmacological data. All sampling procedures complied with relevant biodiversity regulatory requirements. Following collection, the needles were shade-dried under ambient conditions and mechanically pulverised into a fine powder.

Essential oil was extracted from the powdered material by steam distillation. For solvent-based extraction, reflux extraction was conducted at 120 °C for 3 h using a solid-to-liquid ratio of 1:10 (g/mL) with four extraction solvents: deionised water and 30%, 60%, and 90% (v/v) aqueous ethanol. Each extraction (steam distillation and reflux) was performed twice. The resulting crude extracts were vacuum-filtered, concentrated under reduced pressure using a rotary evaporator, and subsequently freeze-dried to yield solvent-specific extract powders. For subsequent experiments, all extracts, including the essential oil and solvent-derived extract powders, were collectively designated as PSNE. The specific extract type and dose used in each assay are indicated in the corresponding results section and figure legends. The yields of the essential oil and the four solvent extracts were 0.2% (w/w) and approximately 5% (w/w), respectively, with the four solvent extracts showing comparable yields. The description of plant material and extraction procedures follows the ConPhyMP guidelines (Heinrich et al., 2022).

### 2.2 Design of animal experiments

All animal procedures were conducted in accordance with protocols approved by the Animal Care Welfare Committee of Guizhou Medical University (Approval No. 2500388). A total of 136 SPF-grade male C57BL/6J mice aged 6 weeks were obtained from SPF Biotechnology Co., Ltd (Beijing, China). The mice were maintained under controlled environmental conditions (22 °C ± 2 °C, 50%–60% relative humidity) with a 12 h light/dark cycle and *ad libitum* access to food and water.

To determine the optimal dose range, a preliminary dose-finding study was performed. Mice were randomly assigned to receive PSNE at three doses: low dose (125 mg/kg), medium dose (250 mg/kg), or high dose (500 mg/kg). No significant dose-dependent effect was observed among the three groups, and key pharmacodynamic endpoints did not differ significantly between the low- and medium-dose groups. These findings indicated comparable efficacy between the low and medium doses, with no additional benefit conferred by the medium dose. In accordance with the Reduction principle of the 3Rs framework, only the low-dose (125 mg/kg) and high-dose (500 mg/kg) groups were retained for the main experiment, thereby minimising unnecessary animal use while preserving scientific validity.

After a 1-week acclimatisation period, mice were randomly allocated into 13 groups using a random number table. The groups were as follows: normal chow diet (NCD, n = 12); high-fat diet (HFD, n = 12); simvastatin (SIM, 20 mg/kg) as a positive control (n = 12); and ten PSNE-treated groups, namely, low-dose aqueous extract (WL, n = 10), high-dose aqueous extract (WH, n = 10), low-dose 30% ethanol extract (E30L, n = 10), high-dose 30% ethanol extract (E30H, n = 10), low-dose 60% ethanol extract (E60L, n = 10), high-dose 60% ethanol extract (E60H, n = 10), low-dose 90% ethanol extract (E90L, n = 10), high-dose 90% ethanol extract (E90H, n = 10), low-dose volatile oil (VOL, n = 10), and high-dose volatile oil (VOH, n = 10).

Treatments were administered once daily by oral gavage for 15 consecutive weeks. Mice in the PSNE groups received the corresponding extracts at either a low dose (125 mg/kg body weight) or a high dose (500 mg/kg body weight), whereas mice in the SIM group received SIM (20 mg/kg body weight; Prom-In, et al., 2020). The NCD and HFD control groups received an equivalent volume of normal saline *via* the same route. With the exception of the NCD group, all groups were fed an HFD (60% kcal from fat; SYHF60-1), while the NCD group received a standard normal diet (10% kcal from fat). Gavage treatment was initiated concomitantly with the onset of HFD feeding. Daily food intake and weekly body weight were recorded throughout the 15-week experimental period.

### 2.3 Oral glucose tolerance test (OGTT)

An OGTT was performed after 15 weeks of PSNE administration. Briefly, mice were fasted overnight for 12 h with *ad libitum* access to water and subsequently administered an oral glucose load (2.0 g/kg body weight). Blood glucose concentrations were measured in whole blood collected from the tail vein at 0 min (baseline), 15 min, 30 min, 60 min, 90 min, and 120 min after glucose challenge using a Sinocare GA-3 glucose meter (Sinocare Inc., Changsha, China). Blood glucose concentration–time curves were generated, and the area under the curve (AUC) was calculated using the trapezoidal rule.

### 2.4 Serum biochemical indices analysis

At the end of the 15-week intervention, mice were deeply anaesthetised by intraperitoneal injection of pentobarbital sodium (50 mg/kg body weight) and euthanised by cervical dislocation. Whole blood was collected from the retro-orbital venous plexus; liver and inguinal white adipose tissue (iWAT) were excised, weighed, snap-frozen in liquid nitrogen, and stored at −80 °C until subsequent analysis. Serum levels of total cholesterol (TC), TG, low-density lipoprotein cholesterol (LDL-C), high-density lipoprotein cholesterol (HDL-C), alanine aminotransferase (ALT), and aspartate aminotransferase (AST) were quantified using commercial assay kits (Nanjing Jiancheng Bioengineering Institute, Nanjing, China) in accordance with the manufacturer’s instructions. Serum concentrations of interleukin-6 (IL-6), interleukin-1β (IL-1β), and tumour necrosis factor α (TNF-α) were determined by enzyme-linked immunosorbent assay (ELISA; Shanghai Fanke Biotechnology Co., Ltd., Shanghai, China) according to the manufacturer’s protocols.

### 2.5 Hepatic biochemical analysis

Hepatic activities of superoxide dismutase (SOD), catalase (CAT), and glutathione peroxidase (GSH-Px), as well as the level of malondialdehyde (MDA), a marker of lipid peroxidation, were measured using commercial assay kits (Jiangsu Edisen Biotechnology Co., Ltd., China) in accordance with the manufacturer’s instructions. Briefly, frozen liver tissues were pulverised in liquid nitrogen and homogenised in ice-cold normal saline at a ratio of 1:9 (w/v). The homogenates were centrifuged at 2,500 × g for 10 min at 4 °C, and the resulting supernatants were collected for immediate analysis.

### 2.6 Histopathological evaluation

Freshly collected iWAT and liver tissues were fixed in 4% paraformaldehyde for 24 h. For haematoxylin and eosin (H&E) staining, tissues were dehydrated, paraffin-embedded, sectioned, and stained with H&E. H&E-stained sections were qualitatively assessed for overall tissue morphology, adipocyte size, crown-like structures, and inflammatory infiltration. For Oil Red O (ORO) staining, liver tissues were embedded in optimal cutting temperature (OCT) compound, cryosectioned, and stained with ORO. Stained sections were imaged using a digital microscope (Motic China Group Co., Ltd., Beijing, China). All histological analyses were performed blinded, with group allocation concealed during both image acquisition and quantification. For each animal, a minimum of three non-overlapping fields per section were randomly selected. ORO-stained liver sections were quantified using Image-Pro Plus 6.0 (Media Cybernetics, United States), and lipid droplet content was expressed as the percentage of lipid droplet area relative to the total field area. All tissue processing, sectioning, and staining procedures were conducted at Chengdu Lilai Biotechnology Co., Ltd (Chengdu, China).

### 2.7 Cecal microbiota analysis

For cecal microbiota profiling, three cecal content samples from each group were randomly selected. Total genomic DNA was extracted using the FastDNA Spin Kit (MP Biomedicals, Santa Ana, CA, United States) in accordance with the manufacturer’s protocol. DNA integrity and purity were evaluated by 1% agarose gel electrophoresis. The V3-V4 hypervariable region of the bacterial 16S rRNA gene was amplified using barcoded primers 338F (5′-ACT​CCT​ACG​GGA​GGC​AGC​AG-3′) and 806R (5′-GGACTACHVGGGTWTCTAAT-3′). Paired-end sequencing was performed on an Illumina MiSeq platform by Shanghai Majorbio Bio-Pharm Technology Co., Ltd (Shanghai, China).

Raw sequencing data were processed to generate ASVs. Based on the ASV dataset, rarefaction curves and alpha diversity indices, including the number of observed ASVs, the ACE index, the Chao1 richness index, and the Shannon diversity index, were calculated using mothur (version 1.30.1). Differences in microbial community structure among samples were assessed by principal coordinate analysis (PCoA) based on Bray–Curtis dissimilarity using the vegan package (v2.5-3). Linear discriminant analysis effect size (LEfSe) was performed to identify differentially abundant bacterial taxa from the phylum to genus levels among groups, with thresholds set at a linear discriminant analysis (LDA) score >2 and *p* < 0.05. Furthermore, Spearman’s rank correlation analysis was conducted to evaluate associations between cecal microbial taxa and host lipid metabolism parameters.

### 2.8 Statistical analysis

Data were analysed using one-way ANOVA followed by Fisher’s LSD *post hoc* test and are expressed as the mean ± standard deviation (x̅ ± SD). Prior to ANOVA, normality of residuals was assessed using the Shapiro-Wilk test, and homogeneity of variances was evaluated using Levene’s test. As variances were homogeneous across groups, Fisher’s LSD was applied as a *post hoc* test for exploratory comparisons. Exact P-values and 95% confidence intervals for all pairwise comparisons are provided in Supplementary Table 1. Statistical analyses were conducted using SPSS version 26.0 (IBM Corp., Armonk, NY, United States). The LSD *post hoc* test was used as an exploratory tool to identify potential differences; findings are considered hypothesis-generating and require further validation. Differences with *p <* 0.05 were considered statistically significant. The number of samples analysed for each experimental endpoint varied across groups, primarily due to unavoidable technical losses during sample processing and limited tissue availability for specific assays. A comprehensive breakdown of sample sizes (n) for all measured parameters across all groups is provided in Supplementary Data Sheet 1 (Supplementary Table).

## 3 Results

### 3.1 Effects of PSNE on body, iWAT and liver weight in HFD-induced obese mice

Throughout the 15-week experimental period, no significant differences in either initial body weight or mean weekly energy intake were observed among the experimental groups (Figures 1A,B). A transient decrease in body weight occurred in all groups during the early phase, followed by a sustained and progressive increase (Figure 1A). At the end of the 15-week dietary intervention, mice fed the HFD alone showed markedly greater body weight gain, iWAT mass, and liver weight than those in the NCD control group. Co-administration of PSNE significantly attenuated HFD-induced body weight gain and reduced iWAT and liver weights (Figures 1C–E). Specifically, the greatest cumulative weight gain was observed in the HFD group. Compared with the HFD group, statistically significant reductions in weight gain were detected in the SIM, E60L, E90L, E90H, and VOH groups (*p* < 0.01), as well as in the WL, WH, and VOL groups (*p* < 0.05) (Figure 1C). Furthermore, iWAT weights were significantly lower in the WH, E60L, E90L, E90H, and VOH groups (*p* < 0.01), and in the SIM, WL, E30L, and VOL groups (*p* < 0.05), compared with the HFD group (Figure 1D). Liver weights were significantly decreased in the SIM, WL, E60H, and E90H groups relative to the HFD group (*p* < 0.05) (Figure 1E).

**FIGURE 1:**
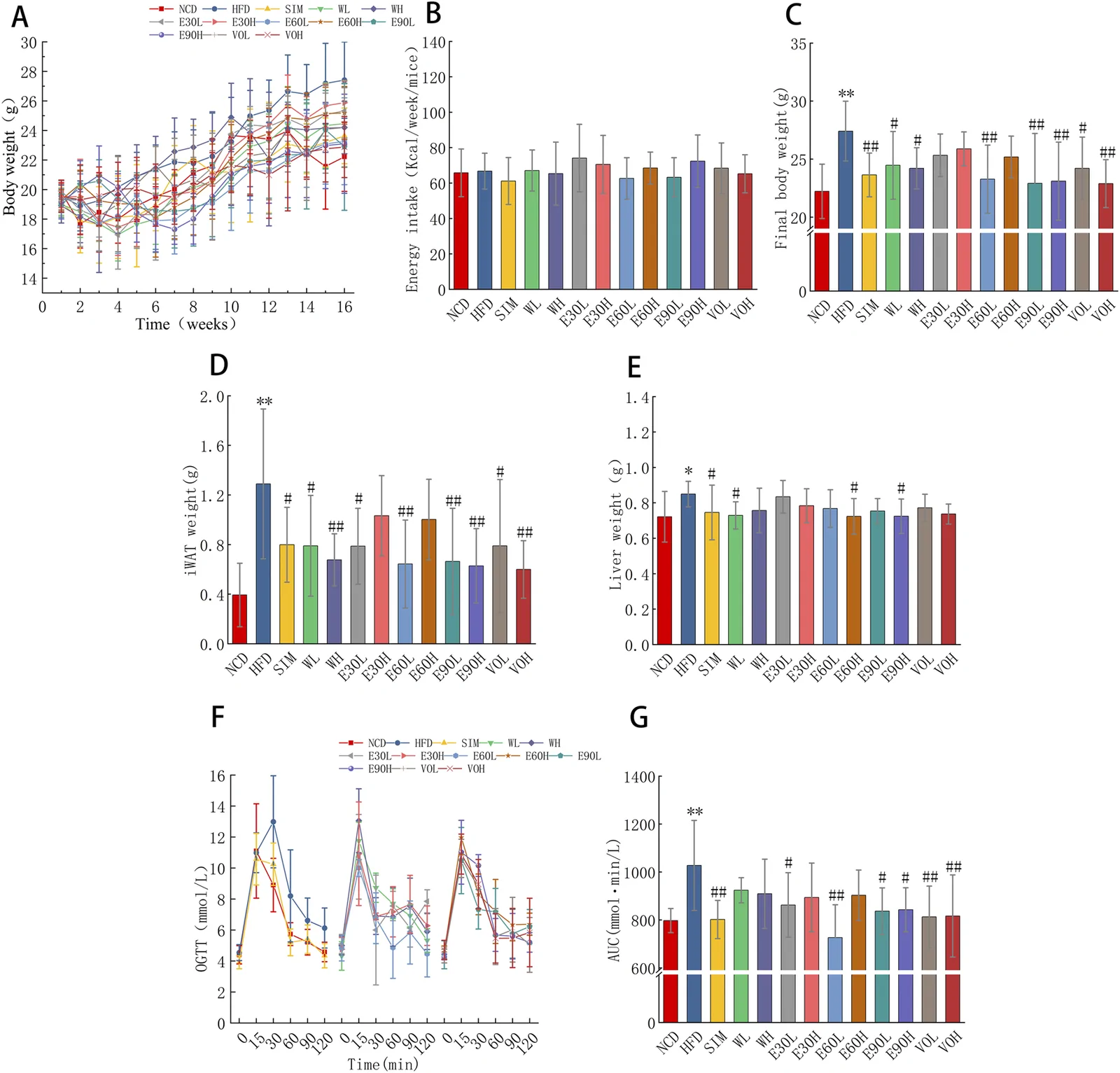
Effects of PSNE supplementation on body weight, iWAT weight, liver weight and OGTT in HFD-induced obese mice. **(A)** Body weight. **(B)** Weekly energy intake. **(C)** Final body weight. **(D)** iWAT weight. **(E)** Liver weight. **(F)** OGTT curve. **(G)** AUC of OGTT. Data are expressed as the mean ± SD. ^∗^ *p* < 0.05, ^∗∗^ *p* < 0.01 compared with the NCD group; ^#^ *p* < 0.05, ^##^ *p* < 0.01 compared with the HFD group. Groups and diets: All groups except NCD were fed an HFD. NCD, normal chow diet; HFD, high-fat diet; SIM, HFD + simvastatin (20 mg/kg, positive control); WL/WH, HFD + low/high-dose aqueous extract; E30L/E30H, HFD + low/high-dose 30% ethanol extract; E60L/E60H, HFD + low/high-dose 60% ethanol extract; E90L/E90H, HFD + low/high-dose 90% ethanol extract; VOL/VOH, HFD + low/high-dose volatile oil.

### 3.2 PSNE improves glucose homeostasis in HFD-induced obese mice

OGTT was performed 1 week before euthanasia. Following glucose administration, blood glucose levels increased rapidly in all groups and peaked at 15 min in the NCD and PSNE-treated groups, whereas the peak was delayed to 30 min in the HFD group (Figure 1F). AUC was significantly lower in the SIM, E60L, VOL, and VOH groups (*p* < 0.01), and in the E30L, E90L, and E90H groups (*p* < 0.05), relative to the HFD group (Figure 1G).

### 3.3 Effects of PSNE on serum biochemical indicators

At the conclusion of the 15-week intervention period, the HFD group exhibited elevated serum levels of TC, TG, and LDL-C (*p* < 0.01), accompanied by a significant reduction in HDL-C (*p* < 0.05), relative to the NCD control group (Figures 2A–D). PSNE administration was associated with changes in these lipid parameters in HFD-fed mice. Specifically, serum TC levels were significantly reduced in the SIM, WH, E90L, E90H, VOL, and VOH groups (*p* < 0.01) (Figure 2A); TG levels were significantly decreased in the E90H and VOH groups (*p* < 0.01) as well as in the WH, E90L, and VOL groups (*p* < 0.05) (Figure 2B); LDL-C levels were significantly lower in the SIM, E90L, E90H, and VOH groups (*p* < 0.01), and in the VOL group (*p* < 0.05) (Figure 2C); and HDL-C levels were significantly elevated in the WL, WH, E30L, and E90H groups (*p* < 0.01), as well as in the E60L and E60H groups (*p* < 0.05) (Figure 2D).

**FIGURE 2:**
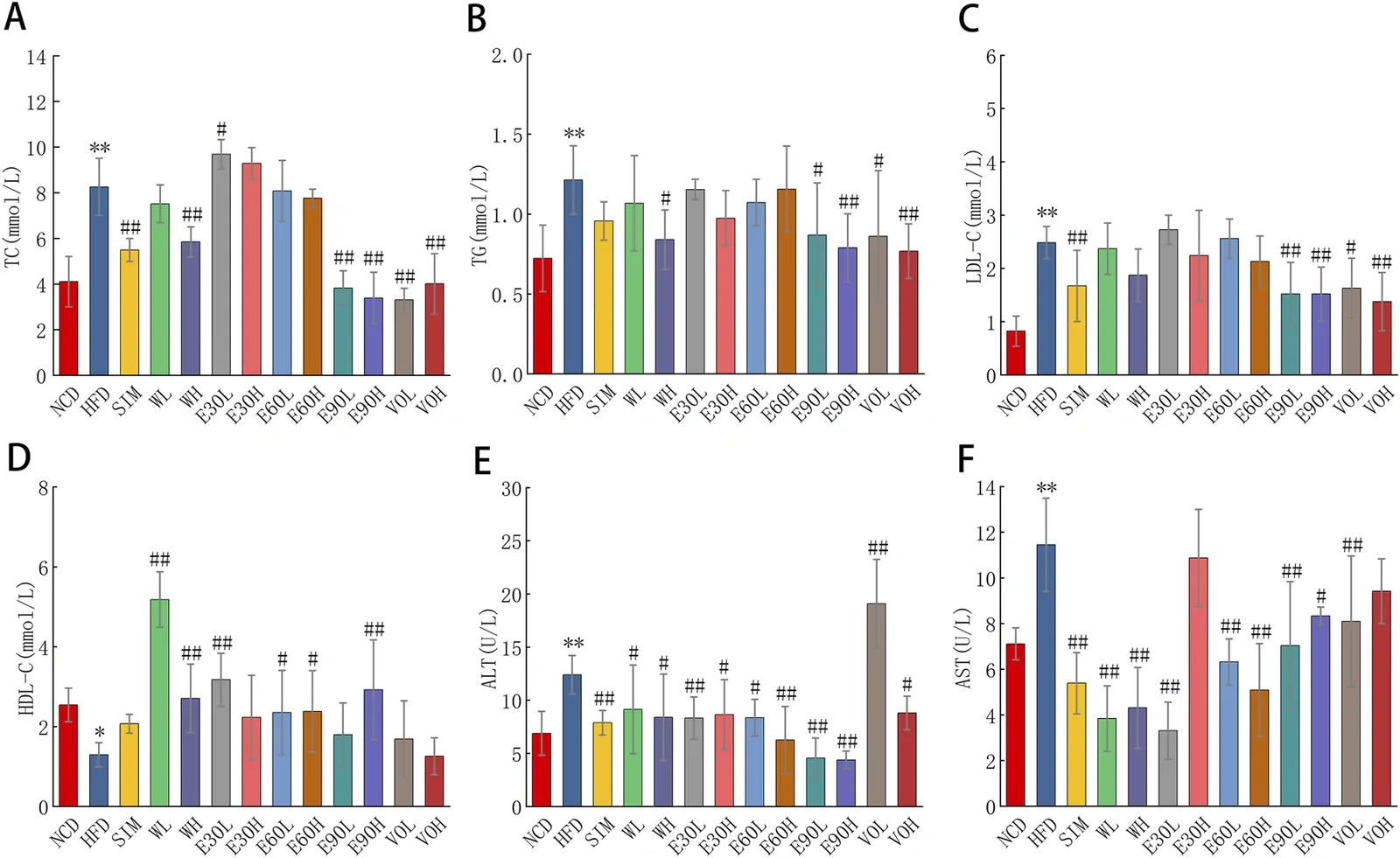
Effects of PSNE supplementation on serum lipid profiles and hepatic injury biomarkers. **(A)** Serum levels of TC, **(B)** TG, **(C)** LDL-C, **(D)** HDL-C, **(E)** ALT and **(F)** AST. Data are expressed as the mean ± SD. ^∗^ *p* < 0.05, ^∗∗^ *p* < 0.01 compared with the NCD group; ^#^ *p* < 0.05, ^##^ *p* < 0.01 compared with the HFD group. Group abbreviations and diets are the same as defined in the legend of Figure 1.

### 3.4 Effects of PSNE on ALT and AST levels

Serum activities of ALT and AST were significantly higher in the HFD group than in the NCD group (*p* < 0.01). PSNE administration was associated with reduced levels of these enzymes. Serum ALT activity was significantly reduced in the SIM, E30L, E60H, E90L, and E90H groups (*p* < 0.01), and in the WL, WH, E30H, E60L, and VOH groups (*p* < 0.05) (Figure 2E); serum AST activity was significantly decreased in the SIM, WL, WH, E30L, E60L, E60H, E90L, and VOL groups (*p* < 0.01), and in the E90H group (*p* < 0.05) (Figure 2F). Collectively, these results demonstrate that PSNE effectively alleviates HFD-induced hepatic injury.

### 3.5 PSNE attenuates hepatic oxidative stress and inflammation

The HFD group exhibited significantly reduced activities of SOD, GSH-Px, and CAT compared with the NCD group (*p* < 0.01). Following PSNE administration, the following changes were observed. SOD activity was significantly increased in the WL group (*p* < 0.05) (Figure 3A); GSH-Px activity was markedly elevated in the WL group (*p* < 0.01), as well as in the SIM and WH groups (*p* < 0.05) (Figure 3B); and CAT activity was significantly increased in the E30H and E90L groups (*p* < 0.01), as well as in the SIM, E60L, E60H, and E90H groups (*p* < 0.05) (Figure 3C). Concurrently, hepatic MDA content, a marker of lipid peroxidation, was significantly reduced in the SIM, E30L, E30H, E60L, E60H, E90L, E90H, VOL, and VOH groups (*p* < 0.01), and in the WH group (*p* < 0.05) (Figure 3D).

**FIGURE 3:**
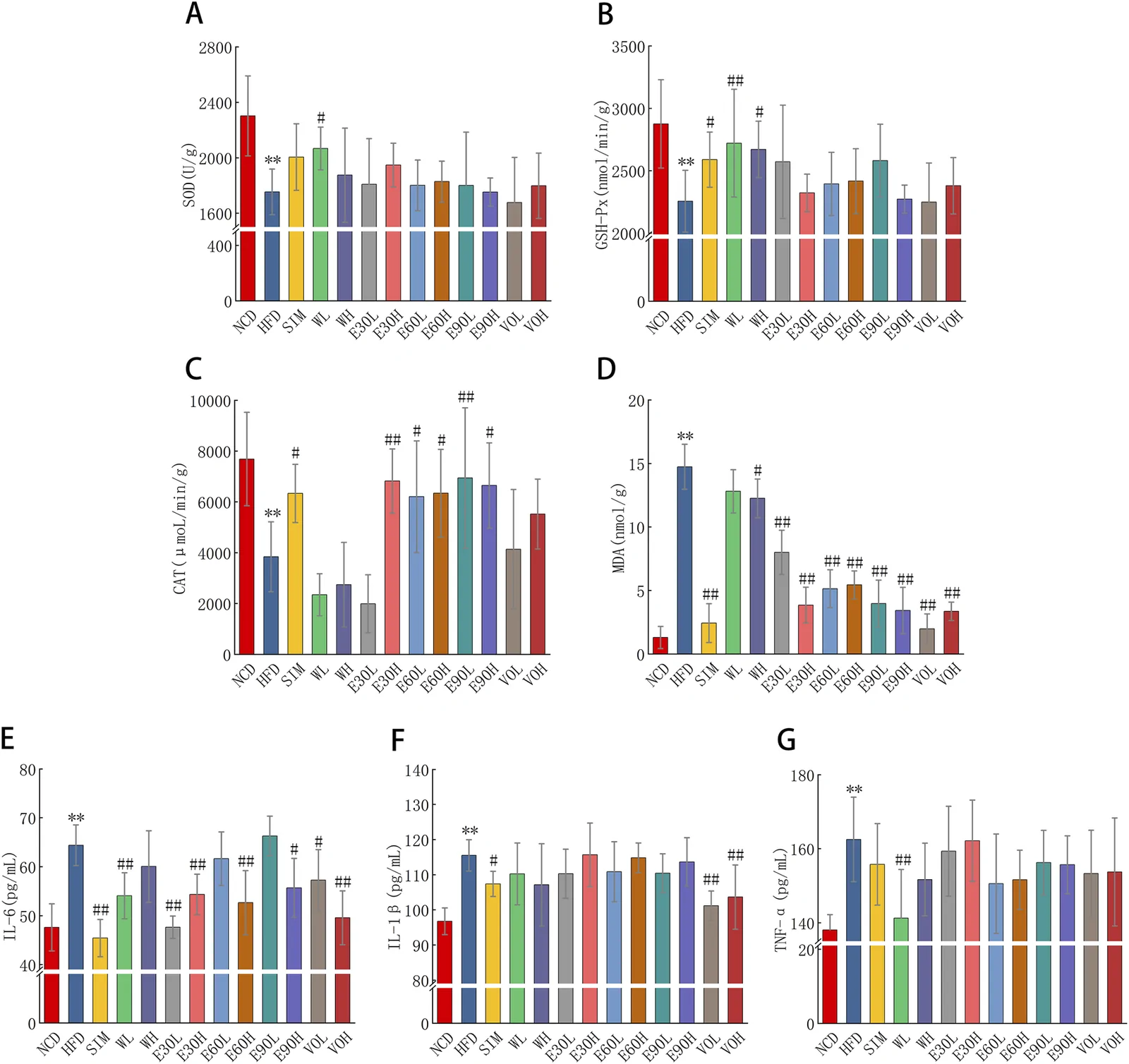
Effects of PSNE on hepatic oxidative stress and inflammation. **(A)** Hepatic SOD, **(B)** GSH-Px, **(C)** CAT, **(D)** MDA, **(E)** Serum IL-6, **(F)** IL-1β and **(G)** TNF-α. Data are expressed as the mean ± SD. ^∗^ *p* < 0.05, ^∗∗^ *p* < 0.01 compared with the NCD group; ^#^ *p* < 0.05, ^##^ *p* < 0.01 compared with the HFD group. Group abbreviations and diets are the same as defined in the legend of Figure 1.

Consistent with the metabolic disturbances described above, serum concentrations of IL-6, IL-1β, and TNF-α were markedly elevated in the HFD group compared with the NCD group (*p* < 0.01). Following PSNE administration, changes in these parameters were observed. Specifically, serum IL-6 levels were significantly reduced in the SIM, WL, E30L, E30H, E60H, and VOH groups (*p* < 0.01), and in the E90H and VOL groups (*p* < 0.05) (Figure 3E); IL-1β levels were markedly decreased in the VOL and VOH groups (*p* < 0.01), and in the SIM group (*p* < 0.05) (Figure 3F); and TNF-α levels were significantly reduced in the WL group (*p* < 0.01) (Figure 3G).

### 3.6 Histopathological evaluation of iWAT and liver tissues

The morphology of iWAT and liver tissue in each group is shown in Supplementary Data Sheet 1 (Supplementary Figures S1 and S2). Histological analysis of iWAT showed that adipocytes in the NCD group exhibited a normal unilocular morphology with a uniform cellular architecture, and no evidence of hypertrophy, shrinkage, or inflammatory cell infiltration. This normal histological phenotype was likewise observed in the SIM, WL, WH, E60L, E60H, E90L, E90H, VOL, and VOH groups. In contrast, the HFD group displayed adipocyte hypertrophy accompanied by pronounced enlargement of cytoplasmic lipid droplets, whereas the E30L and E30H groups exhibited only partial adipocyte expansion (Figure 4A).

**FIGURE 4:**
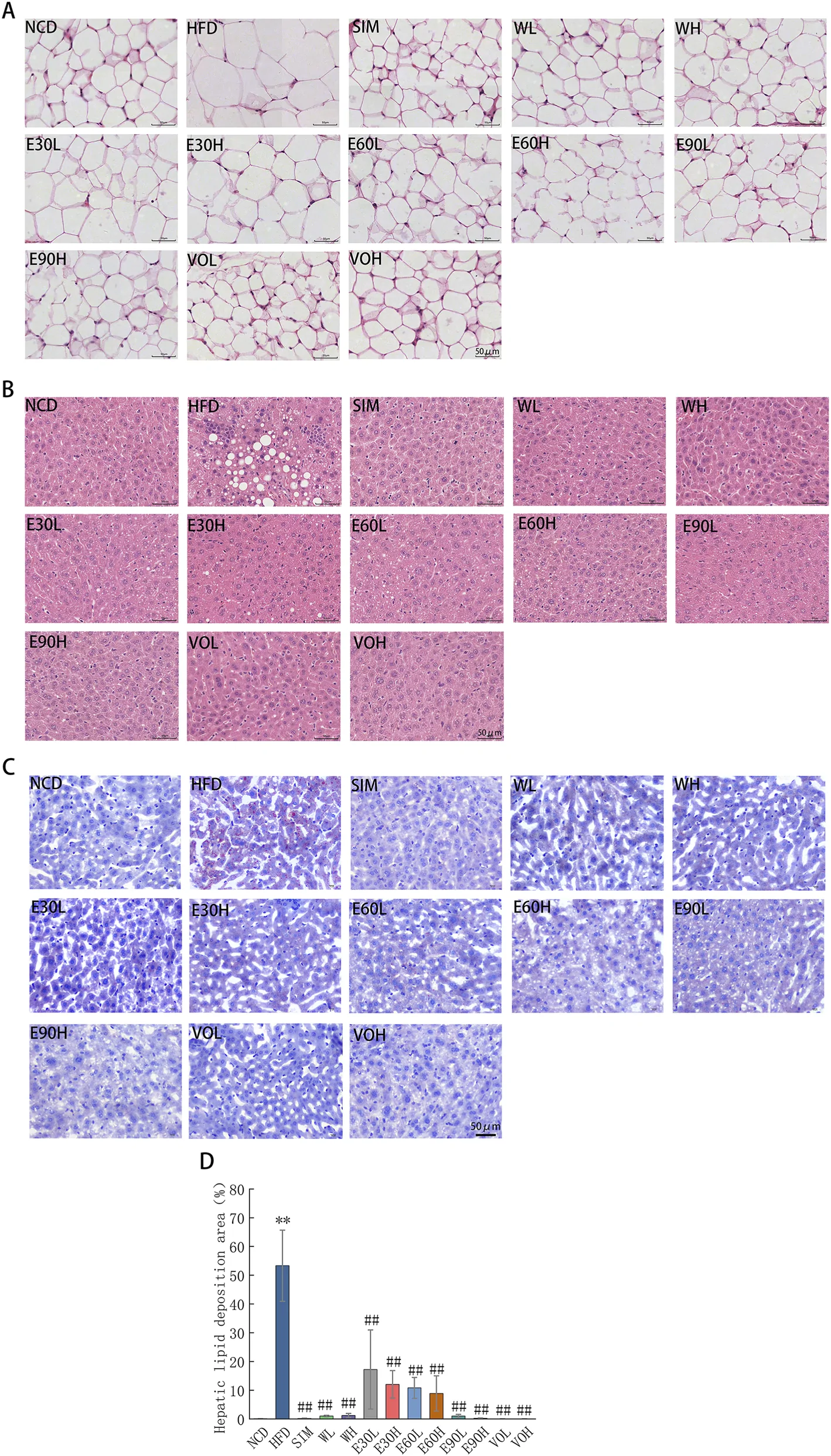
Representative histopathological images of adipose and liver tissues. **(A)** Representative H&E staining of iWAT. **(B)** Representative H&E staining of liver tissue. **(C)** Oil Red O staining of hepatic sections (scale bar, 50 μm). **(D)** Liver lipid accumulation area. Data are expressed as the mean ± SD (*n* = 3). ^∗∗^ *p* < 0.01 compared with the NCD group; ^##^ *p* < 0.01 compared with the HFD group. Group abbreviations and diets are the same as defined in the legend of Figure 1.

Histopathological examination of liver sections revealed distinct morphological alterations among the experimental groups. Liver tissues from the NCD, SIM, WL, WH, E90L, E90H, VOL, and VOH groups displayed largely normal hepatic architecture, characterised by intact hepatic capsules, well-preserved lobular structure, orderly arranged hepatocytes, and normal portal triads, with only sporadic cytoplasmic vacuolation. In contrast, liver tissues from the HFD group exhibited hepatic steatosis, characterised by microvesicular and macrovesicular lipid accumulation, large lipid droplets, and inflammatory cell infiltration. Liver sections from the E30L, E30H, E60L, and E60H groups showed localised microvesicular steatosis interspersed with occasional macrovesicular droplets (Figure 4B).

ORO staining showed greater hepatic lipid accumulation in the HFD group than in the NCD control group. All PSNE-treated groups exhibited significantly lower hepatic lipid deposition relative to the HFD model group; PSNE administration was associated with attenuated lipid deposition and reduced hepatocyte lipid droplet size compared with the HFD group (Figures 4C,D).

### 3.7 PSNE modulates the overall structural composition of the cecal microbiota

After quality filtering and chimera removal, 1,269,821 high-quality reads were obtained from 39 samples across the 13 experimental groups, with a mean of 32,560 ± 4,871 reads per sample. Rarefaction curves and diversity indices indicated that the sequencing depth captured the majority of bacterial diversity in all samples, thereby supporting robust downstream bioinformatic analyses (Supplementary Data Sheet 1; Supplementary Figures S3A-D). ASV analysis revealed that the NCD group harboured 313 unique ASVs, whereas the HFD, SIM, WL, WH, E30L, E30H, E60L, E60H, E90L, E90H, VOL, and VOH groups contained 111, 176, 147, 94, 105, 202, 149, 165, 141, 188, 103, and 114 unique ASVs, respectively. A core set of 15 ASVs was shared across all groups (Figure 5A).

**FIGURE 5:**
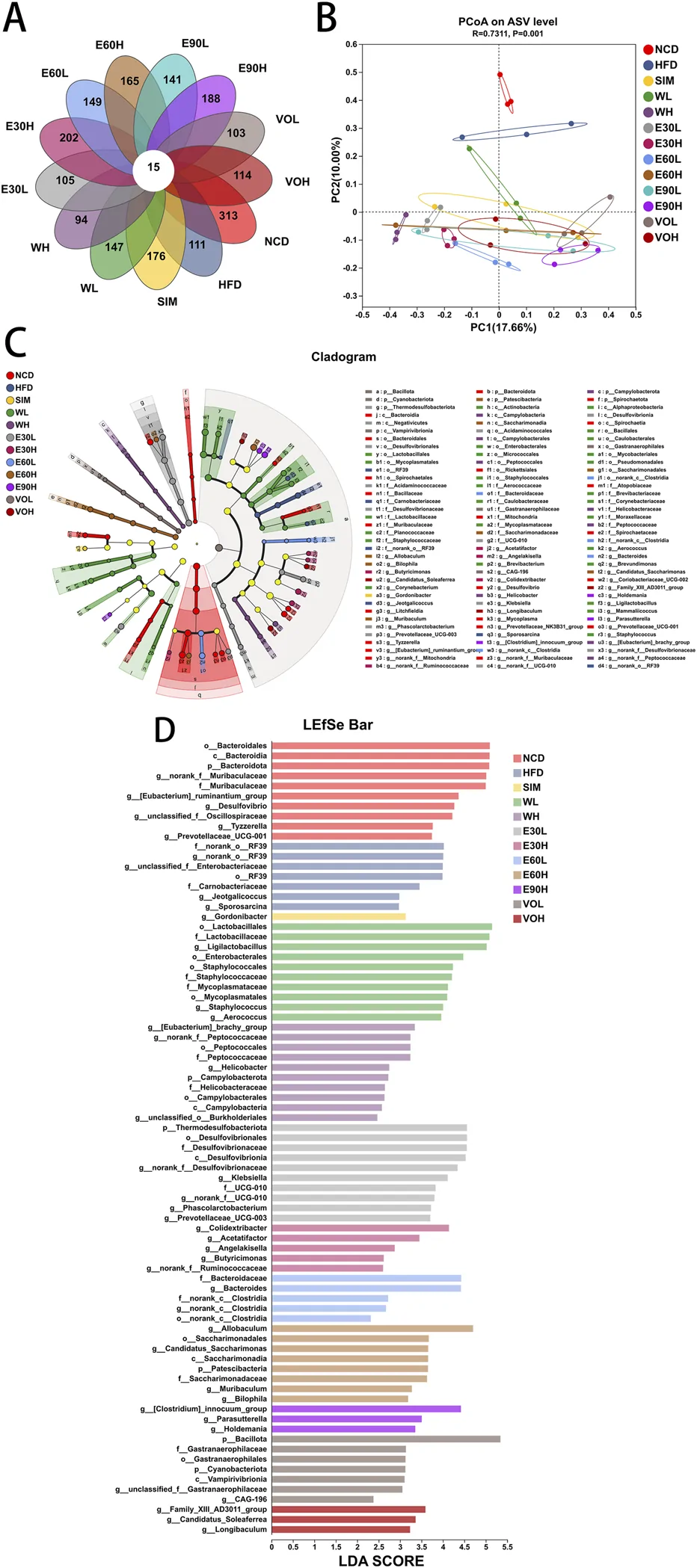
Effects of PSNE on cecal microbiota community structure. **(A)** Venn diagrams showing unique and shared ASVs among groups. **(B)** Weighted PCoA score plot of beta diversity. **(C)** LEfSe cladogram showing the taxonomic hierarchy of differentially abundant taxa, from the inner to outer rings: phylum, class, order, family, and genus. **(D)** LEfSe LDA score plot for biomarker taxa (LDA score >2). Group abbreviations and diets are the same as defined in the legend of Figure 1.

Alpha-diversity, assessed using the ACE index, Shannon diversity index and Chao1 richness index at the ASV level, revealed no statistically significant differences in cecal microbial community diversity or richness among the experimental groups (Supplementary Data Sheet 1; Supplementary Figures S3B-D). Nevertheless, the HFD group exhibited a discernible trend towards reduced diversity and richness compared with the NCD group, although this difference did not reach statistical significance. Beta-diversity analysis, performed using PCoA (Figure 5B) and nonmetric multidimensional scaling (NMDS) (Supplementary Data Sheet 1; Supplementary Figures S3E), revealed a clear separation between the HFD and NCD groups along the second principal coordinate, indicating that chronic HFD consumption altered the overall community structure of the cecal microbiota. PSNE-treated groups were distinctly separated from both the HFD and NCD groups, demonstrating that PSNE administration remodelled cecal microbiota composition under HFD-fed conditions (Figure 5B).

Differentially abundant bacterial taxa across the thirteen experimental groups were identified at taxonomic levels ranging from phylum to genus. A cladogram generated from LEfSe analysis highlighted taxa exhibiting differential enrichment among the groups (Figures 5C,D).

To further characterise the taxonomic composition of the cecal microbiota, the relative abundance of bacterial communities was assessed at the phylum level. The dominant phyla across all groups were Firmicutes, Bacteroidota, Thermodesulfobacteriota, Verrucomicrobiota*,* Pseudomonadota, and Actinomycetota (Figure 6A). PSNE administration prevented the HFD-induced increase in Firmicutes and decrease in Bacteroidota (Figure 6B). *Firmicutes* and Bacteroidota are the two most abundant phyla in both murine and human gut microbiota, and an elevated F/B ratio is consistently associated with obesity in both animal models and human cohorts compared with lean counterparts (Magne et al., 2020). Consistent with this association, the HFD group exhibited a significantly higher F/B ratio than the NCD group (*p* < 0.01), whereas this HFD-induced increase was attenuated in the WH, E30L, E30H, E60L, E60H, E90L, E90H, VOL, and VOH groups (*p <* 0.01), as well as in the SIM and WL groups (*p* < 0.05) (Figure 6C). Furthermore, HFD feeding significantly diminished the relative abundance of Thermodesulfobacteriota, an effect that was reversed by E30L administration (*p* < 0.01) (Figure 6D).

**FIGURE 6:**
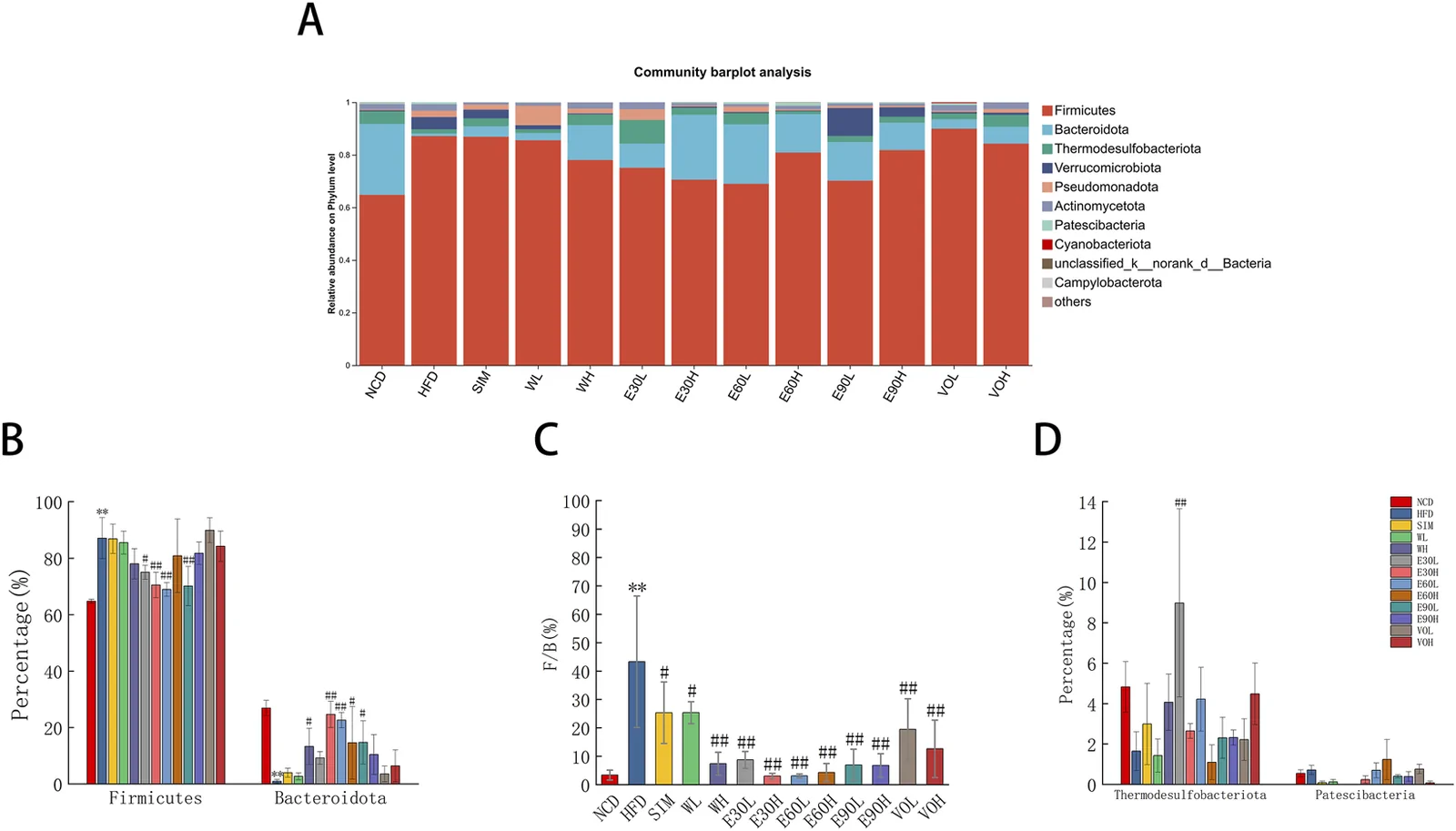
Effects of PSNE on phylum-level cecal microbiota composition. **(A)** Bar plot of relative phylum abundance. **(B)** Relative abundance of Firmicutes and Bacteroidota, **(C)** Firmicutes/Bacteroidota (F/B) ratio, **(D)** Relative abundance of Thermodesulfobacteriota and Patescibacteria. Data are expressed as the mean ± SD (*n* = 3). ^∗^ *p* < 0.05, ^∗∗^ *p* < 0.01 compared with the NCD group; ^#^ *p* < 0.05, ^##^ *p* < 0.01 compared with the HFD group. Group abbreviations and diets are the same as defined in the legend of Figure 1.

At the family level, the relative abundances of bacterial communities were assessed to characterise the taxonomic alterations induced by HFD and PSNE administration. The dominant families across all groups included Lachnospiraceae, norank_o__Clostridia_UCG-014, Muribaculaceae, Lactobacillaceae, Erysipelotrichaceae, Oscillospiraceae, Ruminococcaceae, Desulfovibrionaceae, [Eubacterium]_coprostanoligenes_group and Akkermansiaceae (Figure 7A). Compared with the NCD group, the HFD group showed a higher relative abundance of norank_o_RF39 (*p* < 0.05), Lachnospiraceae, norank_o__Clostridia_UCG-014, Erysipelotrichaceae and Saccharimonadaceae, together with a lower relative abundance of Muribaculaceae (p < 0.01), UCG-010 (p < 0.05), Oscillospiraceae, Lactobacillaceae, Ruminococcaceae, Desulfovibrionaceae, and Bacteroidaceae. PSNE administration counteracted these HFD-induced dysbiotic shifts. Specifically, PSNE administration significantly reduced the relative abundance of norank_o_RF39 (WH, p < 0.01; SIM, WL, E30L, E60H, E90L, E90H, and VOH, p < 0.05), norank_o__Clostridia_UCG-014 (WH, p < 0.05), and Saccharimonadaceae (WH and E30L, p < 0.05), while increasing the relative abundance of Muribaculaceae (E30H and E60L, p < 0.01; E60H, p < 0.05), UCG-010 (E30L, p < 0.01), Oscillospiraceae (WH, E30L, E30H, and E60H, p < 0.05), Desulfovibrionaceae (E30H, p < 0.01), and Bacteroidaceae (E60L, p < 0.01; WH and E30H, *p* < 0.05) (Figures 7B–G).

**FIGURE 7:**
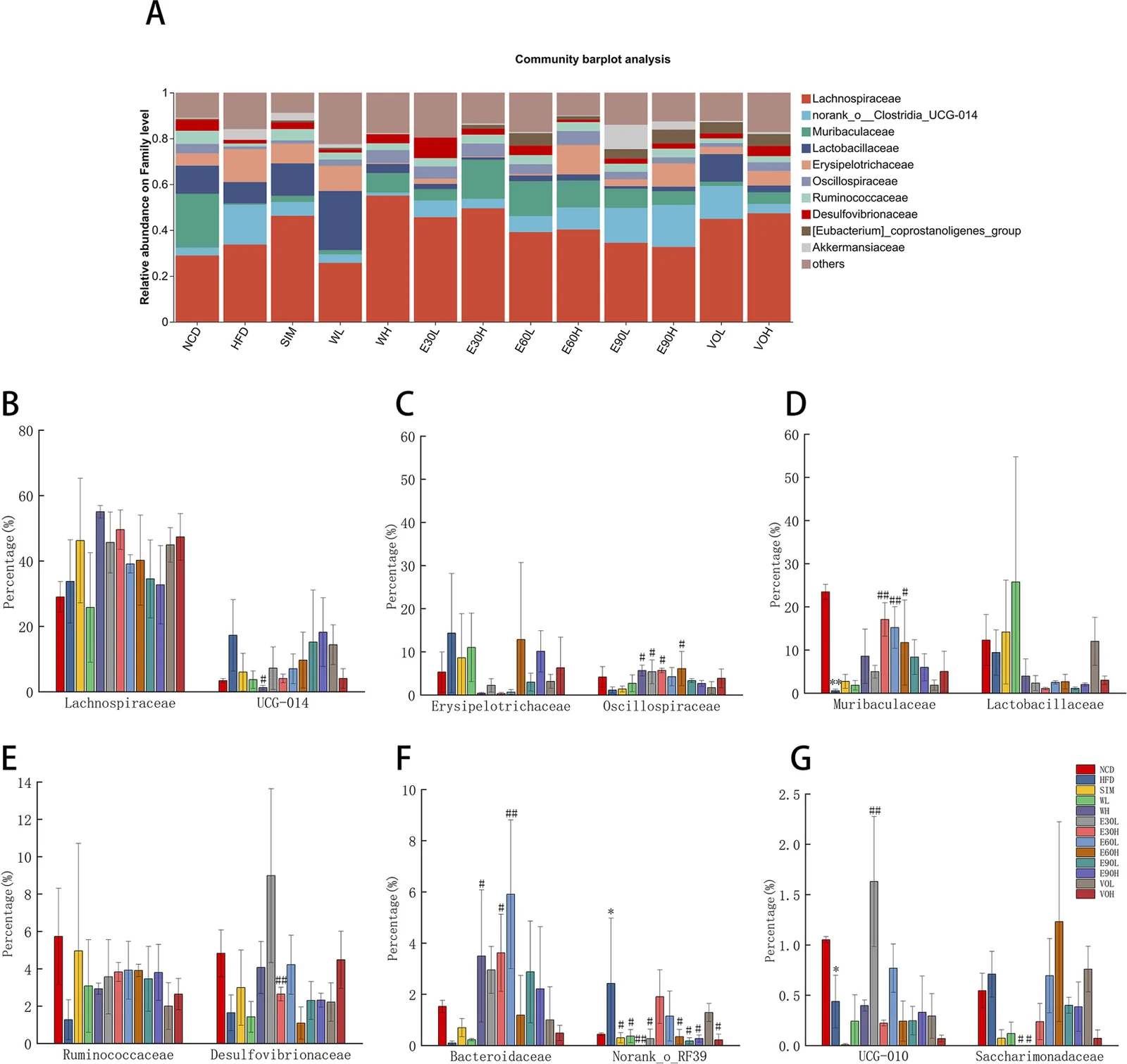
Effects of PSNE on family-level cecal microbiota composition. **(A)** Bar plot of relative family abundance. **(B)** Relative abundance of Lachnospiraceae and *norank_o__Clostridia_UCG-014,* **(C)** Erysipelotrichaceae and Oscillospiraceae, **(D)** Muribaculaceae and Lactobacillaceae, **(E)** Ruminococcaceae and Desulfovibrionaceae*,* **(F)** Bacteroidaceae and norank_o_RF39, **(G)** UCG-010 and Saccharimonadaceae. Data are expressed as the mean ± SD (*n* = 3). ^∗^ *p* < 0.05, ^∗∗^ *p* < 0.01 compared with the NCD group; ^#^ *p* < 0.05, ^##^ *p* < 0.01 compared with the HFD group. Group abbreviations and diets are the same as defined in the legend of Figure 1.

Further analysis was conducted to delineate the relative abundance profiles of bacterial communities at the genus level. The predominant genera detected across all groups comprised unclassified_f__Lachnospiraceae, Lachnospiraceae_NK4A136_group, norank_o__Clostridia_UCG-014, norank_f__Muribaculaceae, *Ligilactobacillus*, Lachnospiraceae_UCG-006, *Desulfovibrio*, norank_f__[Eubacterium]_coprostanoligenes_group, A*kkermansia,* and *Allobaculum* (Figure 8A). Compared with the NCD group, the HFD group exhibited an increased relative abundance of norank_o__RF39 (p < 0.01) and norank_o__Clostridia_UCG-014, accompanied by a reduction in the relative abundance of norank_f__Muribaculaceae (p < 0.01), *Desulfovibrio* (p < 0.01), *Ligilactobacillus*, Lachnospiraceae_NK4A136_group, *Bacteroides*, and *Colidextribacter*. PSNE administration counteracted these HFD-induced alterations. Specifically, PSNE administration significantly decreased the relative abundance of norank_o__RF39 (WH, p < 0.01; SIM, WL, E30L, E60H, E90L, E90H and VOH, p < 0.05) and norank_o__Clostridia_UCG-014 (WH, p < 0.05), while increasing the relative abundance of norank_f__Muribaculaceae (E30H and E60L, p < 0.01; E60H, p < 0.05), *Desulfovibrio* (WH, E30L, E60L and VOH, p < 0.05), *Ligilactobacillus*, Lachnospiraceae_NK4A136_group (E30H, p < 0.05), *Bacteroides* (E60L, p < 0.01; WH and E30H, p < 0.05), and *Colidextribacter* (E30H, *p* < 0.01, E30L, *p* < 0.05) (Figures 8B–E).

**FIGURE 8:**
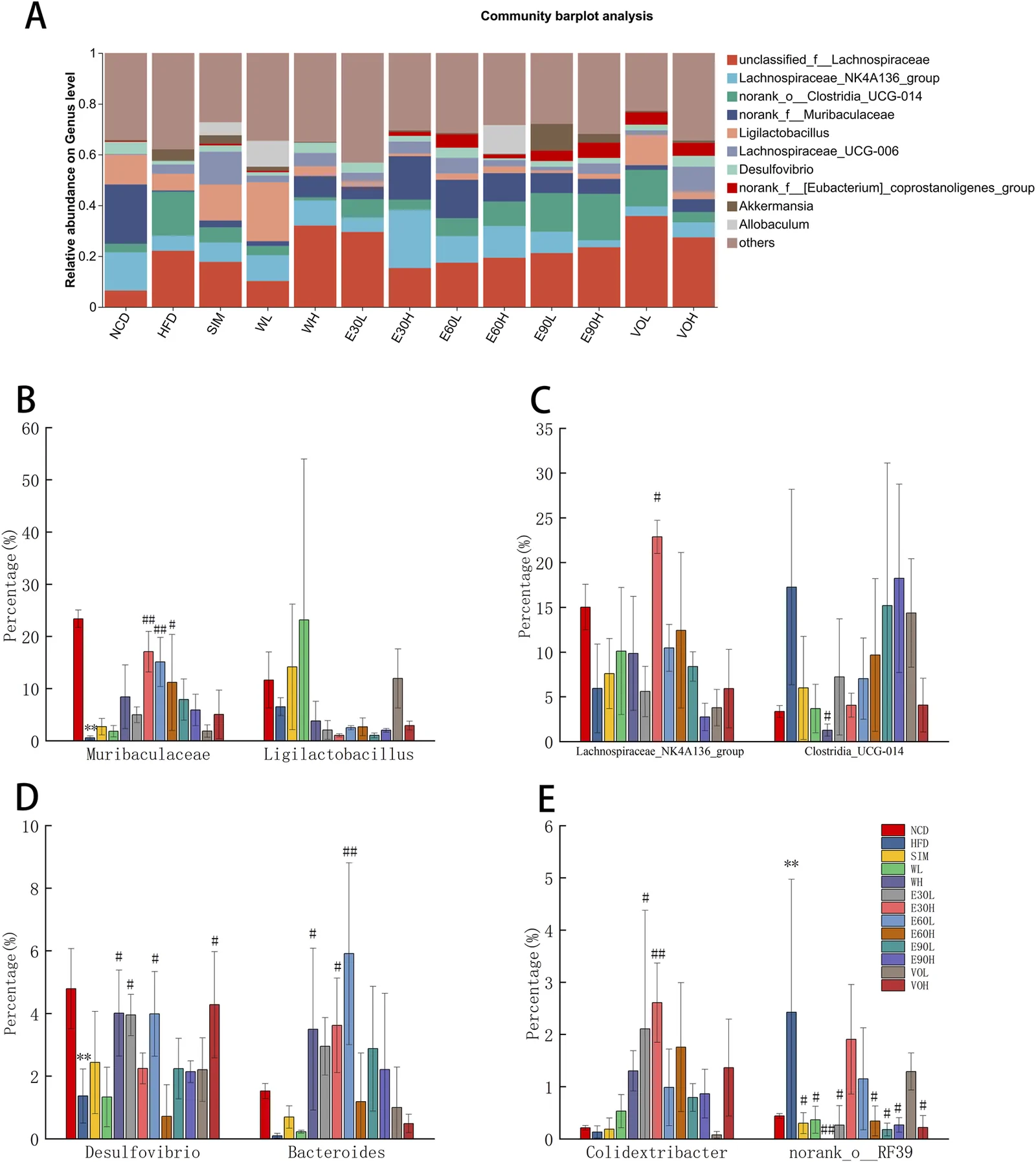
Effects of PSNE on genus-level cecal microbiota composition. **(A)** Bar plot of relative genus abundance. **(B)** Relative abundance of norank_f__ Muribaculaceae and *Ligilactobacillus*, **(C)** Lachnospiraceae_NK4A136_group and norank_o__Clostridia_UCG-014, **(D)** *Desulfovibrio* and *Bacteroides*, **(E)** *Colidextribacter* and norank_o__RF39. Data are expressed as the mean ± SD (*n* = 3). ^∗^ *p* < 0.05, ^∗∗^ *p* < 0.01 compared with the NCD group; ^#^ *p* < 0.05, ^##^ *p* < 0.01 compared with the HFD group. Group abbreviations and diets are the same as defined in the legend of Figure 1.

Heatmaps were generated using the MaAsLin platform (Majorbio). Furthermore, associations between cecal microbiota composition and PSNE-modulated biochemical indicators were assessed using Spearman’s rank correlation analysis. As shown in the correlation heatmap, norank_f__[Eubacterium]_coprostanoligenes_group, [Clostridium]_innocuum_group, *Adlercreutzia*, unclassified_f__Oscillospiraceae, *Ruthenibacterium*, NK4A214_group, Family_XIII_AD3011_group, and Christensenellaceae_R-7_group exhibited significant negative correlations with obesity-associated parameters, indicating potential protective roles against HFD-induced obesity. Network analysis further revealed that norank_f__[Eubacterium]_coprostanoligenes_group, [Clostridium]_innocuum_group, NK4A214_group, Family_XIII_AD3011_group, and Christensenellaceae_R-7_group were significantly negatively correlated with body weight and serum lipid profiles, including TC, TG, and LDL-C. Conversely, *Bacteroides*, *Blautia*, GCA-900066575, and unclassified_f__Enterobacteriaceae displayed significant positive correlations with these metabolic indices. *Blautia* and unclassified_f__Oscillospiraceae were significantly negatively correlated with hepatic enzyme activities, including ALT and AST, whereas norank_f__[Eubacterium]_coprostanoligenes_group and norank_o__RF39 exhibited significant positive correlations with these biomarkers. *Ligilactobacillus*, *Adlercreutzia*, and *Ruthenibacterium* were significantly negatively correlated with serum pro-inflammatory cytokines, including IL-6, IL-1β and TNF-α, whereas [Clostridium]_innocuum_group and *Blautia* were significantly positively correlated with these inflammatory mediators. Unclassified_f__Lachnospiraceae, norank_o__Clostridia_UCG-014, [Clostridium]_innocuum_group, norank_f__[Eubacterium]_coprostanoligenes_group, *Bacteroides*, and *Thomasclavelia* exhibited significant negative correlations with hepatic oxidative stress biomarkers including SOD, MDA, CAT and GSH-Px. Conversely, Lachnospiraceae_NK4A136_group, norank_f__Muribaculaceae, *Allobaculum*, *Blautia*, and norank_o__RF39 were significantly positively correlated with these oxidative stress indices (Figure 9).

**FIGURE 9:**
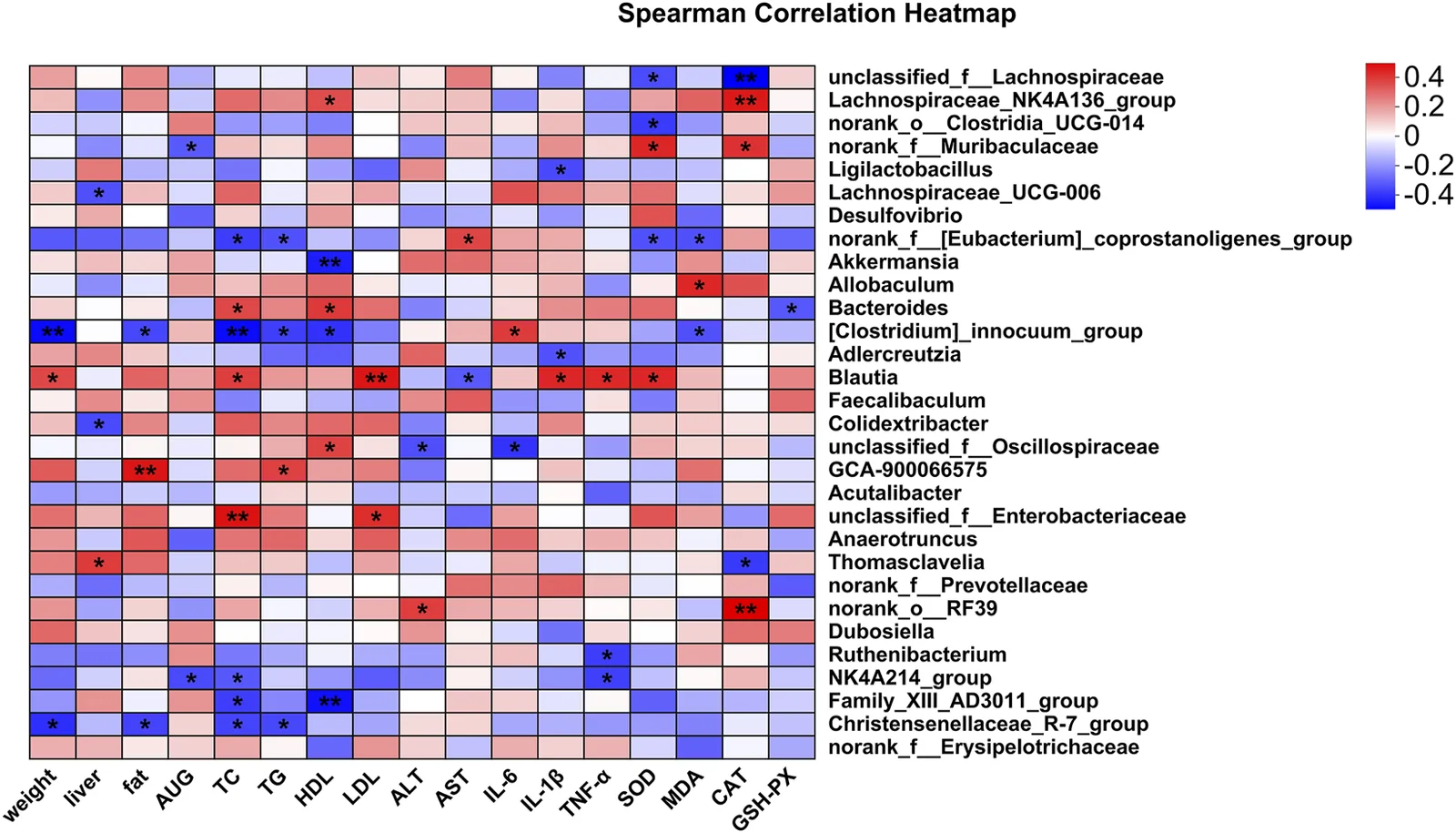
Spearman’s correlation heatmap between cecal microbiota taxa and obesity-related parameters. Colour intensity represents the degree of correlation, with red indicating positive correlations and blue indicating negative correlations. Significant correlations are marked by ^∗^ *p* < 0.05 and ^∗∗^ *p* < 0.01.

## 4 Discussion

The dose–response pattern observed in the pre-experiment merits careful interpretation. The low dose of PSNE (125 mg/kg) was sufficient to elicit near-maximal effects on most metabolic endpoints, while the medium dose (250 mg/kg) provided no additional improvement. This suggests a saturable or ceiling effect, likely reflecting saturation of intestinal absorption or target engagement at the low dose. Notably, the high dose (500 mg/kg) produced further numerical reductions in body weight gain, adipose tissue mass, and liver mass compared with the low dose, indicating that, for certain outcomes, a higher exposure can overcome partial saturation or engage additional pathways. Such non-linear dose–response relationships are common for multi-component plant extracts, where distinct active metabolites may differ in bioavailability, potency, or target accessibility. The medium dose therefore represented a pharmacologically redundant level, justifying its exclusion under the Reduction principle of the 3Rs, while the high-dose group was retained to capture these additional effects and ensure that the full dose–response range was explored.

Body weight gain and adipose tissue expansion were used as the primary phenotypic endpoints to evaluate the effects of PSNE administration in HFD-fed mice. Systemic lipid metabolism was further assessed through serum lipid profiling and histopathological examination of liver and adipose tissues. The liver plays a central role in maintaining glucose and lipid homeostasis. When energy intake chronically exceeds metabolic expenditure, ectopic lipid deposition occurs, resulting in hepatomegaly and pronounced adipose tissue expansion (Li et al., 2023). Adipose tissue, particularly white adipose tissue, not only stores and metabolises lipids but also functions as an active endocrine organ by secreting adipokines and other factors that systemically regulate lipid metabolism and inflammatory processes. Obesity-associated dyslipidaemia represents a major risk factor for a spectrum of cardiovascular diseases and metabolic disorders. Moreover, hypertrophic adipocytes, a hallmark of obesity, are key contributors to the development of dyslipidaemia (Preston et al., 2022).

TC is an essential component required for maintaining membrane integrity and for the synthesis of bile acids and steroid hormones (Attiq et al., 2024). In the present study, serum TC levels were significantly decreased in HFD-fed mice following PSNE administration, an effect likely attributable to enhanced hepatic TC utilisation and biliary excretion. LDL-C and HDL-C represent two major cholesterol-carrying lipoprotein fractions and are directly influenced by circulating TC and TG levels. A pronounced increase in hepatic and serum LDL-C concentrations substantially increases the risk of atherosclerosis, cardiovascular and cerebrovascular diseases, and other related complications (Taylor et al., 2024). Consistent with previous reports (Feng et al., 2022; Li et al., 2021b; Li et al., 2021c; Zhao et al., 2021), the present study showed that chronic HFD feeding significantly increased body weight, liver mass, and adipose tissue weight; elevated serum TC, TG, and LDL-C levels, suppressed HDL-C levels; and induced marked hepatic steatosis in mice.

ALT and AST are two critical transaminases predominantly localised within hepatocytes. Liver injury and hepatocyte degeneration impair the structural integrity of the hepatocellular membrane, leading to increased membrane permeability and subsequent release of ALT and AST into the circulation. Consequently, marked elevations in serum ALT and AST activities serve as reliable indicators of severe liver damage (Stapleton et al., 2020). Our results showed that PSNE administration effectively restored serum ALT and AST activities to near-normal levels, thereby mitigating HFD-induced hepatic damage. Moreover, PSNE intervention markedly ameliorated histopathological lesions in both adipose tissue and liver of HFD-induced obese mice. Collectively, these findings suggest that PSNE exerts lipid-lowering effects and can alleviate adipose tissue dysfunction and hepatic injury.

Obesity is associated with an increased risk of cardiovascular disease, and oxidative stress constitutes a key mechanistic link underlying this pathological process. Under physiological conditions, redox homeostasis is maintained through an intrinsic antioxidant defence network. In obesity, however, this delicate balance is profoundly disrupted, thereby resulting in systemic oxidative stress. MDA, a major end product of lipid peroxidation, serves as a hallmark indicator of oxidative injury (Mas-Bargues et al., 2021). Accordingly, the activities of SOD, CAT, and GSH-Px, together with MDA concentration, were selected as key indices for evaluating the antioxidant capacity of PSNE. Our results demonstrated that PSNE administration significantly alleviated HFD-induced oxidative stress, as evidenced by a marked reduction in MDA levels. Concurrently, PSNE administration substantially enhanced the activities of CAT, GSH-Px, and SOD in the liver of HFD-induced obese mice. Collectively, these findings suggest that PSNE effectively mitigates histological damage, oxidative stress, and lipid accumulation in HFD-induced obese mice. Further investigation is warranted to elucidate the *in vivo* antioxidant mechanisms and hepatoprotective effects of PSNE, thereby providing a scientific basis for its development as a safe and effective natural antioxidant.

Furthermore, chronic low-grade inflammation is closely associated with the progression of obesity and its related metabolic disorders. Sustained HFD feeding induces adipose tissue inflammation and promotes the release of pro-inflammatory cytokines, including IL-6 and TNF-α (Mandal et al., 2025). Accordingly, the present study examined the serum levels of key inflammatory cytokines in HFD-fed mice. Compared with the HFD group, PSNE administration significantly attenuated the serum concentrations of the pro-inflammatory cytokines IL-6, IL-1β, and TNF-α, thereby underscoring its potent anti-inflammatory capacity.

Although PSNE did not significantly restore α-diversity (ACE, Chao1, and Shannon indices), it induced a marked shift in β-diversity, reflecting a profound restructuring of the gut microbial community. This finding aligns with the notion that metabolic improvements are often driven by specific compositional changes—particularly the enrichment of beneficial taxa—rather than by an overall increase in species richness. The absence of α-diversity recovery may also be attributable to the relatively short intervention period or to the fact that HFD-induced dysbiosis did not severely depress α-diversity in this model. Therefore, the significant improvement in metabolic phenotypes despite unchanged α-diversity is biologically plausible.

In the present study, PSNE administration modulated the composition of the cecal microbiota in HFD-fed mice across multiple taxonomic levels, thereby restoring microbial homeostasis disrupted by chronic HFD consumption. Consistent with evidence derived from both human and rodent studies, obesity is closely associated with gut dysbiosis, a condition characterised by an elevated relative abundance of Firmicutes accompanied by a concomitant reduction in Bacteroidota (Rooks et al., 2014). Notably, PSNE intervention effectively reversed the HFD-induced elevation of the F/B ratio while increasing overall cecal microbial richness. Moreover, PSNE administration also restored the HFD-induced decline in the relative abundance of Thermodesulfobacteriota.

In murine models, remission of obesity-related metabolic disorders has been shown to coincide with an increase in the relative abundance of Muribaculaceae (Vezza et al., 2019). Notably, the HFD-induced reduction in Bacteroidota was predominantly driven by a decline in Muribaculaceae, which was restored to baseline levels following PSNE administration. Importantly, Muribaculaceae can degrade dietary polysaccharides, thereby producing short-chain fatty acids (SCFAs), including succinate, acetate, and propionate. SCFAs serve as key mediators linking the gut microbiota to host physiology owing to their established anti-inflammatory properties and critical role in maintaining glycolipid homeostasis (Li et al., 2020). Furthermore, chronic HFD feeding resulted in a marked decrease in the relative abundance of Bacteroidaceae and Ruminococcaceae, together with an increased abundance of Lachnospiraceae. PSNE administration significantly reversed these HFD-induced family-level alterations. Bacteroidaceae has been associated with cytokine production and may contribute to the suppression of autoimmune responses (Zhao et al., 2019). Ruminococcaceae has been linked to enhanced energy metabolism in mice (Menni et al., 2017), whereas an elevated abundance of Lachnospiraceae is associated with increased butyric acid production (Berger et al., 2021).

At the genus level, PSNE administration ameliorated HFD-induced gut dysbiosis, shifting the composition of the cecal microbiota towards that of the NCD group primarily through increasing the relative abundance of *Bacteroides*. *Bacteroides* species are metabolically versatile and capable of degrading both carbohydrates and proteins through enzymes involved in glycolysis and the pentose phosphate pathway. *Lactobacillus*, a genus widely distributed across various mammalian tissues, including the digestive, urinary, and reproductive tracts, is well recognised for its beneficial properties. It can correct gut microbiota dysbiosis, maintain microbial homeostasis, suppress the progression of obesity, and reduce serum cholesterol levels. Consistent with these beneficial effects, the relative abundance of *Lactobacillus* was higher in the WL group than in the HFD group.

Within the phylum Firmicutes, the relative abundance of the Lachnospiraceae_NK4A136_group declined substantially following 15 weeks of HFD feeding, a change that was significantly reversed by PSNE administration. The Lachnospiraceae_NK4A136_group, a member of the Lachnospiraceae family, shares the characteristic anaerobic metabolism and spore forming capacity of this taxonomic group. It ferments plant polysaccharides to produce SCFAs and ethanol. This group has demonstrated efficacy in the prevention and treatment of acute colitis (Wang et al., 2019). As a butyrate-producing bacterium, it contributes to intestinal mucosal protection and attenuates host-mediated inflammation (Li et al., 2021a). Human studies have implicated the phylum Proteobacteria as a potential biomarker for non-alcoholic fatty liver disease (NAFLD). In murine models, prebiotics alleviate HFD-induced NAFLD through modulation of Proteobacteria, specifically Betaproteobacteria, Deltaproteobacteria, particularly the order Desulfovibrionales, and Epsilonproteobacteria, including the genus Firmicutes (Porras et al., 2017). Prebiotics maintain intestinal health through regulation of the gut microbiota, particularly at the phylum Firmicutes (Zheng et al., 2018). Consistent with these findings, PSNE administration (E60H) effectively counteracted the HFD-induced increase in the relative abundance of *Desulfovibrio* in HFD-fed mice. Collectively, these findings suggest that the anti-obesity effects of PSNE in HFD-induced obesity may be attributable to the restoration of these key beneficial taxa.

While our correlation analyses revealed significant associations between gut microbial taxa and host metabolic parameters, these findings are observational and cannot establish a direct causal relationship. Significant associations were observed between specific bacterial taxa and a range of obesity-related metabolic parameters, including glucose tolerance, serum lipid profiles, fat accumulation, oxidative stress indices, and inflammatory markers. The taxa exhibiting these robust correlations included norank_f__[Eubacterium]_coprostanoligenes_group, [Clostridium]_innocuum_group, NK4A214_group, Family_XIII_AD3011_group, Christensenellaceae_R-7_group, *Bacteroides*, *Blautia*, GCA-900066575, unclassified_f__Enterobacteriaceae, *Ligilactobacillus*, *Adlercreutzia*, unclassified_f__Oscillospiraceae, *Ruthenibacterium*, norank_o__RF39, unclassified_f__Lachnospiraceae, norank_o__Clostridia_UCG-014, *Thomasclavelia*, Lachnospiraceae_NK4A136_group, norank_f__Muribaculaceae, and *Allobaculum*. Consistent with previous reports, *Akkermansia* was negatively correlated with HDL-C, and norank_f_[Eubacterium]_coprostanoligenes_group was negatively correlated with TC and TG. Among hepatic and metabolic markers, *Blautia* showed a positive correlation with TC (Fu et al., 2021); *Blautia* also exhibited negative correlations with ALT and AST, whereas Erysipelotrichaceae was positively correlated with mouse body weight and ALT (Gui et al., 2019). In relation to oxidative stress and inflammation, Erysipelotrichaceae and *Bacteroides* were negatively correlated with GSH-Px (Ma et al., 2019), while *Bacteroides* showed positive correlations with the pro-inflammatory cytokines IL-6 and TNF-α (Wan et al., 2022). Collectively, these findings highlight the aforementioned taxa as potential microbial candidates that may be linked to the host metabolic responses against HFD-induced obesity progression. However, the precise mechanistic pathways through which these bacterial candidates exert their effects remain to be elucidated.

Although monoterpene alcohols in VOL exhibit low oral bioavailability, the anti-obesity and anti-inflammatory effects observed here are consistent with the multi-target actions reported for other essential oils. For instance, the essential oil from the pericarp of *Zanthoxylum bungeanum* Maxim. (ZBEO), whose main components include atractylon, D-limonene, and γ-elemene, has been shown to reduce body weight and systemic inflammation through both peripheral and central mechanisms (Ye et al., 2025). ZBEO suppressed appetite by regulating the hypothalamic AMPK/NPY/POMC/CART pathway, ameliorated glucose and lipid metabolism, and attenuated inflammation *in vivo* and *in vitro*. Furthermore, ZBEO modulated the gut microbiota composition and function, as well as metabolic pathways such as unsaturated fatty acid biosynthesis, biotin metabolism, and pyrimidine metabolism. These findings demonstrate that a volatile oil with similar monoterpene constituents can produce significant systemic metabolic benefits by integrating local intestinal effects with central appetite control. We therefore speculate that VOL/VOH may act through analogous mechanisms: locally modulating the gut microbiota and reducing intestinal inflammation, while centrally suppressing appetite *via* gut-brain axis signalling, thereby collectively contributing to its anti-obesity effects despite the limited bioavailability of individual components.

Despite these promising findings, several limitations should be acknowledged. First, the absence of chemical characterisation of the PSNE fractions limits the attribution of the observed effects to specific metabolite classes. Comprehensive phytochemical profiling (e.g., HPLC/GC-MS fingerprinting and marker quantification) is therefore an essential next step to ensure reproducibility and align with the ConPhyMP guidelines. Second, while our correlation analyses revealed significant associations between gut microbial taxa and host metabolic parameters, these findings are observational and cannot establish causality. Third, although we discussed the potential role of SCFA-producing taxa (e.g., Muribaculaceae and Ruminococcaceae) based on previously reported functions, we did not directly measure SCFA concentrations in cecal contents or faeces, nor did we perform metagenomic or metabolomic analyses. Therefore, the proposed link between PSNE-modulated microbiota and SCFA-mediated metabolic improvement remains speculative. Fourth, several statistical limitations should also be acknowledged. Fisher’s LSD *post hoc* test was used for exploratory comparisons without correction for multiple comparisons, and effect sizes (e.g., Partial η^2^ for ANOVA or Cohen’s d for pairwise comparisons) were not calculated. All significant differences identified should therefore be interpreted as hypothesis-generating rather than confirmatory, and future studies with appropriate corrections (e.g., Tukey’s HSD or FDR) and effect size estimates are required to validate these findings.

To address these limitations, future studies should prioritise: (i) chemical characterisation of PSNE to identify its active constituents, including HPLC/GC-MS fingerprinting, marker compound quantification, and assessment of batch-to-batch consistency to ensure reproducibility; (ii) functional validation of the microbiota–host interplay using germ-free animal models, faecal microbiota transplantation (FMT), or targeted antibiotic depletion to definitively establish causality; (iii) targeted quantification of SCFA in cecal contents or faeces, as well as metagenomic or metabolomic profiling, to directly test the hypothesis that PSNE modulates glycolipid metabolism through SCFA-producing bacteria; (iv) direct measurement of circulating LPS, intestinal tight junction proteins (ZO-1 and Occludin), and portal inflammatory markers to functionally validate the hypothesized improvement in gut barrier integrity and reduction of endotoxin translocation; and (v) confirmatory studies with appropriate multiple comparison corrections (e.g., Tukey’s HSD or FDR) and effect size estimates (e.g., Partial η^2^ or Cohen’s d) to validate the statistical robustness and biological magnitude of the observed effects. Beyond causality, mechanistic investigations are warranted to elucidate the underlying molecular pathways—both at the microbial level and at the host level.

Collectively, such studies will advance the current understanding of the therapeutic potential of PSNE and provide a foundation for the development of novel dietary supplements and targeted therapeutic strategies aimed at ameliorating metabolic disorders associated with gut microbiota dysbiosis.

## 5 Conclusion

In summary, the present study suggests that PSNE exerts multifaceted anti-obesity effects in HFD-fed mice. Specifically, PSNE significantly attenuated blood lipid levels, ameliorated hepatic steatosis and liver injury, and reduced the expression of key inflammatory mediators. Furthermore, PSNE administration was associated with remodelling the HFD-induced alterations in the composition and structure of the cecal microbiota. Notably, the distinct pine needle extracts evaluated in this study exhibited differential regulatory capacities in counteracting HFD-induced cecal microbiota dysbiosis, thereby underscoring the complexity of their interactions with the gut microbiome. These findings provide a preliminary basis for the potential use of PSNE in obesity management; however, as an initial exploratory study, further chemical characterisation and mechanistic validation are required to confirm these observations.

## Data Availability

The datasets presented in this study can be found in online repositories. The names of the repository/repositories and accession number(s) can be found below: https://www.cncb.ac.cn, CRA041990.

## Supplementary material

SUPPLEMENT DATA SHEET 1 Supplementary Table: Sample sizes (n) for all measured parameters in each group (Figures 1–3). Supplementary Figure S1: iWAT from mice in each group. Supplementary Figure S2: Liver tissue from mice in each group. Supplementary Figure S3: Rarefaction curves of the Sobs index of cecal flora at the ASV level. Alpha diversity was evaluated by the ACE index, Shannon index and Chao index.

SUPPLEMENT DATA SHEET 2 Administrative Licensing Decision Approving Entry into the Nature Reserve to Conduct Botanical Scientific Research on Pinus squamata X.W. Li.

SUPPLEMENT DATA SHEET 3 ConPhyMP checklist of information for reporting plant material and its initial processing.

SUPPLEMENT DATA SHEET 4 ConPhyMP checklist of items for conducting and reporting analytical methods1,2 relevant for extract type C.

SUPPLEMENT DATA SHEET 5 GC-MS total ion chromatogram (TIC) of the volatile oil from *Pinus squamata* X.W. Li.

SUPPLEMENT TABLE 1 Complete pairwise comparison results for all measured parameters (body weight, TC, TG, HDL-C, LDL-C, ALT, AST, IL-6, IL-1β, TNF-α, SOD, MDA, CAT, GSH-Px, AUC, liver weight, iWAT weight, etc.) following one-way ANOVA with Fisher’s LSD post hoc test. For each comparison, the table reports: mean difference (I-J), standard error, exact P-value, and 95% confidence interval (lower and upper bounds). Asterisks (*) indicate statistically significant differences at p < 0.05.

SUPPLEMENT TABLE 2 Individual data values for Figures 1–3.

SUPPLEMENT TABLE 3 Chemical constituents of the volatile oil from *Pinus squamata* X.W. Li.

SUPPLEMENTARY IMAGE 1 On-site photographs of the collection site confirming cultivated origin.
