# Supplementary material for: Needle extracts of Pinus squamata X.W. Li ameliorate obesity and modulate cecal microbiota in high-fat diet-induced obese mice
Source: Front Pharmacol. 2026 Sep 22;17:1906430. doi: 10.3389/fphar.2026.1906430 (PMC13638554; doi:10.3389/fphar.2026.1906430)
Supplement: Supplementary file 1 [file DataSheet1.pdf]

Supplementary Material

Supplementary Table

Sample sizes (n) for all measured parameters in each group (Figures. 1-3)

n	Final weight	iWAT	Liver	OGTT	AUC	TC	TG	LDL-C	HDL-C	ALT	AST	SOD	GSH-PX	CAT	MDA	IL-6	IL-1β	TNF-α
NCD	5	5	5	6	6	4	5	4	5	5	4	5	5	4	3	5	3	5
HFD	9	6	7	7	7	5	6	6	6	7	4	5	6	5	4	6	8	5
SIM	11	11	11	7	7	5	7	10	6	10	9	8	9	6	6	5	5	7
WL	8	8	8	4	4	6	8	6	6	7	8	6	6	6	3	7	5	6
WH	5	5	5	4	4	5	4	5	5	5	5	4	5	4	3	4	4	5
E30L	4	4	4	4	4	3	3	3	3	4	3	4	4	4	3	4	4	3
E30H	5	5	5	5	5	4	5	5	4	5	3	5	4	5	4	4	5	4
E60L	7	7	7	5	5	4	5	5	6	7	5	5	5	5	5	5	6	6
E60H	7	7	7	4	4	3	7	6	7	7	5	6	6	6	3	5	6	6
E90L	7	7	7	5	5	4	7	6	7	5	5	6	5	4	3	7	6	7
E90H	5	5	5	5	5	3	5	5	5	5	3	5	4	4	3	4	4	5
VOL	5	5	5	5	5	3	5	5	5	5	5	4	4	4	4	5	4	5
VOH	5	5	5	5	5	4	5	5	5	5	3	4	4	4	4	3	5	4

Supplementary Figures

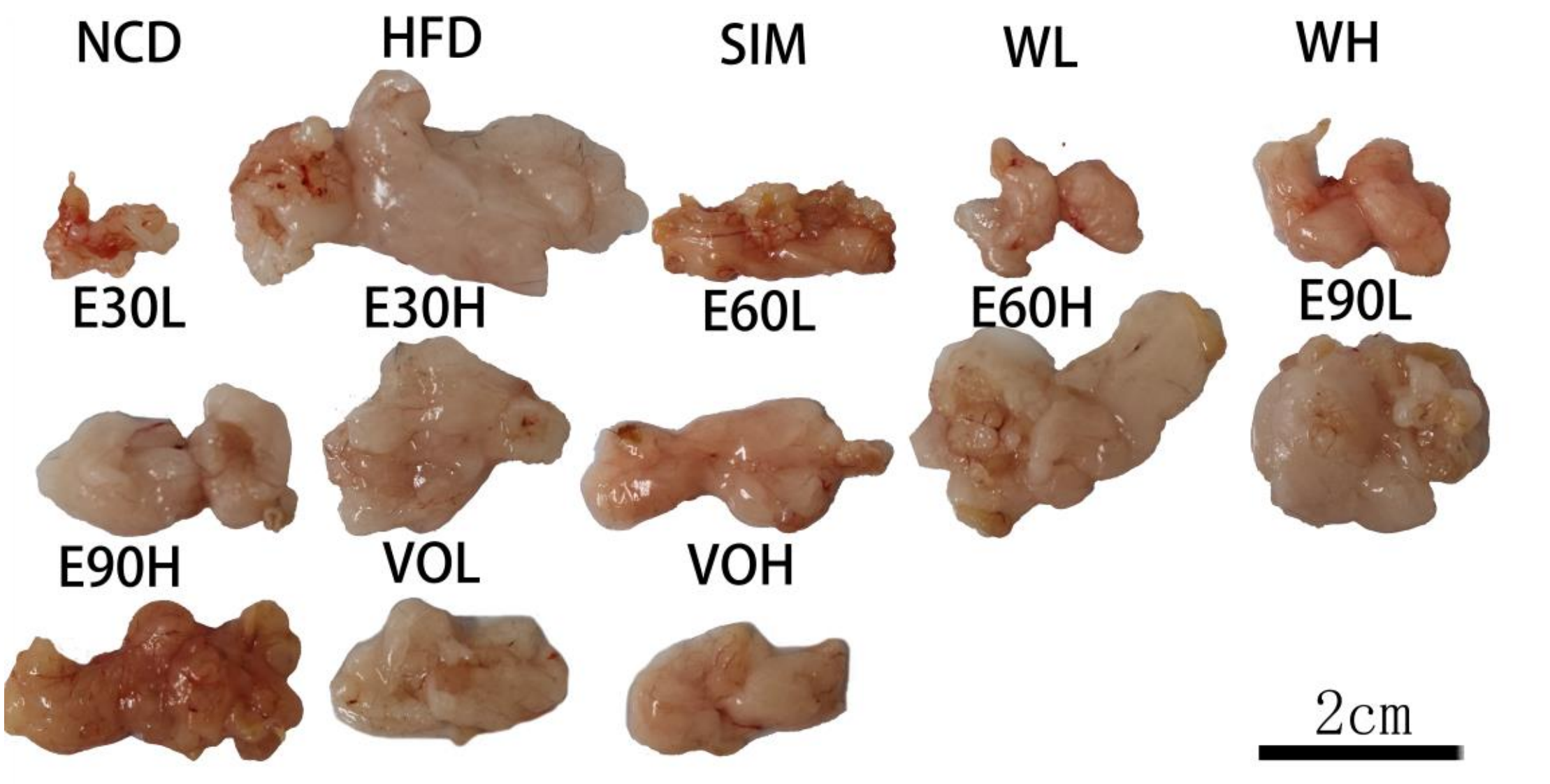

Supplementary Figure 1. Inguinal white adipose tissue (iWAT) from mice in each group.

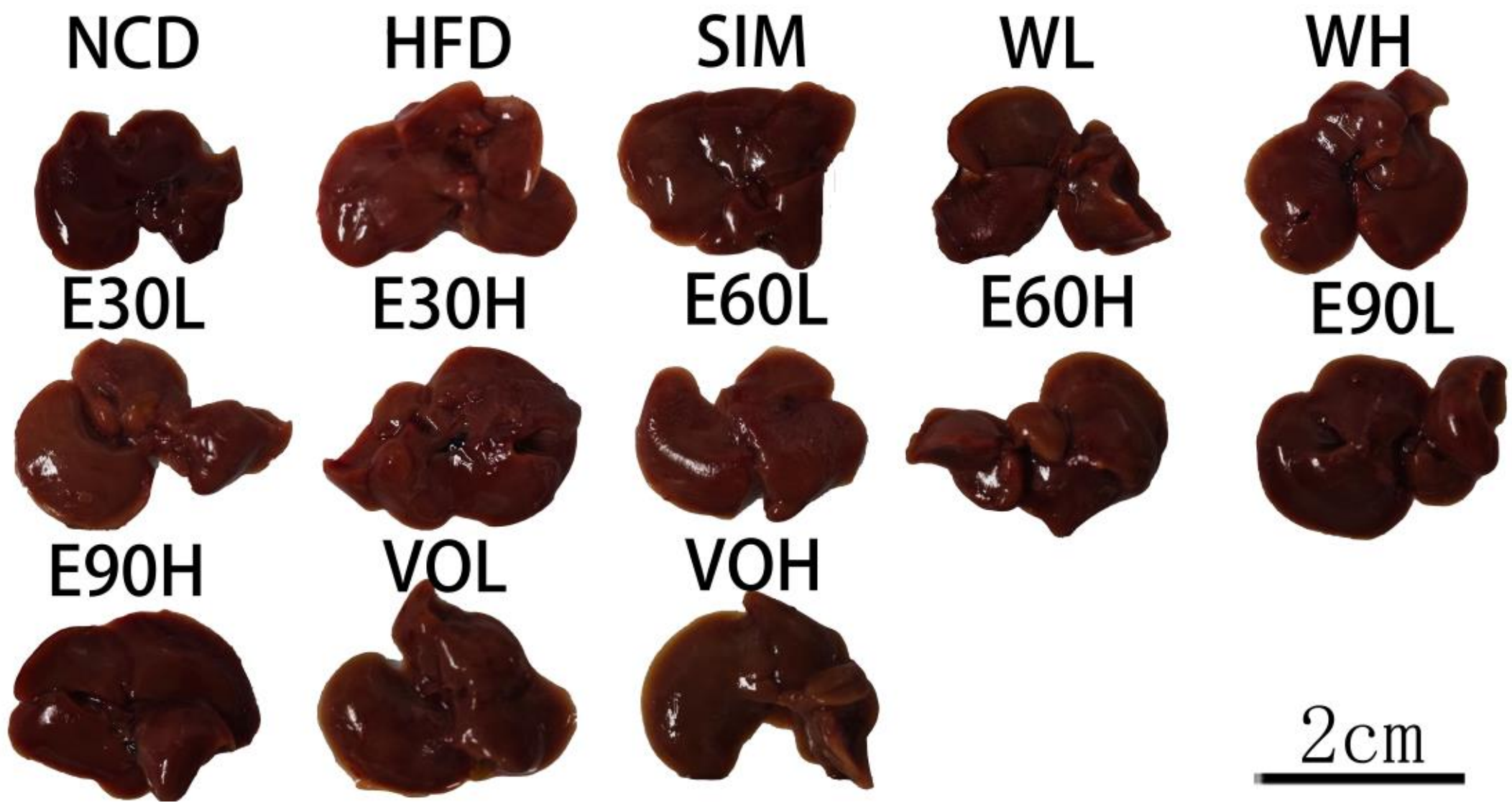

Supplementary Figure 2. Liver tissue from mice in each group

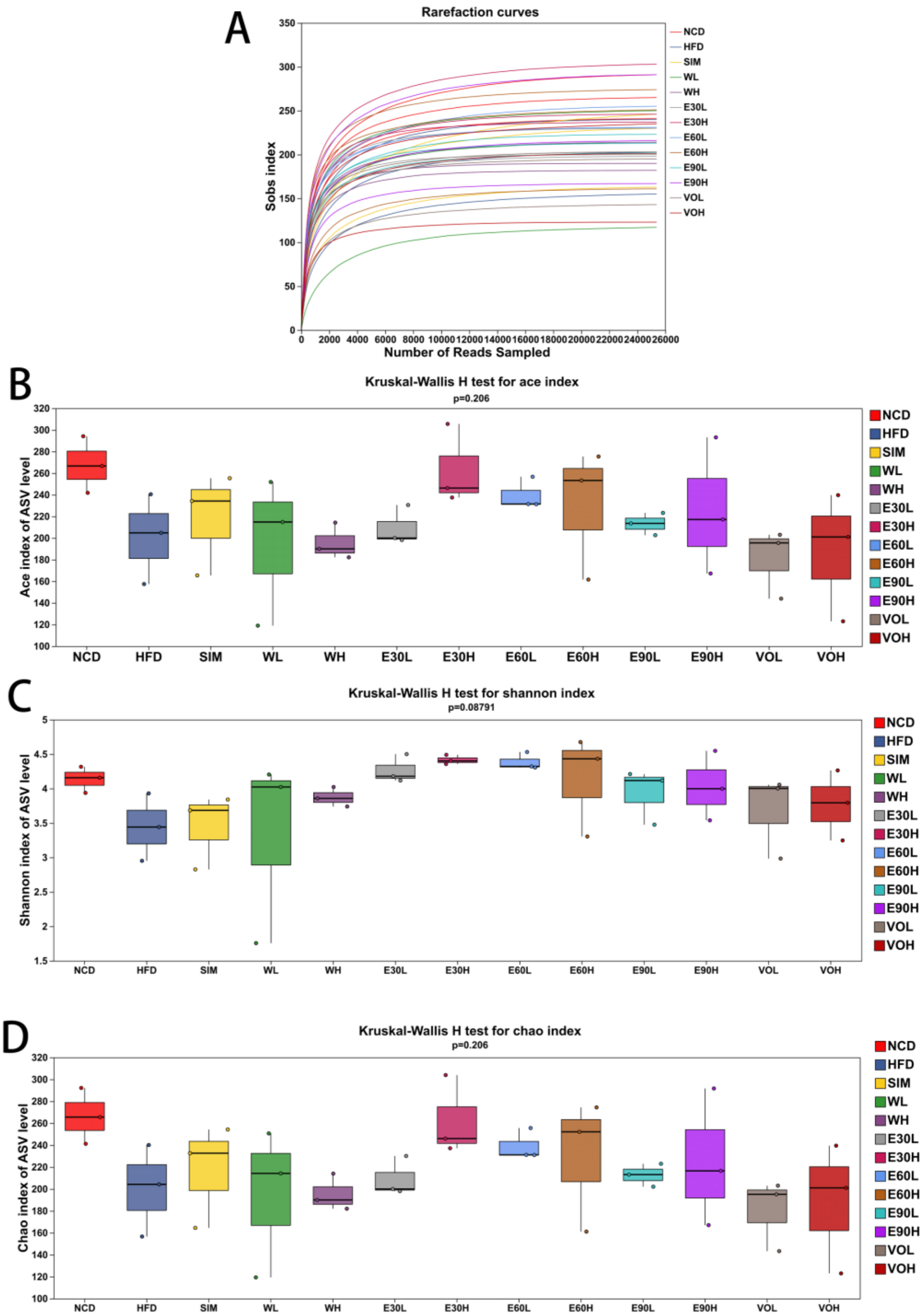
A
Rarefaction curves
Sobs index
Number of Reads Sampled
NCD
HFD
SIM
WL
WH
E30L
E30H
E60L
E60H
E90L
E90H
VOL
VOH
B
Kruskal-Wallis H test for ace index
p=0.206
Ace index of ASV level
C
Kruskal-Wallis H test for shannon index
p=0.08791
Shannon index of ASV level
D
Kruskal-Wallis H test for chao index
p=0.206
Chao index of ASV level

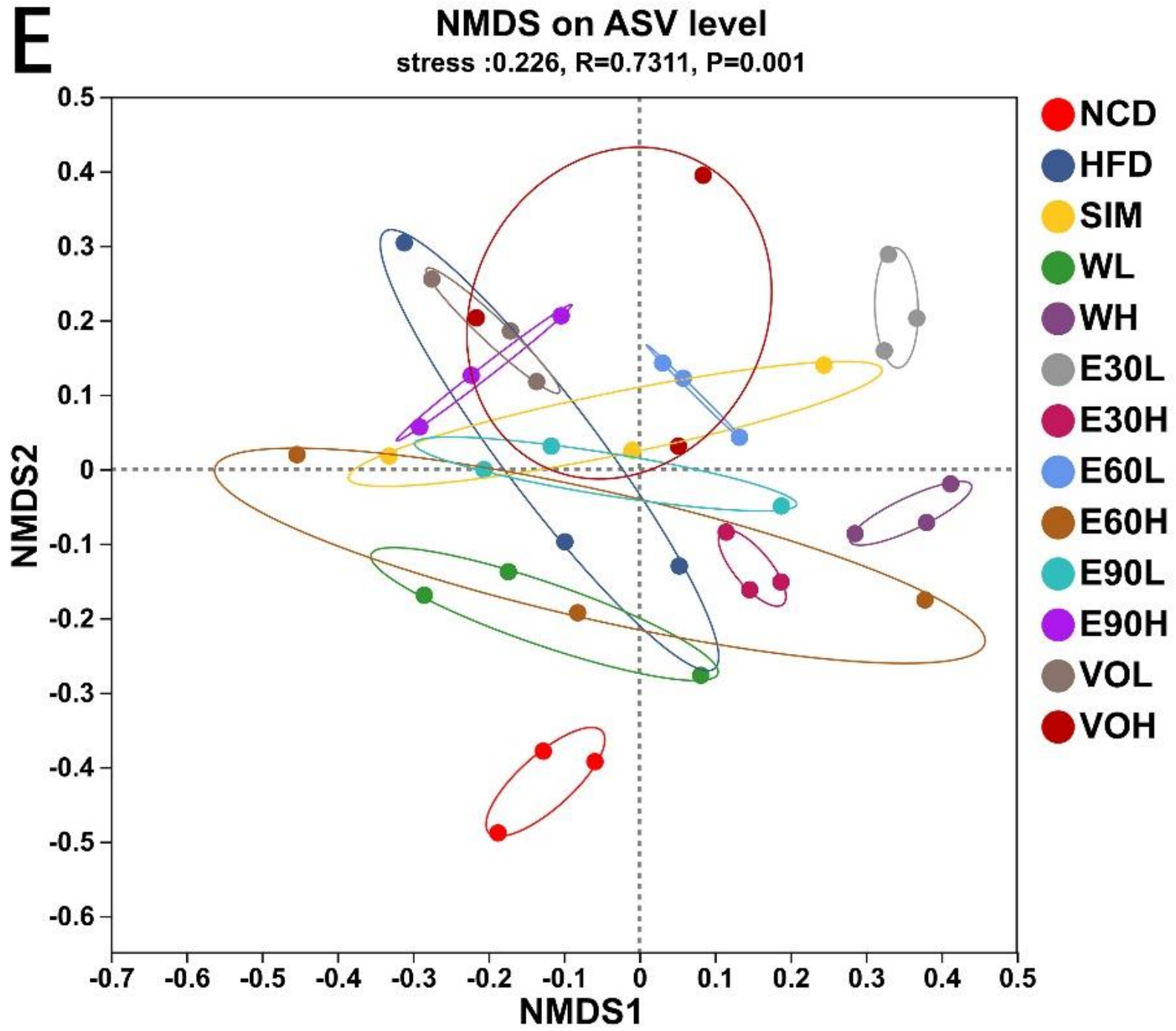

Supplementary Figure 3. Cecal microbiota diversity and community structure. Rarefaction curves of Sobs index of cecal microbiota at the ASV level(A). Alpha diversity was evaluated by Ace index (B), Shannon index(C) and chao index (D). NMDS ordination of cecal microbiota community structure (E).
