# Supplementary material for: Needle extracts of Pinus squamata X.W. Li ameliorate obesity and modulate cecal microbiota in high-fat diet-induced obese mice
Source: Front Pharmacol. 2026 Sep 22;17:1906430. doi: 10.3389/fphar.2026.1906430 (PMC13638554; doi:10.3389/fphar.2026.1906430)
Supplement: Supplementary file 2 [file DataSheet2.pdf]

昭通市林业和草原局

准予行政许可决定书

昭市林审许准〔2023〕1 号

昭通市林业和草原局关于准予贵州医科大学常帅等五人进入自然保护区开展植物科考研究的行政许可决定

贵州医科大学：

你单位关于进入云南药山国家级自然保护区开展植物科考等工作的申请及相关材料收悉。申报材料我局通过初步审核，经研究，决定如下：

一、同意你单位常帅、王天爽、任玉林、卢雪、喻甜五名科研人员进入云南药山国家级自然保护区开展五针松（Pinus suqamata）研究工作，具体科考时间为 2023 年 2 月 1日 - 2024 年 6 月 30 日，调查地点及线路由巧家药山国家级自然保护区管护局根据情况安排。

二、本次调查工作如果涉及标本采集，调查工作开始前要将拟采集物种名录和数量送巧家药山国家级自然保护区管护局审查，经保护区管护局同意后采集并将研究成果抄送保护区管护局

留存。如需采集国家和省级重点保护野生动植物物种，应办理相关行政许可手续。

三、在野外工作中必须遵守国家有关法律、法规，做好人身安全、生物安全和森林防火等相关工作。

昭通市林业和草原局

2023 年 1 月 16 日

抄 报：云南省林业和草原局；　　　发：巧家县林业和草原局

昭通市林业和草原局办公室　　　　2023 年 1 月 6 日印
