# Supplementary material for: Needle extracts of Pinus squamata X.W. Li ameliorate obesity and modulate cecal microbiota in high-fat diet-induced obese mice
Source: Front Pharmacol. 2026 Sep 22;17:1906430. doi: 10.3389/fphar.2026.1906430 (PMC13638554; doi:10.3389/fphar.2026.1906430)

样品信息

分析者	: Admin
分析日期	: 2024/09/11 10:13:34
样品类型	: 未知物
级别号	: 1
样品名	: OX3
样品 ID	:
内标量	: [1]=1
样品量	: 1
稀释因子	: 1
瓶号	: 1
进样体积	: 1.00
数据文件	: E:\数据\王洽\OX3.qgd
原始数据文件	: E:\数据\王洽\OX3.qgd
方法文件	: E:\数据\王洽\液体进样0911.qgm
原始方法文件	: E:\数据\王洽\液体进样0911.qgm
报告文件	:
调谐文件	: E:\调谐\调谐20240704.qgt
修改者	: Admin
修改日期	: 2024/09/11 11:50:35

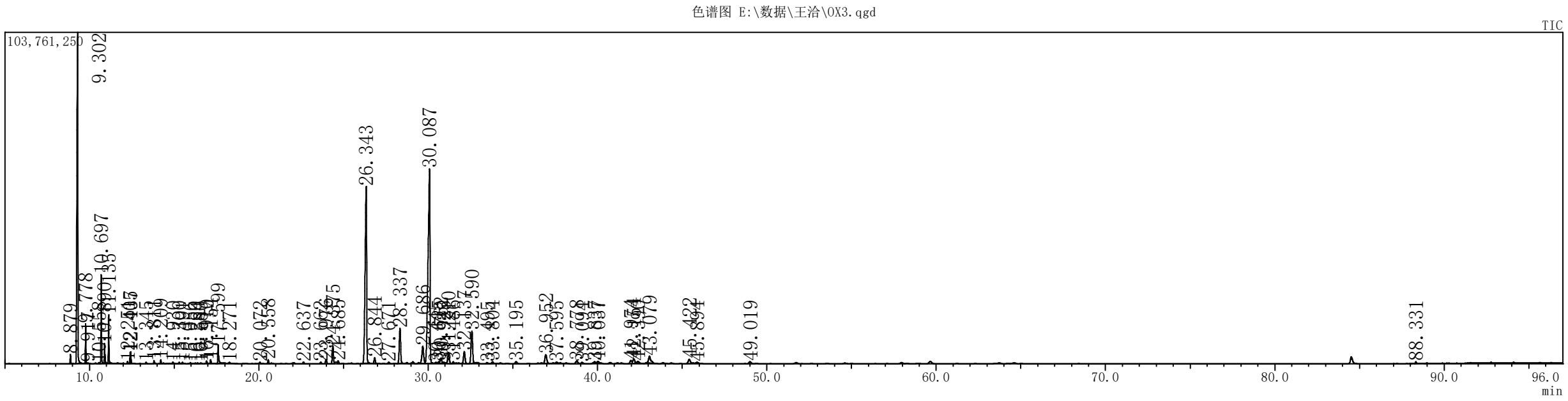

峰表 TIC

峰号	保留时间	I.Time	F.Time	峰面积	峰面积%	峰高	峰高%	积/高度	标记	名称
1	8.879	8.825	8.950	6922767	0.37	2925617	0.79	2.37		Tricyclo[2.2.1.0(2,6)]
2	9.302	9.180	9.500	427745406	22.64	103613490	28.00	4.13		3-Octen-5-yne, 2,7-dim
3	9.778	9.715	9.860	28834938	1.53	12411202	3.35	2.32		Camphene
4	9.917	9.860	9.955	476989	0.03	213195	0.06	2.24	V	Thuja-2,4(10)-diene
5	10.558	10.520	10.610	736077	0.04	314771	0.09	2.34		Sabinene
6	10.697	10.625	10.820	68337468	3.62	27800866	7.51	2.46		Pinene <beta->
7	10.890	10.820	11.010	15550549	0.82	6245667	1.69	2.49	V	trans-2-Hexenal dimeth
8	11.135	11.010	11.240	35445790	1.88	15165609	4.10	2.34	V	Myrcene
9	12.251	12.205	12.315	1821860	0.10	739808	0.20	2.46		Cymene <para->
10	12.405	12.315	12.420	7782813	0.41	3530265	0.95	2.20	V	1,5-Cyclooctadiene, 3,
11	12.437	12.420	12.540	8476309	0.45	3679215	0.99	2.30	V	.alpha.-Phellandrene
12	13.345	13.300	13.405	1137567	0.06	466814	0.13	2.44		.gamma.-Terpinene
13	13.811	13.770	13.865	2001969	0.11	869347	0.23	2.30		3-Cyclohexene-1-methan
14	14.209	14.160	14.275	2919424	0.15	1192586	0.32	2.45		(+)-4-Carene
15	14.930	14.885	14.985	1083261	0.06	439470	0.12	2.46		Benzaldehyde dimethyl
16	15.300	15.260	15.360	994216	0.05	391150	0.11	2.54		Fenchyl alcohol
17	15.491	15.445	15.550	1097065	0.06	422029	0.11	2.60		Campholenic aldehyde <
18	15.956	15.920	16.010	699428	0.04	283046	0.08	2.47		Pinocarveol <trans->
19	16.250	16.225	16.355	439696	0.02	157472	0.04	2.79		p-Mentha-1,5-dien-8-ol
20	16.384	16.355	16.435	537018	0.03	222059	0.06	2.42	V	Camphene hydrate
21	16.580	16.545	16.625	434349	0.02	200283	0.05	2.17		Pinocarvone
22	16.910	16.865	16.970	2027124	0.11	806436	0.22	2.51		endo-Borneol
23	17.154	17.105	17.230	3202756	0.17	1198143	0.32	2.67		3-Cyclohexen-1-ol, 4-m
24	17.599	17.535	17.795	20170738	1.07	5939133	1.60	3.40		.alpha.-Terpineol
25	18.271	18.245	18.325	826742	0.04	333486	0.09	2.48	V	3-Cyclohexene-1-acetal
26	20.072	20.025	20.140	1260856	0.07	414643	0.11	3.04		1H-2-Indenone,2,4,5,6,
27	20.558	20.495	20.635	3760994	0.20	1234881	0.33	3.05		2H-1-Benzopyran, 3,4,4
28	22.637	22.580	22.710	1911577	0.10	533281	0.14	3.58		Cubebene <alpha->
29	23.662	23.605	23.735	1483375	0.08	388675	0.11	3.82		Ylangene
30	23.976	23.895	24.075	5739175	0.30	1280676	0.35	4.48		Copaene
31	24.375	24.275	24.465	24860125	1.32	5526027	1.49	4.50		(-)-.beta.-Bourbonene
32	24.685	24.640	24.785	3870667	0.20	883927	0.24	4.38	V	Cyclohexane, 1-ethenyl-
33	26.343	26.135	26.515	351294738	18.59	55322242	14.95	6.35		Caryophyllene
34	26.844	26.745	26.955	8741632	0.46	1823921	0.49	4.79		(1R,2S,6S,7S,8S)-8-Iso
35	27.671	27.595	27.770	2210732	0.12	452959	0.12	4.88		Isogermacrene D
36	28.337	28.190	28.485	61877522	3.27	11061650	2.99	5.59		Humulene <alpha->
37	29.686	29.530	29.820	37257315	1.97	5364033	1.45	6.95		Muurolene <gamma->
38	30.087	29.820	30.325	500361460	26.48	60785663	16.42	8.23	V	Germacrene D
39	30.445	30.325	30.575	2521187	0.13	291375	0.08	8.65	V	Naphthalene, decahydro-
40	30.625	30.575	30.685	3113075	0.16	542249	0.15	5.74	V	cis-Muurola-4(15),5-di
41	30.762	30.685	30.865	10132040	0.54	1671738	0.45	6.06	V	Ylangene
42	30.925	30.865	31.015	2670029	0.14	452617	0.12	5.90	V	2-Isopropenyl-4a,8-dim
43	31.210	31.015	31.365	22715148	1.20	3470084	0.94	6.55	V	.alpha.-Muurolene
44	31.486	31.365	31.575	2725551	0.14	498880	0.13	5.46		Cadinene <delta->
45	32.137	31.975	32.305	23233459	1.23	3740648	1.01	6.21		Cadinene <gamma->

峰号	保留时间	I.Time	F.Time	峰面积	峰面积%	峰高	峰高%	积/高度	标记	名称
46	32.590	32.415	32.765	63198460	3.34	9923985	2.68	6.37		Cadinene <delta->
47	33.495	33.375	33.625	2265362	0.12	369872	0.10	6.12		Naphthalene <1,2,3,4,4a
48	33.804	33.635	33.925	3384569	0.18	566494	0.15	5.97		Cadinene <alpha->
49	35.195	35.065	35.375	4382042	0.23	625473	0.17	7.01		2-(4a,8-Dimethyl-1,2,3,
50	36.952	36.795	37.175	20864077	1.10	2690002	0.73	7.76		Caryophyllene oxide
51	37.595	37.485	37.705	2544514	0.13	403908	0.11	6.30		Ylangenol
52	38.778	38.615	38.955	7265213	0.38	864367	0.23	8.41		Dodecanoate <ethyl->
53	39.094	38.955	39.255	3315481	0.18	414490	0.11	8.00	V	Humulene epoxide II
54	39.837	39.715	39.935	3333574	0.18	472348	0.13	7.06		1H-Cycloprop[e]azulen-
55	40.037	39.935	40.175	3865149	0.20	585230	0.16	6.60	V	Junenol
56	41.974	41.815	42.055	7833907	0.41	1006898	0.27	7.78		.tau.-Cadinol
57	42.154	42.055	42.275	11048221	0.58	1450945	0.39	7.61	V	.alpha.-Cadinol
58	42.390	42.275	42.585	5125811	0.27	611969	0.17	8.38	V	1-Naphthalenol, 1,2,3,
59	43.079	42.905	43.355	19611397	1.04	2090327	0.56	9.38		Cadin-4-en-10-ol
60	45.422	45.245	45.605	10410240	0.55	1247251	0.34	8.35		(1R,7S,E)-7-Isopropyl-
61	45.894	45.765	46.075	2633336	0.14	333564	0.09	7.89		7R,8R-8-Hydroxy-4-isop
62	49.019	48.865	49.195	5049143	0.27	627260	0.17	8.05		Pentadecanal-
63	88.331	88.255	88.425	2045925	0.11	498770	0.13	4.10		(E)-3-Methyl-5-((1R,4a
				1889619397	100.00	370084481	100.00			

谱库

<< 目标组分 >>
行号#:1　保留时间:8.880(扫描数#:777)　质量峰:252
原始模式:平均 8.875-8.885(776-778)　基峰:93.10(896103)
背景模式:峰校准　组 1 - 事件 1　Scan

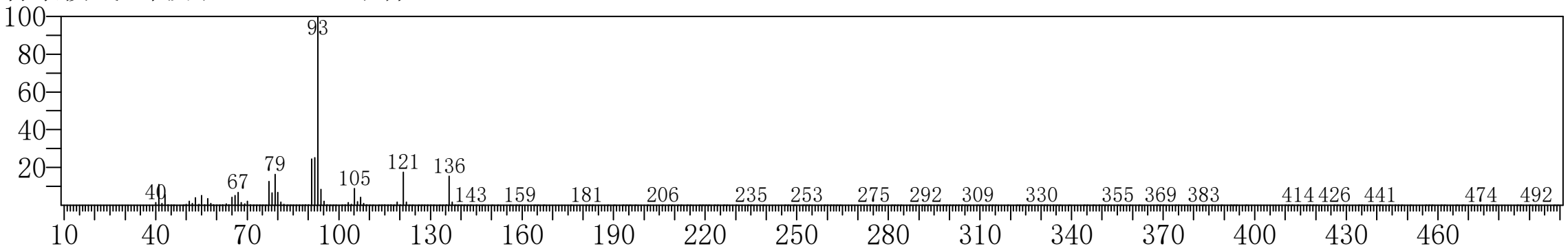

命中#:1　输入:7945　谱库:NIST17s.lib
SI:94　分子式:C10H16　CAS:508-32-7　摩尔质量:136　保留指数:729
组分名称:Tricyclo[2.2.1.0(2,6)]heptane, 1,7,7-trimethyl- $$ Tricyclene $$ 1,7,7-Trimethyltricyclo[2.2.1.0,2,

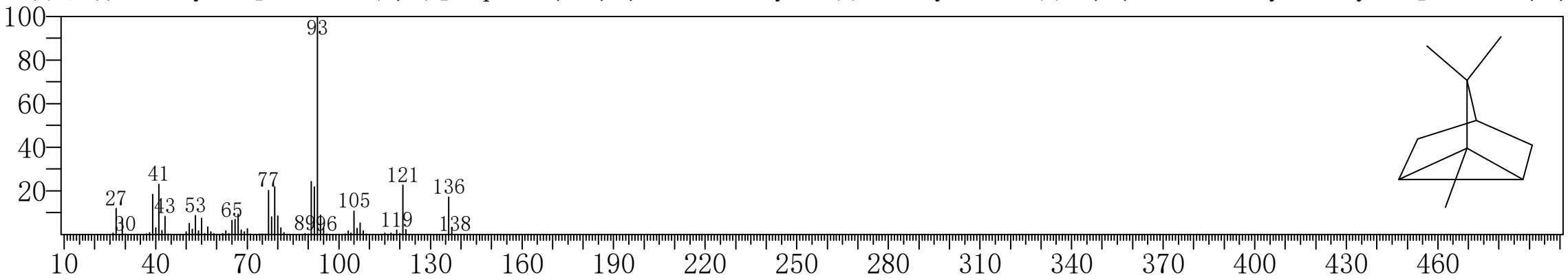

命中#:2　输入:986　谱库:FFNSC1.3.lib
SI:94　分子式:C10 H16　CAS:508-32-7　摩尔质量:136　保留指数:923
组分名称:Tricyclene

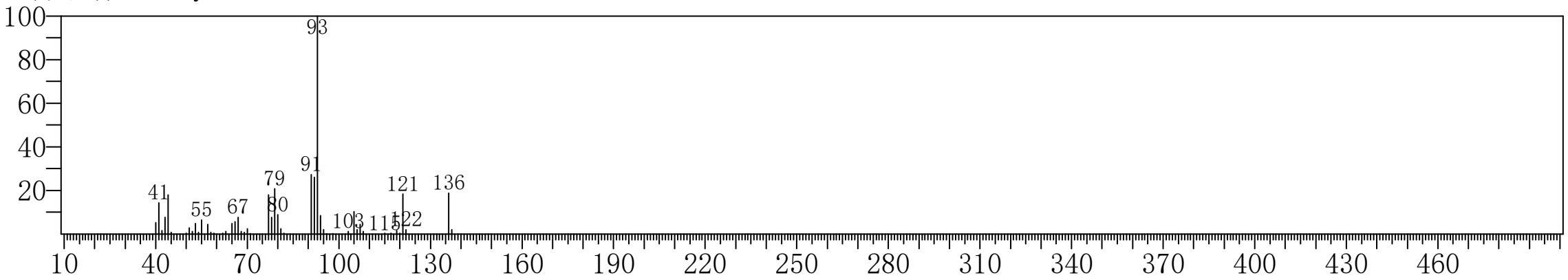

命中#:3　输入:10394　谱库:NIST17-1.lib
SI:94　分子式:C10H16　CAS:7785-70-8　摩尔质量:136　保留指数:948
组分名称:(1R)-2,6,6-Trimethylbicyclo[3.1.1]hept-2-ene $$ 1R-.alpha.-Pinene $$ Bicyclo[3.1.1]hept-2-ene, 2,6,

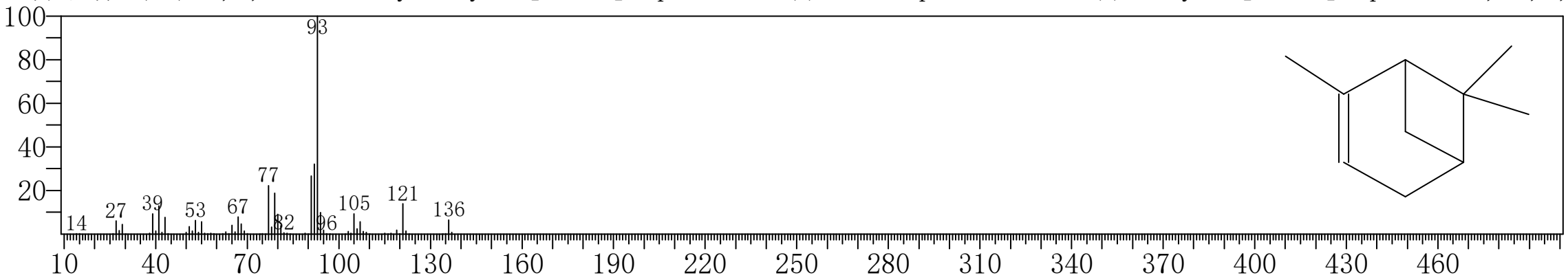

命中#:4　输入:10393　谱库:NIST17-1.lib
SI:94　分子式:C10H16　CAS:7785-26-4　摩尔质量:136　保留指数:948
组分名称:(1S)-2,6,6-Trimethylbicyclo[3.1.1]hept-2-ene $$ 1S-.alpha.-Pinene $$ (-)-.alpha.-Pinene $$ L-.alpha

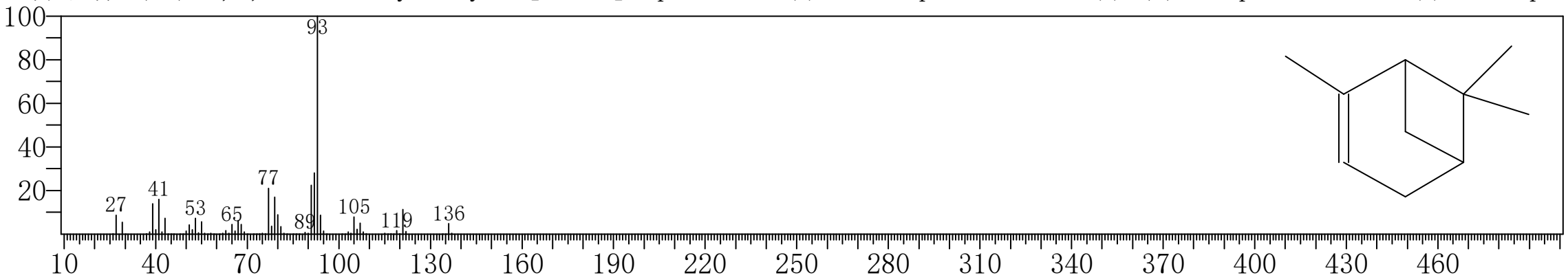

<< 目标组分 >>
行号#:1　保留时间:8.880(扫描数#:777)　质量峰:252
原始模式:平均 8.875-8.885(776-778)　基峰:93.10(896103)
背景模式:峰校准　组 1 - 事件 1　Scan

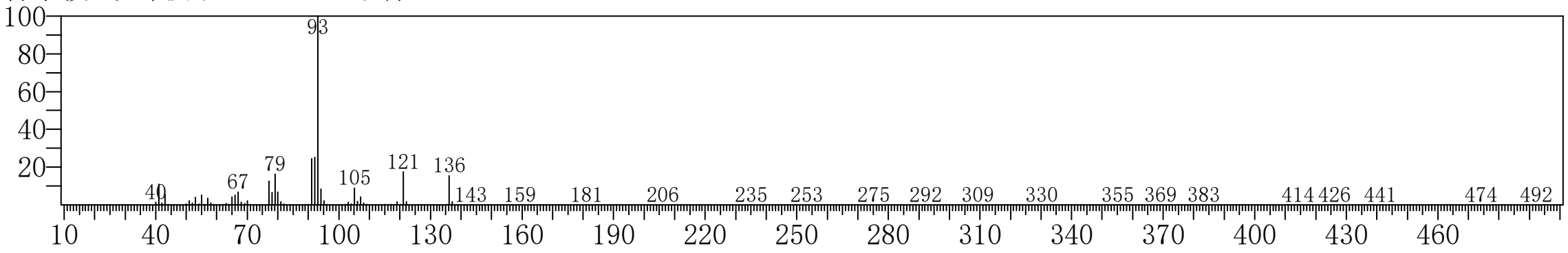

命中#:5　输入:10388　谱库:NIST17-1.lib
SI:94　分子式:C10H16　CAS:508-32-7　摩尔质量:136　保留指数:729
组分名称:Tricyclo[2.2.1.0(2,6)]heptane, 1,7,7-trimethyl- $$ Tricyclene $$ 1,7,7-Trimethyltricyclo[2.2.1.0,2,

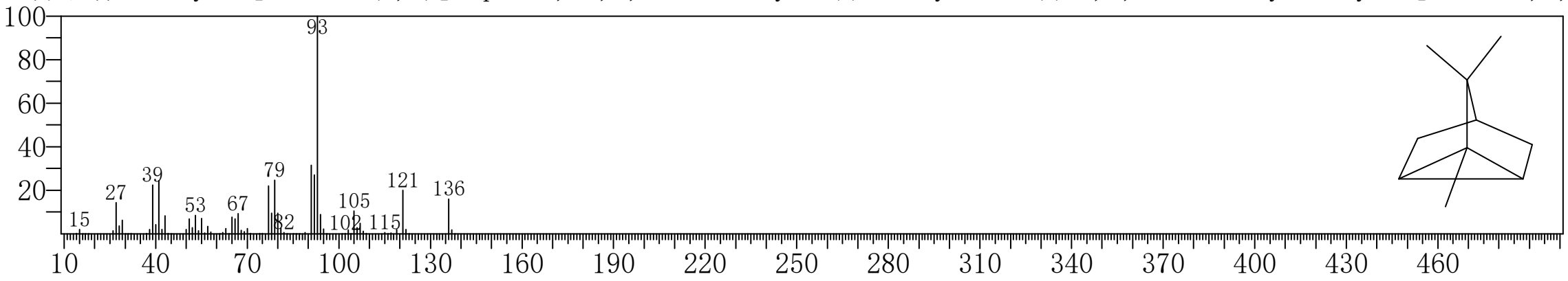

<< 目标组分 >>
行号#:2　保留时间:9.300(扫描数#:861)　质量峰:274
原始模式:平均 9.295-9.305(860-862)　基峰:91.15(8390566)
背景模式:峰校准　组 1 - 事件 1　Scan

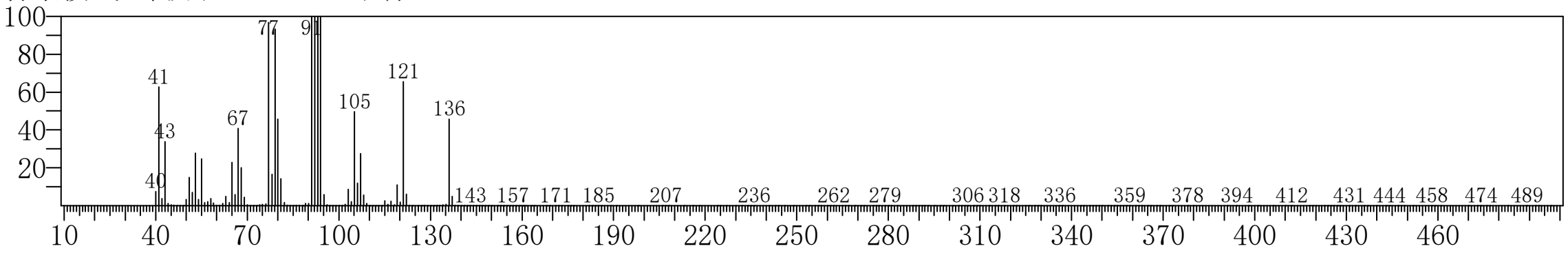

命中#:1　输入:10372　谱库:NIST17-1.lib
SI:89　分子式:C10H16　CAS:55956-33-7　摩尔质量:136　保留指数:912
组分名称:3-Octen-5-yne, 2,7-dimethyl-, (E)- $$ (3E)-2,7-Dimethyl-3-octen-5-yne # $$

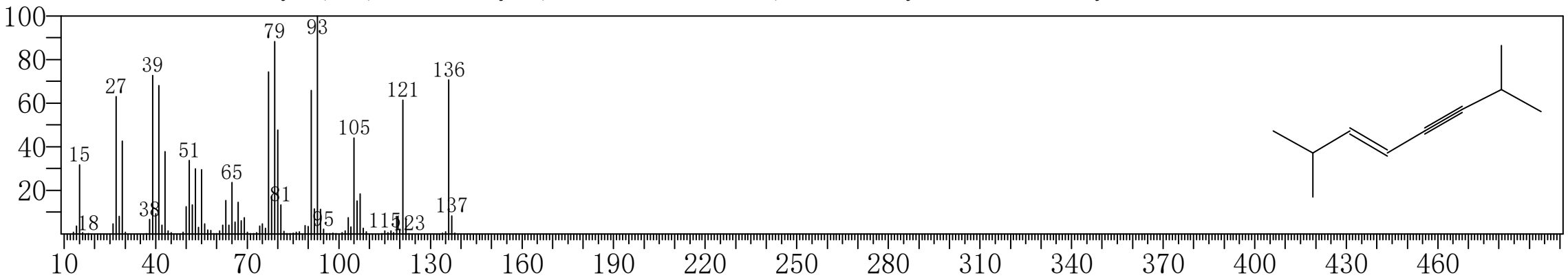

命中#:2　输入:10370　谱库:NIST17-1.lib
SI:89　分子式:C10H16　CAS:28935-76-4　摩尔质量:136　保留指数:912
组分名称:3-Octen-5-yne, 2,7-dimethyl-, (Z)- $$ (3Z)-2,7-Dimethyl-3-octen-5-yne # $$

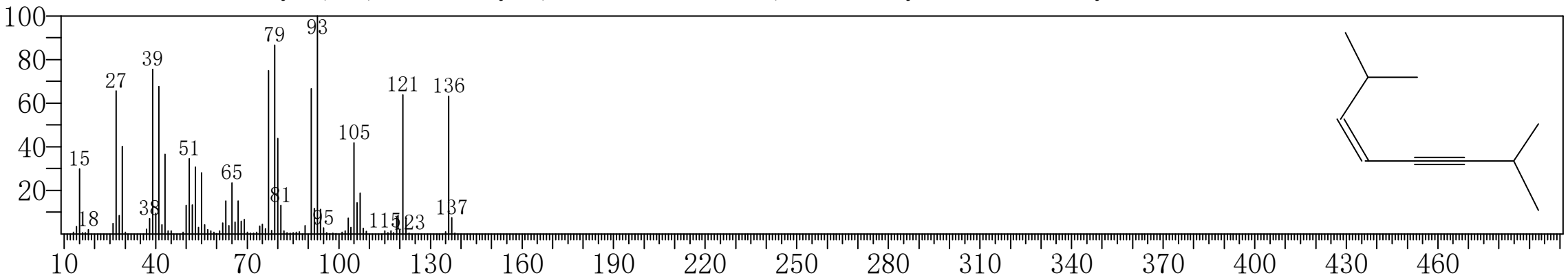

命中#:3　输入:10378　谱库:NIST17-1.lib
SI:86　分子式:C10H16　CAS:24648-33-7　摩尔质量:136　保留指数:935
组分名称:2,4,6-Trimethyl-1,3,6-heptatriene $$ (3E)-2,4,6-Trimethyl-1,3,6-heptatriene # $$

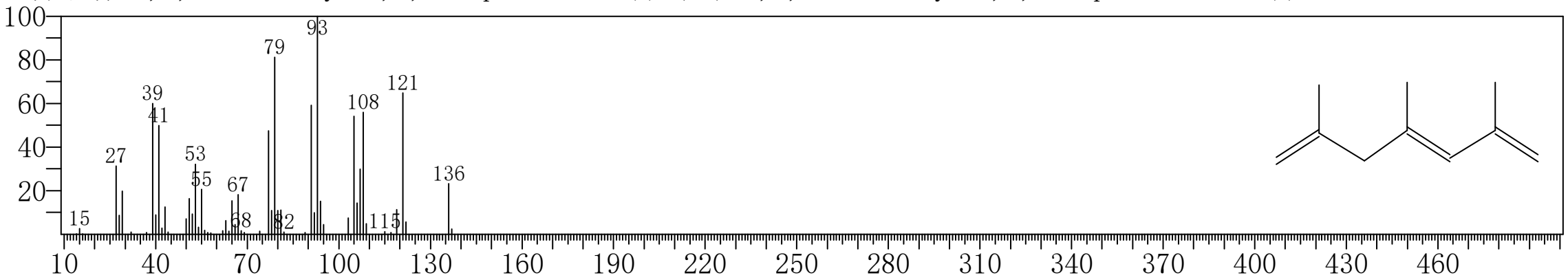

命中#:4　输入:10399　谱库:NIST17-1.lib
SI:86　分子式:C10H16　CAS:42123-66-0　摩尔质量:136　保留指数:894
组分名称:1,3,6-Heptatriene, 2,5,6-trimethyl- $$ 2,5,6-Trimethyl-1,3,6-heptatriene $$ 2,5,6-Trimethyl-hepta-

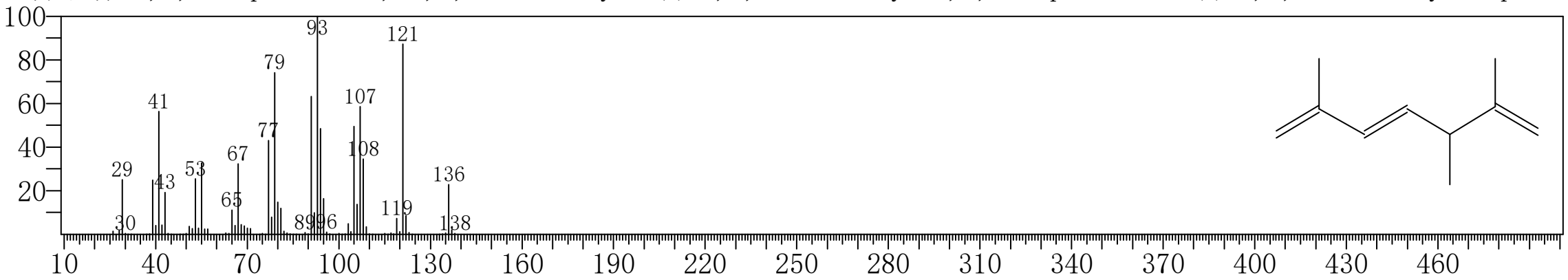

命中#:5　输入:7928　谱库:NIST17s.lib
SI:86　分子式:C10H16　CAS:2153-66-4　摩尔质量:136　保留指数:894
组分名称:Santolina triene $$ 2,5-Dimethyl-3-vinyl-1,4-hexadiene # $$

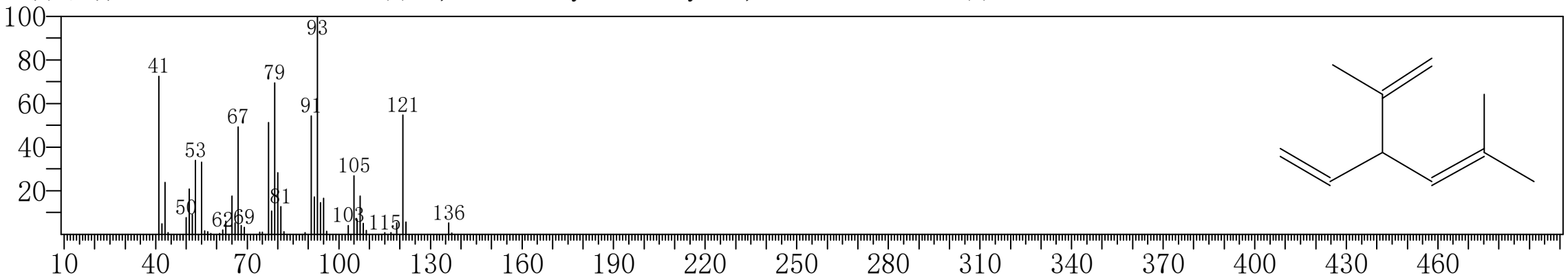

<< 目标组分 >>
行号#:3　保留时间:9.780(扫描数#:957)　质量峰:243
原始模式:平均 9.775-9.785(956-958)　基峰:93.10(2406529)
背景模式:峰校准　组 1 - 事件 1　Scan

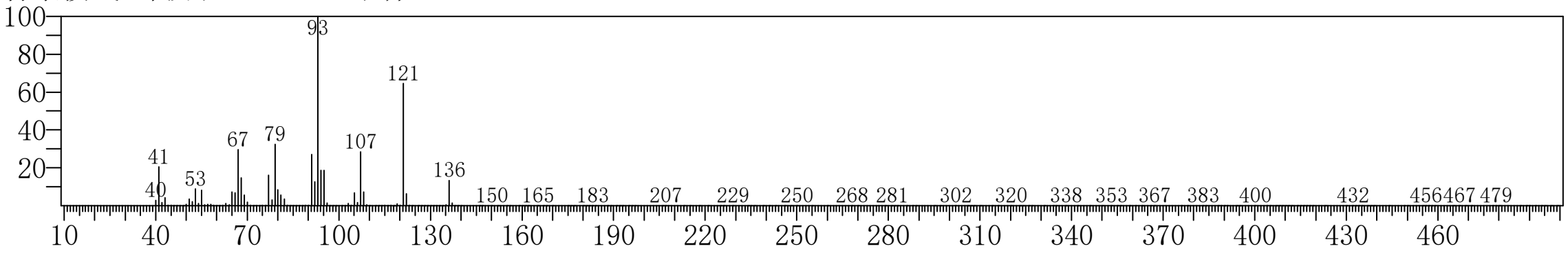

命中#:1　输入:952　谱库:FFNSC1.3.lib
SI:96　分子式:C10 H16　CAS:79-92-5　摩尔质量:136　保留指数:953
组分名称:Camphene

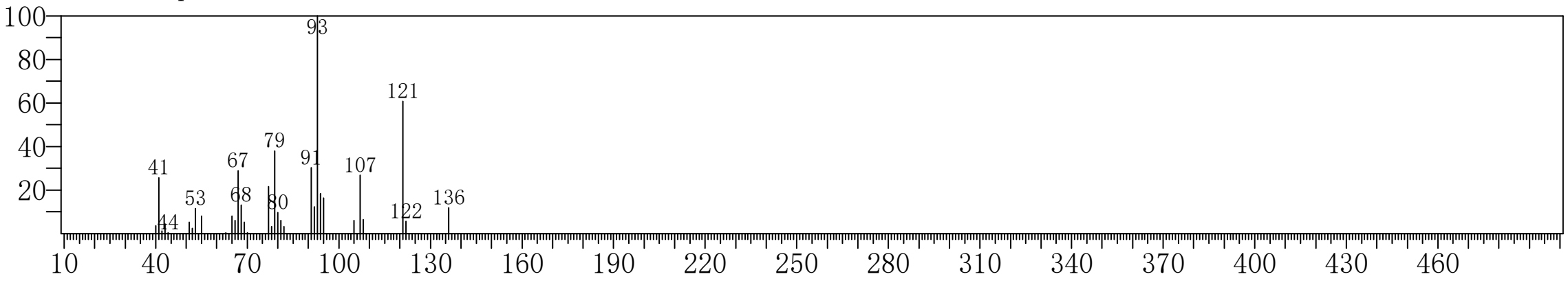

命中#:2　输入:7967　谱库:NIST17s.lib
SI:96　分子式:C10H16　CAS:79-92-5　摩尔质量:136　保留指数:943
组分名称:Camphene $$ Bicyclo[2.2.1]heptane, 2,2-dimethyl-3-methylene- $$ 2,2-Dimethyl-3-methylenebicyclo[2.2

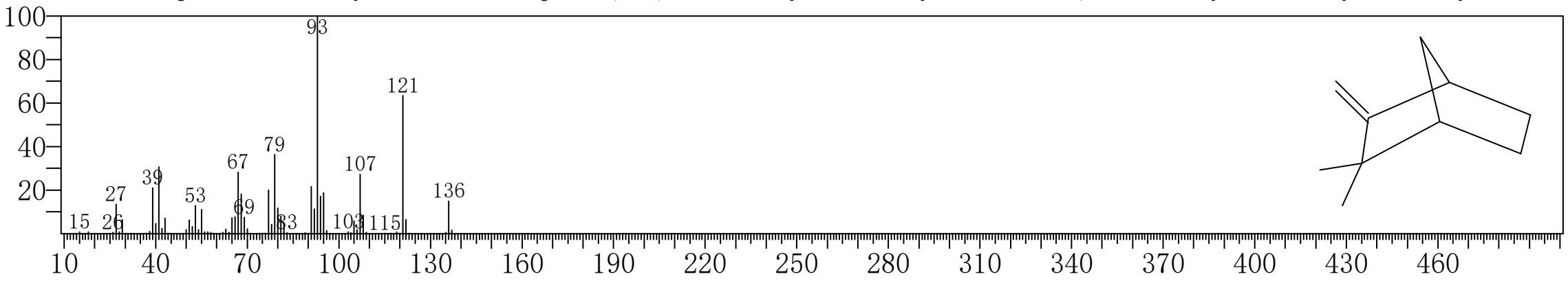

命中#:3　输入:10397　谱库:NIST17-1.lib
SI:94　分子式:C10H16　CAS:79-92-5　摩尔质量:136　保留指数:943
组分名称:Camphene $$ Bicyclo[2.2.1]heptane, 2,2-dimethyl-3-methylene- $$ 2,2-Dimethyl-3-methylenebicyclo[2.2

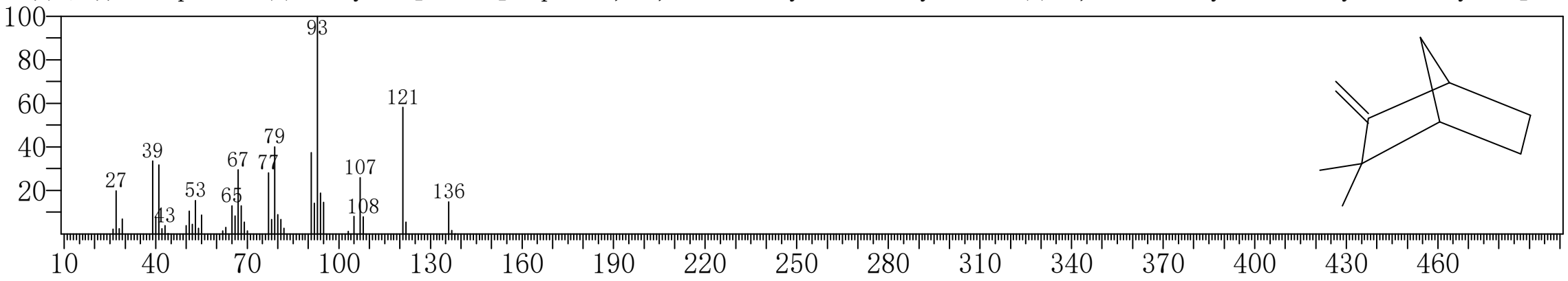

命中#:4　输入:10395　谱库:NIST17-1.lib
SI:94　分子式:C10H16　CAS:5794-04-7　摩尔质量:136　保留指数:943
组分名称:Bicyclo[2.2.1]heptane, 2,2-dimethyl-3-methylene-, (1S)- $$ 2,2-Dimethyl-3-methylenebicyclo[2.2.1]he

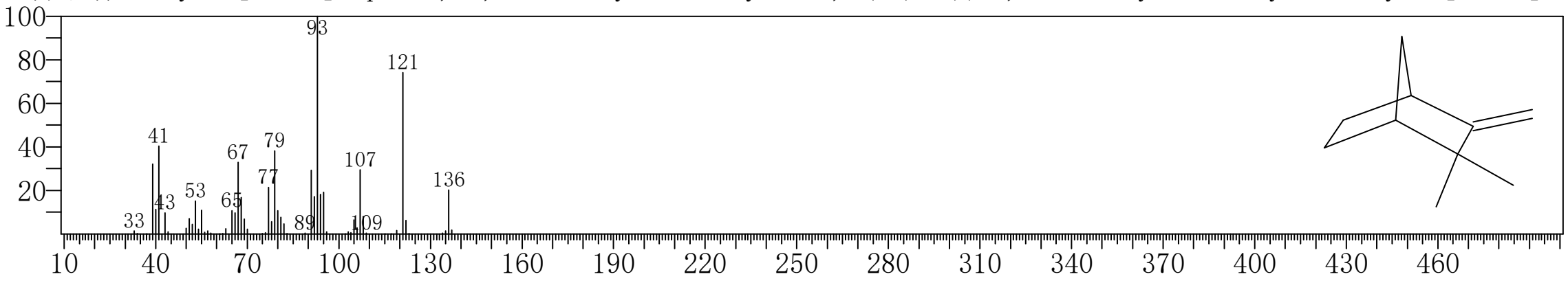

命中#:5　输入:7966　谱库:NIST17s.lib
SI:92　分子式:C10H16　CAS:79-92-5　摩尔质量:136　保留指数:943
组分名称:Camphene $$ Bicyclo[2.2.1]heptane, 2,2-dimethyl-3-methylene- $$ 2,2-Dimethyl-3-methylenebicyclo[2.2

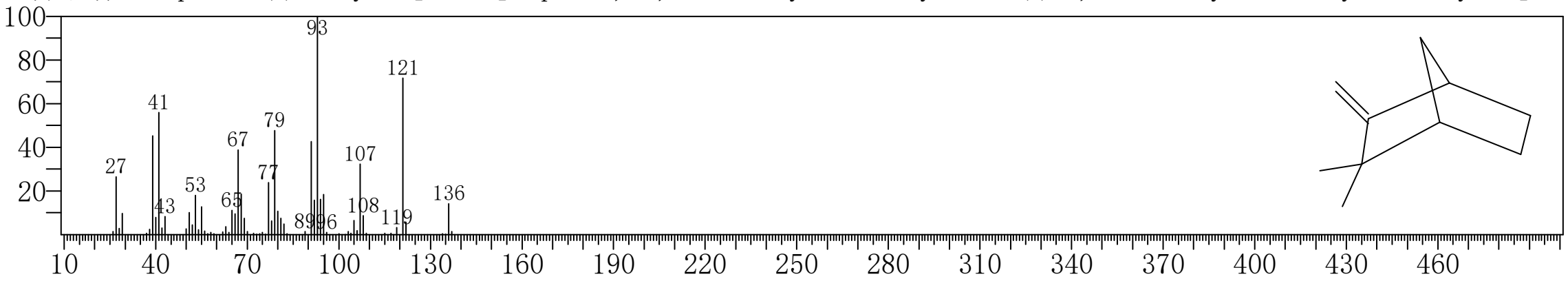

<< 目标组分 >>
行号#:4　保留时间:9.915(扫描数#:984)　质量峰:243
原始模式:平均 9.910-9.920(983-985)　基峰:91.10(69421)
背景模式:峰校准　组 1 - 事件 1　Scan

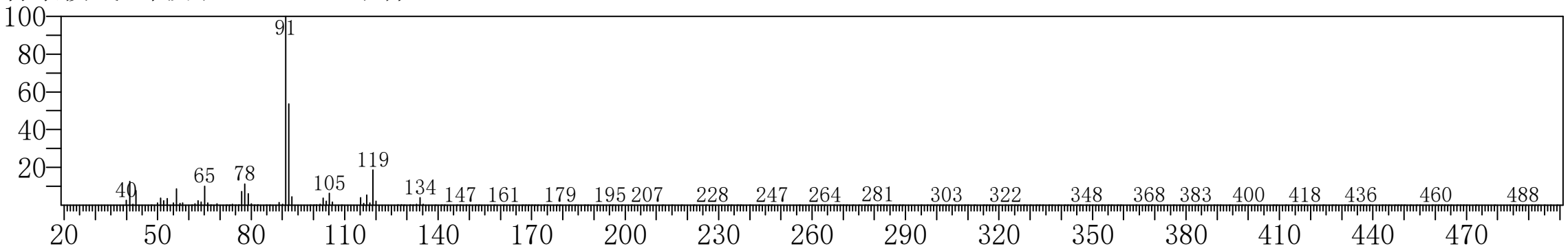

命中#:1　输入:556　谱库:FFNSC1.3.lib
SI:96　分子式:C10 H14　CAS:36262-09-6　摩尔质量:134　保留指数:953
组分名称:Thuja-2,4(10)-diene

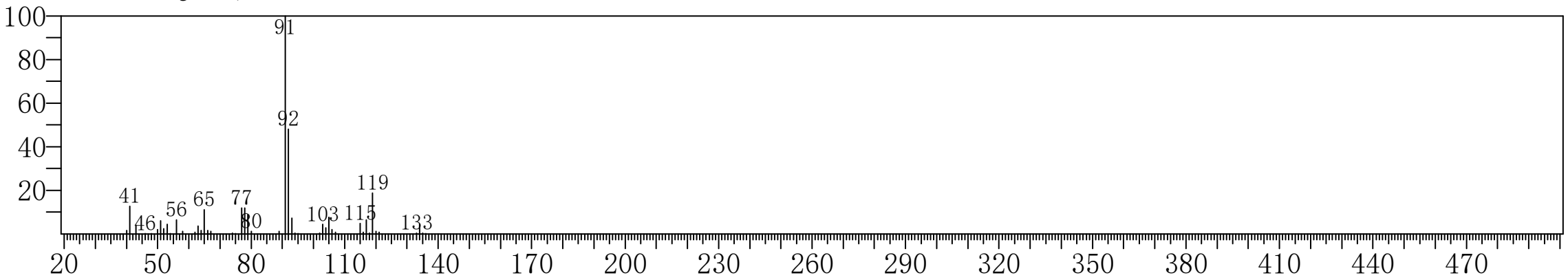

命中#:2　输入:7374　谱库:NIST17s.lib
SI:95　分子式:C10H14　CAS:36262-09-6　摩尔质量:134　保留指数:879
组分名称:Bicyclo[3.1.0]hex-2-ene, 4-methylene-1-(1-methylethyl)- $$ 1-Isopropyl-4-methylenebicyclo[3.1.0]he:

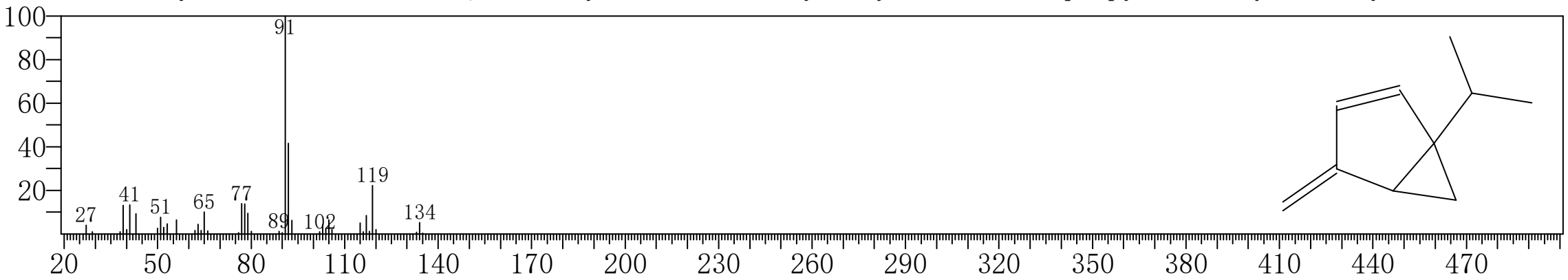

命中#:3　输入:9619　谱库:NIST17-1.lib
SI:93　分子式:C10H14　CAS:36262-09-6　摩尔质量:134　保留指数:879
组分名称:Bicyclo[3.1.0]hex-2-ene, 4-methylene-1-(1-methylethyl)- $$ 1-Isopropyl-4-methylenebicyclo[3.1.0]he:

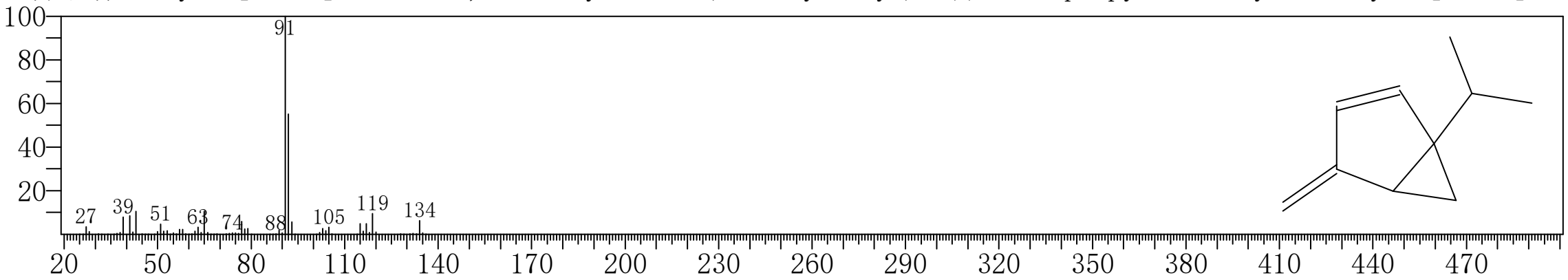

命中#:4　输入:7380　谱库:NIST17s.lib
SI:90　分子式:C10H14　CAS:104-51-8　摩尔质量:134　保留指数:1092
组分名称:Benzene, n-butyl- $$ 1-Phenylbutane $$ n-Butylbenzene $$ Butylbenzene $$ UN 2709 $$

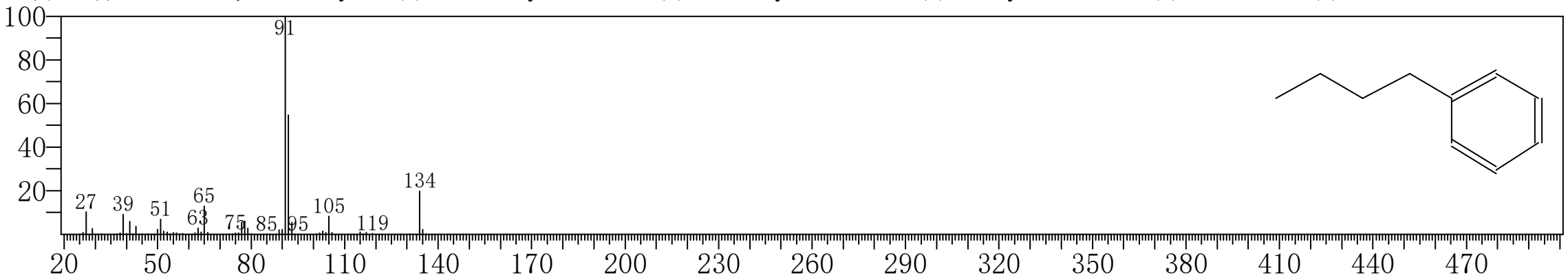

命中#:5　输入:7379　谱库:NIST17s.lib
SI:90　分子式:C10H14　CAS:104-51-8　摩尔质量:134　保留指数:1092
组分名称:Benzene, n-butyl- $$ 1-Phenylbutane $$ n-Butylbenzene $$ Butylbenzene $$ UN 2709 $$

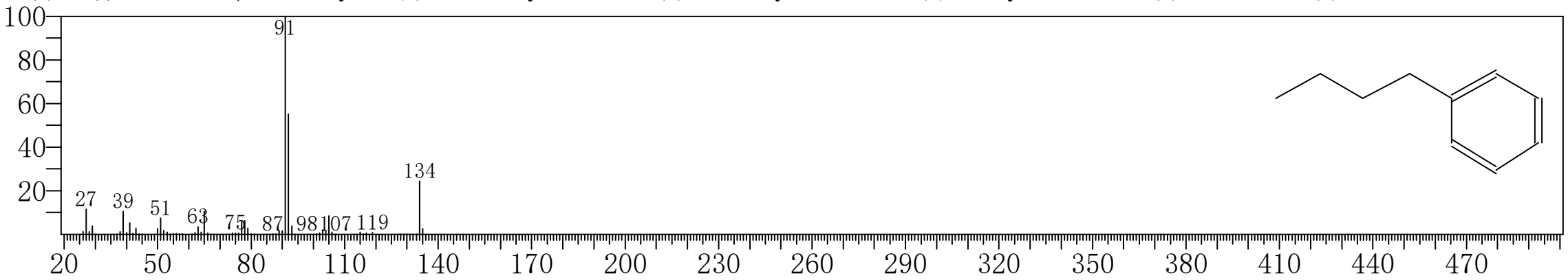

<< 目标组分 >>
行号#:5　保留时间:10.560(扫描数#:1113)　质量峰:195
原始模式:平均 10.555-10.565(1112-1114)　基峰:93.10(97520)
背景模式:峰校准　组 1 - 事件 1　Scan

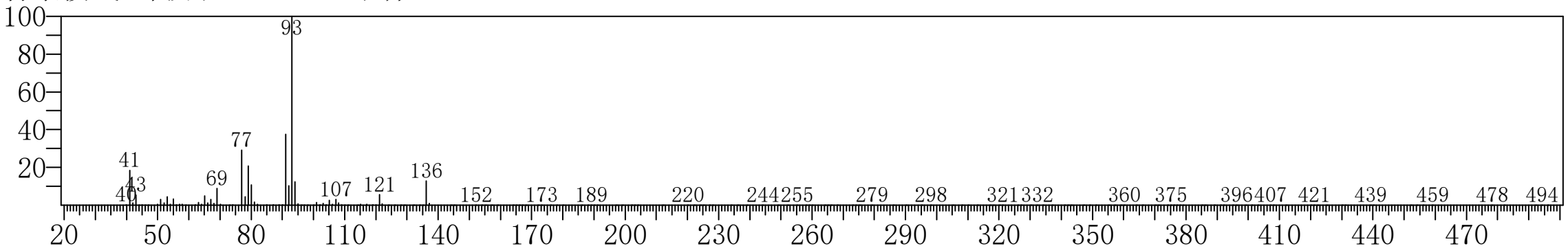

命中#:1　输入:958　谱库:FFNSC1.3.lib
SI:96　分子式:C10 H16　CAS:3387-41-5　摩尔质量:136　保留指数:972
组分名称:Sabinene

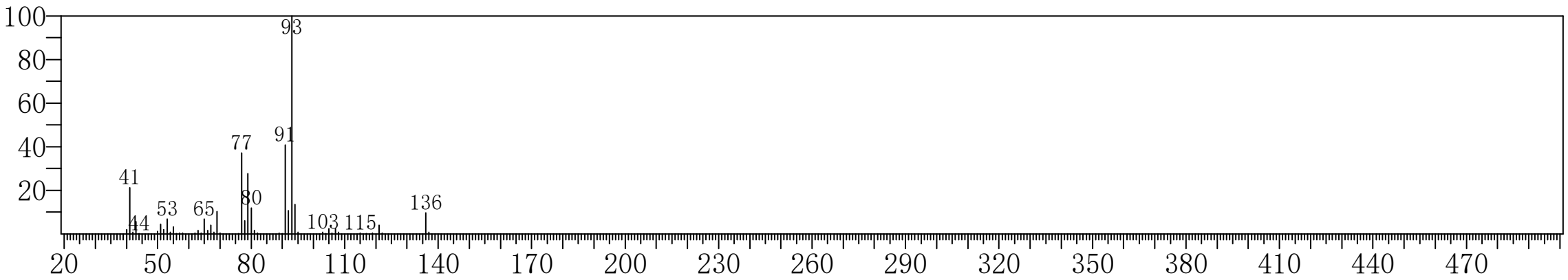

命中#:2　输入:10383　谱库:NIST17-1.lib
SI:95　分子式:C10H16　CAS:3387-41-5　摩尔质量:136　保留指数:897
组分名称:Bicyclo[3.1.0]hexane, 4-methylene-1-(1-methylethyl)- $$ 1-Isopropyl-4-methylenebicyclo[3.1.0]hexane

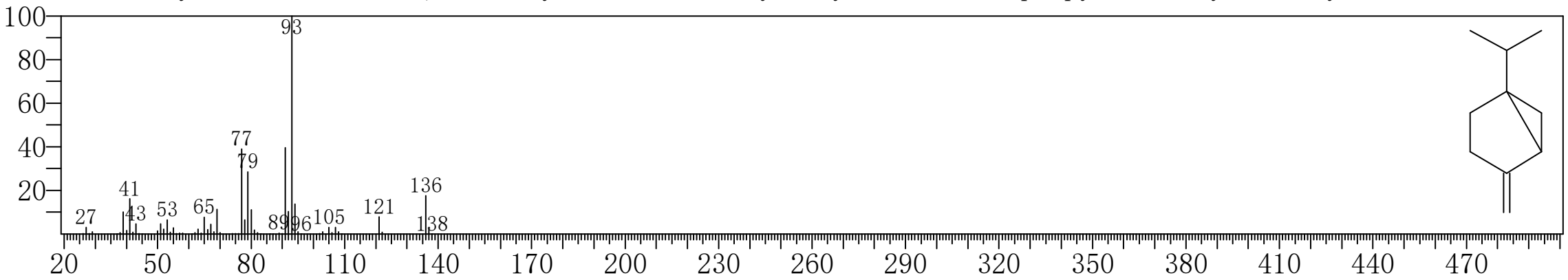

命中#:3　输入:7935　谱库:NIST17s.lib
SI:95　分子式:C10H16　CAS:555-10-2　摩尔质量:136　保留指数:964
组分名称:.beta.-Phellandrene $$ Cyclohexene, 3-methylene-6-(1-methylethyl)- $$ p-Mentha-1(7),2-diene $$ Phe

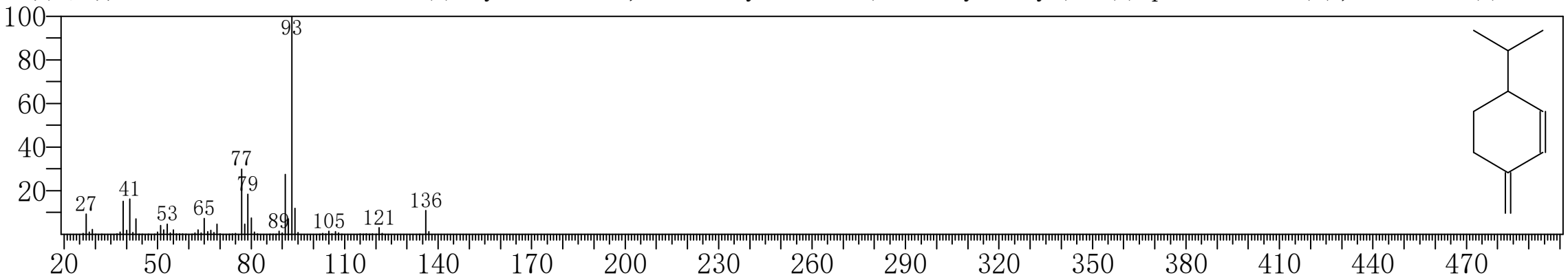

命中#:4　输入:10369　谱库:NIST17-1.lib
SI:94　分子式:C10H16　CAS:555-10-2　摩尔质量:136　保留指数:964
组分名称:.beta.-Phellandrene $$ Cyclohexene, 3-methylene-6-(1-methylethyl)- $$ p-Mentha-1(7),2-diene $$ Phe

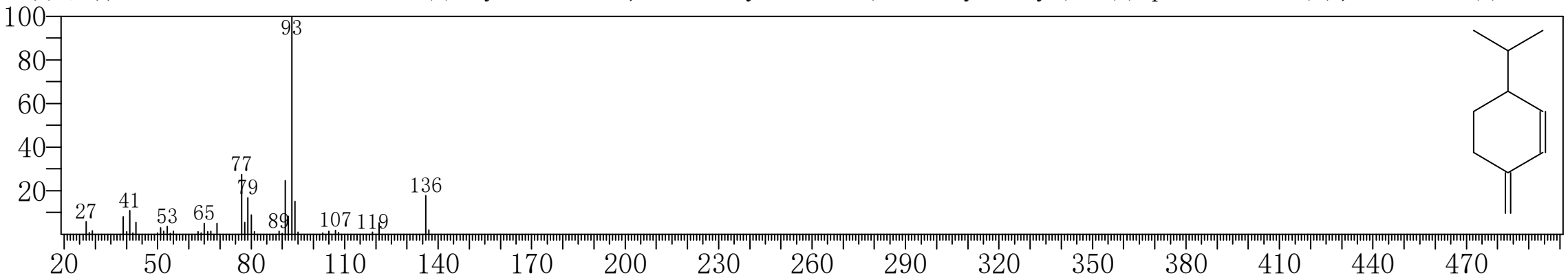

命中#:5　输入:7936　谱库:NIST17s.lib
SI:94　分子式:C10H16　CAS:555-10-2　摩尔质量:136　保留指数:964
组分名称:.beta.-Phellandrene $$ Cyclohexene, 3-methylene-6-(1-methylethyl)- $$ p-Mentha-1(7),2-diene $$ Phe

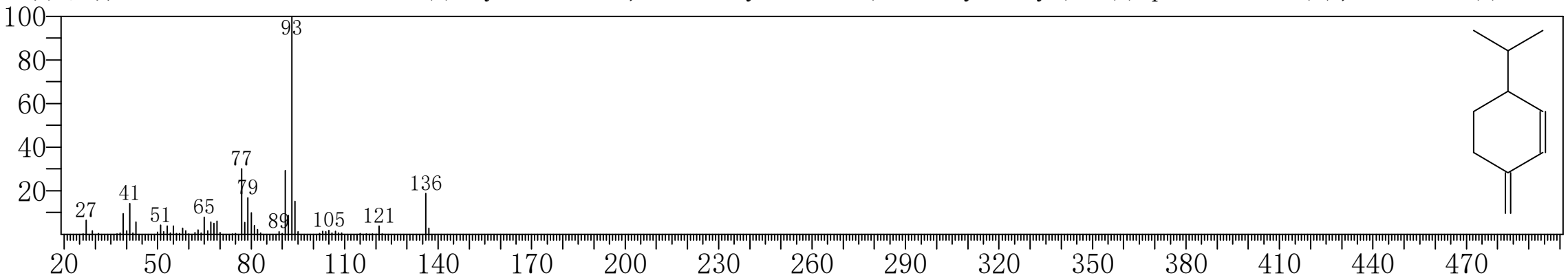

<< 目标组分 >>
行号#:6　保留时间:10.695(扫描数#:1140)　质量峰:288
原始模式:平均 10.690-10.700(1139-1141)　基峰:93.10(7580802)
背景模式:峰校准　组 1 - 事件 1　Scan

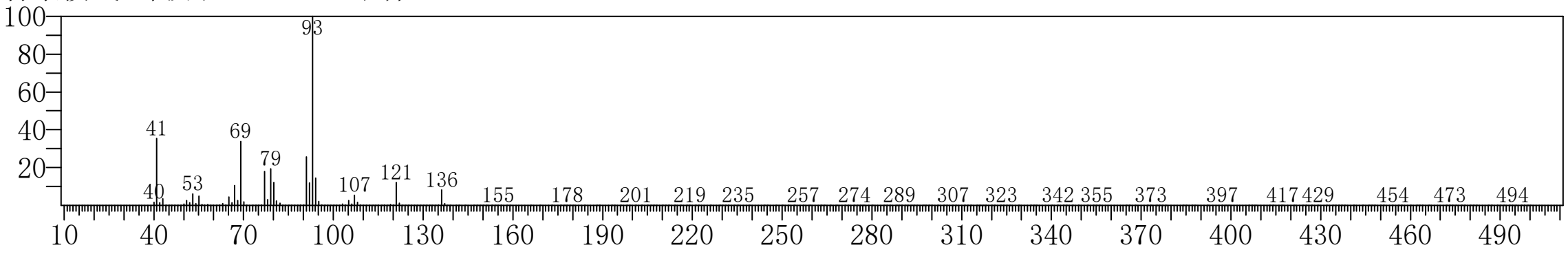

命中#:1　输入:837　谱库:FFNSC1.3.lib
SI:97　分子式:C10 H16　CAS:127-91-3　摩尔质量:136　保留指数:978
组分名称:Pinene <beta->

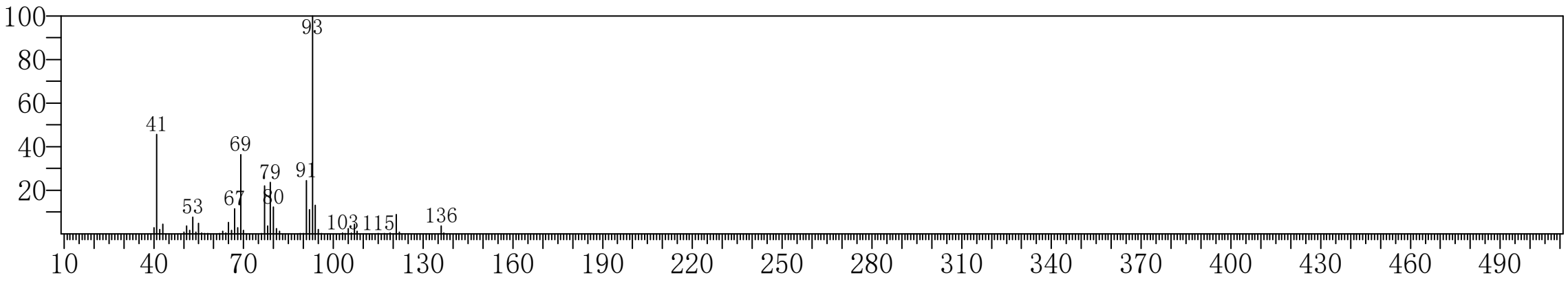

命中#:2　输入:10355　谱库:NIST17-1.lib
SI:97　分子式:C10H16　CAS:18172-67-3　摩尔质量:136　保留指数:943
组分名称:Bicyclo[3.1.1]heptane, 6,6-dimethyl-2-methylene-, (1S)- $$ 2(10)-Pinene, (1S,5S)-(-)- $$ (-)-.beta.

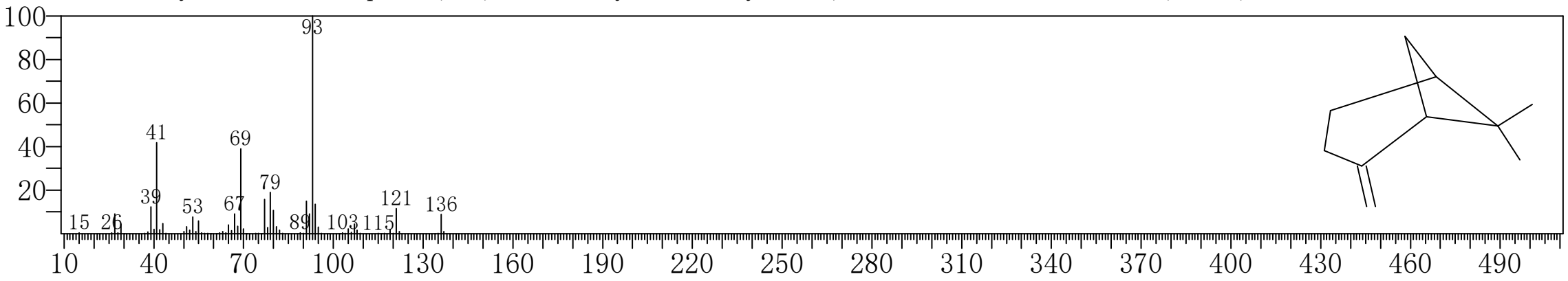

命中#:3　输入:7921　谱库:NIST17s.lib
SI:95　分子式:C10H16　CAS:127-91-3　摩尔质量:136　保留指数:943
组分名称:.beta.-Pinene $$ Bicyclo[3.1.1]heptane, 6,6-dimethyl-2-methylene- $$ 2(10)-Pinene $$ Nopinen $$ No

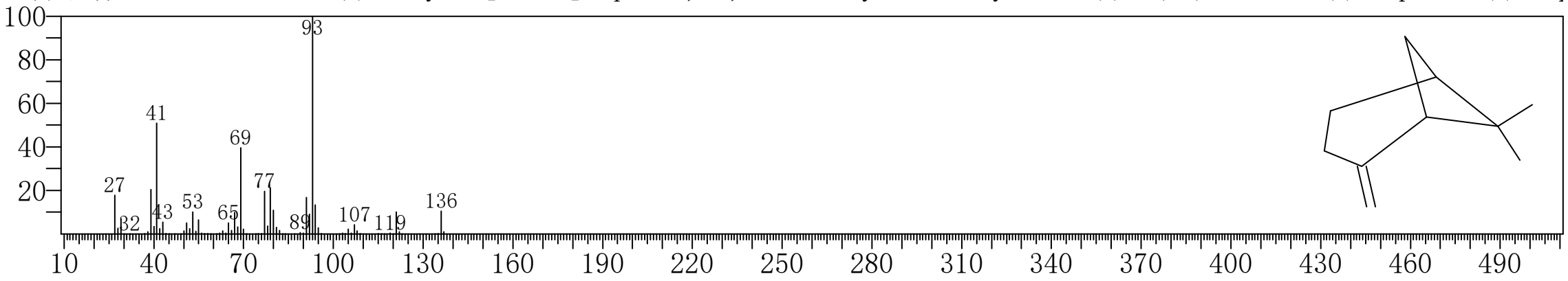

命中#:4　输入:7918　谱库:NIST17s.lib
SI:95　分子式:C10H16　CAS:18172-67-3　摩尔质量:136　保留指数:943
组分名称:Bicyclo[3.1.1]heptane, 6,6-dimethyl-2-methylene-, (1S)- $$ 2(10)-Pinene, (1S,5S)-(-)- $$ (-)-.beta.

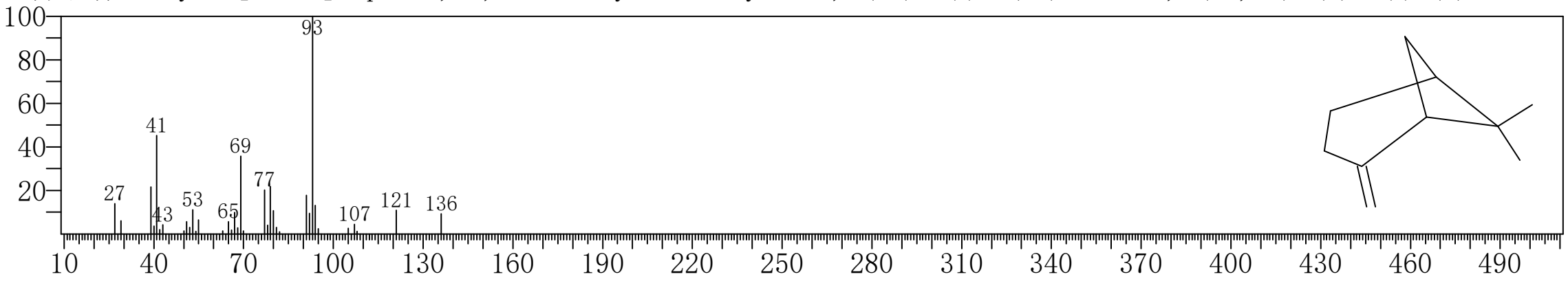

命中#:5　输入:7929　谱库:NIST17s.lib
SI:95　分子式:C10H16　CAS:18172-67-3　摩尔质量:136　保留指数:943
组分名称:Bicyclo[3.1.1]heptane, 6,6-dimethyl-2-methylene-, (1S)- $$ 2(10)-Pinene, (1S,5S)-(-)- $$ (-)-.beta.

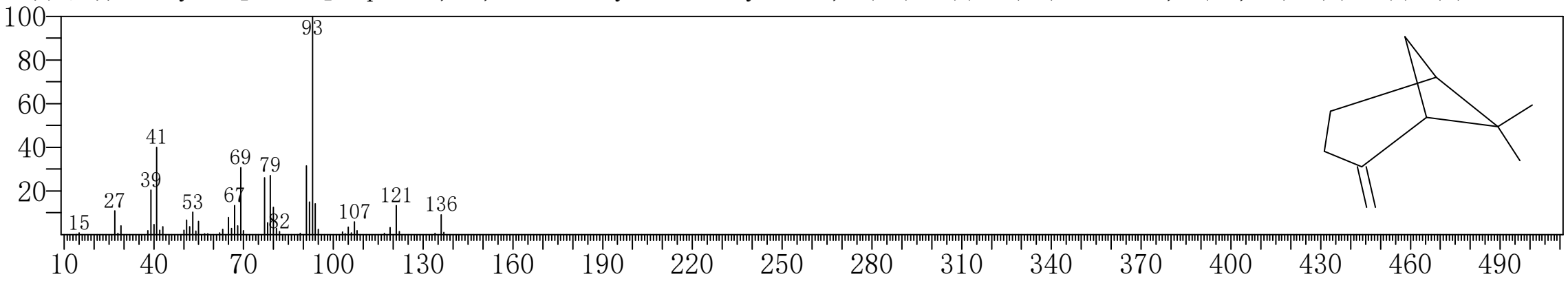

<< 目标组分 >>
行号#:7   保留时间:10.890(扫描数#:1179) 质量峰:276
原始模式:平均 10.885-10.895(1178-1180)   基峰:71.05(1520743)
背景模式:峰校准 组 1 - 事件 1 Scan

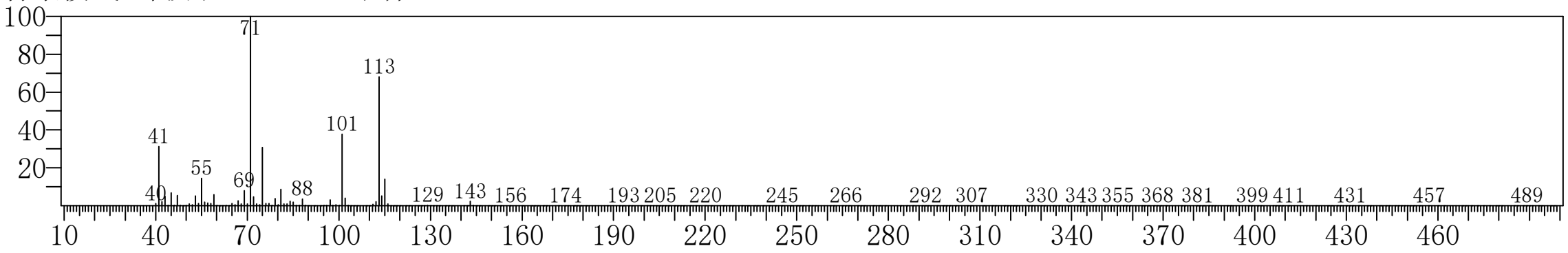

命中#:1 输入:13837 谱库:NIST17-1.lib
SI:96 分子式:C8H16O2 CAS:0-00-0 摩尔质量:144 保留指数:912
组分名称:trans-2-Hexenal dimethyl acetal

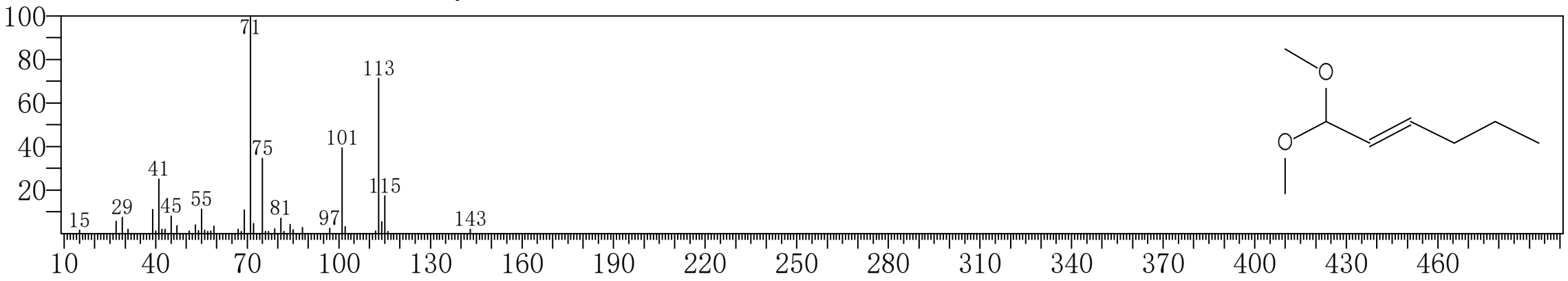

命中#:2 输入:13688 谱库:NIST17-1.lib
SI:80 分子式:C7H12O3 CAS:58185-66-3 摩尔质量:144 保留指数:968
组分名称:3-Pentenoic acid, 5-methoxy-, methyl ester $$ Methyl (3E)-5-methoxy-3-pentenoate # $$

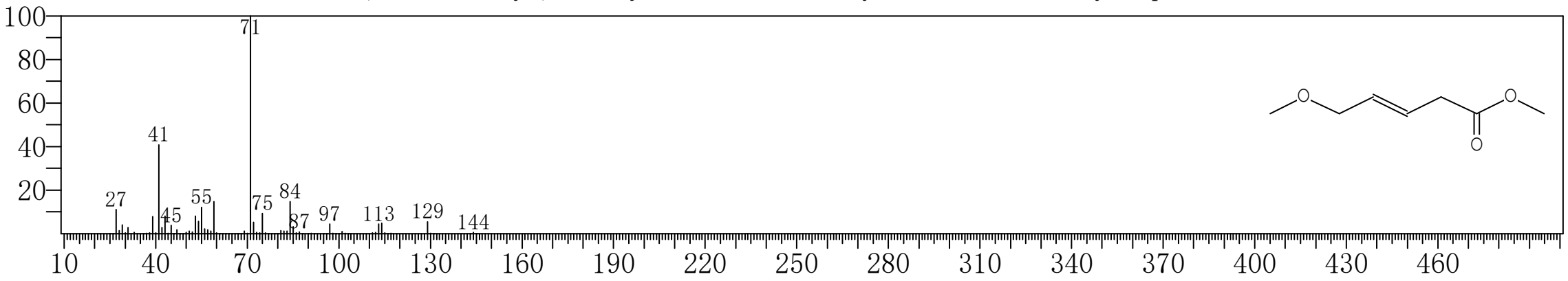

命中#:3 输入:31719 谱库:NIST17-1.lib
SI:79 分子式:C8H16O4 CAS:23068-91-9 摩尔质量:176 保留指数:1071
组分名称:Pentanoic acid, 5,5-dimethoxy-, methyl ester $$ Methyl 5,5-dimethoxypentanoate # $$

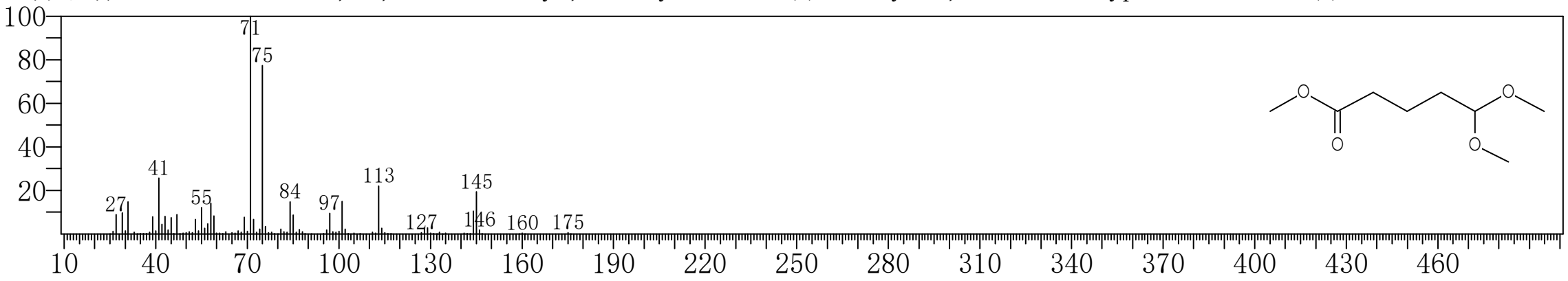

命中#:4 输入:13689 谱库:NIST17-1.lib
SI:75 分子式:C7H12O3 CAS:143538-29-8 摩尔质量:144 保留指数:968
组分名称:5-Methoxy-pent-4-enoic acid, methyl ester $$ Methyl (4E)-5-methoxy-4-pentenoate # $$

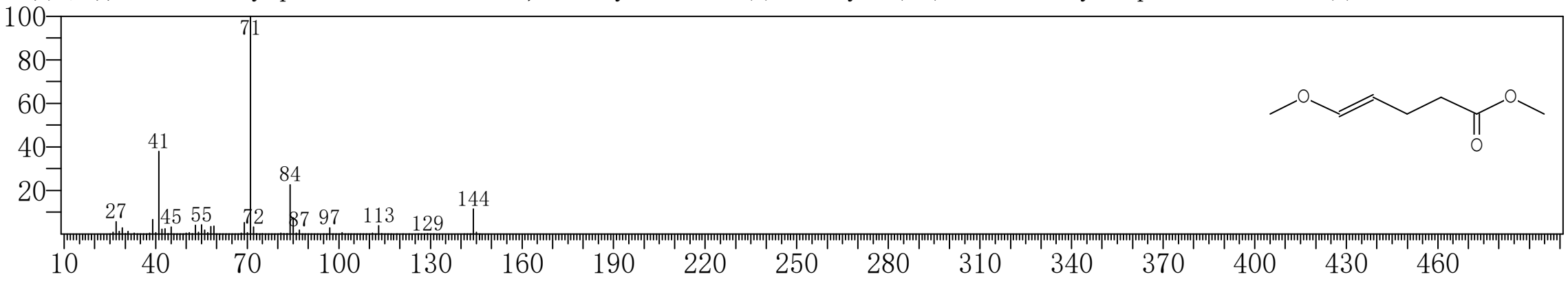

命中#:5 输入:2448 谱库:NIST17s.lib
SI:73 分子式:C5H10O2 CAS:6044-68-4 摩尔质量:102 保留指数:596
组分名称:Acrolein,dimethyl acetal $$ 1-Propene, 3,3-dimethoxy- $$ 3,3-Dimethoxypropene-1 $$ 3,3-Dimethoxy-1-

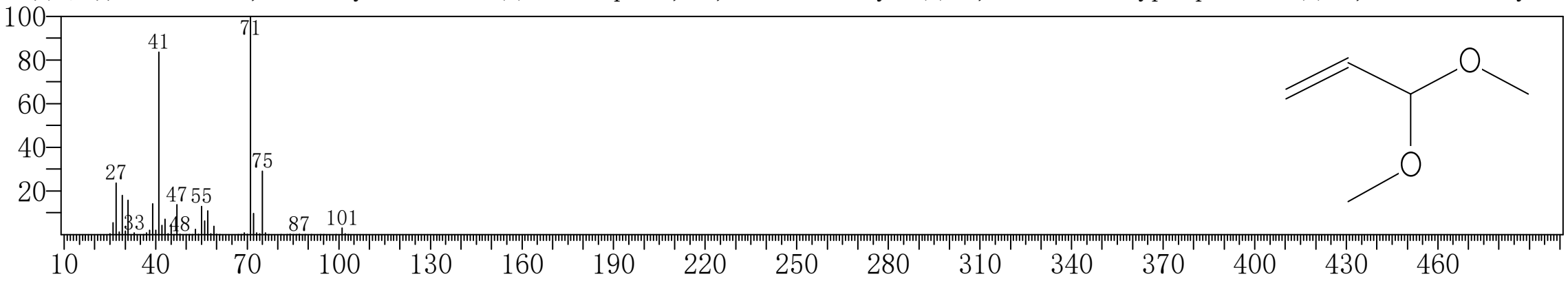

<< 目标组分 >>
行号#:8　保留时间:11.135(扫描数#:1228)　质量峰:301
原始模式:平均 11.130-11.140(1227-1229)　基峰:93.10(3700220)
背景模式:峰校准　组 1 - 事件 1　Scan

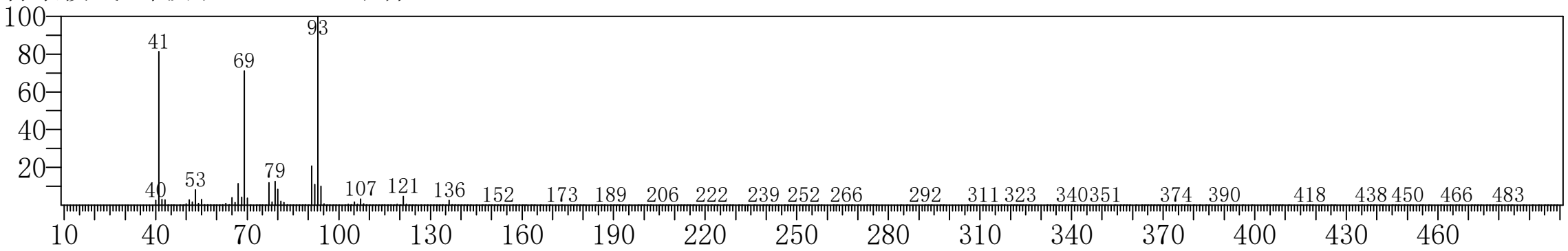

命中#:1　输入:628　谱库:FFNSC1.3.lib
SI:96　分子式:C10 H16　CAS:123-35-3　摩尔质量:136　保留指数:991
组分名称:Myrcene

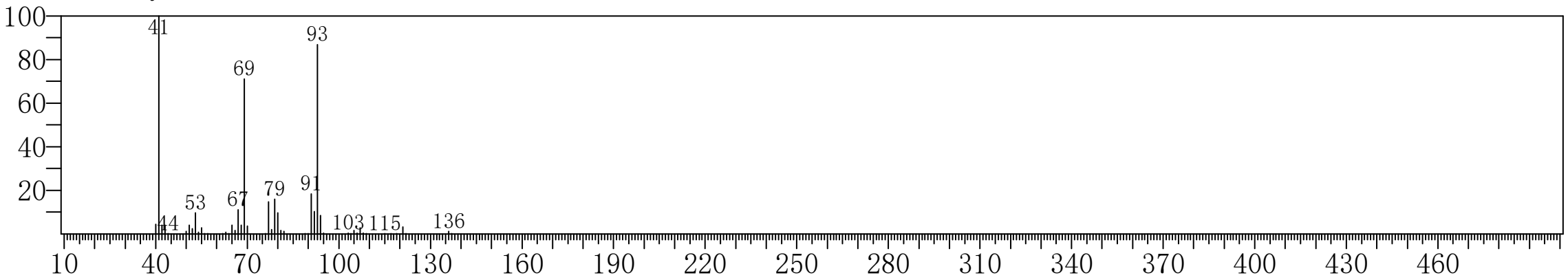

命中#:2　输入:7933　谱库:NIST17s.lib
SI:96　分子式:C10H16　CAS:123-35-3　摩尔质量:136　保留指数:958
组分名称:.beta.-Myrcene $$ 1,6-Octadiene, 7-methyl-3-methylene- $$ Myrcene $$ 7-Methyl-3-methylene-1,6-octa

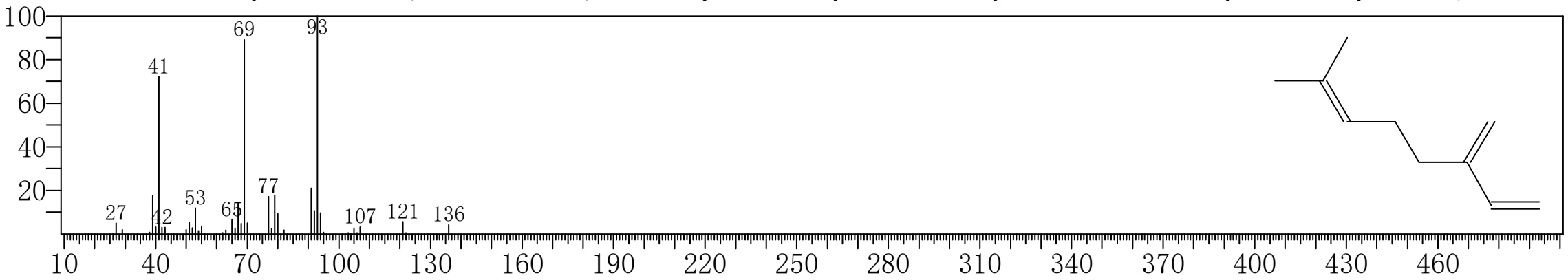

命中#:3　输入:7923　谱库:NIST17s.lib
SI:95　分子式:C10H16　CAS:123-35-3　摩尔质量:136　保留指数:958
组分名称:.beta.-Myrcene $$ 1,6-Octadiene, 7-methyl-3-methylene- $$ Myrcene $$ 7-Methyl-3-methylene-1,6-octa

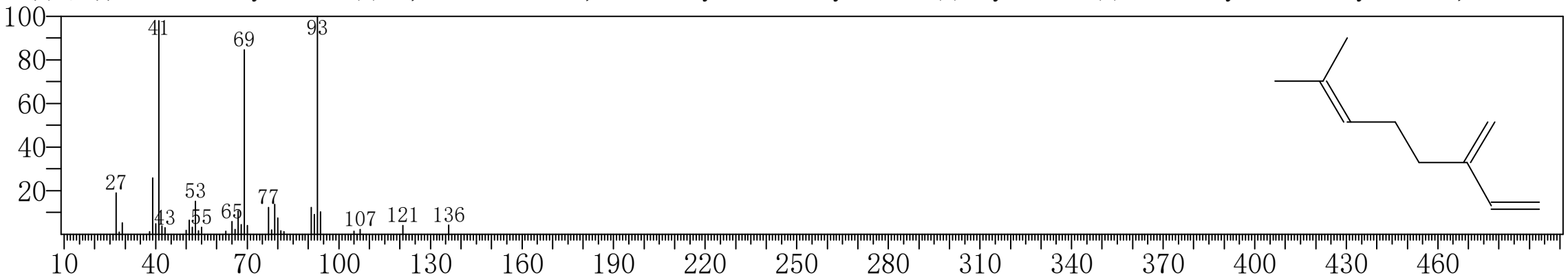

命中#:4　输入:7922　谱库:NIST17s.lib
SI:94　分子式:C10H16　CAS:123-35-3　摩尔质量:136　保留指数:958
组分名称:.beta.-Myrcene $$ 1,6-Octadiene, 7-methyl-3-methylene- $$ Myrcene $$ 7-Methyl-3-methylene-1,6-octa

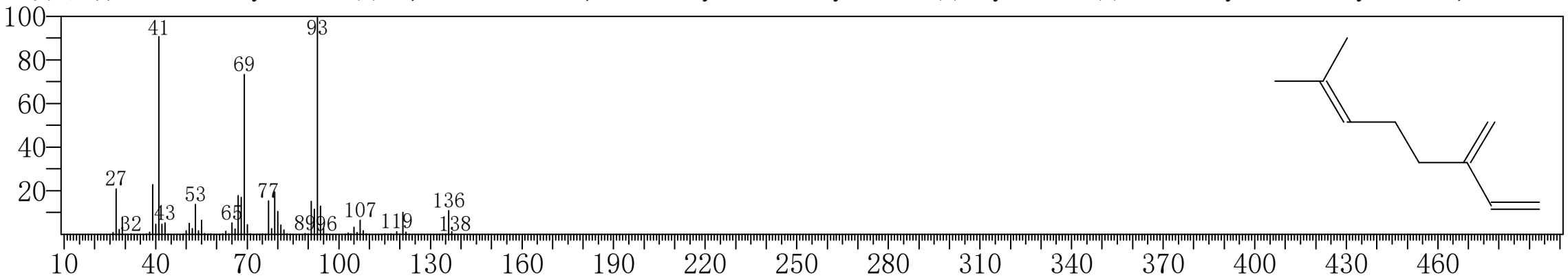

命中#:5　输入:10297　谱库:NIST17-1.lib
SI:94　分子式:C10H16　CAS:123-35-3　摩尔质量:136　保留指数:958
组分名称:.beta.-Myrcene $$ 1,6-Octadiene, 7-methyl-3-methylene- $$ Myrcene $$ 7-Methyl-3-methylene-1,6-octa

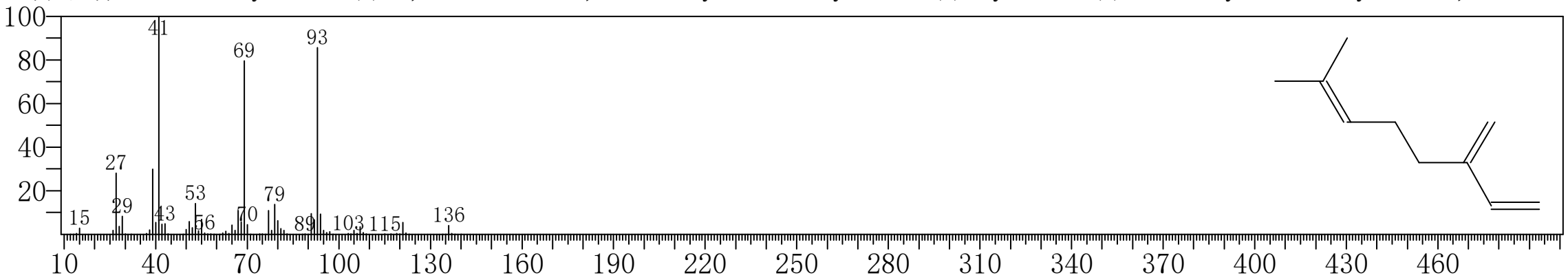

<< 目标组分 >>
行号#:9　保留时间:12.250(扫描数#:1451)　质量峰:266
原始模式:平均 12.245-12.255(1450-1452)　基峰:119.10(285068)
背景模式:峰校准　组 1 - 事件 1　Scan

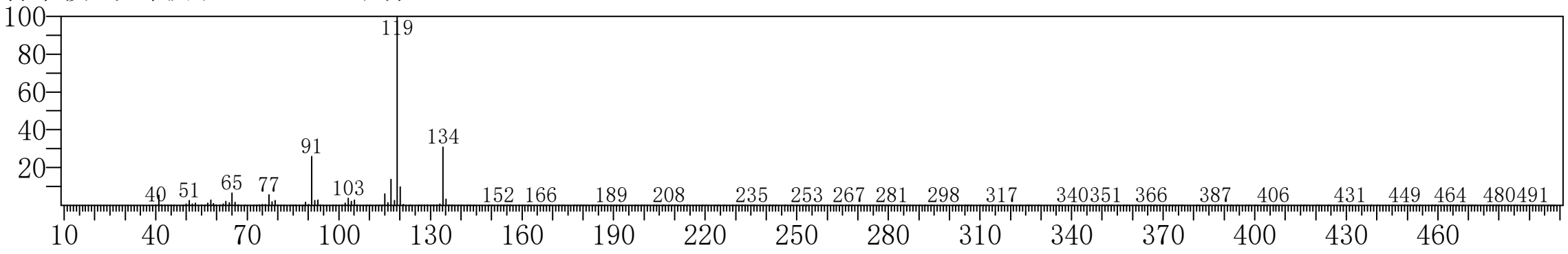

命中#:1　输入:317　谱库:FFNSC1.3.lib
SI:97　分子式:C10 H14　CAS:99-87-6　摩尔质量:134　保留指数:1025
组分名称:Cymene <para->

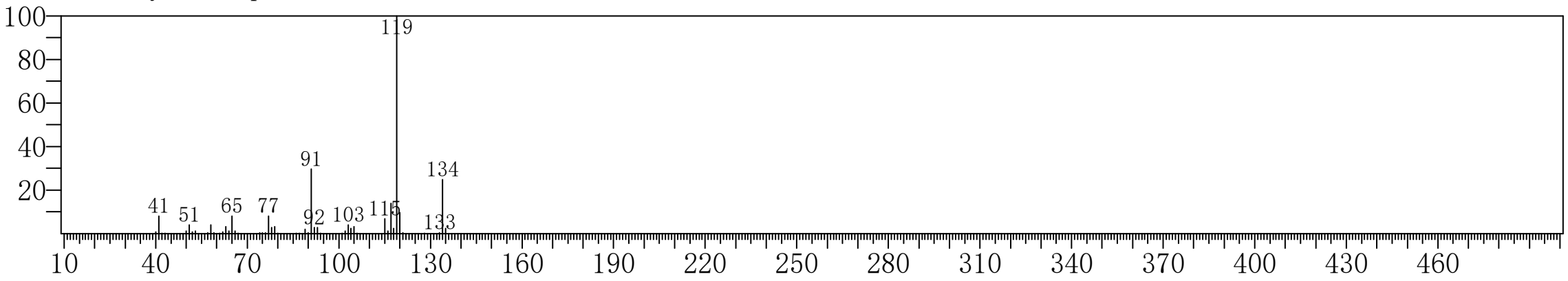

命中#:2　输入:7439　谱库:NIST17s.lib
SI:97　分子式:C10H14　CAS:527-84-4　摩尔质量:134　保留指数:1042
组分名称:o-Cymene $$ Benzene, 1-methyl-2-(1-methylethyl)- $$ o-Cymol $$ o-Isopropyltoluene $$ 1-Isopropyl-2-

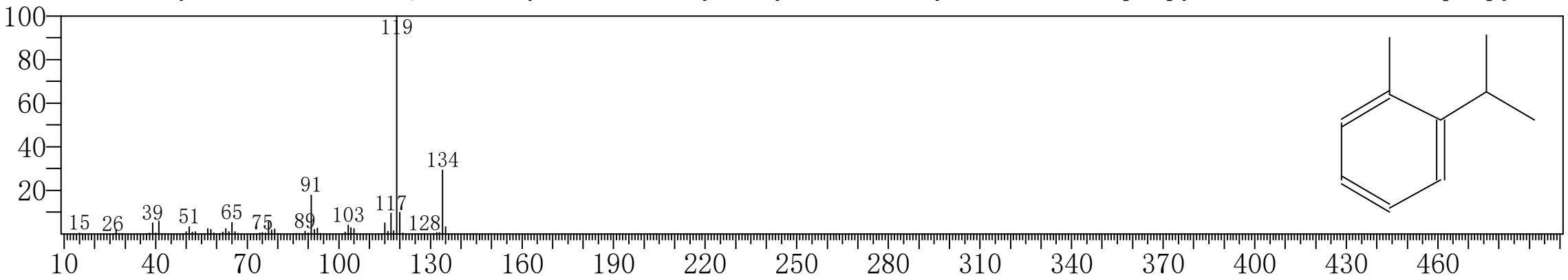

命中#:3　输入:7435　谱库:NIST17s.lib
SI:96　分子式:C10H14　CAS:535-77-3　摩尔质量:134　保留指数:1042
组分名称:Benzene, 1-methyl-3-(1-methylethyl)- $$ m-Cymene $$ .beta.-Cymene $$ m-Cymol $$ m-Isopropyltoluene

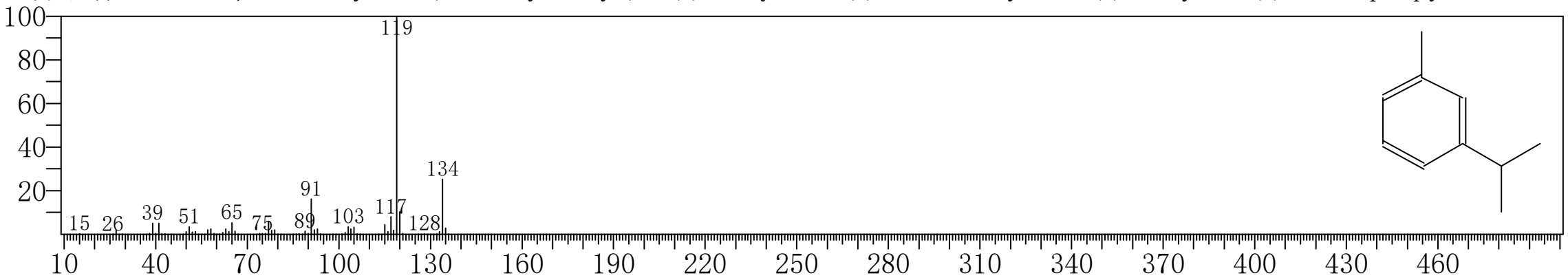

命中#:4　输入:9667　谱库:NIST17-1.lib
SI:96　分子式:C10H14　CAS:535-77-3　摩尔质量:134　保留指数:1042
组分名称:Benzene, 1-methyl-3-(1-methylethyl)- $$ m-Cymene $$ .beta.-Cymene $$ m-Cymol $$ m-Isopropyltoluene

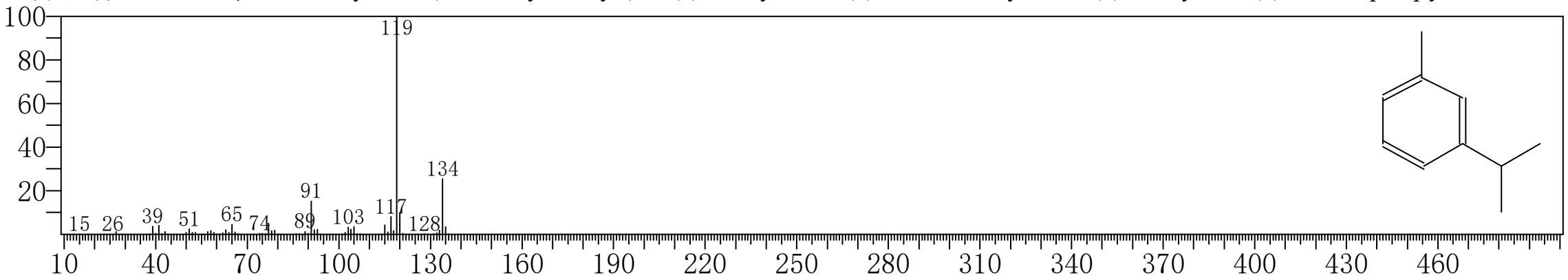

命中#:5　输入:7438　谱库:NIST17s.lib
SI:95　分子式:C10H14　CAS:527-84-4　摩尔质量:134　保留指数:1042
组分名称:o-Cymene $$ Benzene, 1-methyl-2-(1-methylethyl)- $$ o-Cymol $$ o-Isopropyltoluene $$ 1-Isopropyl-2-

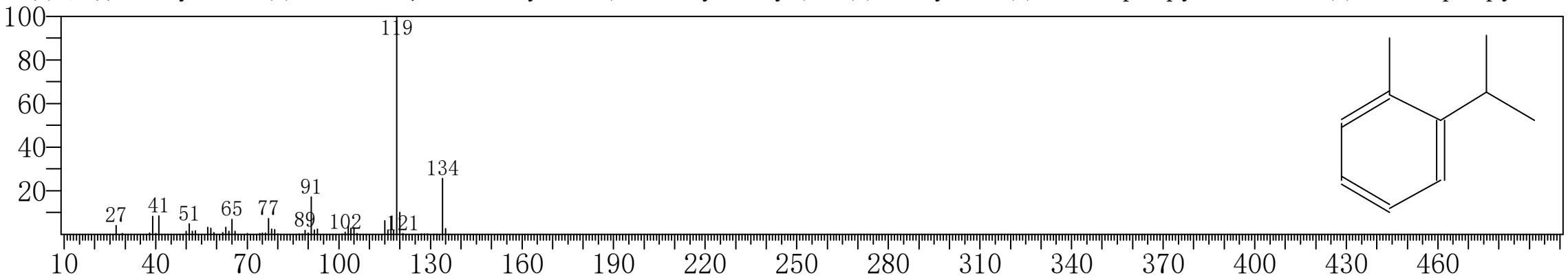

<< 目标组分 >>
行号#:10　 保留时间:12.405(扫描数#:1482)　质量峰:232
原始模式:平均 12.400-12.410(1481-1483)　 基峰:68.05(267747)
背景模式:峰校准　组 1 - 事件 1　Scan

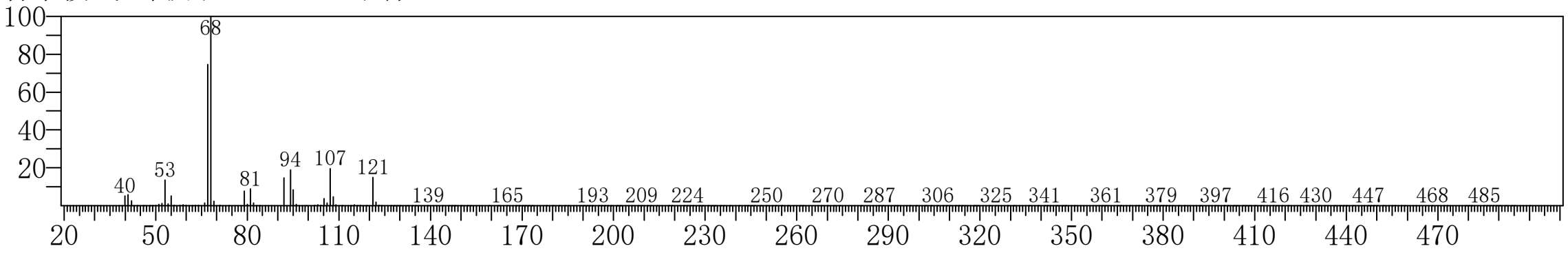

命中#:1　输入:10316　谱库:NIST17-1.lib
SI:84　分子式:C10H16　CAS:21284-05-9　摩尔质量:136　保留指数:1046
组分名称:1,5-Cyclooctadiene, 3,4-dimethyl- $$ 3,4-Dimethyl-1,5-cyclooctadiene # $$

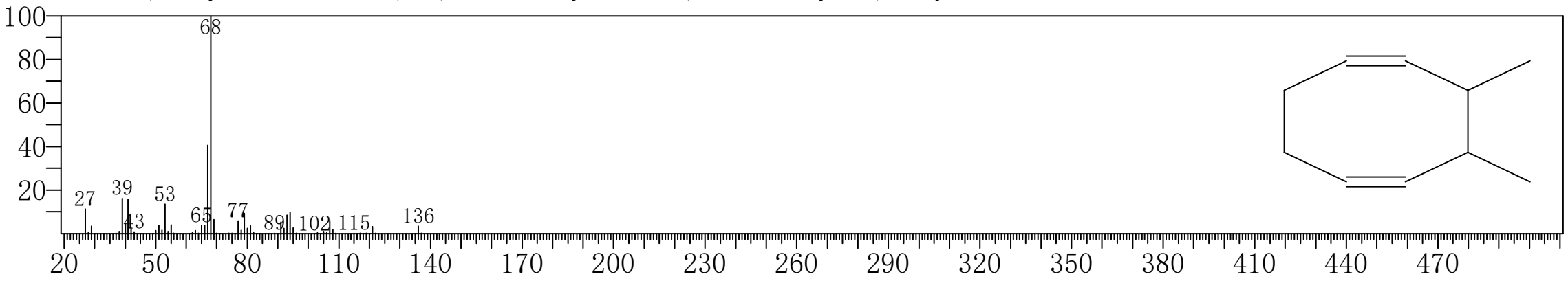

命中#:2　输入:10317　谱库:NIST17-1.lib
SI:83　分子式:C10H16　CAS:61141-62-6　摩尔质量:136　保留指数:1003
组分名称:Cyclobutane, 1,2-dicyclopropyl- $$ 1,2-Dicyclopropylcyclobutane # $$

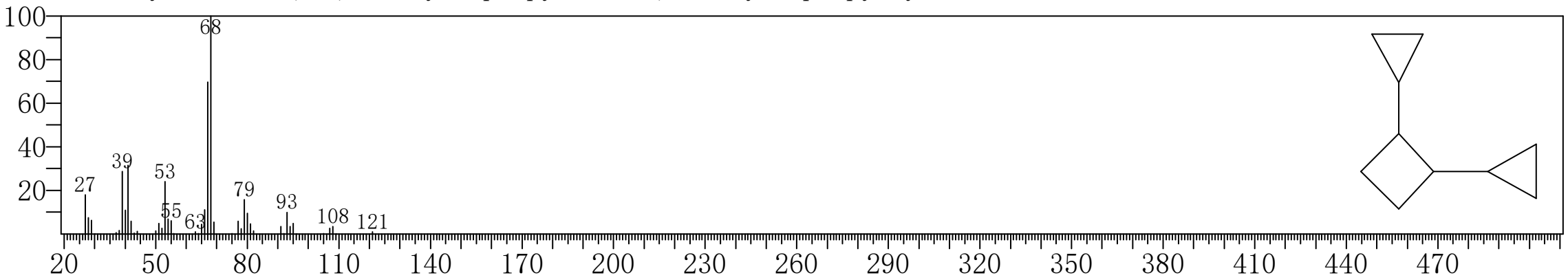

命中#:3　输入:10320　谱库:NIST17-1.lib
SI:81　分子式:C10H16　CAS:3760-13-2　摩尔质量:136　保留指数:1103
组分名称:1,5-Cyclooctadiene, 1,6-dimethyl- $$ 1,6-Dimethyl-1,5-cyclooctadiene # $$

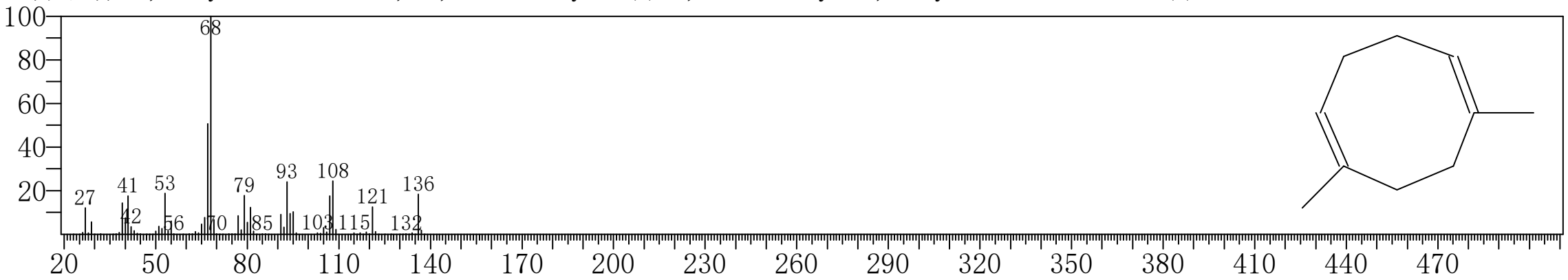

命中#:4　输入:10319　谱库:NIST17-1.lib
SI:80　分子式:C10H16　CAS:0-00-0　摩尔质量:136　保留指数:903
组分名称:Cyclobutane, 1,2-diethenyl-3,4-dimethyl- $$ 1,2-Dimethyl-3,4-divinylcyclobutane # $$

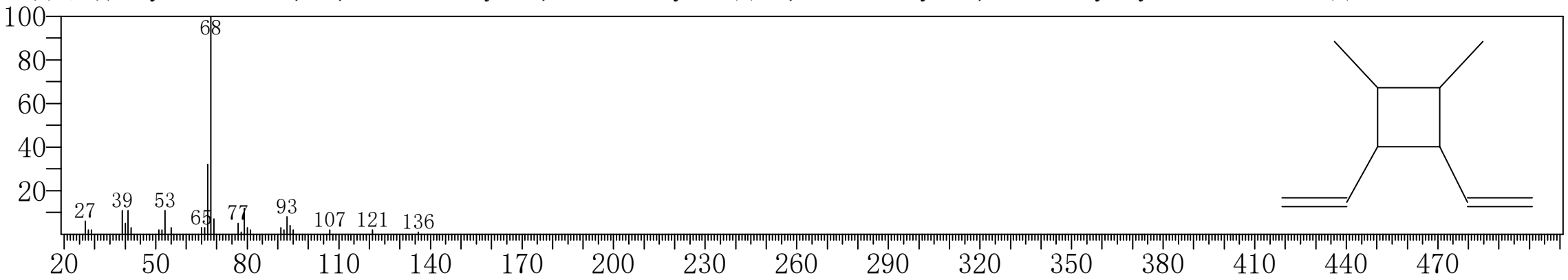

命中#:5　输入:10323　谱库:NIST17-1.lib
SI:80　分子式:C10H16　CAS:1743-61-9　摩尔质量:136　保留指数:1015
组分名称:Cyclohexene, 4-ethenyl-1,4-dimethyl- $$ 1,4-Dimethyl-4-vinylcyclohexene $$ 1,4-Dimethyl-4-vinyl-1-c

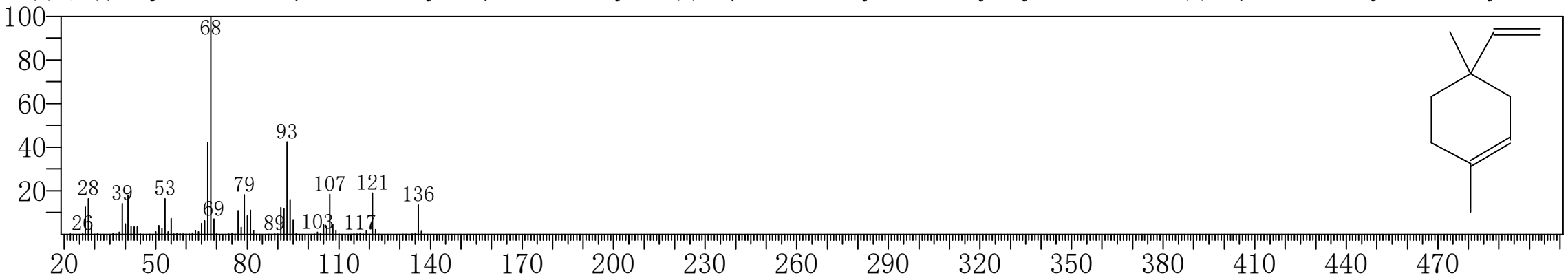

<< 目标组分 >>
行号#:11　保留时间:12.435(扫描数#:1488)　质量峰:245
原始模式:平均 12.430-12.440(1487-1489)　基峰:93.10(486993)
背景模式:峰校准　组 1 - 事件 1　Scan

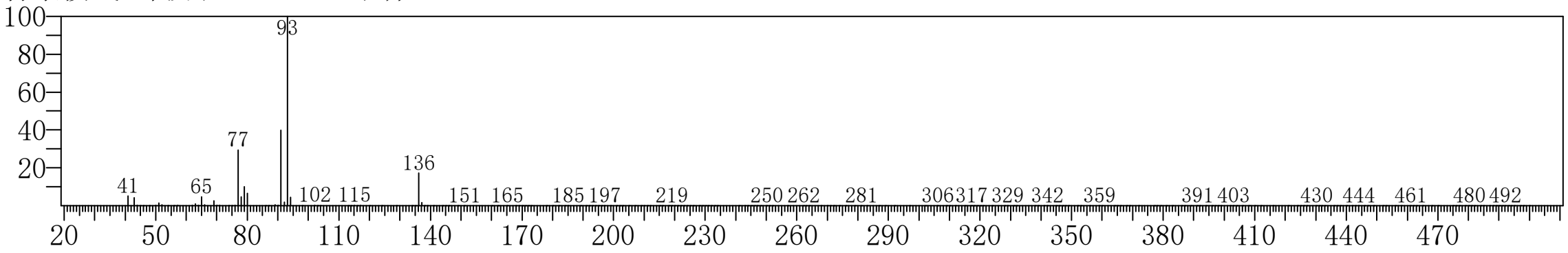

命中#:1　输入:7955　谱库:NIST17s.lib
SI:91　分子式:C10H16　CAS:99-83-2　摩尔质量:136　保留指数:969
组分名称:.alpha.-Phellandrene $$ 1,3-Cyclohexadiene, 2-methyl-5-(1-methylethyl)- $$ .alpha.-Fellandrene $$ p

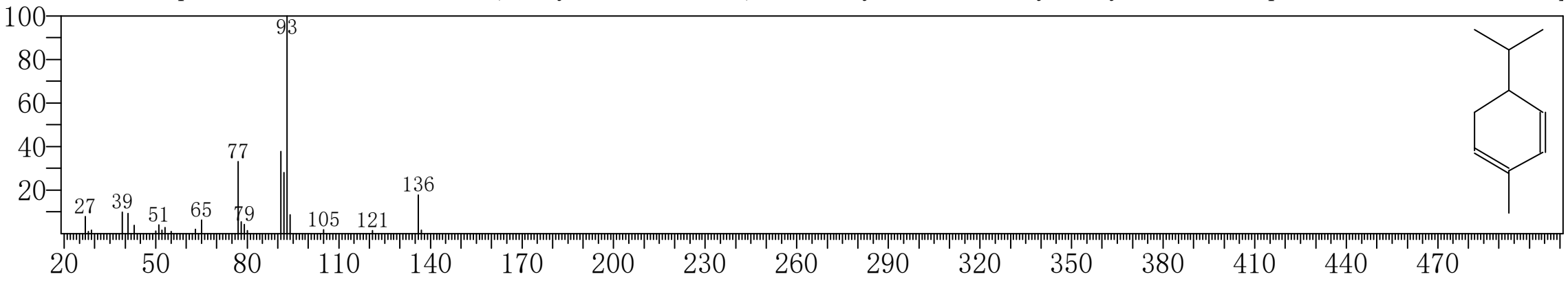

命中#:2　输入:7952　谱库:NIST17s.lib
SI:90　分子式:C10H16　CAS:99-83-2　摩尔质量:136　保留指数:969
组分名称:.alpha.-Phellandrene $$ 1,3-Cyclohexadiene, 2-methyl-5-(1-methylethyl)- $$ .alpha.-Fellandrene $$ p

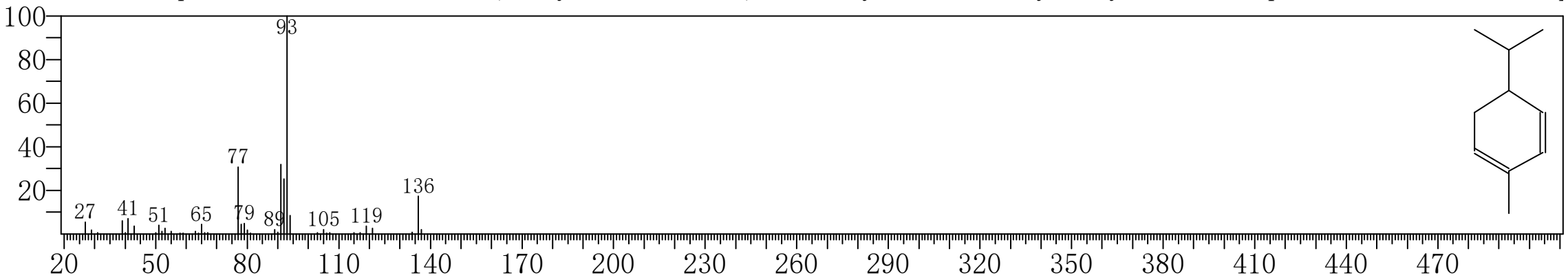

命中#:3　输入:7954　谱库:NIST17s.lib
SI:90　分子式:C10H16　CAS:99-83-2　摩尔质量:136　保留指数:969
组分名称:.alpha.-Phellandrene $$ 1,3-Cyclohexadiene, 2-methyl-5-(1-methylethyl)- $$ .alpha.-Fellandrene $$ p

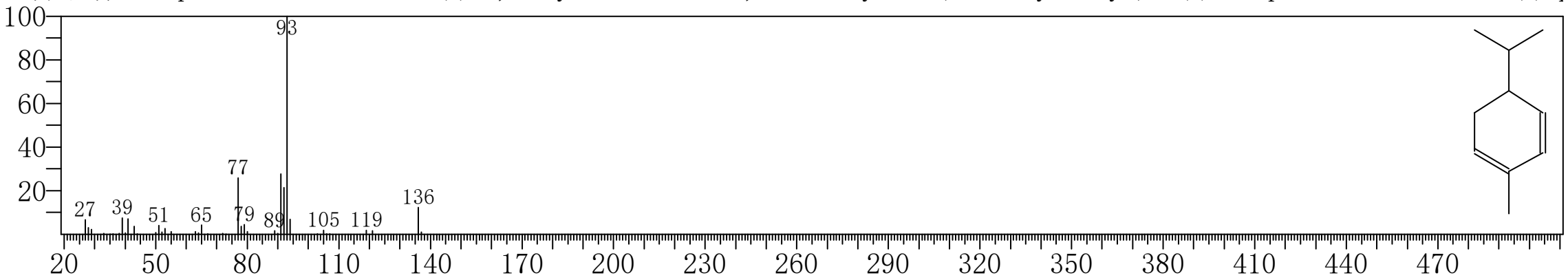

命中#:4　输入:648　谱库:FFNSC1.3.lib
SI:90　分子式:C10 H16　CAS:99-83-2　摩尔质量:136　保留指数:1007
组分名称:Phellandrene <alpha->

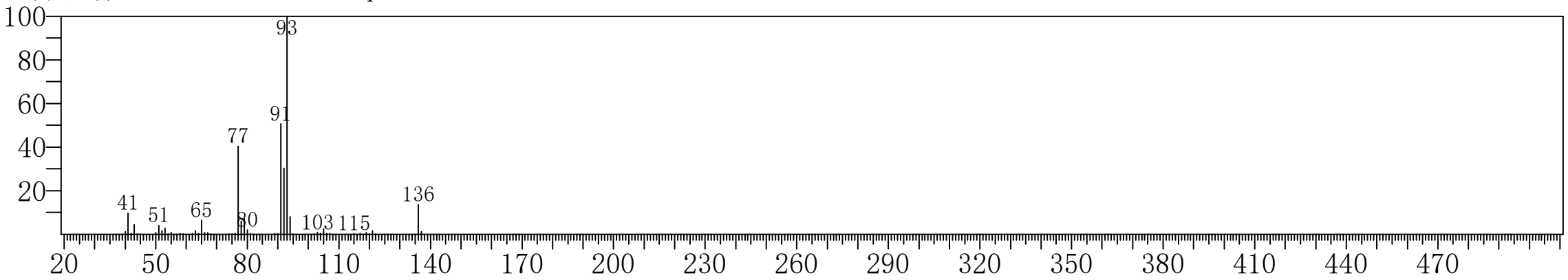

命中#:5　输入:10369　谱库:NIST17-1.lib
SI:90　分子式:C10H16　CAS:555-10-2　摩尔质量:136　保留指数:964
组分名称:.beta.-Phellandrene $$ Cyclohexene, 3-methylene-6-(1-methylethyl)- $$ p-Mentha-1(7),2-diene $$ Phe

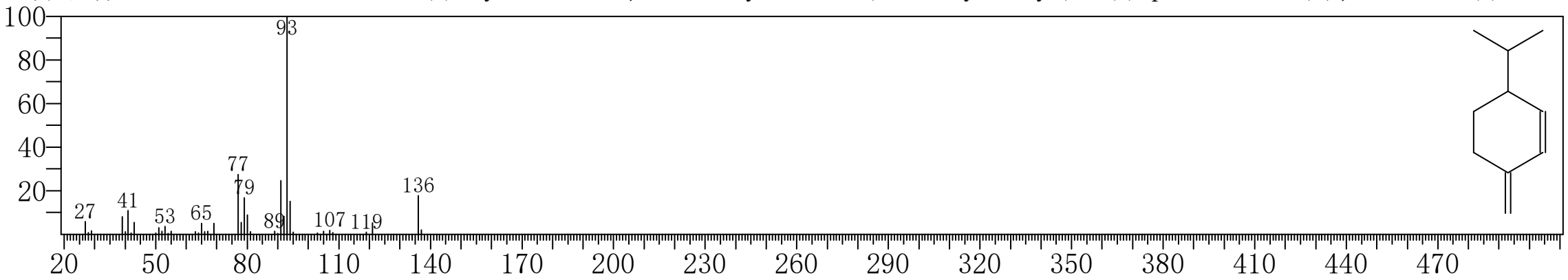

<< 目标组分 >>
行号#:12　保留时间:13.345(扫描数#:1670)　质量峰:255
原始模式:平均 13.340-13.350(1669-1671)　基峰:93.10(121734)
背景模式:峰校准　组 1 - 事件 1　Scan

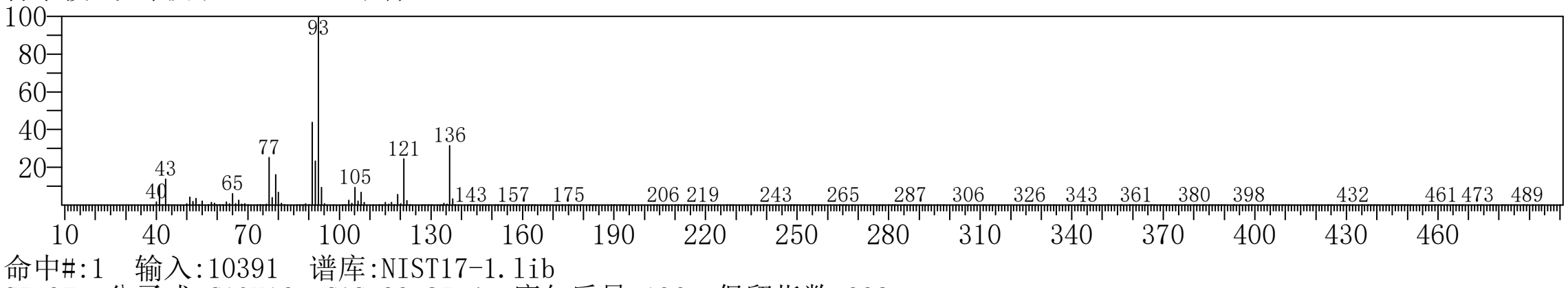

命中#:1　输入:10391　谱库:NIST17-1.lib
SI:97　分子式:C10H16　CAS:99-85-4　摩尔质量:136　保留指数:998
组分名称:.gamma.-Terpinene $$ 1,4-Cyclohexadiene, 1-methyl-4-(1-methylethyl)- $$ .gamma.-Terpinen $$ p-Mentl

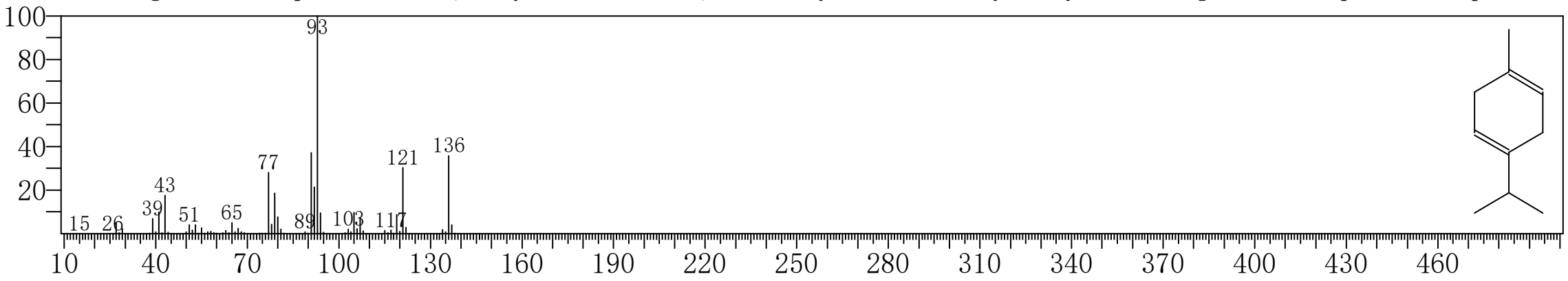

命中#:2　输入:818　谱库:FFNSC1.3.lib
SI:95　分子式:C10 H16　CAS:99-85-4　摩尔质量:136　保留指数:1058
组分名称:Terpinene <gamma->

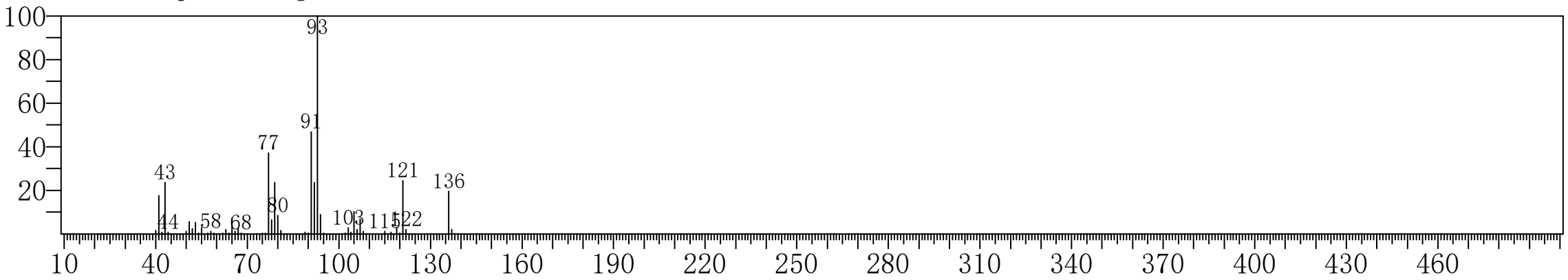

命中#:3　输入:7959　谱库:NIST17s.lib
SI:95　分子式:C10H16　CAS:99-85-4　摩尔质量:136　保留指数:998
组分名称:.gamma.-Terpinene $$ 1,4-Cyclohexadiene, 1-methyl-4-(1-methylethyl)- $$ .gamma.-Terpinen $$ p-Mentl

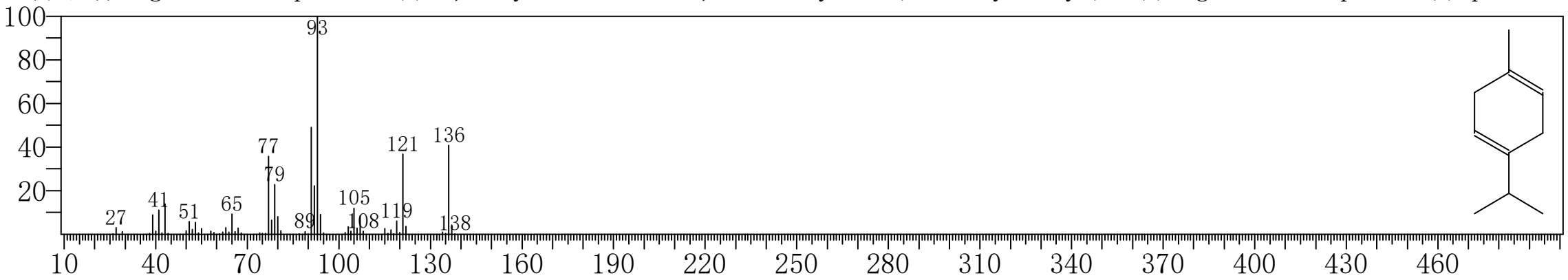

命中#:4　输入:7949　谱库:NIST17s.lib
SI:94　分子式:C10H16　CAS:99-85-4　摩尔质量:136　保留指数:998
组分名称:.gamma.-Terpinene $$ 1,4-Cyclohexadiene, 1-methyl-4-(1-methylethyl)- $$ .gamma.-Terpinen $$ p-Mentl

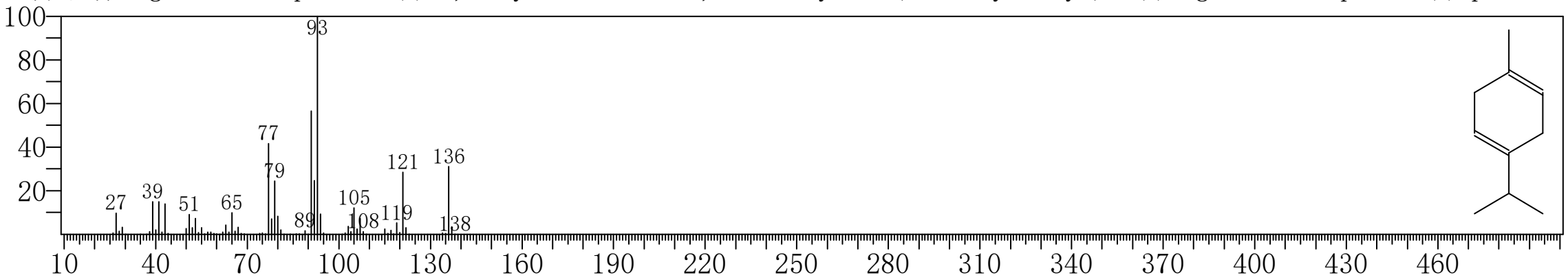

命中#:5　输入:7948　谱库:NIST17s.lib
SI:94　分子式:C10H16　CAS:99-85-4　摩尔质量:136　保留指数:998
组分名称:.gamma.-Terpinene $$ 1,4-Cyclohexadiene, 1-methyl-4-(1-methylethyl)- $$ .gamma.-Terpinen $$ p-Mentl

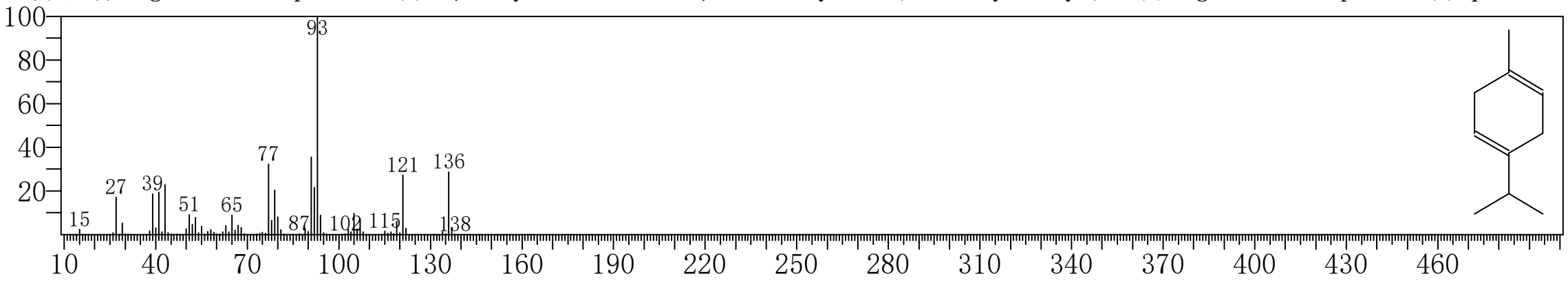

<< 目标组分 >>
行号#:13　保留时间:13.810(扫描数#:1763)　质量峰:235
原始模式:平均 13.805-13.815(1762-1764)　基峰:79.05(165131)
背景模式:峰校准　组 1 - 事件 1　Scan

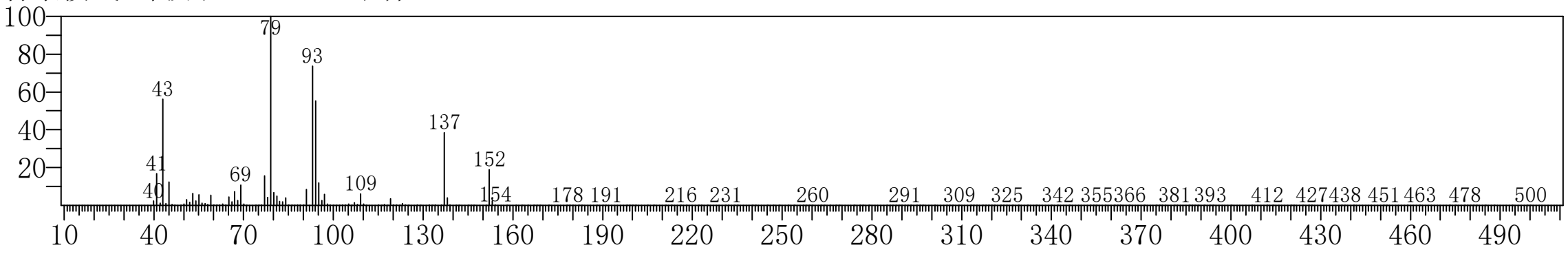

命中#:1　输入:15456　谱库:NIST17s.lib
SI:81　分子式:C10H18O2　CAS:6252-34-2　摩尔质量:170　保留指数:1331
组分名称:3-Cyclohexene-1-methanol, 2-hydroxy-.alpha.,.alpha.,4-trimethyl- $$ 6-(1-Hydroxy-1-methylethyl)-3-r

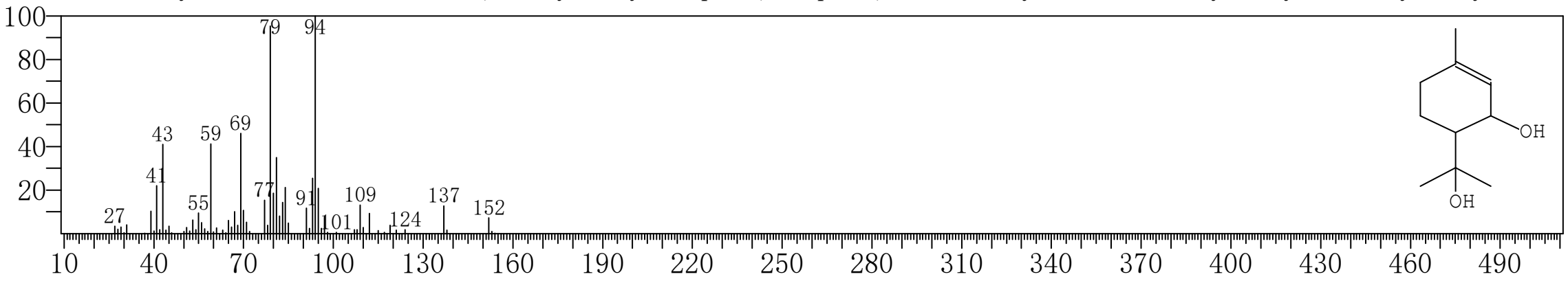

命中#:2　输入:59542　谱库:NIST17-1.lib
SI:79　分子式:C12H20O3　CAS:121958-61-0　摩尔质量:212　保留指数:1455
组分名称:2-((1R,4R)-4-Hydroxy-4-methylcyclohex-2-enyl)propan-2-yl acetate $$ 2-Cyclohexene-1-methanol, 4-hy

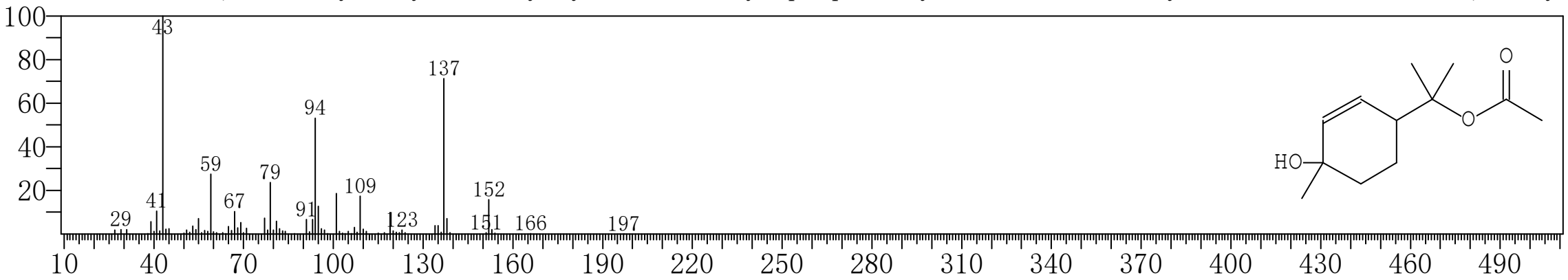

命中#:3　输入:28291　谱库:NIST17-1.lib
SI:78　分子式:C10H18O2　CAS:6252-34-2　摩尔质量:170　保留指数:1331
组分名称:3-Cyclohexene-1-methanol, 2-hydroxy-.alpha.,.alpha.,4-trimethyl- $$ 6-(1-Hydroxy-1-methylethyl)-3-r

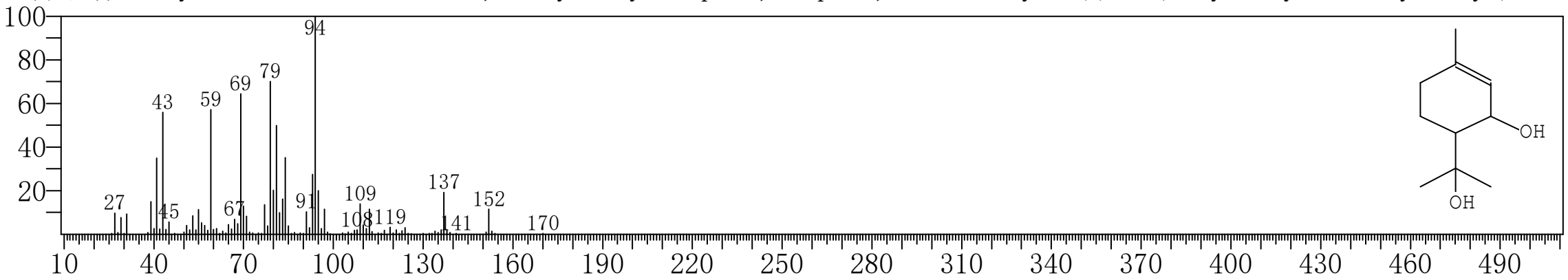

命中#:4　输入:18503　谱库:NIST17-1.lib
SI:78　分子式:C9H14O2　CAS:0-00-0　摩尔质量:154　保留指数:1129
组分名称:1,2,3,6-Tetrahydrobenzylalcohol, acetate

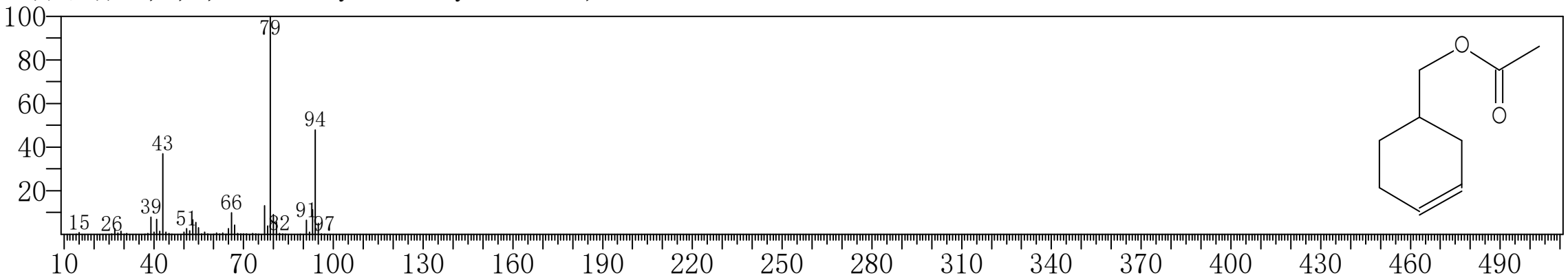

命中#:5　输入:59535　谱库:NIST17-1.lib
SI:78　分子式:C12H20O3　CAS:0-00-0　摩尔质量:212　保留指数:1455
组分名称:1-Methyl-4-(1-acetoxy-1-methylethyl)-cyclohex-2-enol $$ 1-(4-Hydroxy-4-methyl-2-cyclohexen-1-yl)-1-

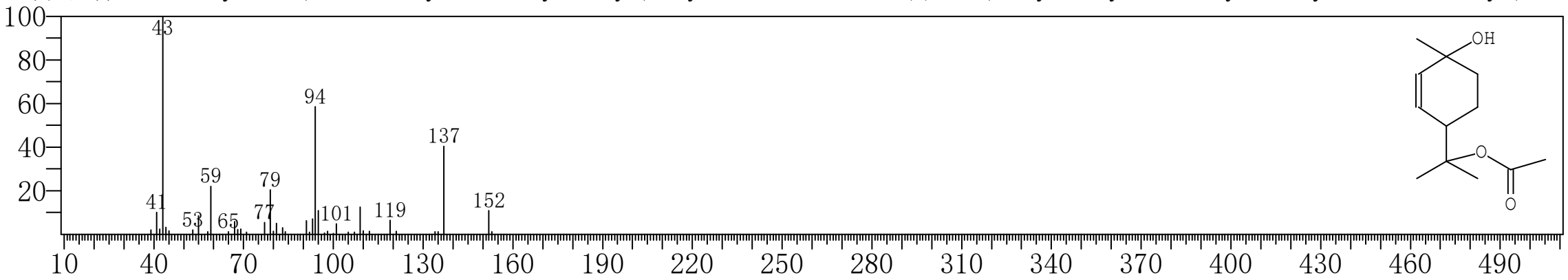

<< 目标组分 >>
行号#:14　保留时间:14.210(扫描数#:1843)　质量峰:254
原始模式:平均 14.205-14.215(1842-1844)　基峰:93.10(204916)
背景模式:峰校准　组 1 - 事件 1　Scan

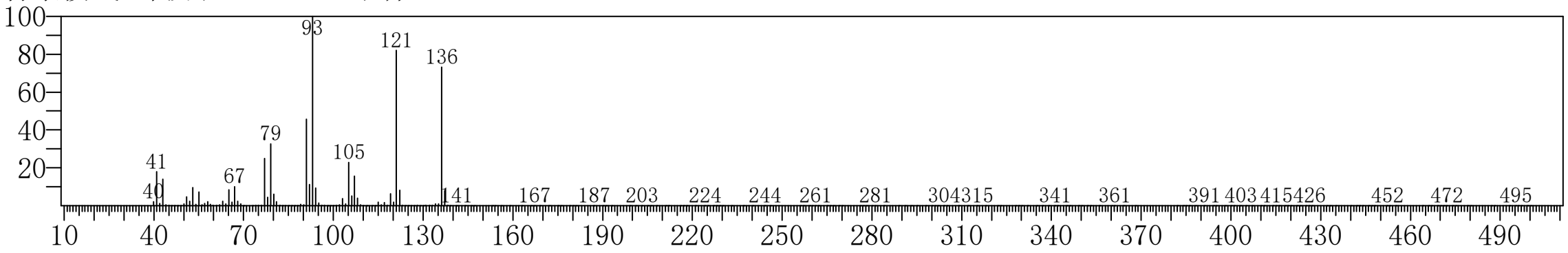

命中#:1　输入:10407　谱库:NIST17-1.lib
SI:96　分子式:C10H16　CAS:29050-33-7　摩尔质量:136　保留指数:919
组分名称:(+)-4-Carene $$ 4,7,7-Trimethylbicyclo[4.1.0]hept-2-ene # $$

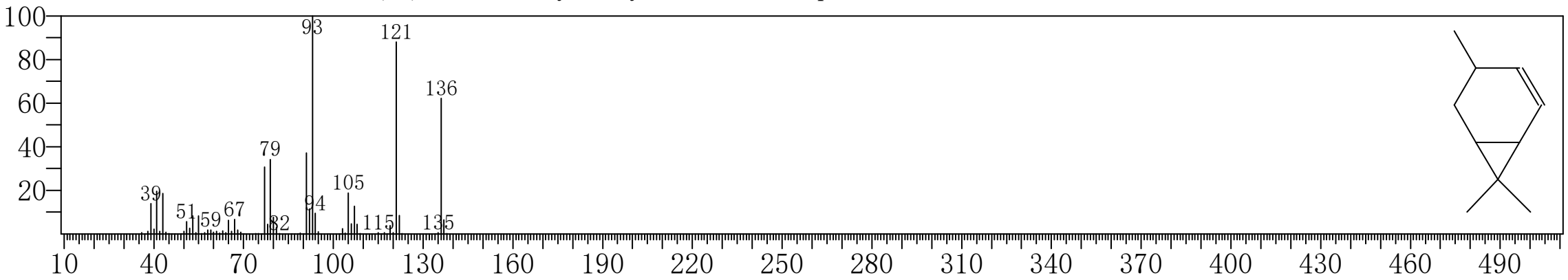

命中#:2　输入:603　谱库:FFNSC1.3.lib
SI:95　分子式:C10 H16　CAS:586-62-9　摩尔质量:136　保留指数:1086
组分名称:Terpinolene

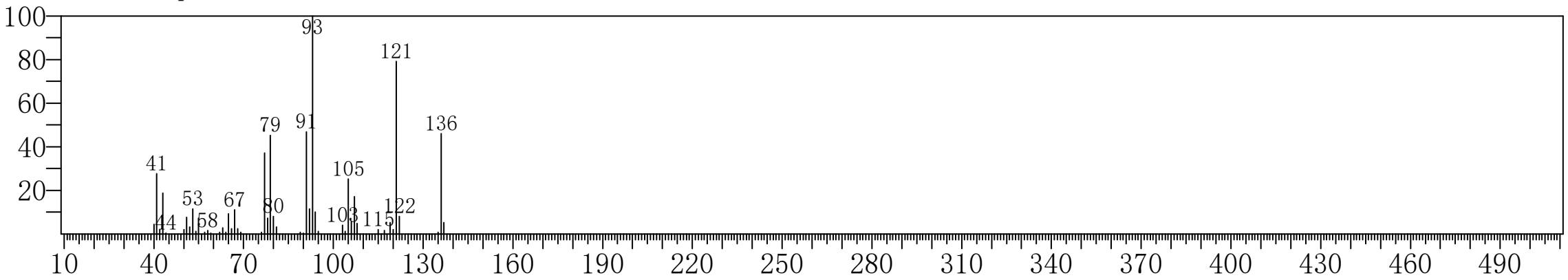

命中#:3　输入:7983　谱库:NIST17s.lib
SI:95　分子式:C10H16　CAS:586-62-9　摩尔质量:136　保留指数:1052
组分名称:Cyclohexene, 1-methyl-4-(1-methylethylidene)- $$ p-Mentha-1,4(8)-diene $$ Terpinolene $$ Terpinoler

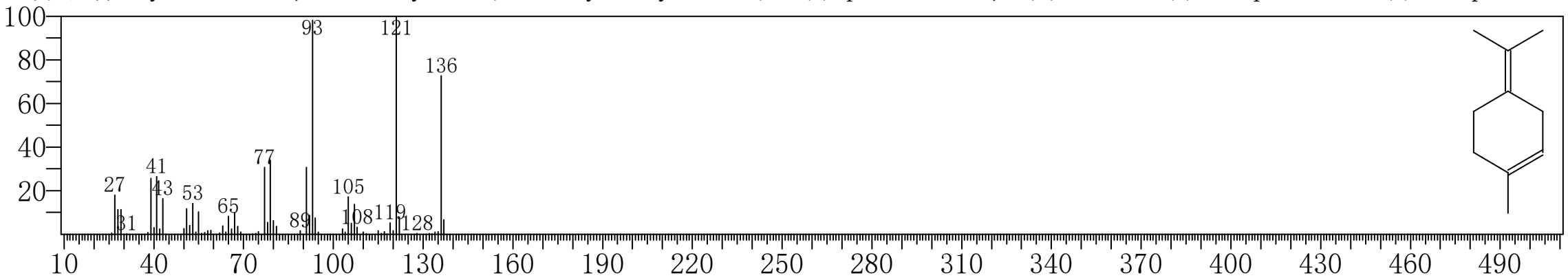

命中#:4　输入:10406　谱库:NIST17-1.lib
SI:95　分子式:C10H16　CAS:554-61-0　摩尔质量:136　保留指数:948
组分名称:2-Carene $$ Bicyclo[4.1.0]hept-2-ene, 3,7,7-trimethyl- $$ .delta.-2-Carene $$ (.+/-.)-2-Carene $$:

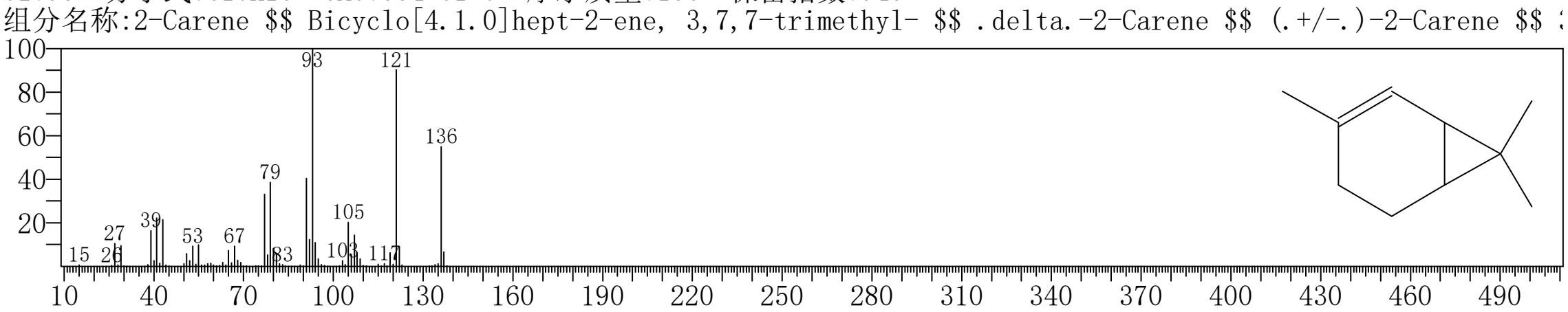

命中#:5　输入:7984　谱库:NIST17s.lib
SI:95　分子式:C10H16　CAS:586-62-9　摩尔质量:136　保留指数:1052
组分名称:Cyclohexene, 1-methyl-4-(1-methylethylidene)- $$ p-Mentha-1,4(8)-diene $$ Terpinolene $$ Terpinoler

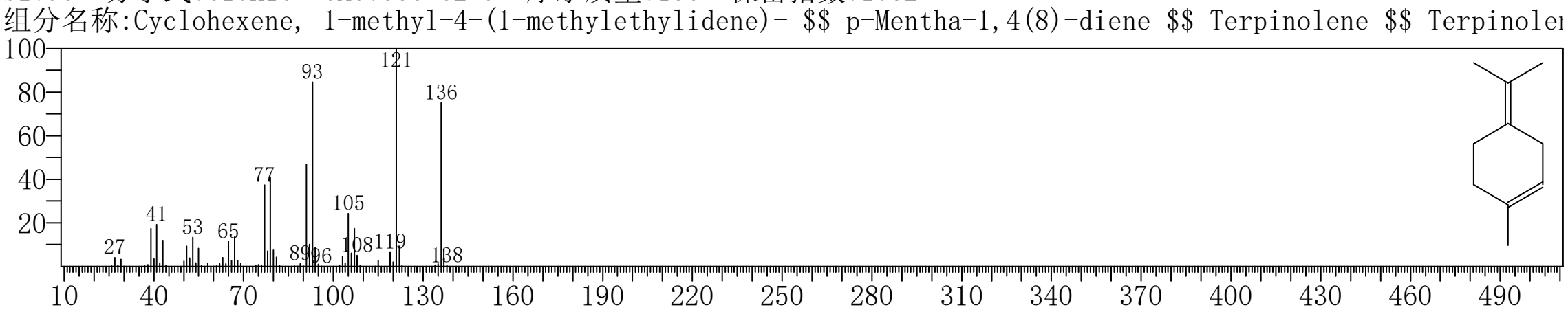

<< 目标组分 >>
行号#:15　保留时间:14.930(扫描数#:1987)　质量峰:214
原始模式:平均 14.925-14.935(1986-1988)　基峰:121.10(230640)
背景模式:峰校准　组 1 - 事件 1　Scan

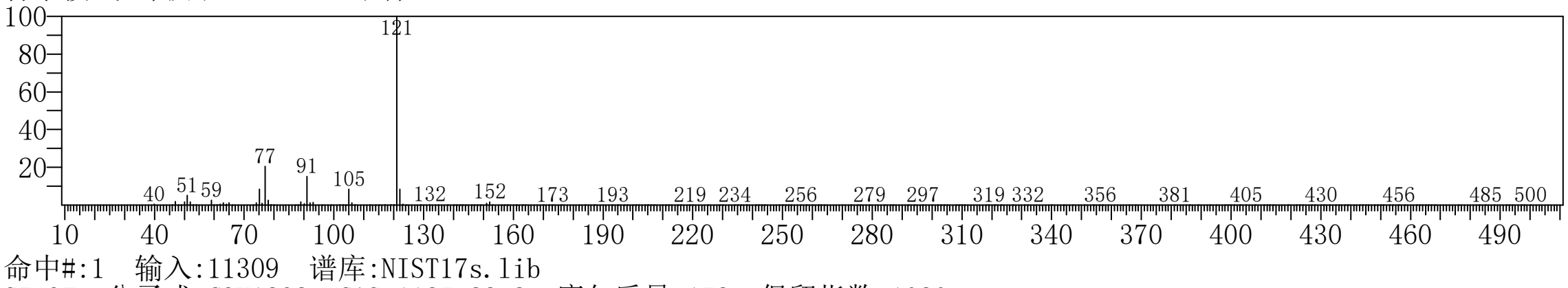

命中#:1　输入:11309　谱库:NIST17s.lib
SI:97　分子式:C9H12O2　CAS:1125-88-8　摩尔质量:152　保留指数:1080
组分名称:Benzaldehyde dimethyl acetal $$ Benzene, (dimethoxymethyl)- $$ .alpha.,.alpha.-Dimethoxytoluene $$

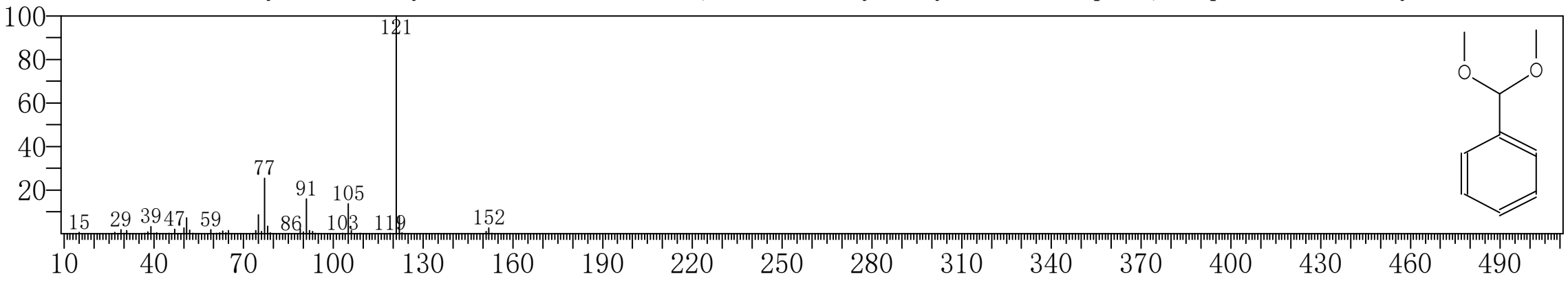

命中#:2　输入:11312　谱库:NIST17s.lib
SI:97　分子式:C9H12O2　CAS:1125-88-8　摩尔质量:152　保留指数:1080
组分名称:Benzaldehyde dimethyl acetal $$ Benzene, (dimethoxymethyl)- $$ .alpha.,.alpha.-Dimethoxytoluene $$

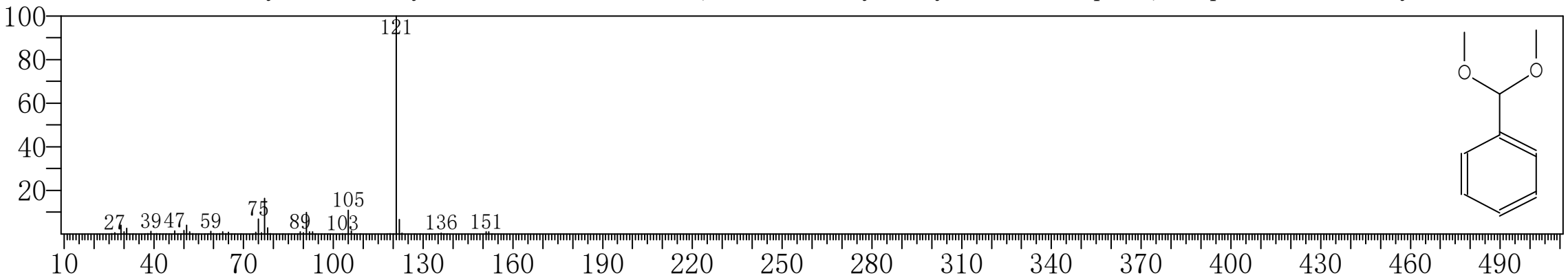

命中#:3　输入:17337　谱库:NIST17-1.lib
SI:96　分子式:C9H12O2　CAS:1125-88-8　摩尔质量:152　保留指数:1080
组分名称:Benzaldehyde dimethyl acetal $$ Benzene, (dimethoxymethyl)- $$ .alpha.,.alpha.-Dimethoxytoluene $$

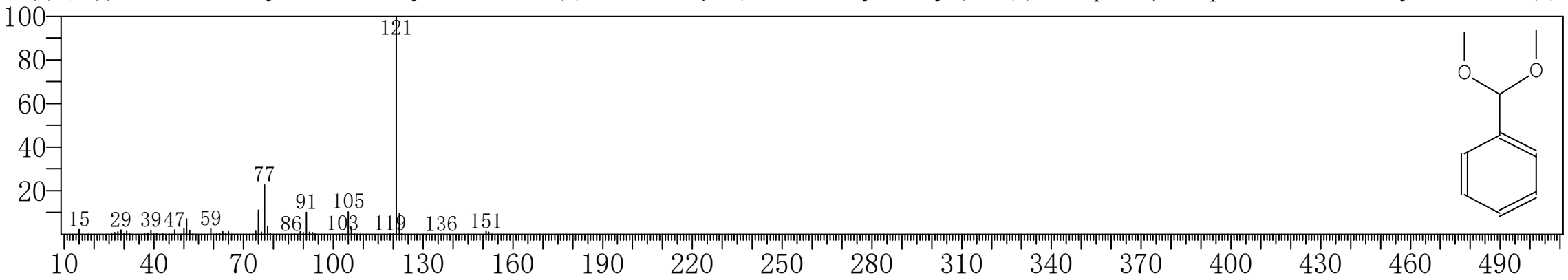

命中#:4　输入:201　谱库:FFNSC1.3.lib
SI:96　分子式:C9 H12 O2　CAS:1125-88-8　摩尔质量:152　保留指数:1108
组分名称:Benzaldehyde <dimethyl-> acetal

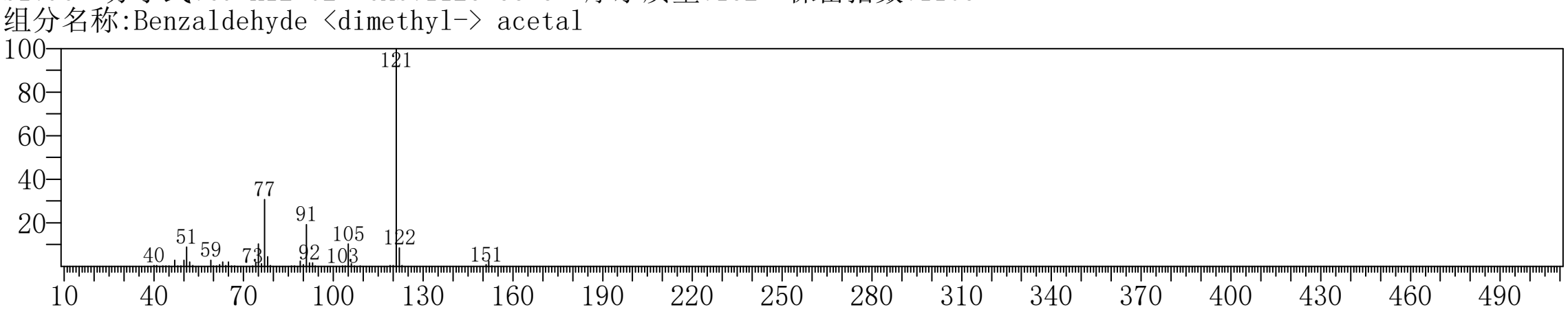

命中#:5　输入:11311　谱库:NIST17s.lib
SI:94　分子式:C9H12O2　CAS:1125-88-8　摩尔质量:152　保留指数:1080
组分名称:Benzaldehyde dimethyl acetal $$ Benzene, (dimethoxymethyl)- $$ .alpha.,.alpha.-Dimethoxytoluene $$

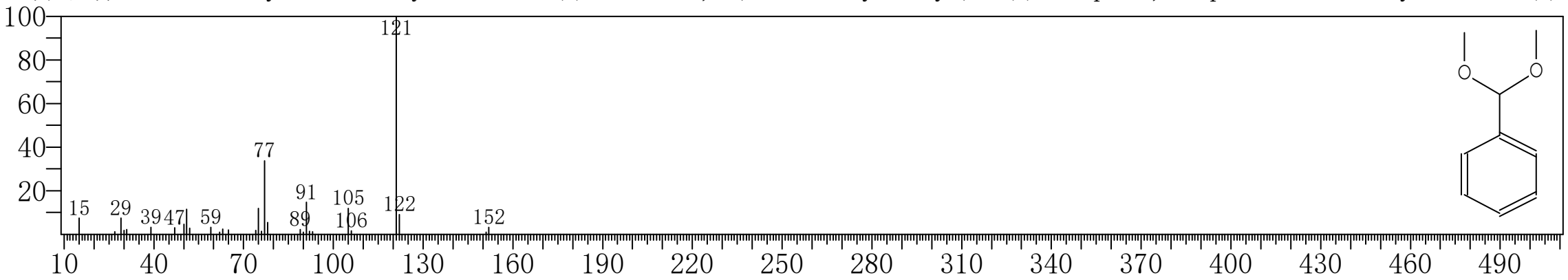

<< 目标组分 >>
行号#:16　保留时间:15.300(扫描数#:2061)　质量峰:262
原始模式:平均 15.295-15.305(2060-2062)　基峰:81.05(64770)
背景模式:峰校准　组 1 - 事件 1　Scan

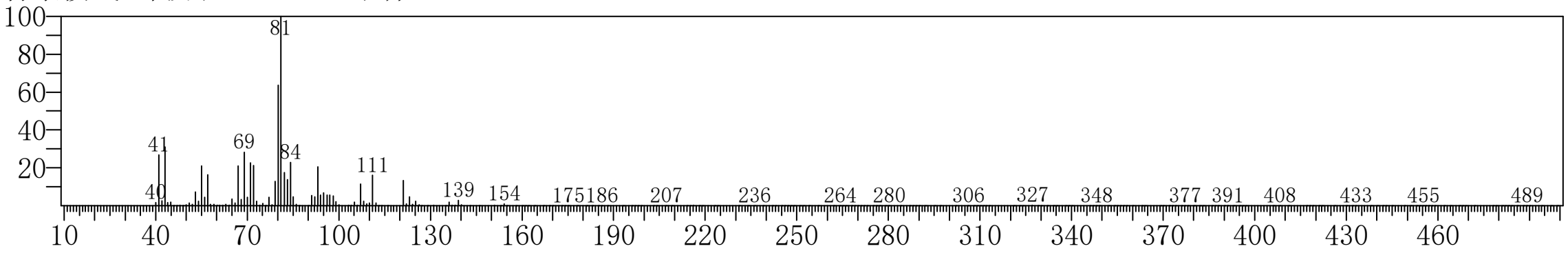

命中#:1　输入:909　谱库:FFNSC1.3.lib
SI:98　分子式:C10 H18 O　CAS:1632-73-1　摩尔质量:154　保留指数:1123
组分名称:Fenchyl alcohol

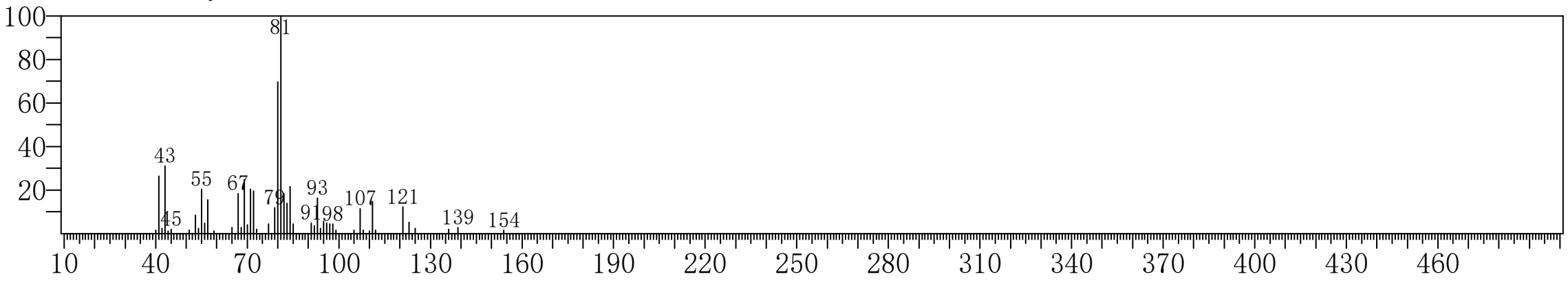

命中#:2　输入:11996　谱库:NIST17s.lib
SI:97　分子式:C10H18O　CAS:1632-73-1　摩尔质量:154　保留指数:1138
组分名称:Fenchol $$ Bicyclo[2.2.1]heptan-2-ol, 1,3,3-trimethyl- $$ 2-Norbornanol, 1,3,3-trimethyl- $$ Fenchy

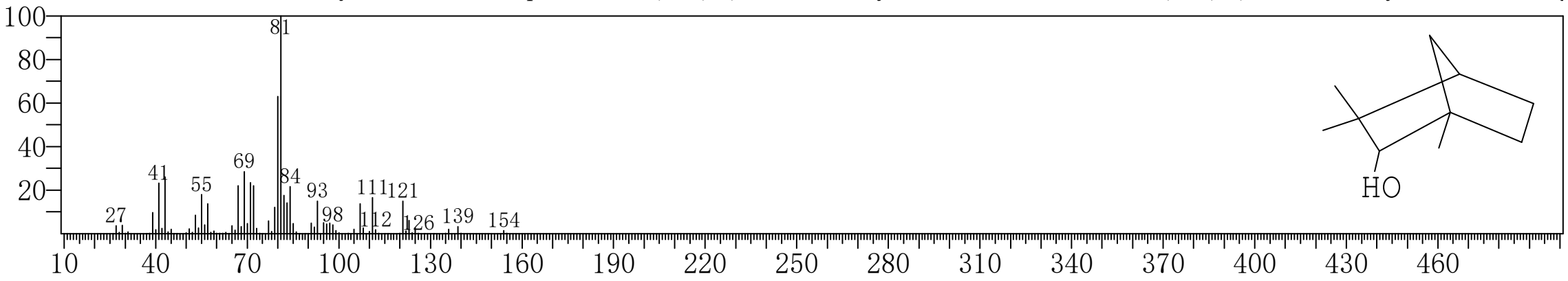

命中#:3　输入:18823　谱库:NIST17-1.lib
SI:96　分子式:C10H18O　CAS:1632-73-1　摩尔质量:154　保留指数:1138
组分名称:Fenchol $$ Bicyclo[2.2.1]heptan-2-ol, 1,3,3-trimethyl- $$ 2-Norbornanol, 1,3,3-trimethyl- $$ Fenchy

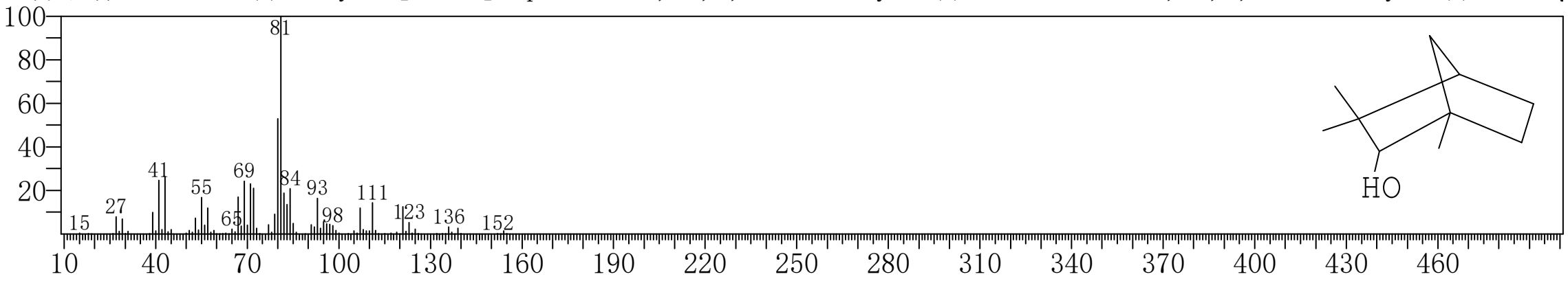

命中#:4　输入:11993　谱库:NIST17s.lib
SI:96　分子式:C10H18O　CAS:1632-73-1　摩尔质量:154　保留指数:1138
组分名称:Fenchol $$ Bicyclo[2.2.1]heptan-2-ol, 1,3,3-trimethyl- $$ 2-Norbornanol, 1,3,3-trimethyl- $$ Fenchy

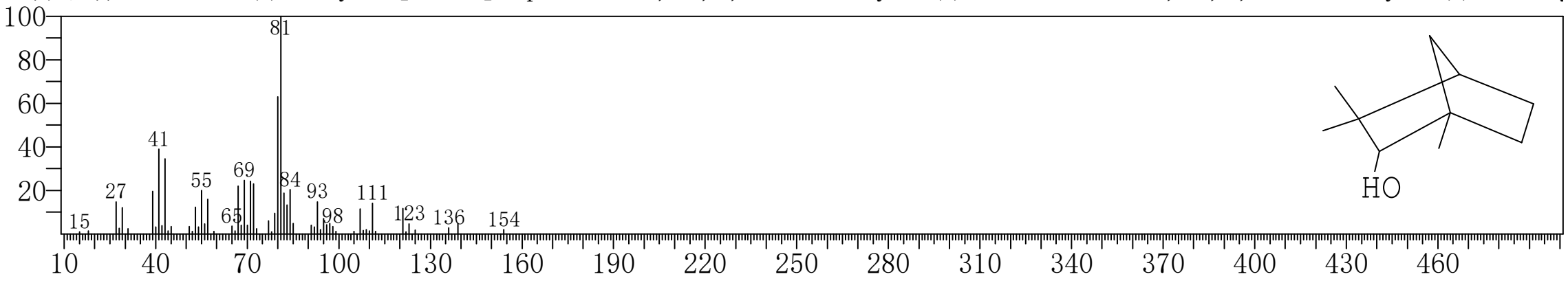

命中#:5　输入:11994　谱库:NIST17s.lib
SI:95　分子式:C10H18O　CAS:1632-73-1　摩尔质量:154　保留指数:1138
组分名称:Fenchol $$ Bicyclo[2.2.1]heptan-2-ol, 1,3,3-trimethyl- $$ 2-Norbornanol, 1,3,3-trimethyl- $$ Fenchy

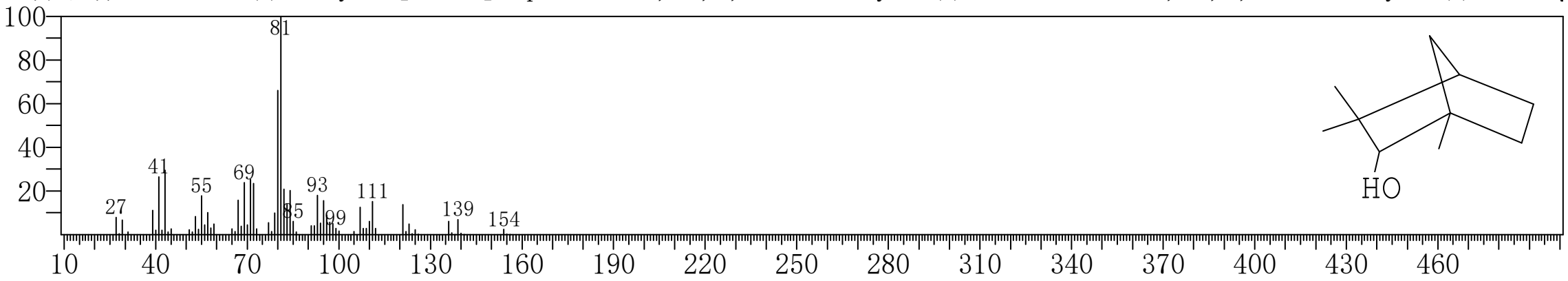

<< 目标组分 >>
行号#:17　保留时间:15.490(扫描数#:2099)　质量峰:257
原始模式:平均 15.485-15.495(2098-2100)　基峰:108.10(105194)
背景模式:峰校准　组 1 - 事件 1　Scan

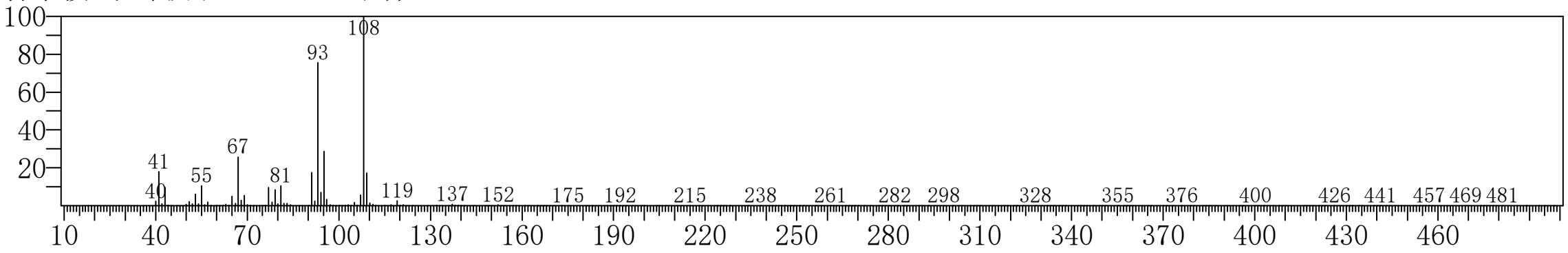

命中#:1　输入:1461　谱库:FFNSC1.3.lib
SI:94　分子式:C10 H16 O　CAS:91819-58-8　摩尔质量:152　保留指数:1126
组分名称:Campholenic aldehyde <alpha->

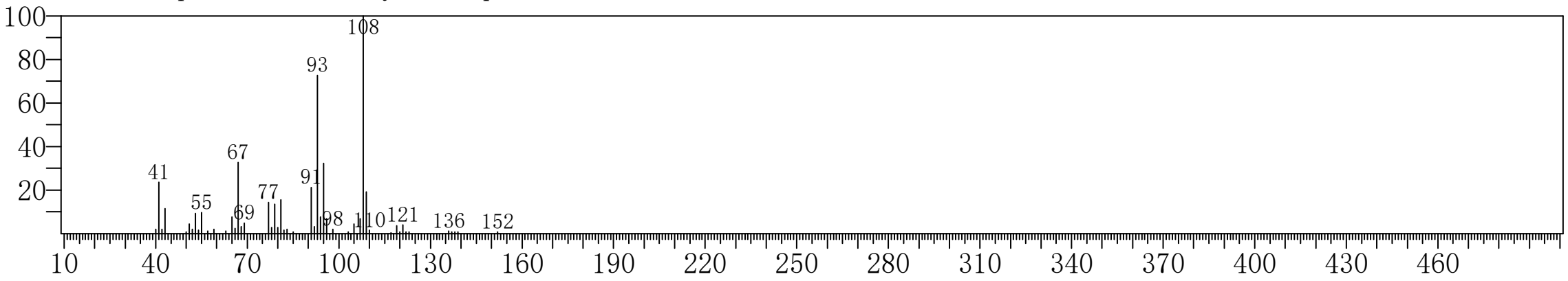

命中#:2　输入:11474　谱库:NIST17s.lib
SI:94　分子式:C10H16O　CAS:4501-58-0　摩尔质量:152　保留指数:1155
组分名称:.alpha.-Campholenal $$ (R)-.alpha.-Campholene aldehyde $$ (R)-2-(2,2,3-Trimethylcyclopent-3-en-1-y

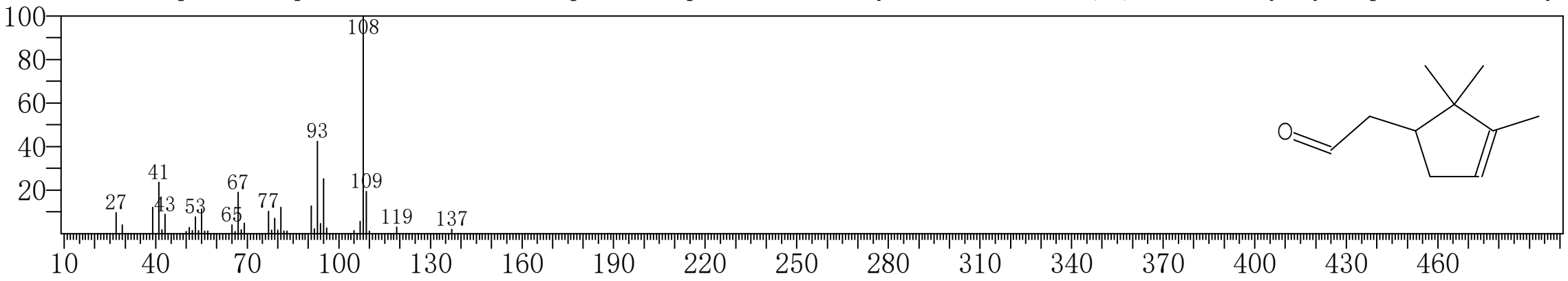

命中#:3　输入:17596　谱库:NIST17-1.lib
SI:94　分子式:C10H16O　CAS:4501-58-0　摩尔质量:152　保留指数:1155
组分名称:.alpha.-Campholenal $$ (R)-.alpha.-Campholene aldehyde $$ (R)-2-(2,2,3-Trimethylcyclopent-3-en-1-y

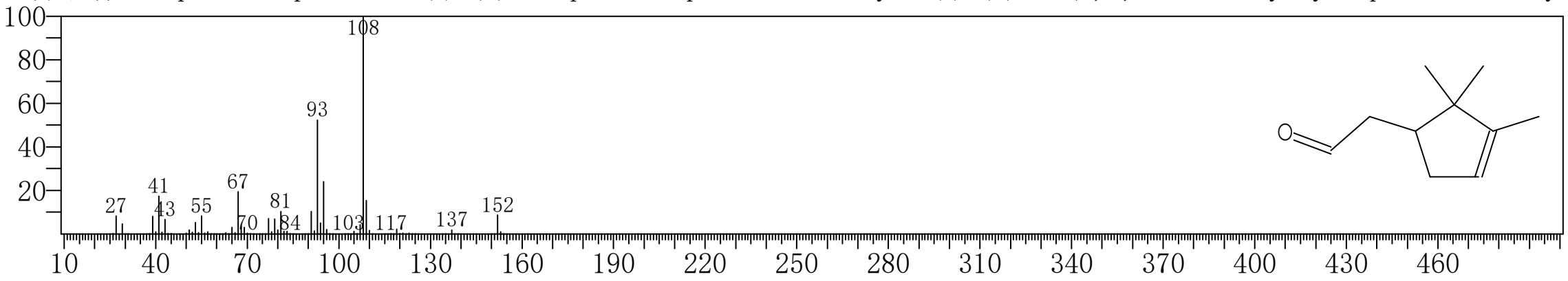

命中#:4　输入:11473　谱库:NIST17s.lib
SI:94　分子式:C10H16O　CAS:4501-58-0　摩尔质量:152　保留指数:1155
组分名称:.alpha.-Campholenal $$ (R)-.alpha.-Campholene aldehyde $$ (R)-2-(2,2,3-Trimethylcyclopent-3-en-1-y

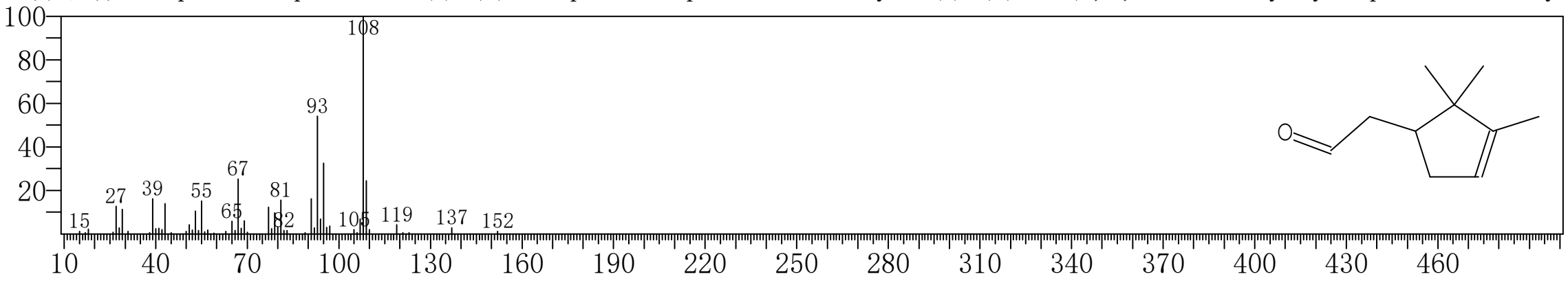

命中#:5　输入:17595　谱库:NIST17-1.lib
SI:93　分子式:C10H16O　CAS:0-00-0　摩尔质量:152　保留指数:1120
组分名称:1,7,7-Trimethylbicyclo[2.2.1]hept-5-en-2-ol

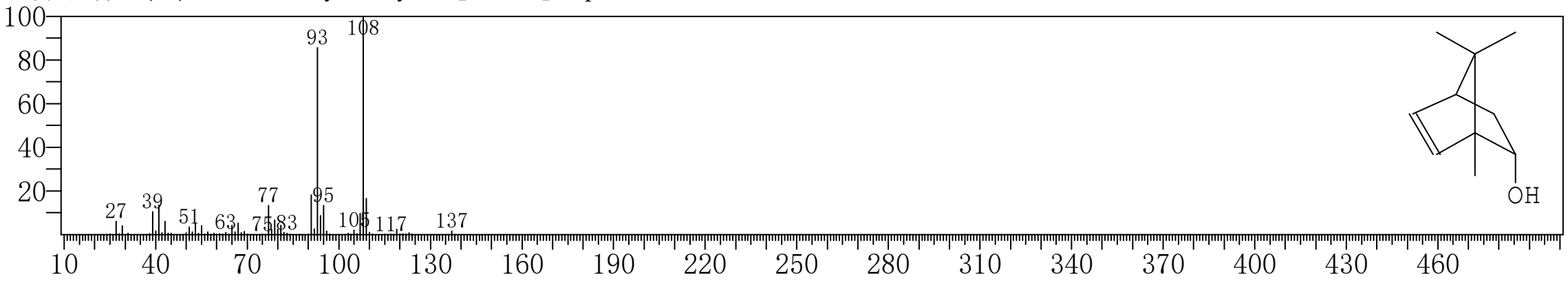

<< 目标组分 >>
行号#:18　保留时间:15.955(扫描数#:2192)　质量峰:247
原始模式:平均 15.950-15.960(2191-2193)　基峰:92.05(33308)
背景模式:峰校准　组 1 - 事件 1　Scan

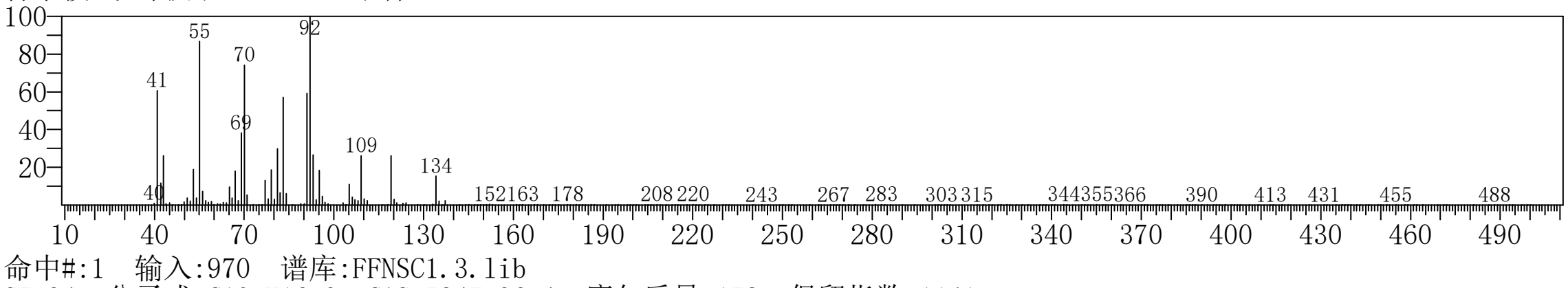

命中#:1　输入:970　谱库:FFNSC1.3.lib
SI:94　分子式:C10 H16 O　CAS:5947-36-4　摩尔质量:152　保留指数:1141
组分名称:Pinocarveol <trans->

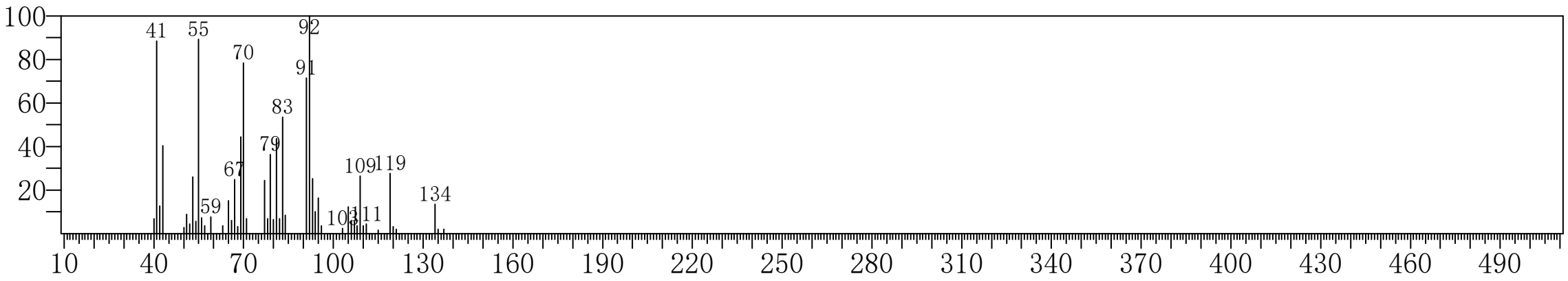

命中#:2　输入:11346　谱库:NIST17s.lib
SI:94　分子式:C10H16O　CAS:547-61-5　摩尔质量:152　保留指数:1131
组分名称:Bicyclo[3.1.1]heptan-3-ol, 6,6-dimethyl-2-methylene-, [1S-(1.alpha.,3.alpha.,5.alpha.)]- $$ (1S,3R,

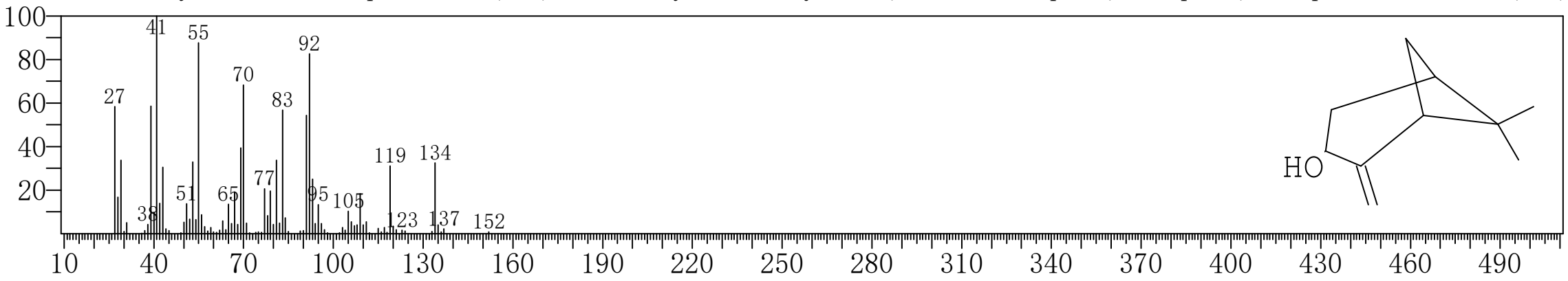

命中#:3　输入:17548　谱库:NIST17-1.lib
SI:94　分子式:C10H16O　CAS:547-61-5　摩尔质量:152　保留指数:1131
组分名称:Bicyclo[3.1.1]heptan-3-ol, 6,6-dimethyl-2-methylene-, [1S-(1.alpha.,3.alpha.,5.alpha.)]- $$ (1S,3R,

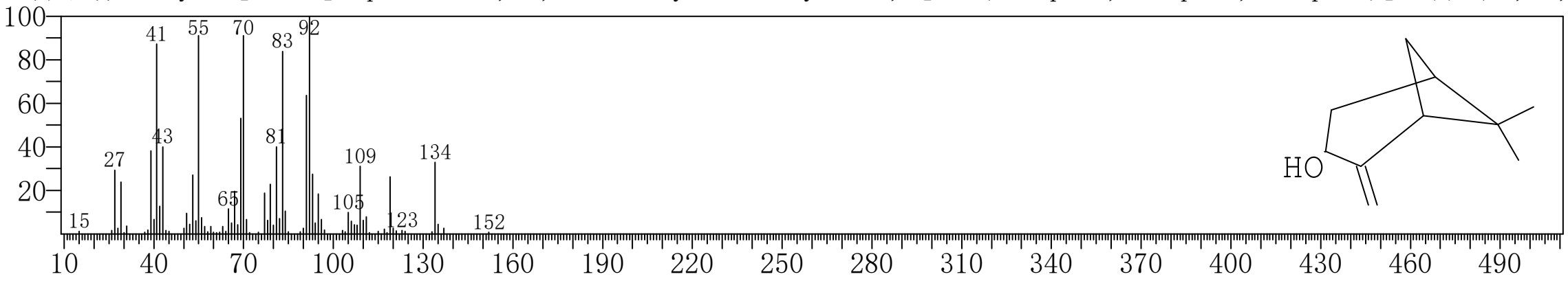

命中#:4　输入:17478　谱库:NIST17-1.lib
SI:94　分子式:C10H16O　CAS:5947-36-4　摩尔质量:152　保留指数:1131
组分名称:Bicyclo[3.1.1]heptan-3-ol, 6,6-dimethyl-2-methylene- $$ Pinocarveol $$ 2(10)-Pinen-3-ol $$ 6,6-Dim

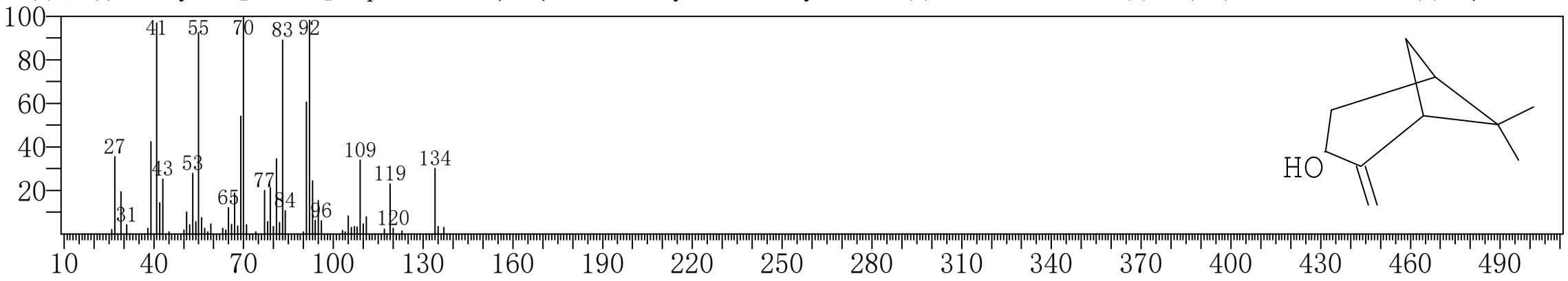

命中#:5　输入:11445　谱库:NIST17s.lib
SI:94　分子式:C10H16O　CAS:547-61-5　摩尔质量:152　保留指数:1131
组分名称:Bicyclo[3.1.1]heptan-3-ol, 6,6-dimethyl-2-methylene-, [1S-(1.alpha.,3.alpha.,5.alpha.)]- $$ (1S,3R,

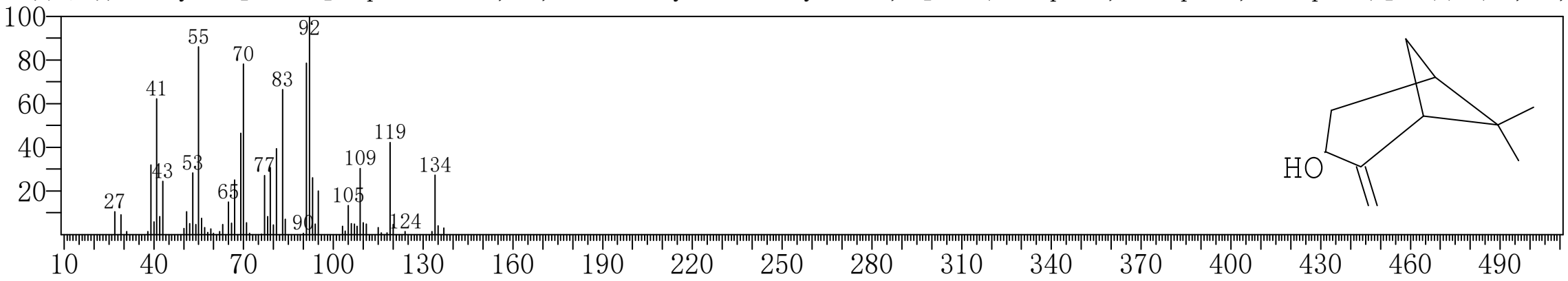

<< 目标组分 >>
行号#:19　保留时间:16.250(扫描数#:2251)　质量峰:250
原始模式:平均 16.245-16.255(2250-2252)　基峰:59.05(37505)
背景模式:峰校准　组 1 - 事件 1　Scan

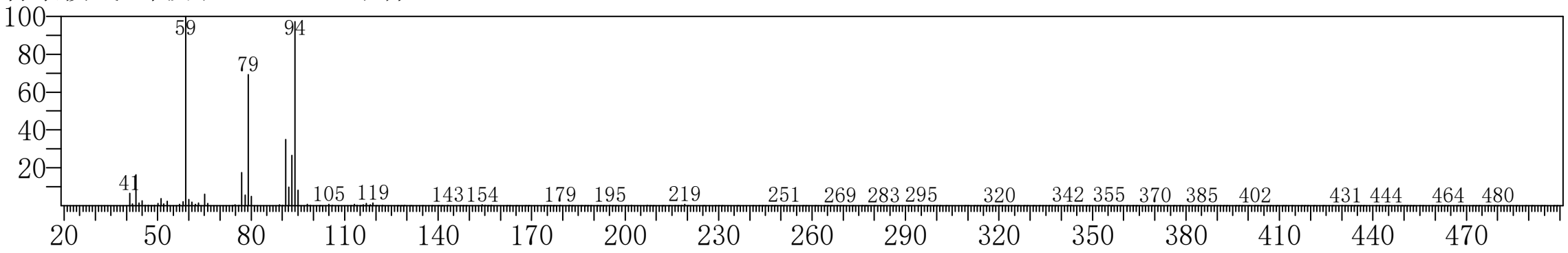

命中#:1　输入:17447　谱库:NIST17-1.lib
SI:92　分子式:C10H16O　CAS:1686-20-0　摩尔质量:152　保留指数:1125
组分名称:p-Mentha-1,5-dien-8-ol $$ 2,4-Cyclohexadiene-1-methanol, .alpha.,.alpha.,4-trimethyl- $$ .alpha.-Pl

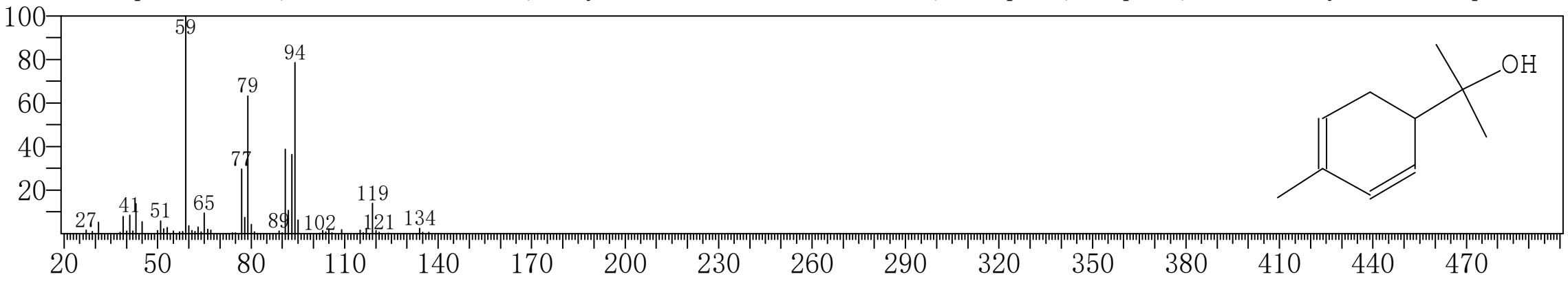

命中#:2　输入:11367　谱库:NIST17s.lib
SI:86　分子式:C10H16O　CAS:1686-20-0　摩尔质量:152　保留指数:1125
组分名称:p-Mentha-1,5-dien-8-ol $$ 2,4-Cyclohexadiene-1-methanol, .alpha.,.alpha.,4-trimethyl- $$ .alpha.-Pl

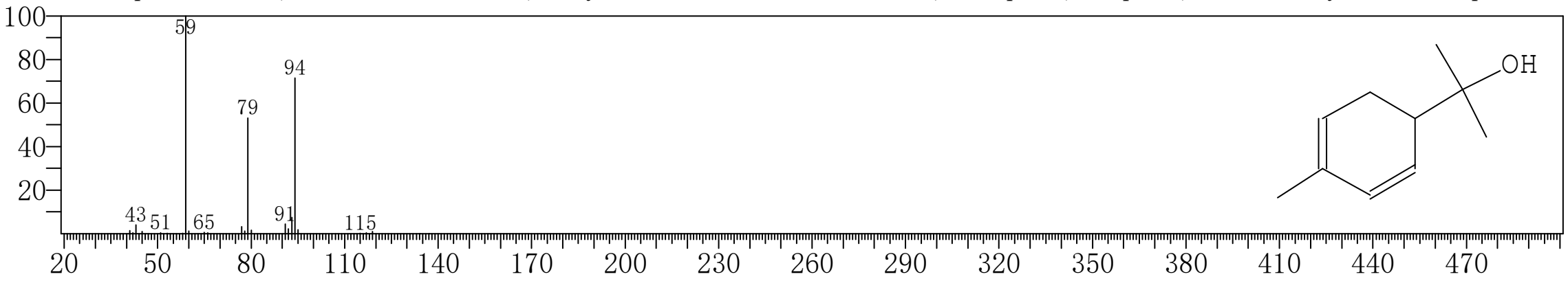

命中#:3　输入:59548　谱库:NIST17-1.lib
SI:84　分子式:C12H20O3　CAS:0-00-0　摩尔质量:212　保留指数:1471
组分名称:Cyclohexene, 3-acetoxy-4-(1-hydroxy-1-methylethyl)-1-methyl- $$ 6-(1-Hydroxy-1-methylethyl)-3-methy

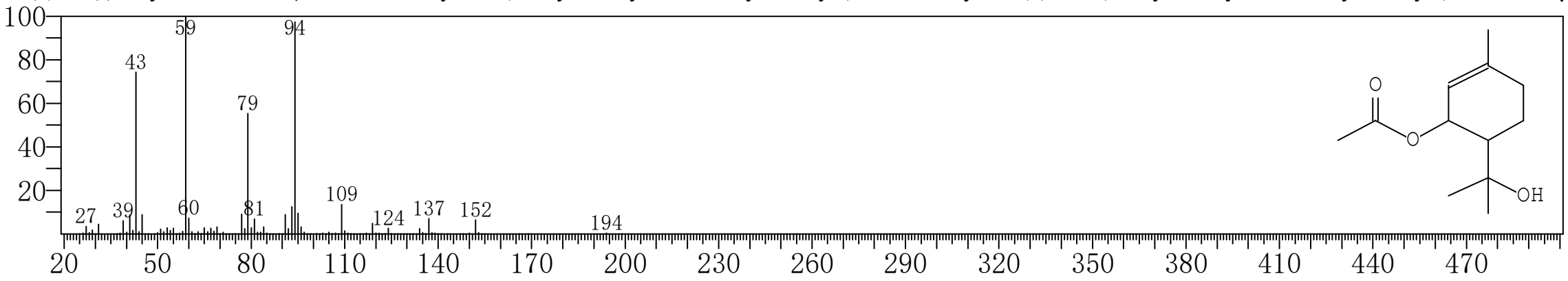

命中#:4　输入:446　谱库:FFNSC1.3.lib
SI:81　分子式:C9 H14　CAS:529-16-8　摩尔质量:122　保留指数:880
组分名称:Santene

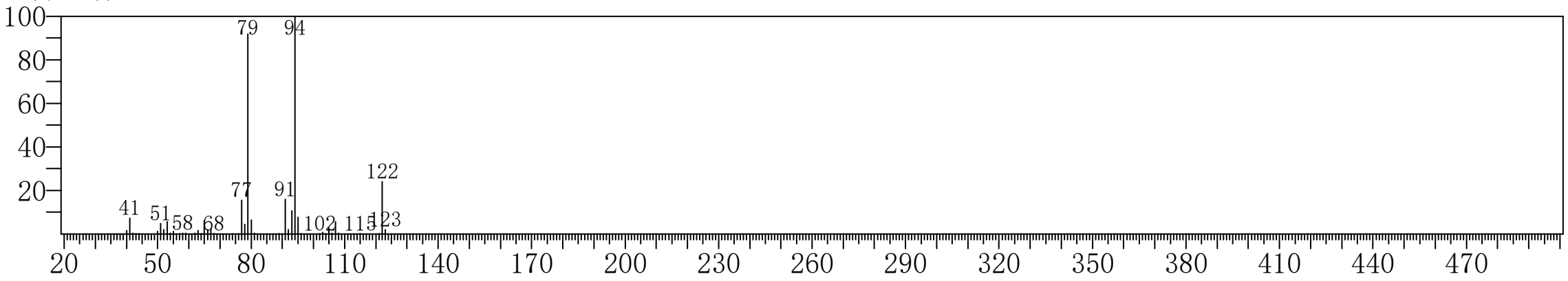

命中#:5　输入:5949　谱库:NIST17-1.lib
SI:80　分子式:C9H14　CAS:529-16-8　摩尔质量:122　保留指数:903
组分名称:Bicyclo[2.2.1]hept-2-ene, 2,3-dimethyl- $$ 2-Norbornene, 2,3-dimethyl- $$ Santen $$ Santene $$ 2,3-

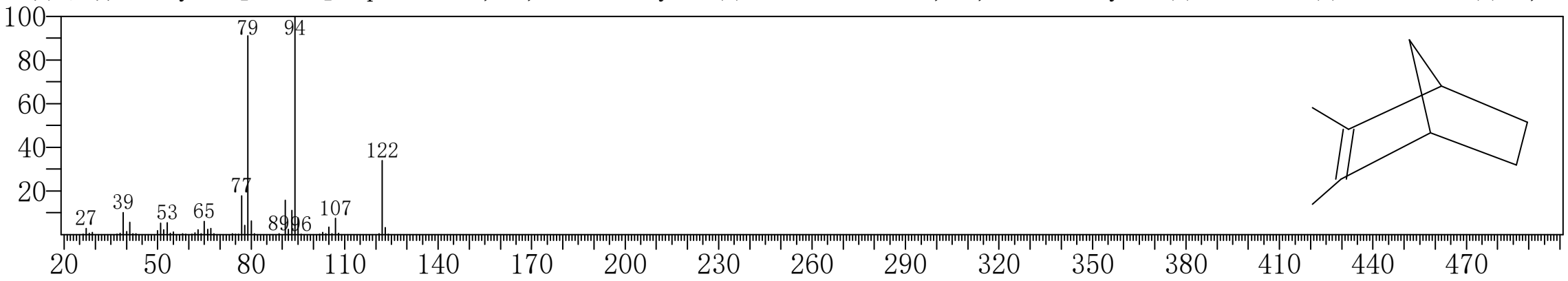

<< 目标组分 >>
行号#:20　保留时间:16.385(扫描数#:2278)　质量峰:259
原始模式:平均 16.380-16.390(2277-2279)　基峰:71.05(26360)
背景模式:峰校准　组 1 - 事件 1　Scan

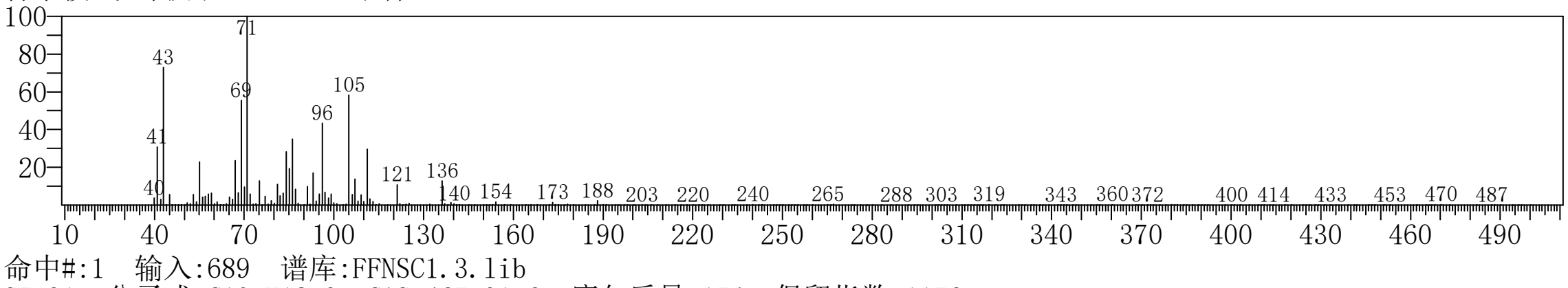

命中#:1　输入:689　谱库:FFNSC1.3.lib
SI:91　分子式:C10 H18 O　CAS:465-31-6　摩尔质量:154　保留指数:1156
组分名称:Camphene hydrate

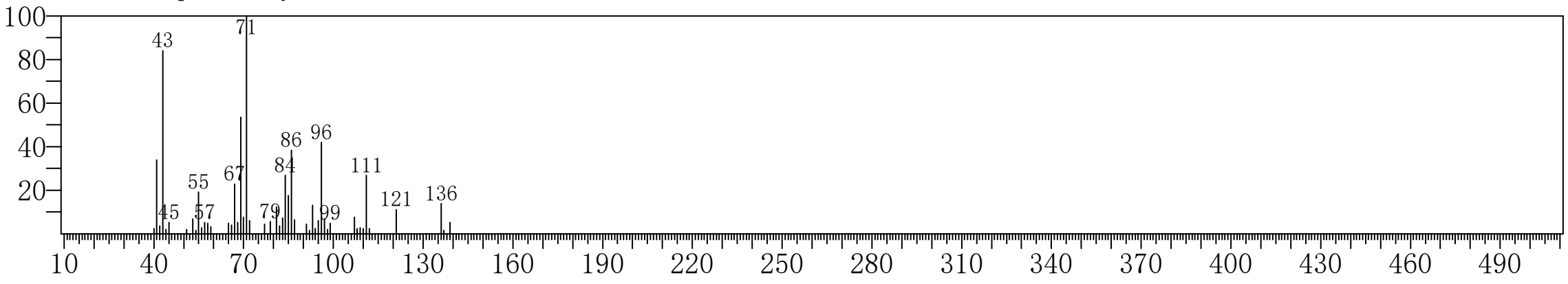

命中#:2　输入:18796　谱库:NIST17-1.lib
SI:88　分子式:C10H18O　CAS:465-31-6　摩尔质量:154　保留指数:1088
组分名称:Bicyclo[2.2.1]heptan-2-ol, 2,3,3-trimethyl- $$ Camphene hydrate $$ Camphenilanol, 3-methyl- $$ 3-Me

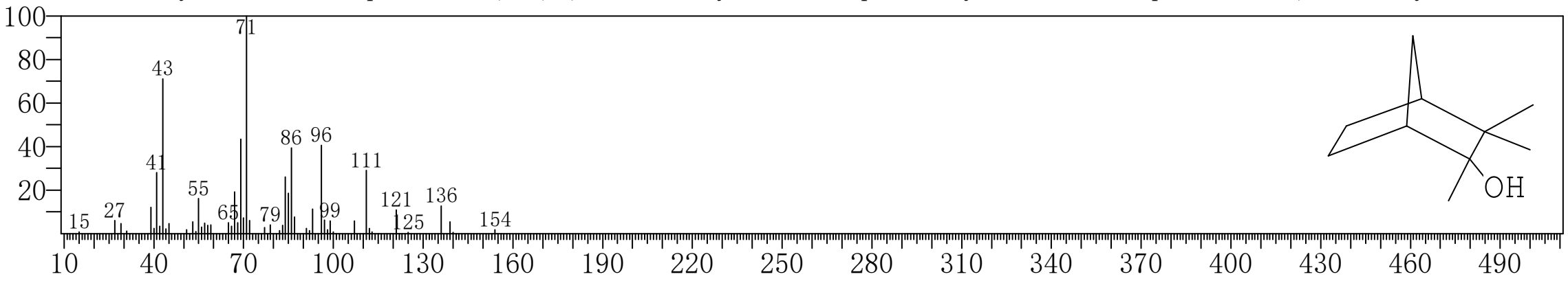

命中#:3　输入:11914　谱库:NIST17s.lib
SI:83　分子式:C10H18O　CAS:465-31-6　摩尔质量:154　保留指数:1088
组分名称:Bicyclo[2.2.1]heptan-2-ol, 2,3,3-trimethyl- $$ Camphene hydrate $$ Camphenilanol, 3-methyl- $$ 3-Me

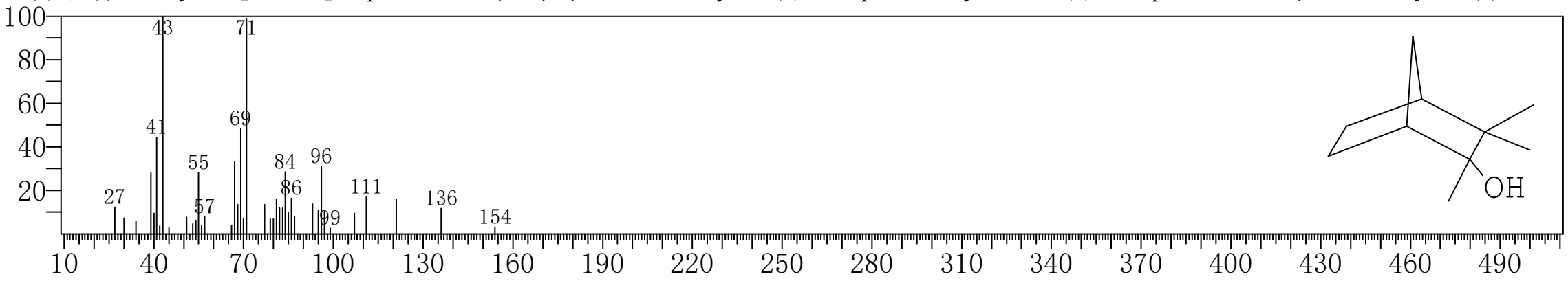

命中#:4　输入:36377　谱库:NIST17-1.lib
SI:80　分子式:C12H22O　CAS:72845-33-1　摩尔质量:182　保留指数:1181
组分名称:1,6-Octadiene, 3-ethoxy-3,7-dimethyl- $$ 3-Ethoxy-3,7-dimethyl-1,6-octadiene $$ Ethyl linalool $$

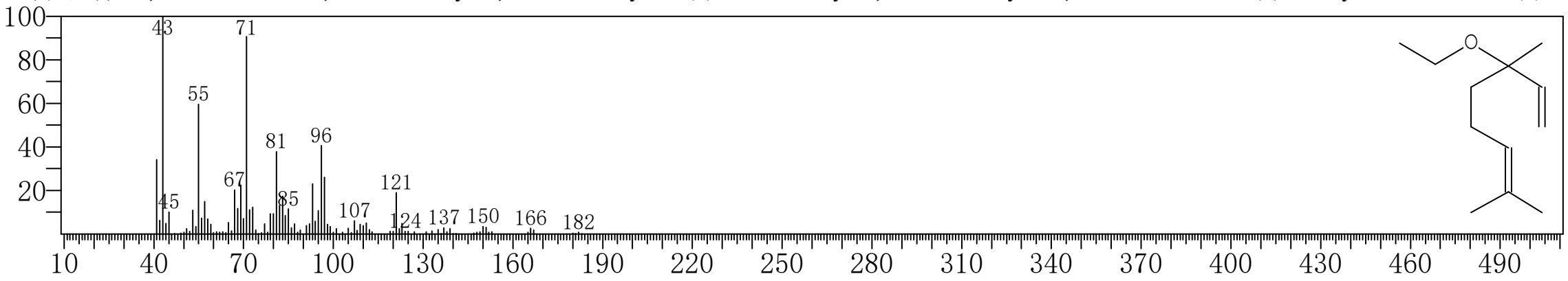

命中#:5　输入:18697　谱库:NIST17-1.lib
SI:78　分子式:C10H18O　CAS:4028-59-5　摩尔质量:154　保留指数:1099
组分名称:Cyclopentanol, 1,2-dimethyl-3-(1-methylethenyl)-, [1R-(1.alpha.,2.alpha.,3.beta.)]- $$ Cyclopentano

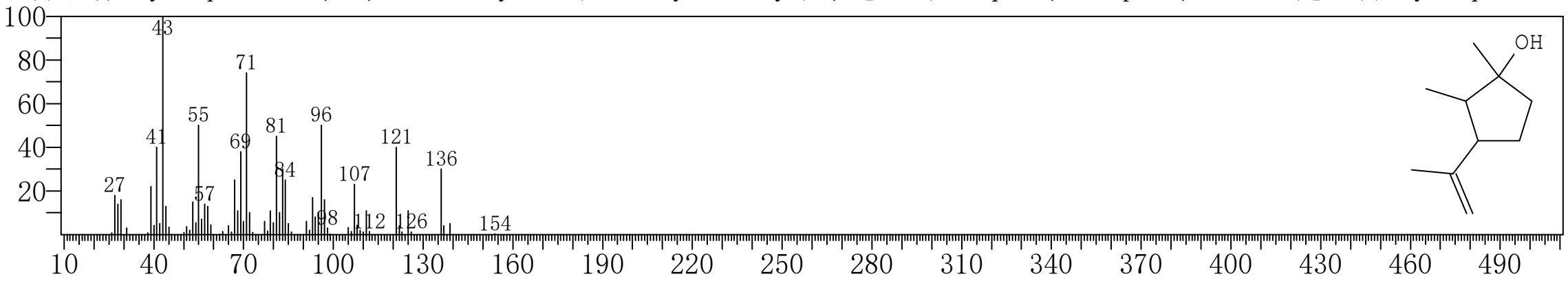

<< 目标组分 >>
行号#:21　保留时间:16.580(扫描数#:2317)　质量峰:249
原始模式:平均 16.575-16.585(2316-2318)　基峰:53.05(28985)
背景模式:峰校准　组 1 - 事件 1　Scan

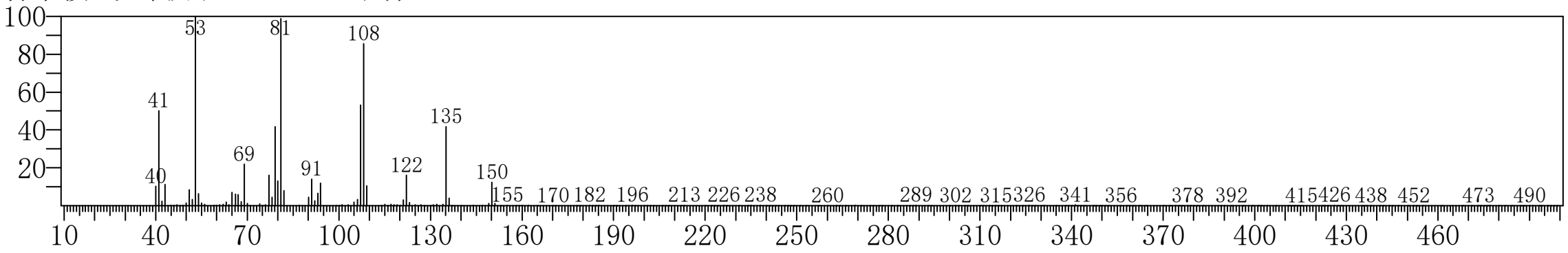

命中#:1　输入:1096　谱库:FFNSC1.3.lib
SI:95　分子式:C10 H14 O　CAS:30460-92-5　摩尔质量:150　保留指数:1164
组分名称:Pinocarvone

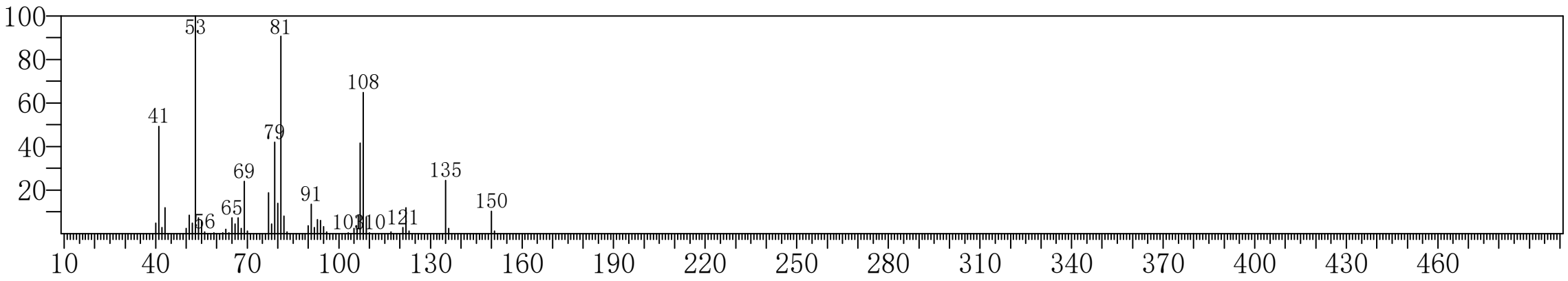

命中#:2　输入:10791　谱库:NIST17s.lib
SI:94　分子式:C10H14O　CAS:30460-92-5　摩尔质量:150　保留指数:1114
组分名称:Pinocarvone $$ 2(10)-Pinen-3-one $$ 6,6-Dimethyl-2-methylenebicyclo[3.1.1]heptan-3-one $$ .alpha.-l

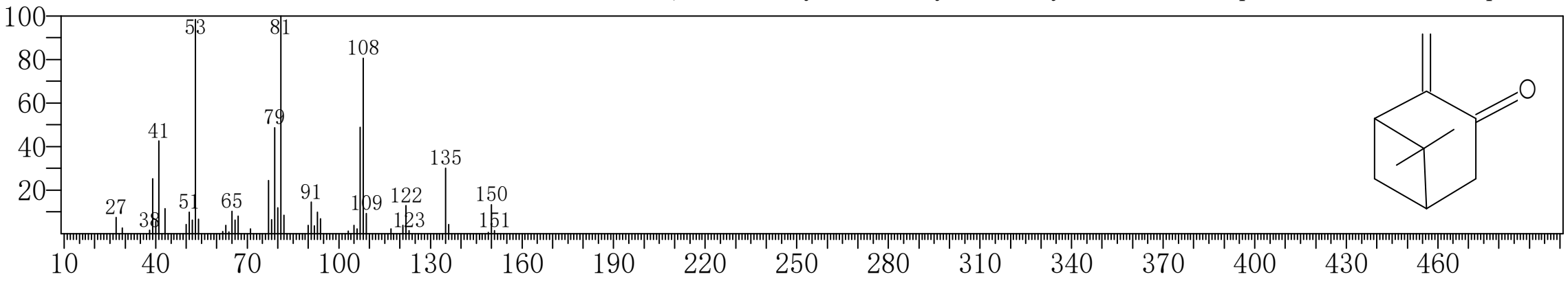

命中#:3　输入:10790　谱库:NIST17s.lib
SI:91　分子式:C10H14O　CAS:30460-92-5　摩尔质量:150　保留指数:1114
组分名称:Pinocarvone $$ 2(10)-Pinen-3-one $$ 6,6-Dimethyl-2-methylenebicyclo[3.1.1]heptan-3-one $$ .alpha.-l

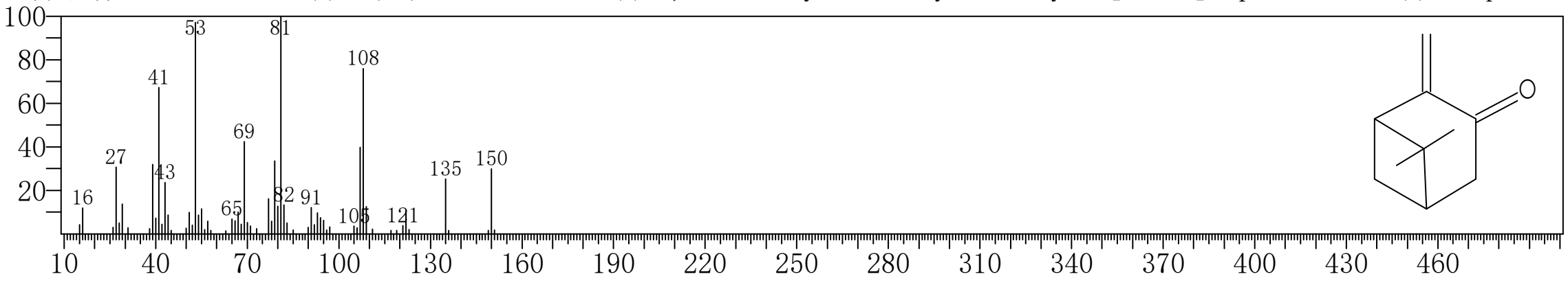

命中#:4　输入:16463　谱库:NIST17-1.lib
SI:90　分子式:C10H14O　CAS:30460-92-5　摩尔质量:150　保留指数:1114
组分名称:Pinocarvone $$ 2(10)-Pinen-3-one $$ 6,6-Dimethyl-2-methylenebicyclo[3.1.1]heptan-3-one $$ .alpha.-l

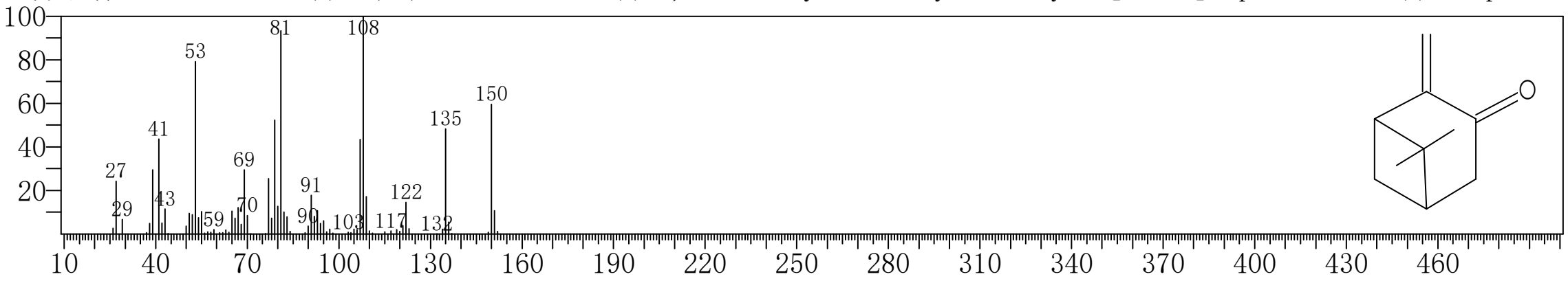

命中#:5　输入:10771　谱库:NIST17s.lib
SI:86　分子式:C10H14O　CAS:30460-92-5　摩尔质量:150　保留指数:1114
组分名称:Pinocarvone $$ 2(10)-Pinen-3-one $$ 6,6-Dimethyl-2-methylenebicyclo[3.1.1]heptan-3-one $$ .alpha.-l

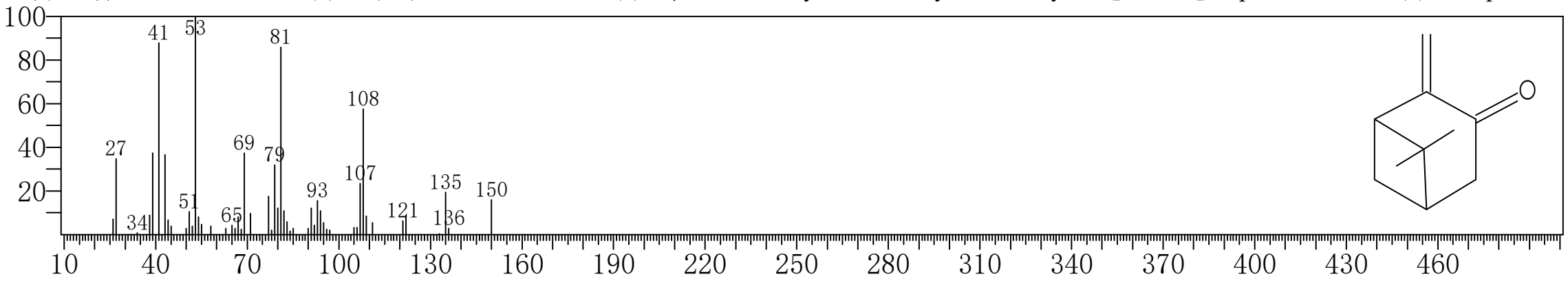

<< 目标组分 >>
行号#:22　保留时间:16.910(扫描数#:2383)　质量峰:263
原始模式:平均 16.905-16.915(2382-2384)　基峰:95.10(238540)
背景模式:峰校准　组 1 - 事件 1　Scan

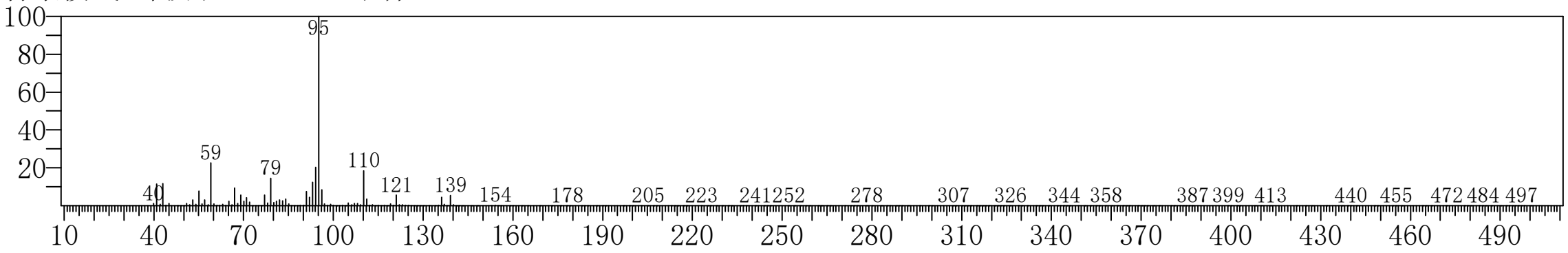

命中#:1　输入:12028　谱库:NIST17s.lib
SI:90　分子式:C10H18O　CAS:507-70-0　摩尔质量:154　保留指数:1138
组分名称:endo-Borneol $$ endo-2-Hydroxy-1,7,7-trimethylnorbornane $$ Bicyclo[2.2.1]heptan-2-ol, 1,7,7-trime

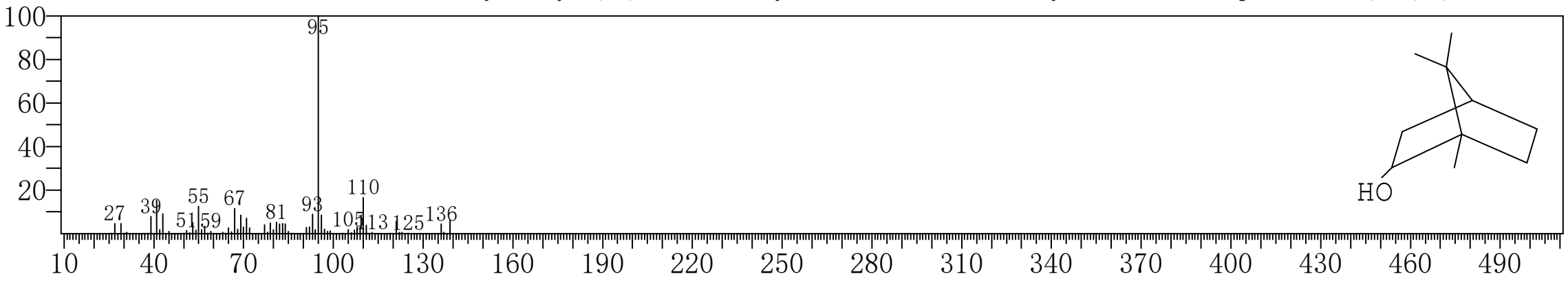

命中#:2　输入:1262　谱库:FFNSC1.3.lib
SI:90　分子式:C10 H18 O　CAS:507-70-0　摩尔质量:154　保留指数:1173
组分名称:Borneol

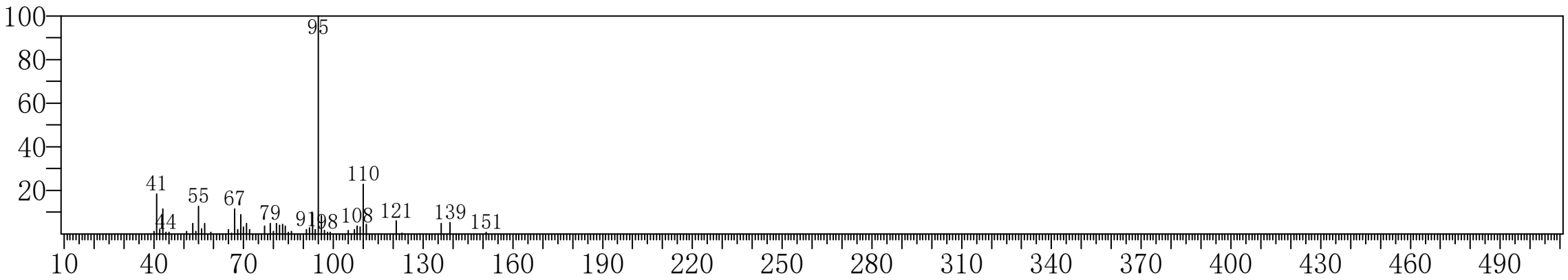

命中#:3　输入:12026　谱库:NIST17s.lib
SI:90　分子式:C10H18O　CAS:507-70-0　摩尔质量:154　保留指数:1138
组分名称:endo-Borneol $$ endo-2-Hydroxy-1,7,7-trimethylnorbornane $$ Bicyclo[2.2.1]heptan-2-ol, 1,7,7-trime

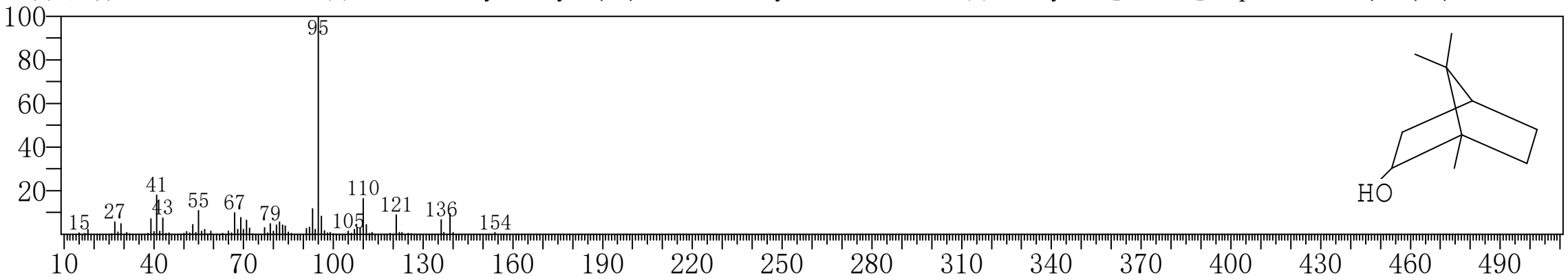

命中#:4　输入:18874　谱库:NIST17-1.lib
SI:90　分子式:C10H18O　CAS:464-45-9　摩尔质量:154　保留指数:1138
组分名称:Bicyclo[2.2.1]heptan-2-ol, 1,7,7-trimethyl-, (1S-endo)- $$ Borneol, (1S,2R,4S)-(-)- $$ (-)-Borneol

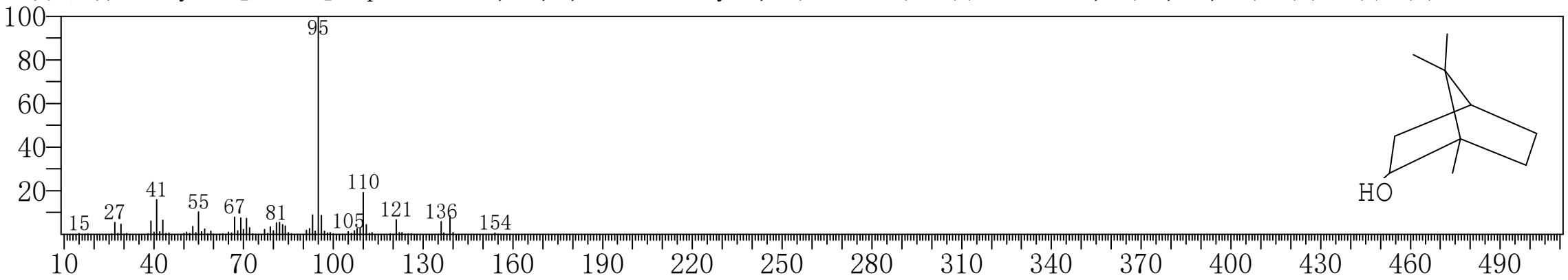

命中#:5　输入:12029　谱库:NIST17s.lib
SI:89　分子式:C10H18O　CAS:464-45-9　摩尔质量:154　保留指数:1138
组分名称:Bicyclo[2.2.1]heptan-2-ol, 1,7,7-trimethyl-, (1S-endo)- $$ Borneol, (1S,2R,4S)-(-)- $$ (-)-Borneol

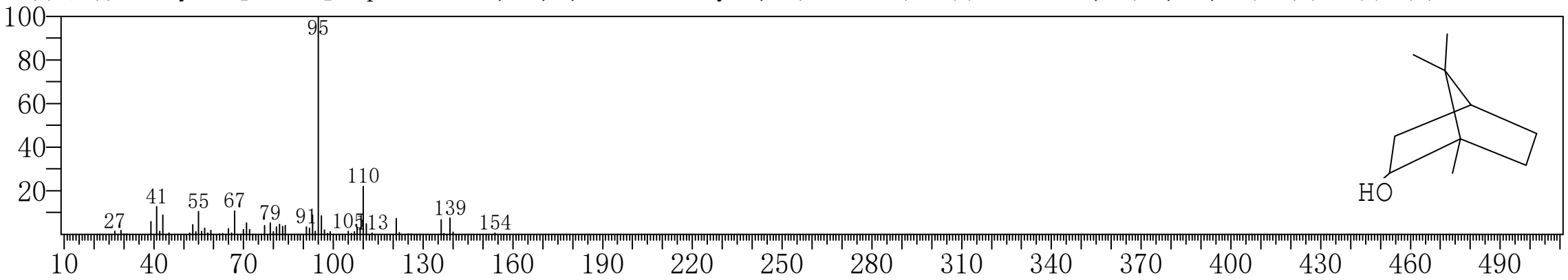

<< 目标组分 >>
行号#:23　 保留时间:17.155(扫描数#:2432)　质量峰:251
原始模式:平均 17.150-17.160(2431-2433)　　基峰:71.05(222772)
背景模式:峰校准　 组 1 - 事件 1　Scan

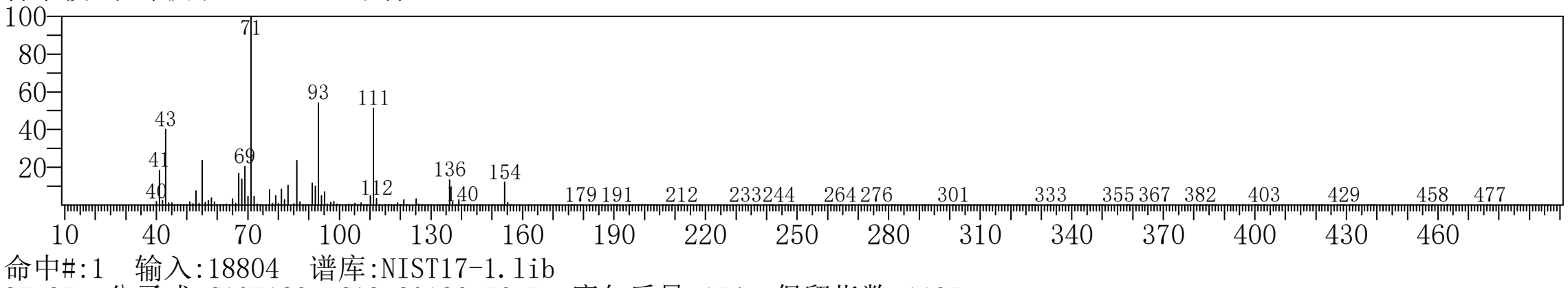

命中#:1　输入:18804　谱库:NIST17-1.lib
SI:97　分子式:C10H18O　CAS:20126-76-5　摩尔质量:154　保留指数:1137
组分名称:3-Cyclohexen-1-ol, 4-methyl-1-(1-methylethyl)-, (R)- $$ p-Menth-1-en-4-ol, (R)-(-)- $$ (-)-Terpinen

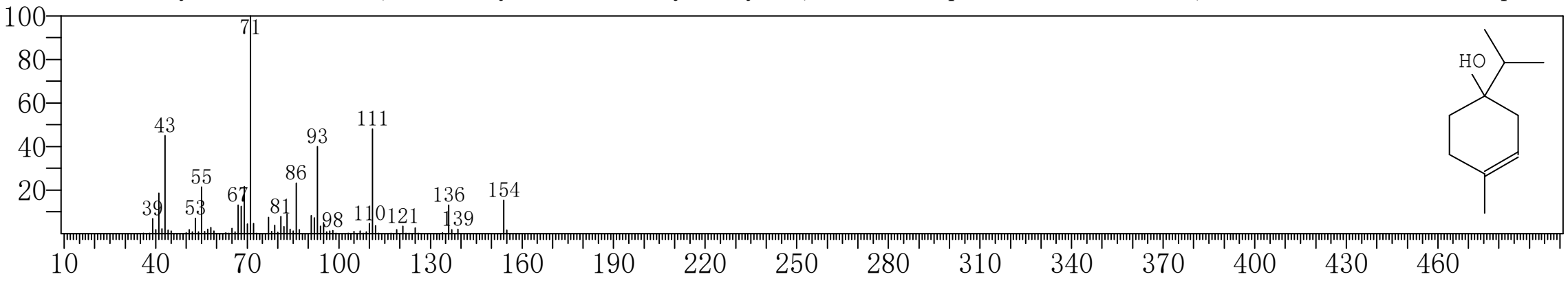

命中#:2　输入:11980　谱库:NIST17s.lib
SI:95　分子式:C10H18O　CAS:562-74-3　摩尔质量:154　保留指数:1137
组分名称:Terpinen-4-ol $$ 3-Cyclohexen-1-ol, 4-methyl-1-(1-methylethyl)- $$ p-Menth-1-en-4-ol $$ 1-Terpinen-

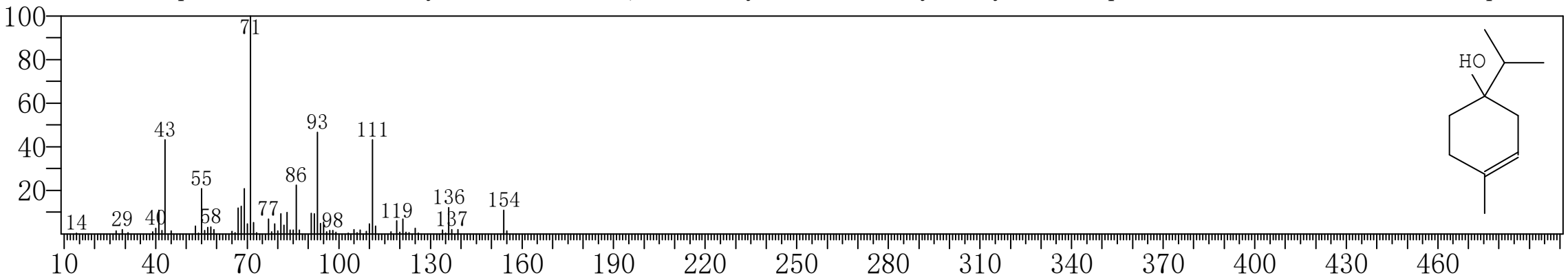

命中#:3　输入:18805　谱库:NIST17-1.lib
SI:95　分子式:C10H18O　CAS:562-74-3　摩尔质量:154　保留指数:1137
组分名称:Terpinen-4-ol $$ 3-Cyclohexen-1-ol, 4-methyl-1-(1-methylethyl)- $$ p-Menth-1-en-4-ol $$ 1-Terpinen-

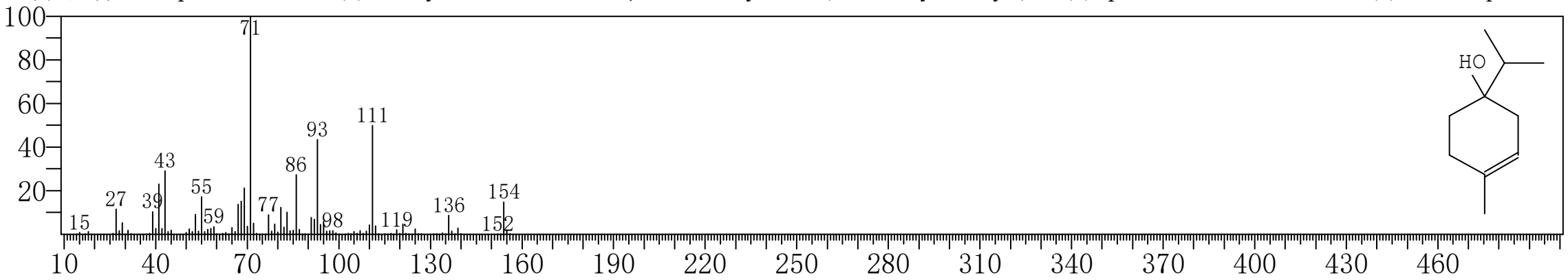

命中#:4　输入:11981　谱库:NIST17s.lib
SI:95　分子式:C10H18O　CAS:20126-76-5　摩尔质量:154　保留指数:1137
组分名称:3-Cyclohexen-1-ol, 4-methyl-1-(1-methylethyl)-, (R)- $$ p-Menth-1-en-4-ol, (R)-(-)- $$ (-)-Terpinen

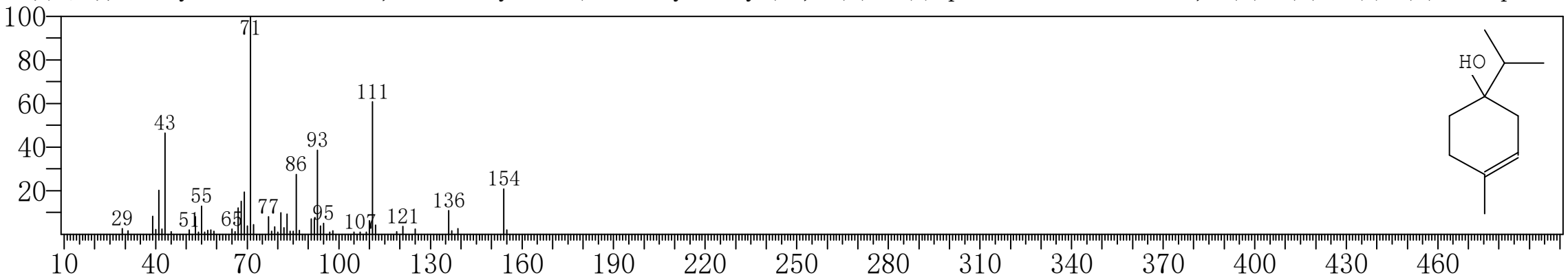

命中#:5　输入:11982　谱库:NIST17s.lib
SI:94　分子式:C10H18O　CAS:562-74-3　摩尔质量:154　保留指数:1137
组分名称:Terpinen-4-ol $$ 3-Cyclohexen-1-ol, 4-methyl-1-(1-methylethyl)- $$ p-Menth-1-en-4-ol $$ 1-Terpinen-

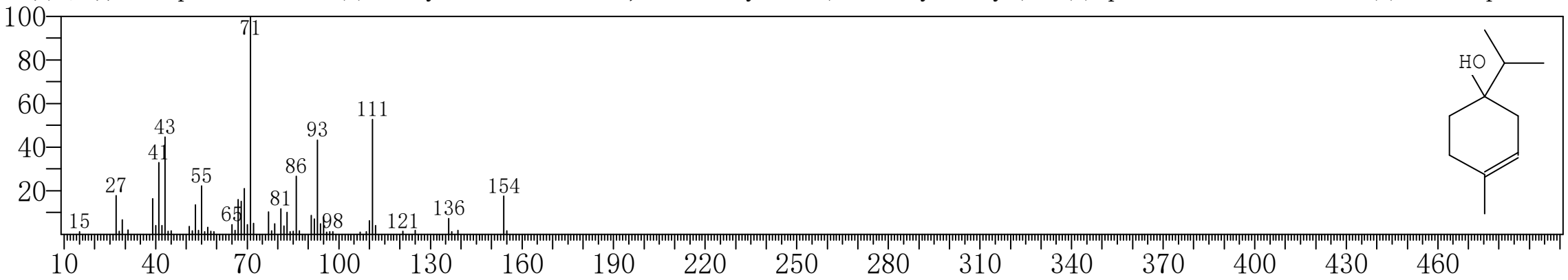

<< 目标组分 >>
行号#:24　保留时间:17.600(扫描数#:2521)　质量峰:291
原始模式:平均 17.595-17.605(2520-2522)　基峰:59.05(1165553)
背景模式:峰校准　组 1 - 事件 1　Scan

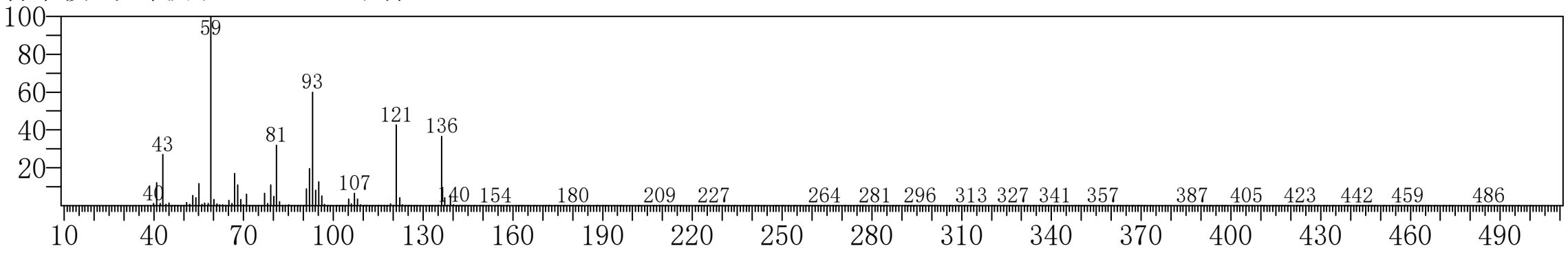

命中#:1　输入:11945　谱库:NIST17s.lib
SI:96　分子式:C10H18O　CAS:98-55-5　摩尔质量:154　保留指数:1143
组分名称:.alpha.-Terpineol $$ 3-Cyclohexene-1-methanol, .alpha.,.alpha.4-trimethyl- $$ p-Menth-1-en-8-ol $$

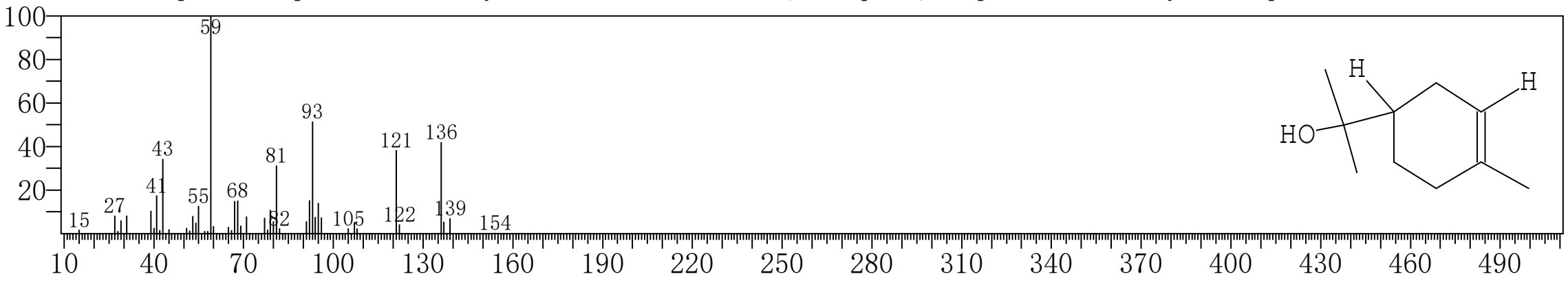

命中#:2　输入:18749　谱库:NIST17-1.lib
SI:96　分子式:C10H18O　CAS:10482-56-1　摩尔质量:154　保留指数:1143
组分名称:L-.alpha.-Terpineol $$ 3-Cyclohexene-1-methanol, .alpha.,.alpha.,4-trimethyl-, (S)- $$ p-Menth-1-en

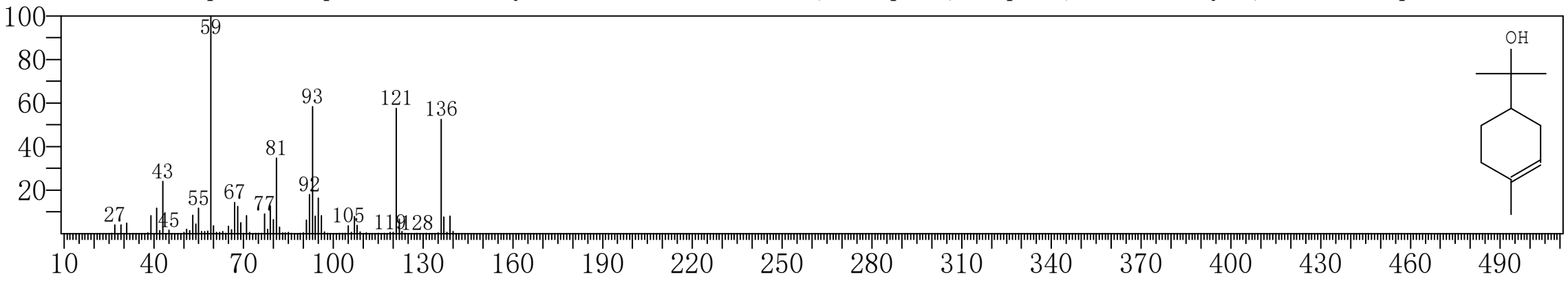

命中#:3　输入:18748　谱库:NIST17-1.lib
SI:95　分子式:C10H18O　CAS:7785-53-7　摩尔质量:154　保留指数:1143
组分名称:3-Cyclohexene-1-methanol, .alpha.,.alpha.,4-trimethyl-, (R)-

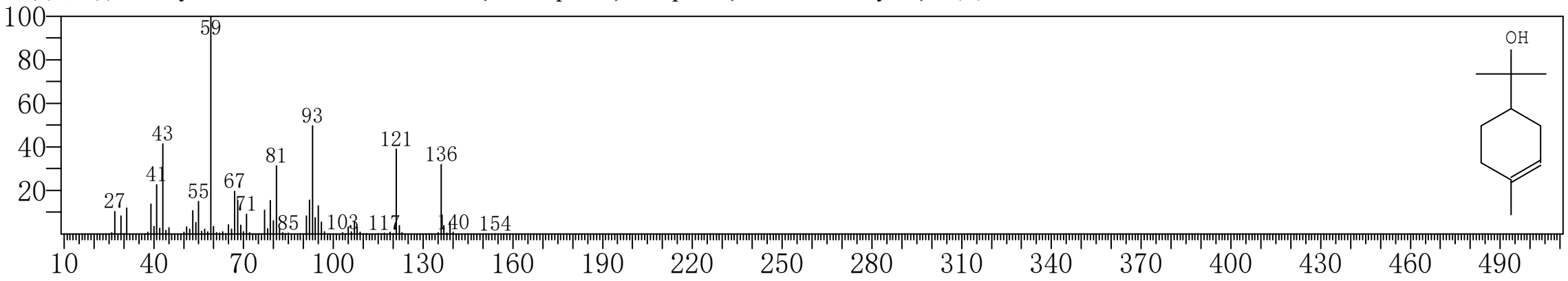

命中#:4　输入:11942　谱库:NIST17s.lib
SI:94　分子式:C10H18O　CAS:10482-56-1　摩尔质量:154　保留指数:1143
组分名称:L-.alpha.-Terpineol $$ 3-Cyclohexene-1-methanol, .alpha.,.alpha.,4-trimethyl-, (S)- $$ p-Menth-1-en

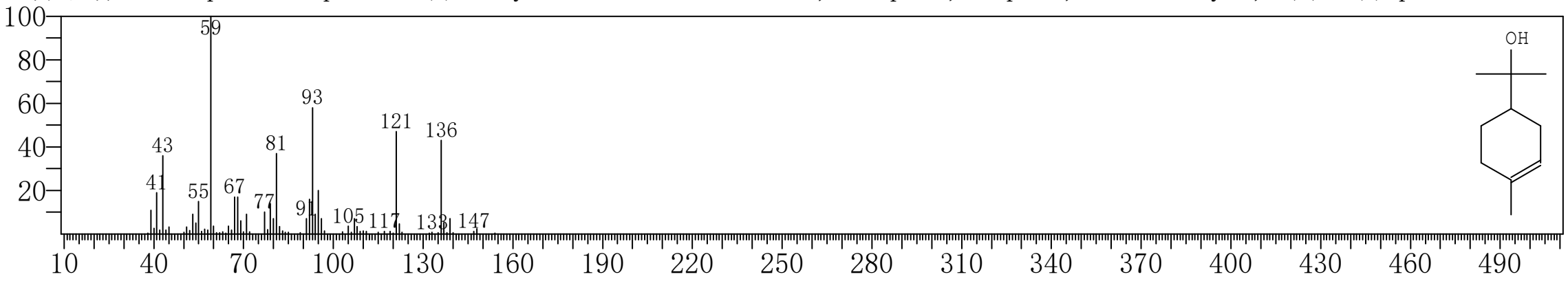

命中#:5　输入:497　谱库:FFNSC1.3.lib
SI:94　分子式:C10 H18 O　CAS:98-55-5　摩尔质量:154　保留指数:1195
组分名称:Terpineol <alpha->

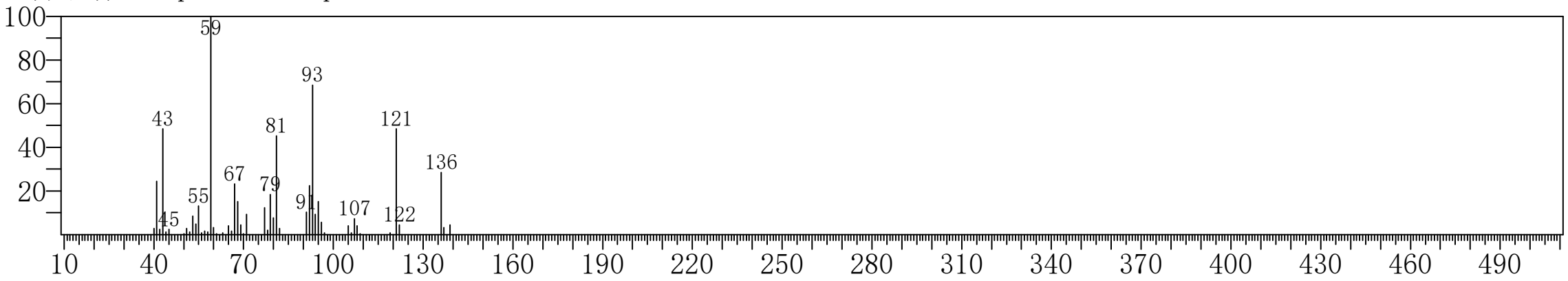

<< 目标组分 >>
行号#:25   保留时间:18.270(扫描数#:2655) 质量峰:301
原始模式:平均 18.265-18.275(2654-2656)   基峰:94.10(53228)
背景模式:峰校准 组 1 - 事件 1 Scan

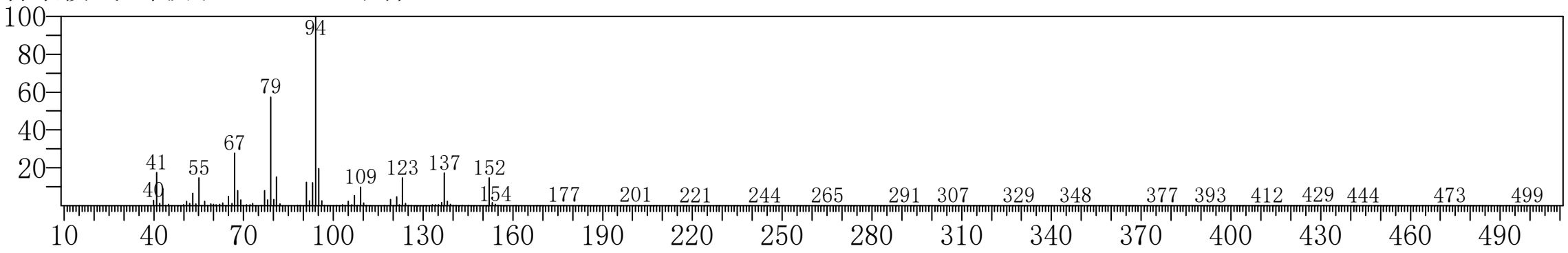

命中#:1 输入:17560 谱库:NIST17-1.lib
SI:88 分子式:C10H16O CAS:29548-14-9 摩尔质量:152 保留指数:1175
组分名称:3-Cyclohexene-1-acetaldehyde, .alpha.,4-dimethyl- $$ .alpha.,4-Dimethyl-3-cyclohexene-1-acetaldehyd

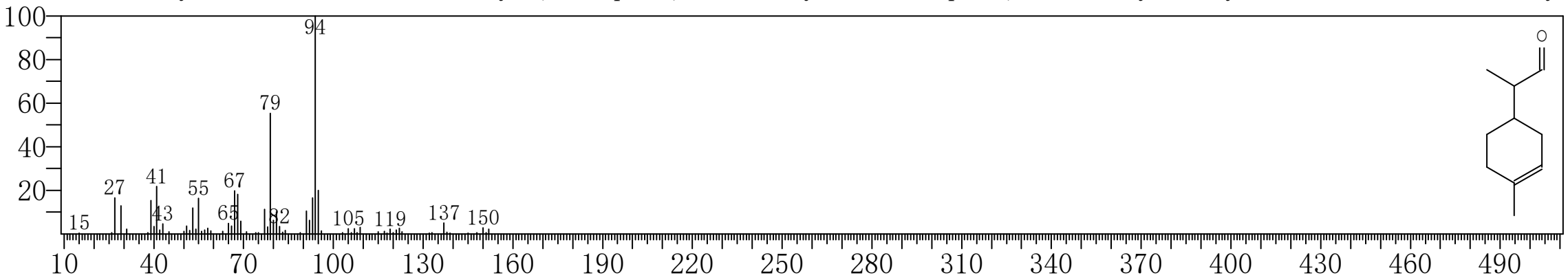

命中#:2 输入:11449 谱库:NIST17s.lib
SI:88 分子式:C10H16O CAS:29548-14-9 摩尔质量:152 保留指数:1175
组分名称:3-Cyclohexene-1-acetaldehyde, .alpha.,4-dimethyl- $$ .alpha.,4-Dimethyl-3-cyclohexene-1-acetaldehyd

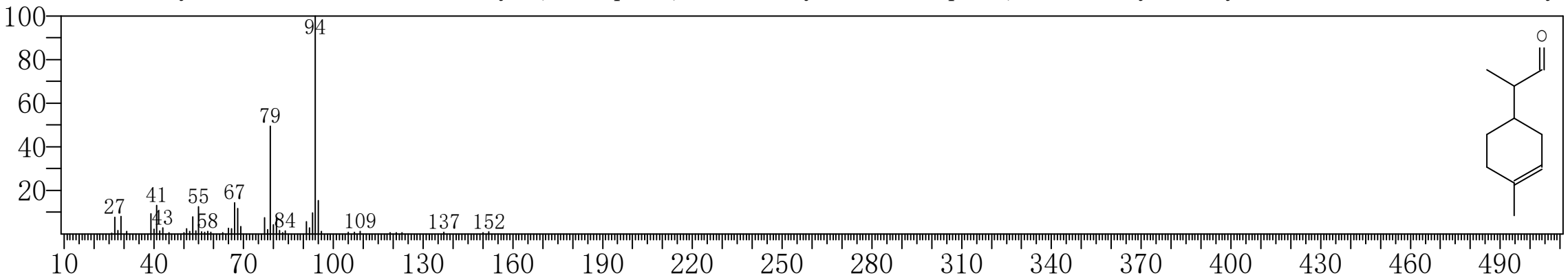

命中#:3 输入:169505 谱库:NIST17-1.lib
SI:85 分子式:C17H26O4S CAS:0-00-0 摩尔质量:326 保留指数:2410
组分名称:Camphor-10-sulfonic acid, (3-methylenecyclopentyl)methyl ester $$ (3-Methylenecyclopentyl)methyl (

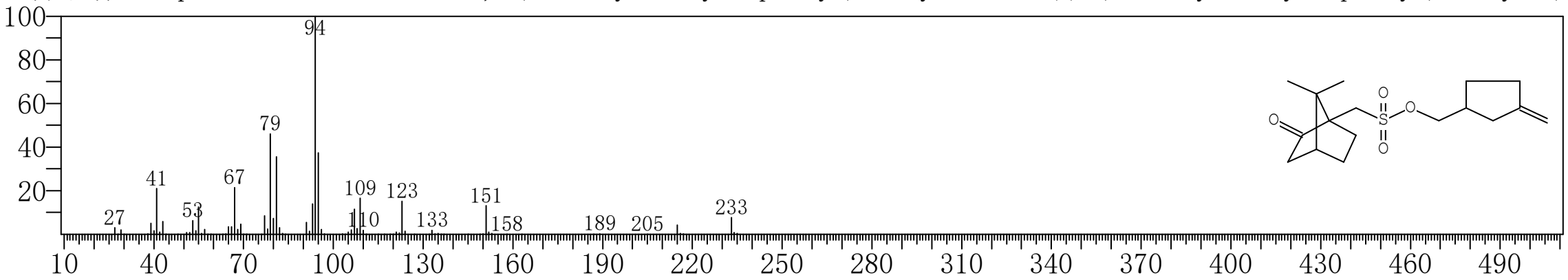

命中#:4 输入:1176 谱库:FFNSC1.3.lib
SI:83 分子式:C14 H24 O CAS:65405-84-7 摩尔质量:208 保留指数:1479
组分名称:Cetonal

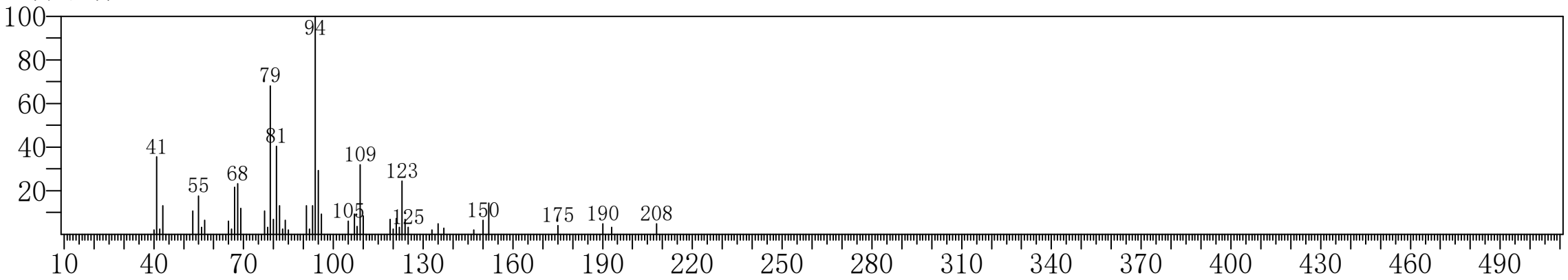

命中#:5 输入:11060 谱库:NIST17-1.lib
SI:82 分子式:C9H14O CAS:129993-40-4 摩尔质量:138 保留指数:1140
组分名称:Cyclohexene, 1-methyl-3-(formylmethyl)- $$ (3-Methyl-2-cyclohexen-1-yl)acetaldehyde # $$

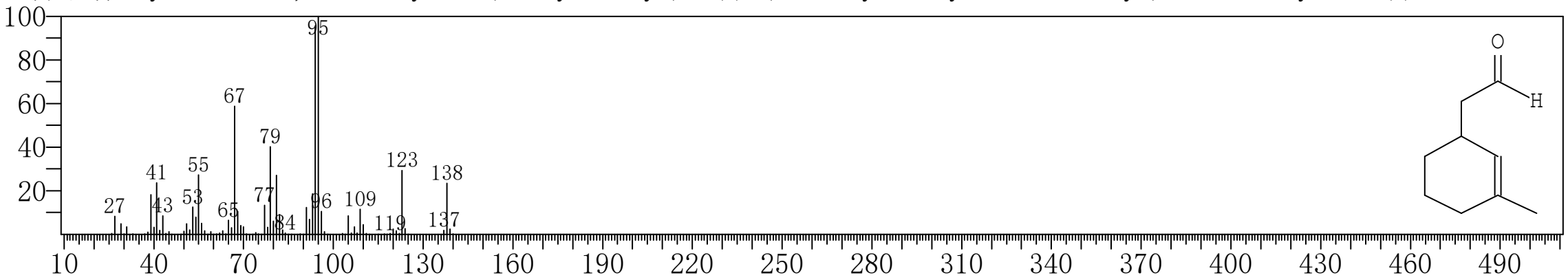

<< 目标组分 >>
行号#:26　保留时间:20.070(扫描数#:3015)　质量峰:265
原始模式:平均 20.065-20.075(3014-3016)　基峰:165.10(51121)
背景模式:峰校准　组 1 - 事件 1　Scan

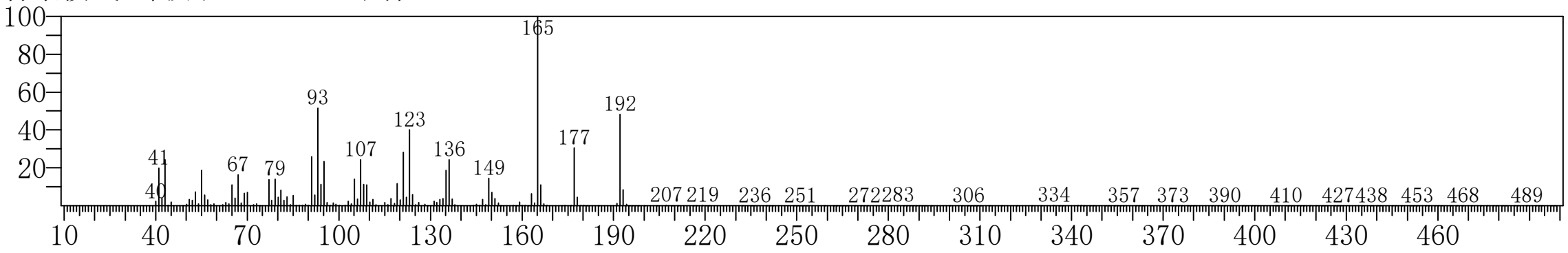

命中#:1　输入:43409　谱库:NIST17-1.lib
SI:76　分子式:C13H20O　CAS:0-00-0　摩尔质量:192　保留指数:1461
组分名称:1H-2-Indenone,2,4,5,6,7,7a-hexahydro-3-(1-methylethyl)-7a-methyl $$ 3-Isopropyl-7a-methyl-1,4,5,6,7

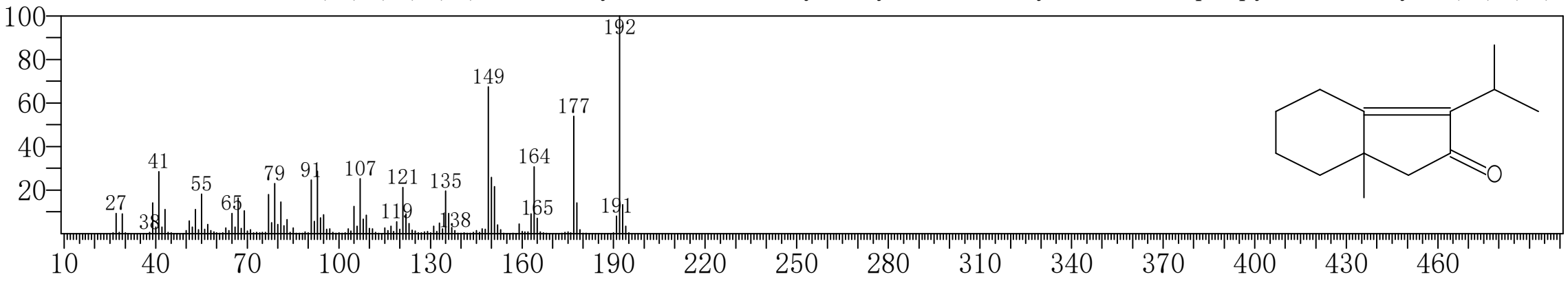

命中#:2　输入:43444　谱库:NIST17-1.lib
SI:74　分子式:C14H24　CAS:193695-14-6　摩尔质量:192　保留指数:1315
组分名称:(1R,2R,5R,E)-7-Ethylidene-1,2,8,8-tetramethylbicyclo[3.2.1]octane $$ Bicyclo[3.2.1]octane, 7-ethyl

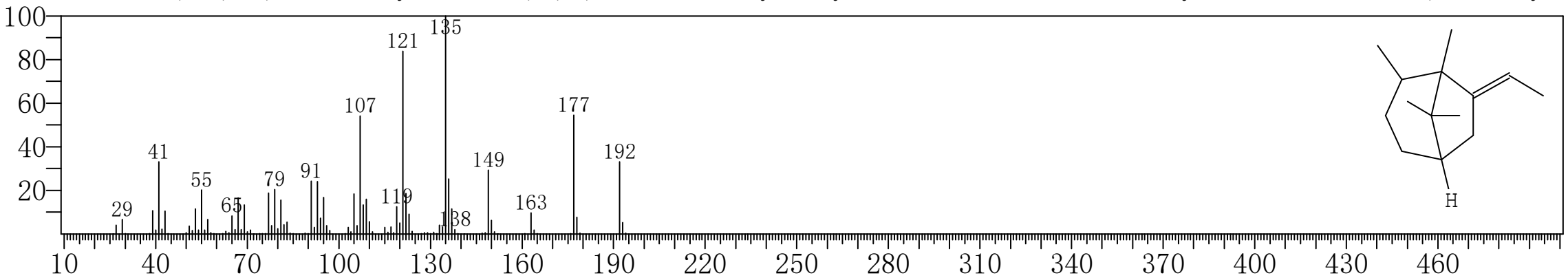

命中#:3　输入:43402　谱库:NIST17-1.lib
SI:74　分子式:C13H20O　CAS:35044-68-9　摩尔质量:192　保留指数:1457
组分名称:2-Buten-1-one, 1-(2,6,6-trimethyl-1-cyclohexen-1-yl)- $$ 1-(2,6,6-Trimethyl-1-cyclohexen-1-yl)-2-bu

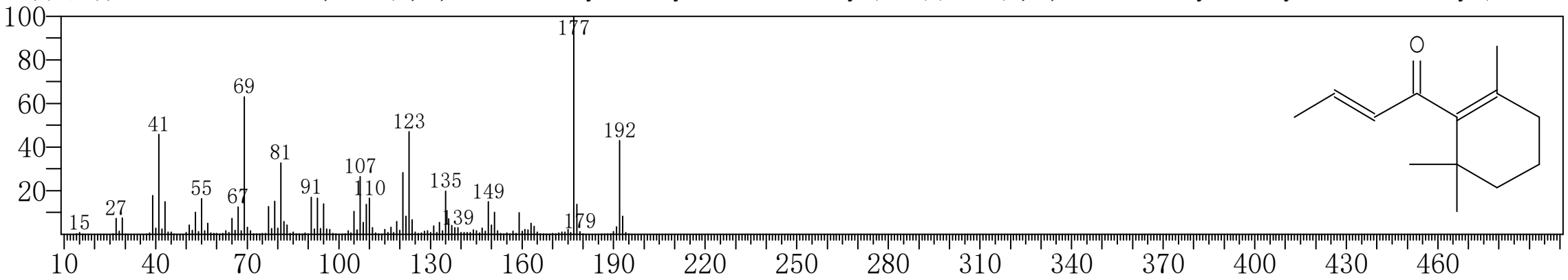

命中#:4　输入:43318　谱库:NIST17-1.lib
SI:74　分子式:C13H20O　CAS:55123-72-3　摩尔质量:192　保留指数:1491
组分名称:2(3H)-Naphthalenone, 4,4a,5,6,7,8-hexahydro-4a,7,7-trimethyl-, (R)- $$ 4a,7,7-Trimethyl-4,4a,5,6,7,

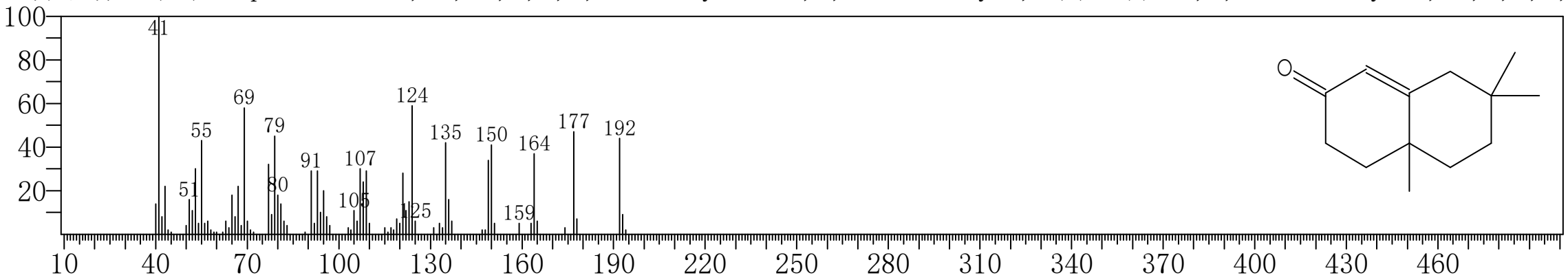

命中#:5　输入:33445　谱库:NIST17-1.lib
SI:73　分子式:C12H18O　CAS:0-00-0　摩尔质量:178　保留指数:1446
组分名称:4,4A,5,6,7,8-Hexahydro-1,4A-dimethyl-2(3H)-naphthalenone

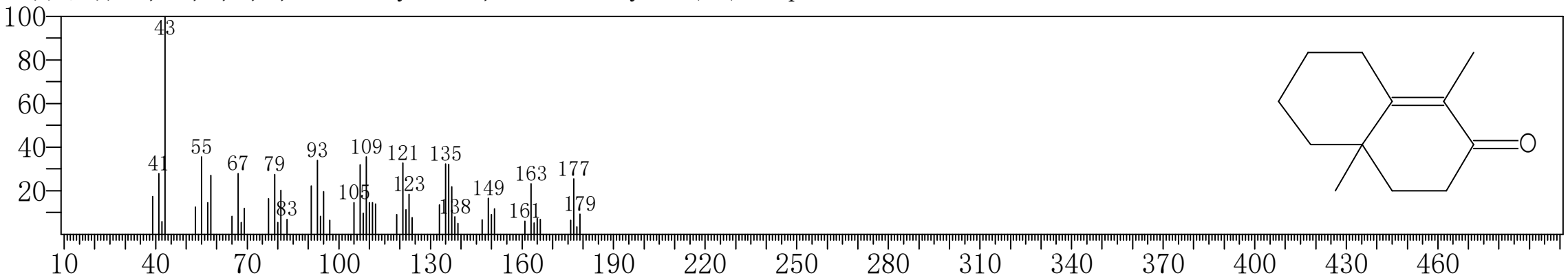

<< 目标组分 >>
行号#:27　保留时间:20.560(扫描数#:3113)　质量峰:289
原始模式:平均 20.555-20.565(3112-3114)　基峰:179.15(287312)
背景模式:峰校准　组 1 - 事件 1　Scan

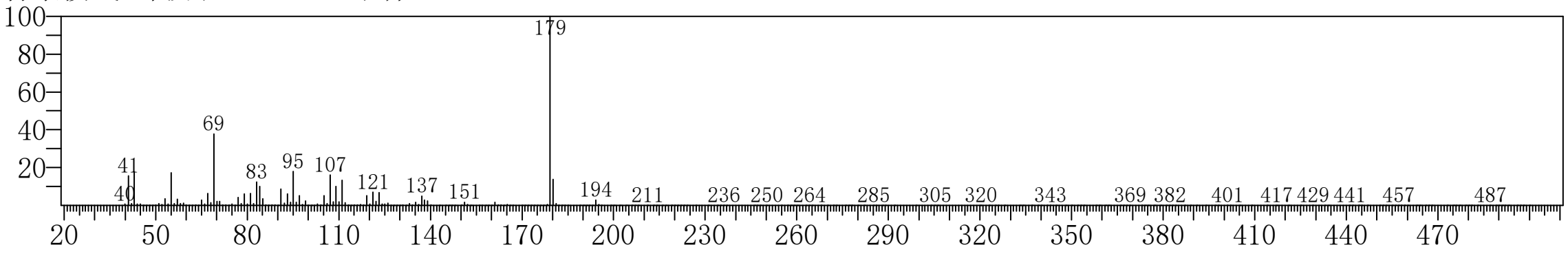

命中#:1　输入:20394　谱库:NIST17s.lib
SI:91　分子式:C13H22O　CAS:41678-32-4　摩尔质量:194　保留指数:1342
组分名称:2H-1-Benzopyran, 3,4,4a,5,6,8a-hexahydro-2,5,5,8a-tetramethyl-, (2.alpha.,4a.alpha.,8a.alpha.)- $$

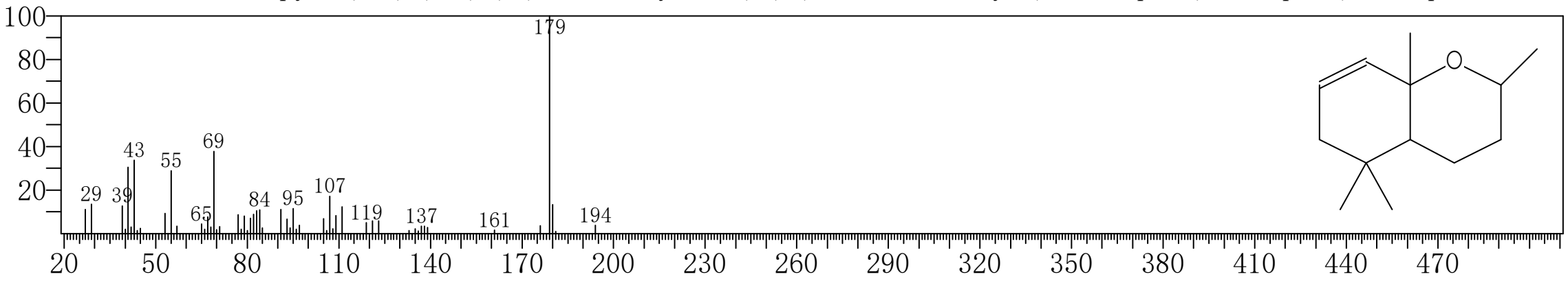

命中#:2　输入:45142　谱库:NIST17-1.lib
SI:88　分子式:C13H22O　CAS:41678-32-4　摩尔质量:194　保留指数:1342
组分名称:2H-1-Benzopyran, 3,4,4a,5,6,8a-hexahydro-2,5,5,8a-tetramethyl-, (2.alpha.,4a.alpha.,8a.alpha.)- $$

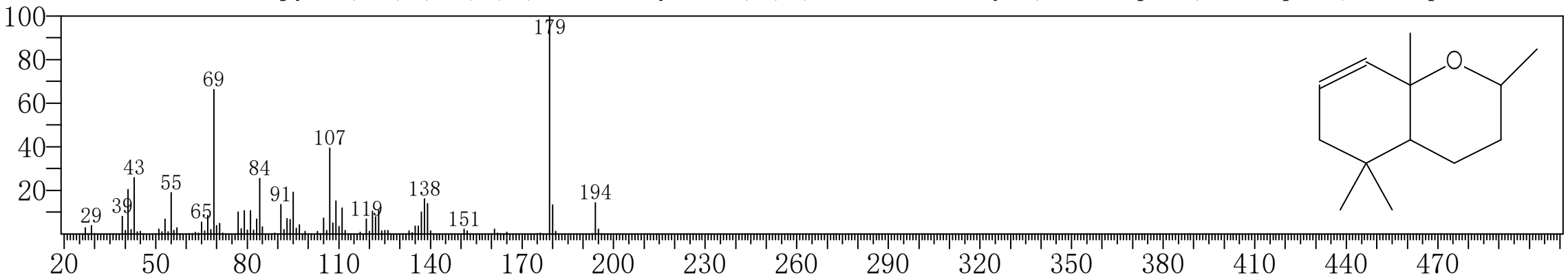

命中#:3　输入:45141　谱库:NIST17-1.lib
SI:83　分子式:C13H22O　CAS:72746-44-2　摩尔质量:194　保留指数:1342
组分名称:2,5,5,8a-Tetramethyl-3,4,4a,5,6,8a-hexahydro-2H-chromene $$ Dihydroedulan $$ 3.2H-1-Benzopyran, 3,4

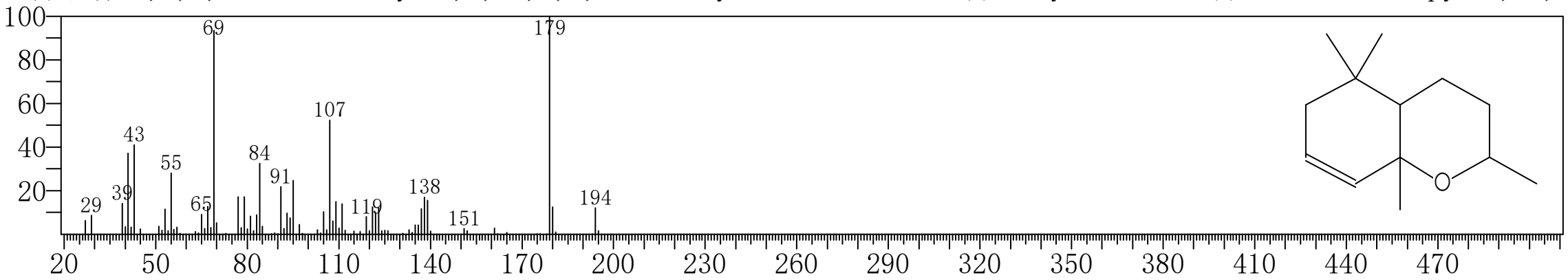

命中#:4　输入:68262　谱库:NIST17-1.lib
SI:79　分子式:C13H18O3　CAS:80114-25-6　摩尔质量:222　保留指数:1497
组分名称:Ethanone, 1-(1a,2,3,5,6a,6b-hexahydro-3,3,6a-trimethyloxireno[g]benzofuran-5-yl)- $$ 1-(3,3,6a-Tri

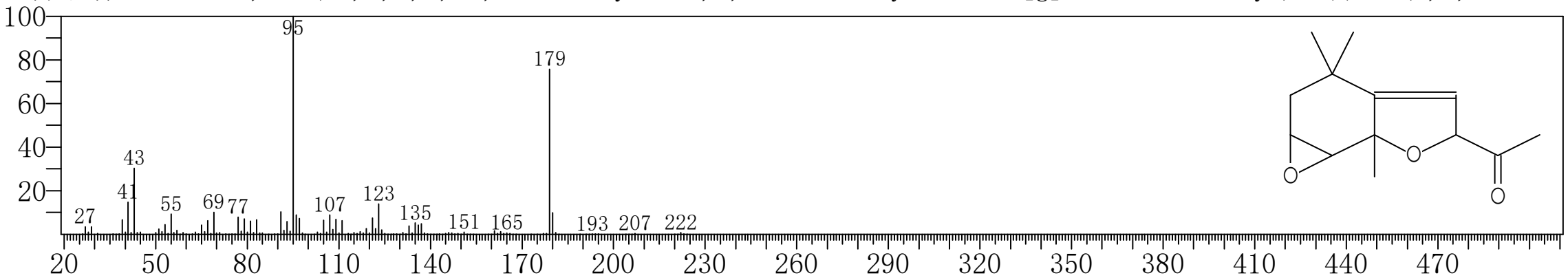

命中#:5　输入:68495　谱库:NIST17-1.lib
SI:76　分子式:C14H22O2　CAS:65868-86-2　摩尔质量:222　保留指数:1565
组分名称:2-Heptanone, 6-(3-acetyl-2-methyl-1-cyclopropen-1-yl)-6-methyl- $$ 6-(3-Acetyl-2-methyl-1-cyclopro

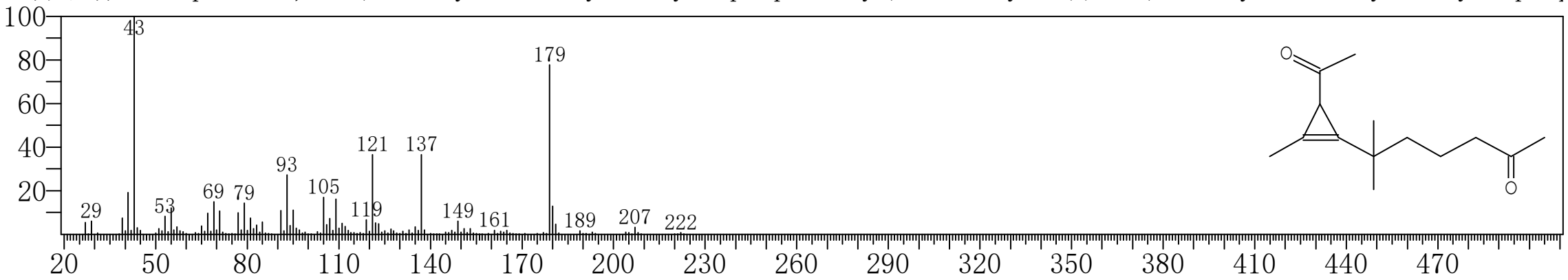

<< 目标组分 >>
行号#:28　保留时间:22.635(扫描数#:3528)　质量峰:262
原始模式:平均 22.630-22.640(3527-3529)　基峰:105.10(76427)
背景模式:峰校准　组 1 - 事件 1　Scan

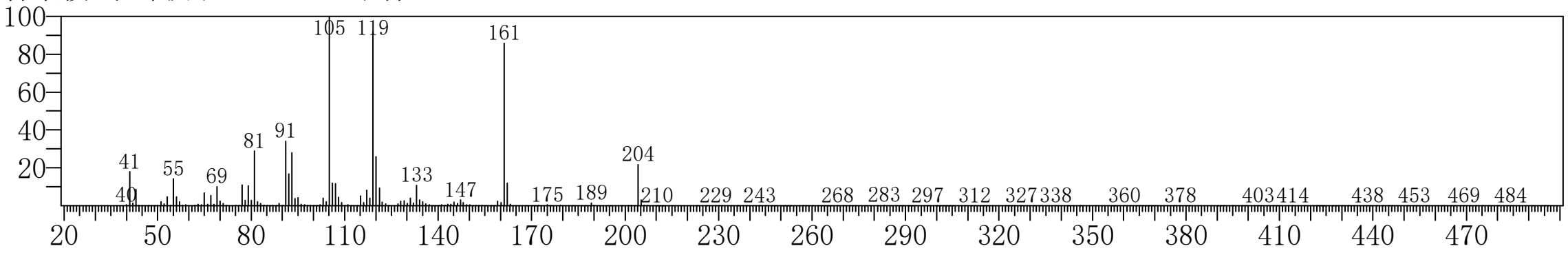

命中#:1　输入:1095　谱库:FFNSC1.3.lib
SI:97　分子式:C15 H24　CAS:31141-66-9　摩尔质量:204　保留指数:1349
组分名称:Cubebene <alpha->

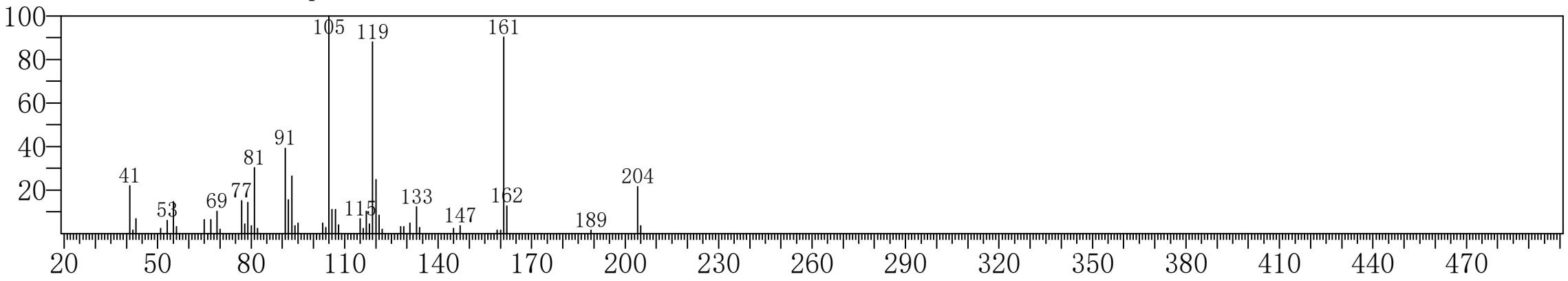

命中#:2　输入:22349　谱库:NIST17s.lib
SI:96　分子式:C15H24　CAS:17699-14-8　摩尔质量:204　保留指数:1344
组分名称:.alpha.-Cubebene $$ 1H-Cyclopenta[1,3]cyclopropa[1,2]benzene, 3a,3b,4,5,6,7-hexahydro-3,7-dimethyl-

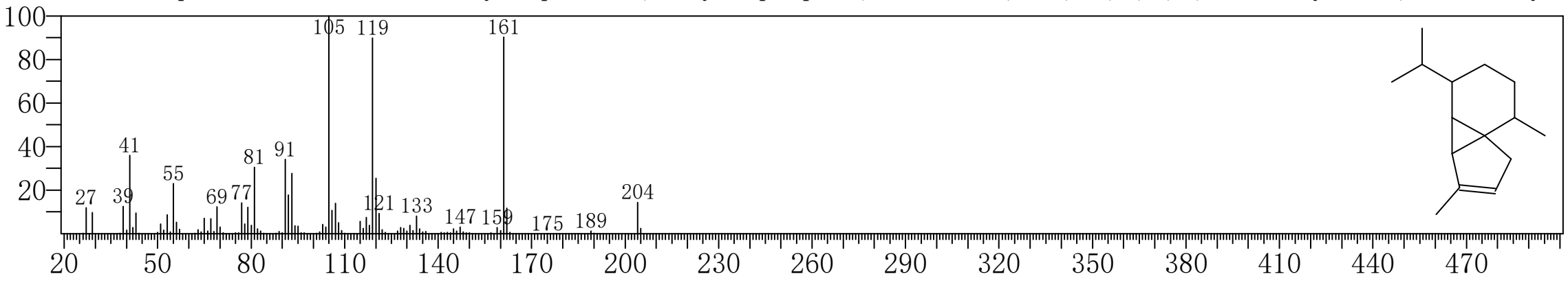

命中#:3　输入:52923　谱库:NIST17-1.lib
SI:95　分子式:C15H24　CAS:17699-14-8　摩尔质量:204　保留指数:1344
组分名称:.alpha.-Cubebene $$ 1H-Cyclopenta[1,3]cyclopropa[1,2]benzene, 3a,3b,4,5,6,7-hexahydro-3,7-dimethyl-

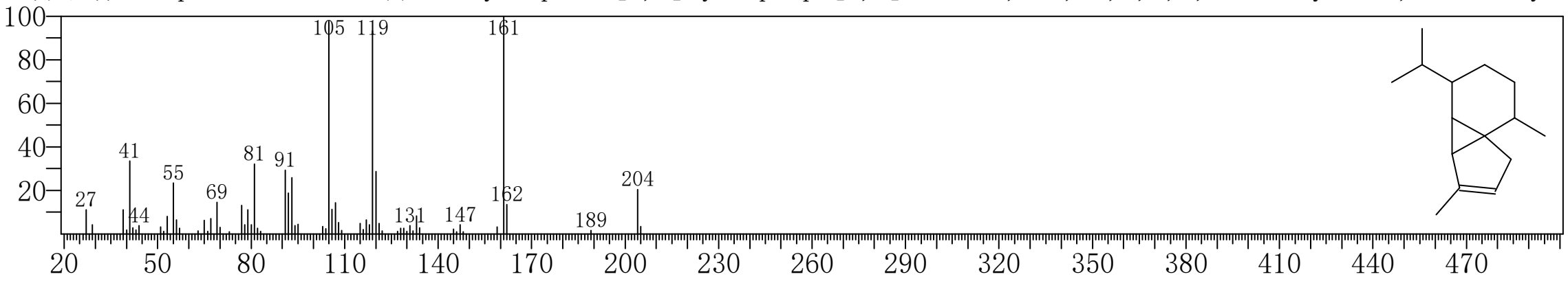

命中#:4　输入:22433　谱库:NIST17s.lib
SI:93　分子式:C15H24　CAS:3856-25-5　摩尔质量:204　保留指数:1221
组分名称:Copaene $$ Tricyclo[4.4.0.02,7]dec-3-ene, 1,3-dimethyl-8-(1-methylethyl)-, stereoisomer $$ Tricycl

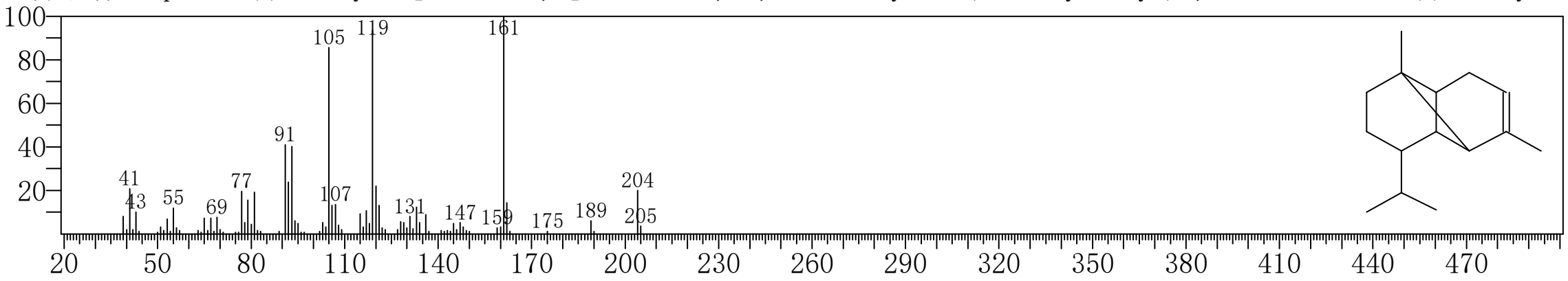

命中#:5　输入:22425　谱库:NIST17s.lib
SI:93　分子式:C15H24　CAS:17699-14-8　摩尔质量:204　保留指数:1344
组分名称:.alpha.-Cubebene $$ 1H-Cyclopenta[1,3]cyclopropa[1,2]benzene, 3a,3b,4,5,6,7-hexahydro-3,7-dimethyl-

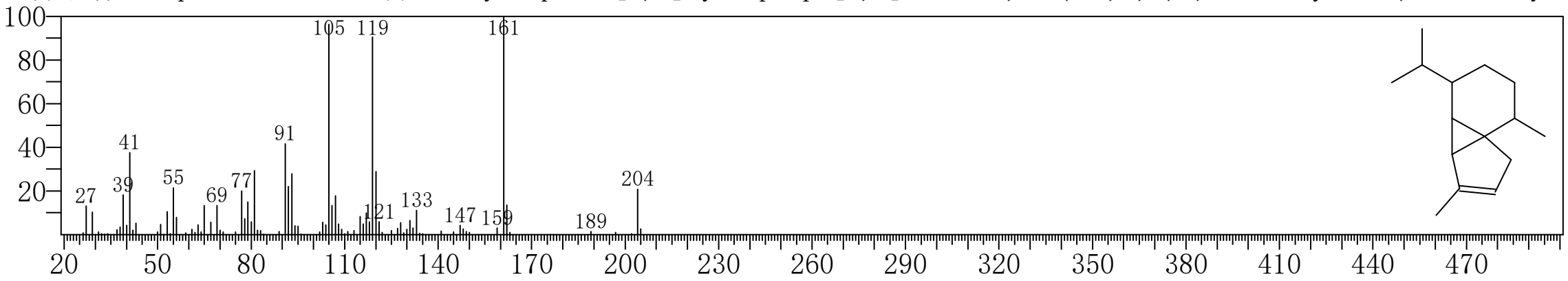

<< 目标组分 >>
行号#:29　保留时间:23.660(扫描数#:3733)　质量峰:266
原始模式:平均 23.655-23.665(3732-3734)　基峰:105.05(45536)
背景模式:峰校准　组 1 - 事件 1　Scan

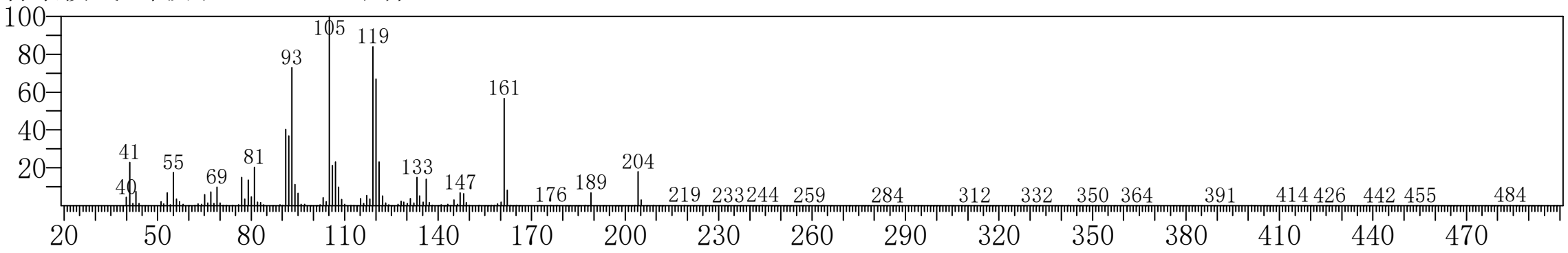

命中#:1　输入:52807　谱库:NIST17-1.lib
SI:94　分子式:C15H24　CAS:14912-44-8　摩尔质量:204　保留指数:1221
组分名称:Ylangene $$ Tricyclo[4.4.0.0(2,7)]dec-3-ene, 8-isopropyl-1,3-dimethyl-, (1S,2R,6R,7R,8S)-(+)- $$ (

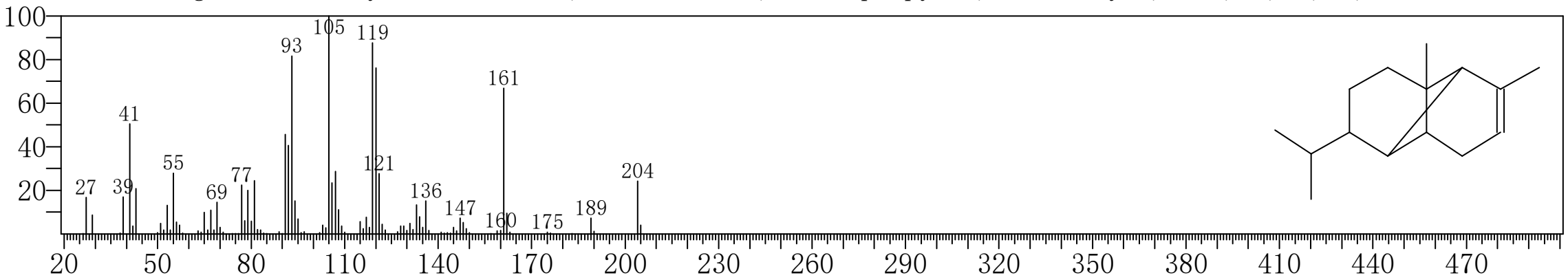

命中#:2　输入:22344　谱库:NIST17s.lib
SI:92　分子式:C15H24　CAS:14912-44-8　摩尔质量:204　保留指数:1221
组分名称:Ylangene $$ Tricyclo[4.4.0.0(2,7)]dec-3-ene, 8-isopropyl-1,3-dimethyl-, (1S,2R,6R,7R,8S)-(+)- $$ (

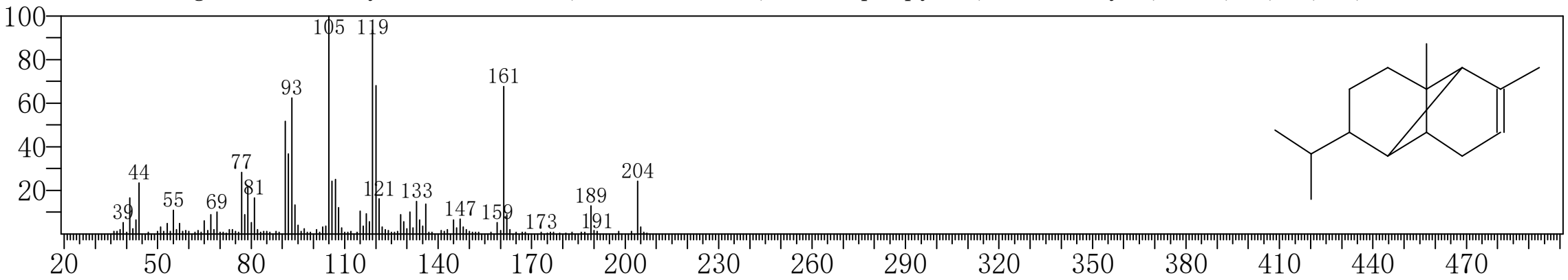

命中#:3　输入:1495　谱库:FFNSC1.3.lib
SI:92　分子式:C15 H24　CAS:14912-44-8　摩尔质量:204　保留指数:1371
组分名称:Ylangene

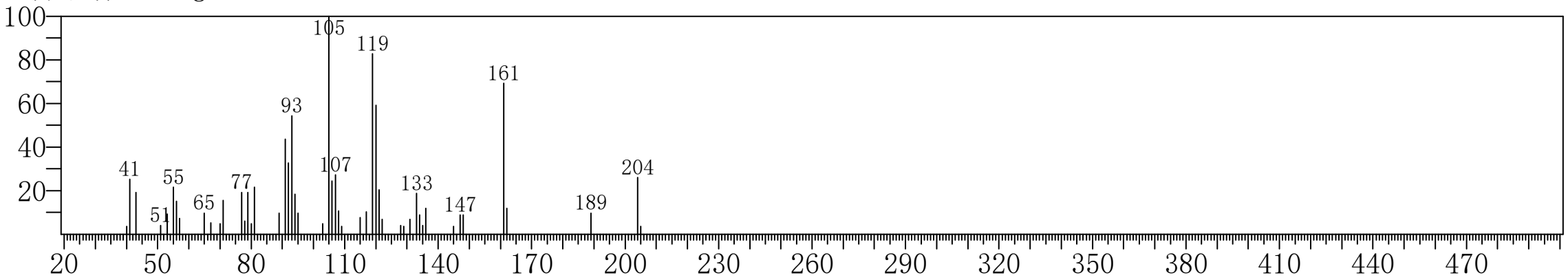

命中#:4　输入:22343　谱库:NIST17s.lib
SI:92　分子式:C15H24　CAS:14912-44-8　摩尔质量:204　保留指数:1221
组分名称:Ylangene $$ Tricyclo[4.4.0.0(2,7)]dec-3-ene, 8-isopropyl-1,3-dimethyl-, (1S,2R,6R,7R,8S)-(+)- $$ (

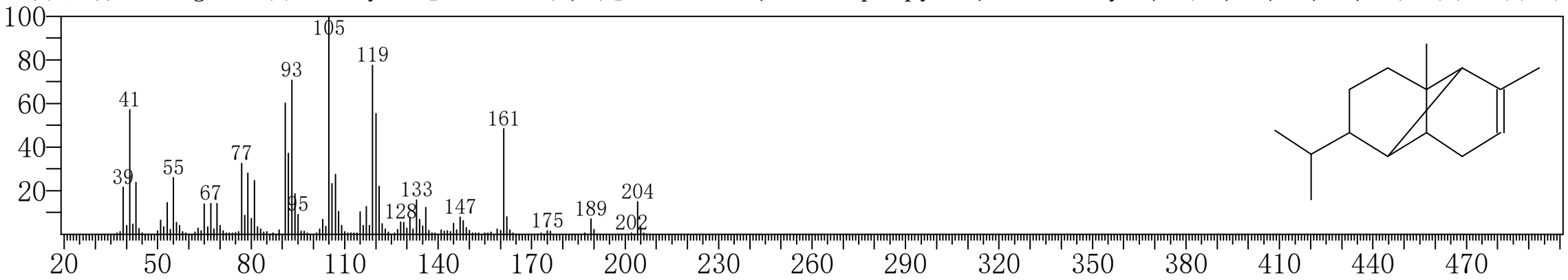

命中#:5　输入:22375　谱库:NIST17s.lib
SI:92　分子式:C15H24　CAS:14912-44-8　摩尔质量:204　保留指数:1221
组分名称:Ylangene $$ Tricyclo[4.4.0.0(2,7)]dec-3-ene, 8-isopropyl-1,3-dimethyl-, (1S,2R,6R,7R,8S)-(+)- $$ (

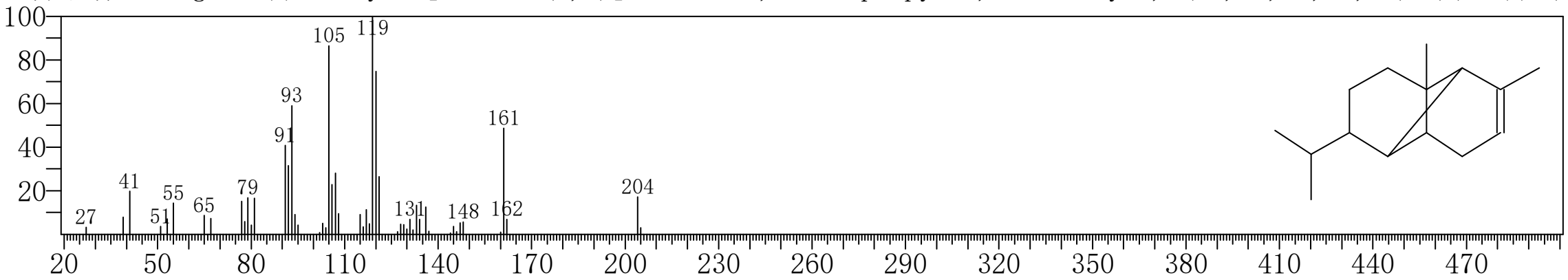

<< 目标组分 >>
行号#:30　 保留时间:23.975(扫描数#:3796)　质量峰:284
原始模式:平均 23.970-23.980(3795-3797)　 基峰:119.10(154889)
背景模式:峰校准　组 1 - 事件 1　Scan

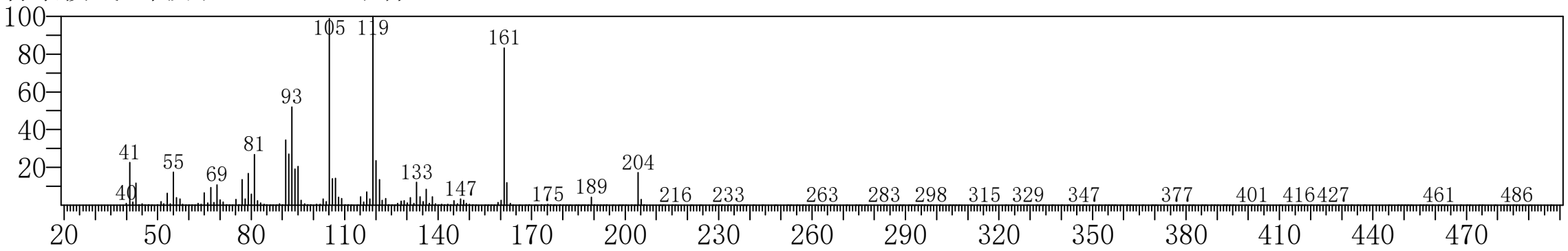

命中#:1　输入:52935　谱库:NIST17-1.lib
SI:94　分子式:C15H24　CAS:3856-25-5　摩尔质量:204　保留指数:1221
组分名称:Copaene $$ Tricyclo[4.4.0.02,7]dec-3-ene, 1,3-dimethyl-8-(1-methylethyl)-, stereoisomer $$ Tricycl

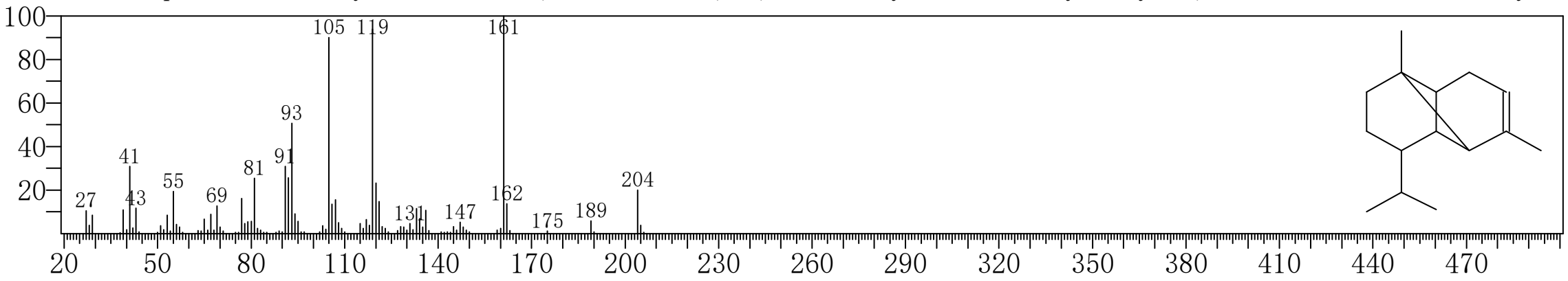

命中#:2　输入:1009　谱库:FFNSC1.3.lib
SI:93　分子式:C15 H24　CAS:138874-68-7　摩尔质量:204　保留指数:1375
组分名称:Copaene <alpha->

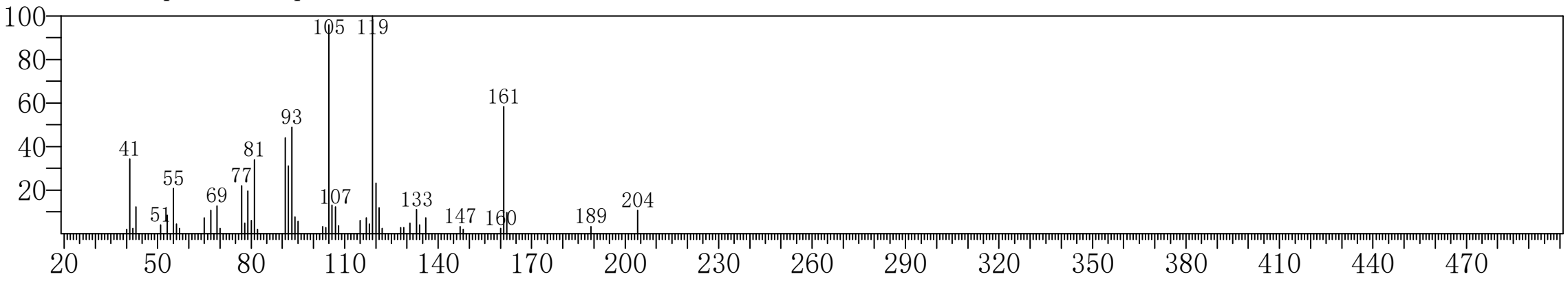

命中#:3　输入:22379　谱库:NIST17s.lib
SI:92　分子式:C15H24　CAS:3856-25-5　摩尔质量:204　保留指数:1221
组分名称:Copaene $$ Tricyclo[4.4.0.02,7]dec-3-ene, 1,3-dimethyl-8-(1-methylethyl)-, stereoisomer $$ Tricycl

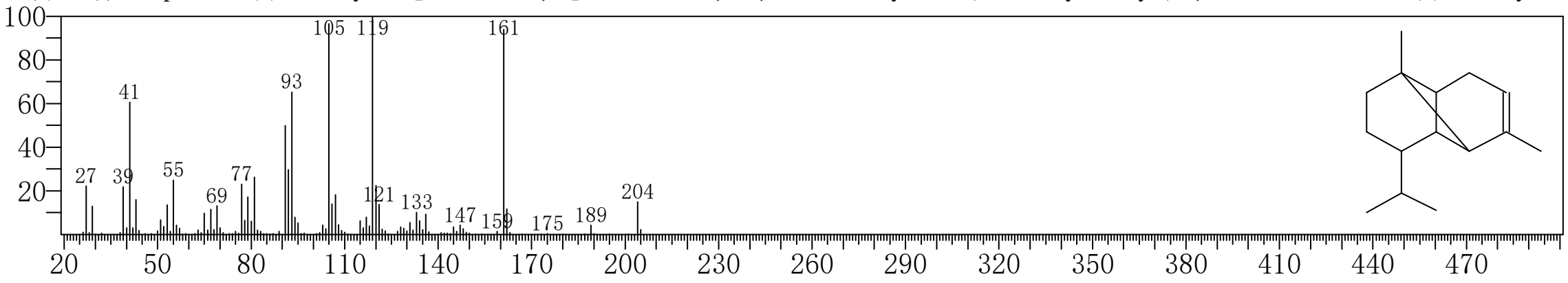

命中#:4　输入:22433　谱库:NIST17s.lib
SI:92　分子式:C15H24　CAS:3856-25-5　摩尔质量:204　保留指数:1221
组分名称:Copaene $$ Tricyclo[4.4.0.02,7]dec-3-ene, 1,3-dimethyl-8-(1-methylethyl)-, stereoisomer $$ Tricycl

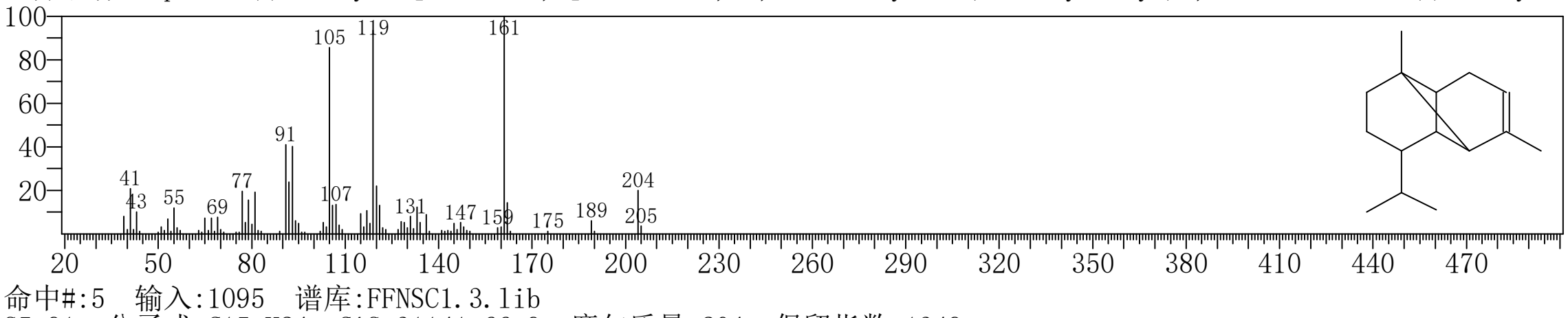

命中#:5　输入:1095　谱库:FFNSC1.3.lib
SI:91　分子式:C15 H24　CAS:31141-66-9　摩尔质量:204　保留指数:1349
组分名称:Cubebene <alpha->

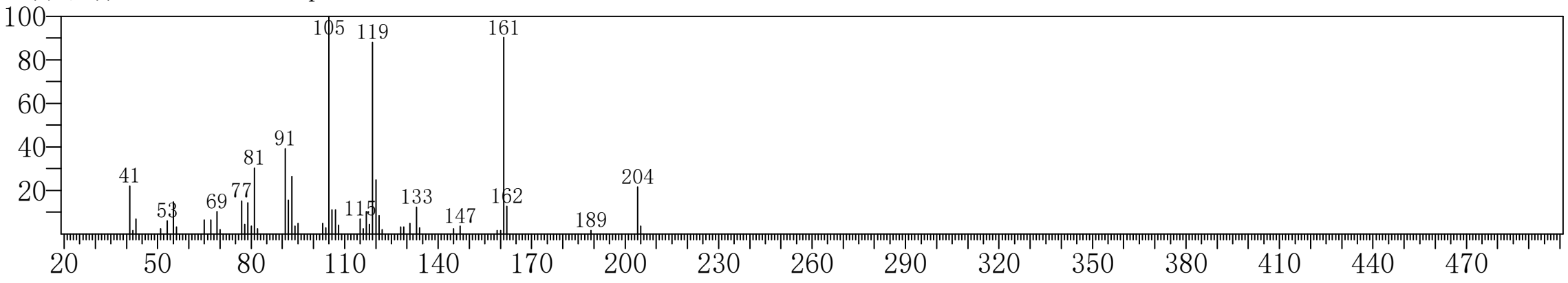

<< 目标组分 >>
行号#:31　保留时间:24.375(扫描数#:3876)　质量峰:279
原始模式:平均 24.370-24.380(3875-3877)　基峰:81.05(1049567)
背景模式:峰校准　组 1 - 事件 1　Scan

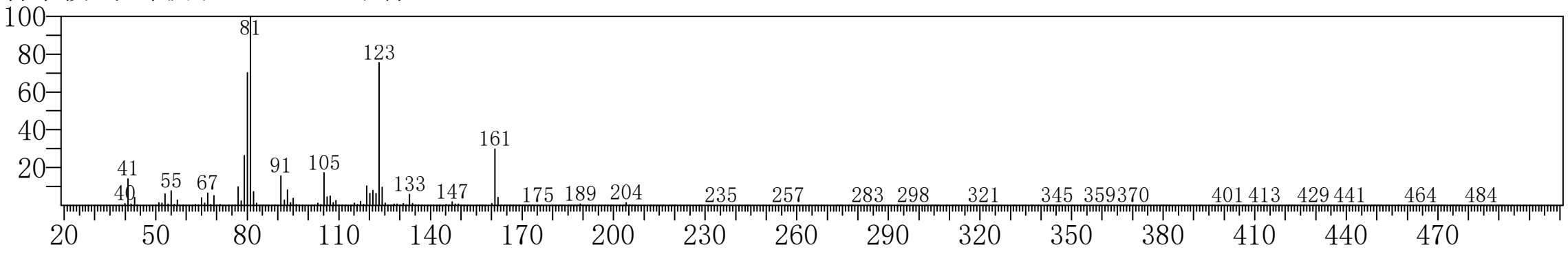

命中#:1　输入:22294　谱库:NIST17s.lib
SI:97　分子式:C15H24　CAS:5208-59-3　摩尔质量:204　保留指数:1339
组分名称:(-)-.beta.-Bourbonene $$ Cyclobuta[1,2:3,4]dicyclopentene, decahydro-3a-methyl-6-methylene-1-(1-me

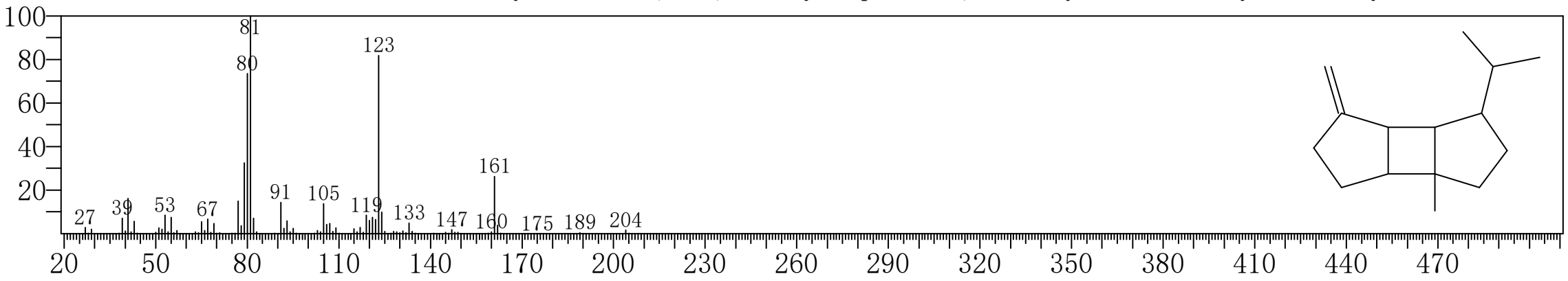

命中#:2　输入:22293　谱库:NIST17s.lib
SI:96　分子式:C15H24　CAS:5208-59-3　摩尔质量:204　保留指数:1339
组分名称:(-)-.beta.-Bourbonene $$ Cyclobuta[1,2:3,4]dicyclopentene, decahydro-3a-methyl-6-methylene-1-(1-me

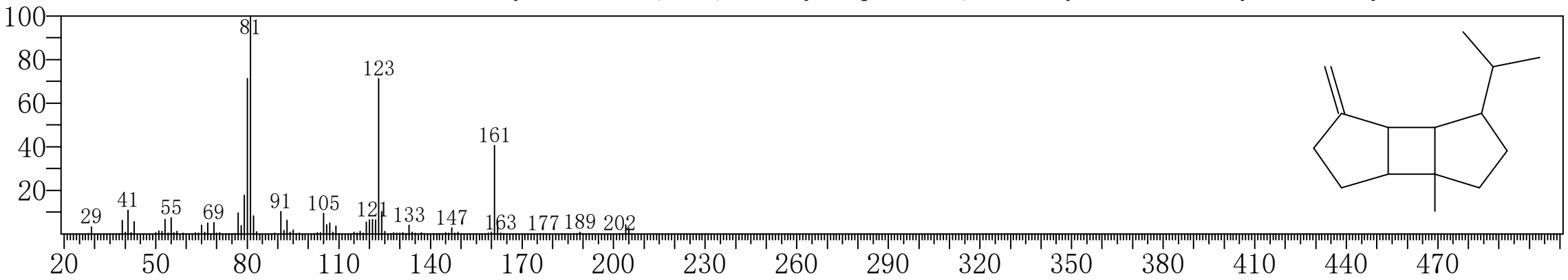

命中#:3　输入:52752　谱库:NIST17-1.lib
SI:95　分子式:C15H24　CAS:5208-59-3　摩尔质量:204　保留指数:1339
组分名称:(-)-.beta.-Bourbonene $$ Cyclobuta[1,2:3,4]dicyclopentene, decahydro-3a-methyl-6-methylene-1-(1-me

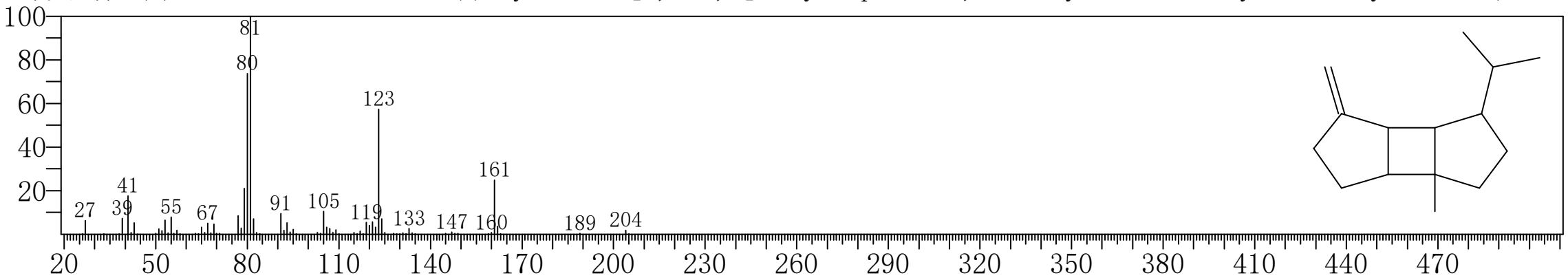

命中#:4　输入:1040　谱库:FFNSC1.3.lib
SI:95　分子式:C15 H24　CAS:119903-95-6　摩尔质量:204　保留指数:1382
组分名称:Bourbonene <beta->

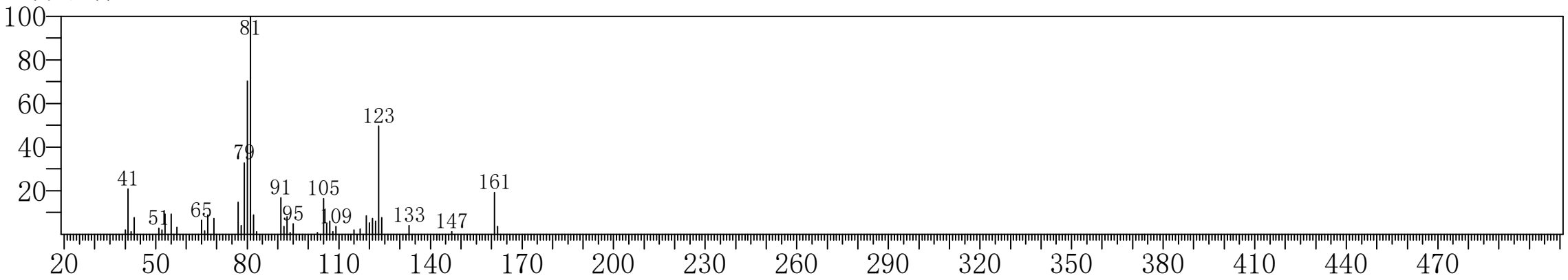

命中#:5　输入:22290　谱库:NIST17s.lib
SI:90　分子式:C15H24　CAS:5208-59-3　摩尔质量:204　保留指数:1339
组分名称:(-)-.beta.-Bourbonene $$ Cyclobuta[1,2:3,4]dicyclopentene, decahydro-3a-methyl-6-methylene-1-(1-me

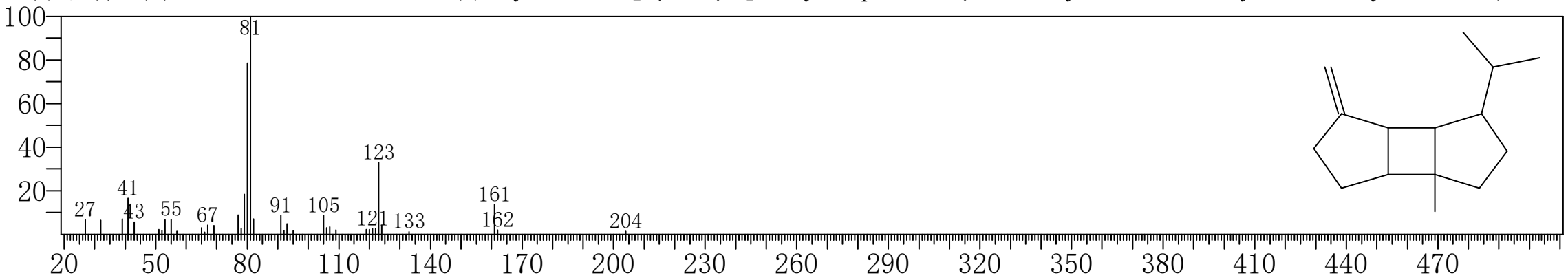

<< 目标组分 >>
行号#:32　保留时间:24.685(扫描数#:3938)　质量峰:284
原始模式:平均 24.680-24.690(3937-3939)　基峰:93.10(55281)
背景模式:峰校准　组 1 - 事件 1　Scan

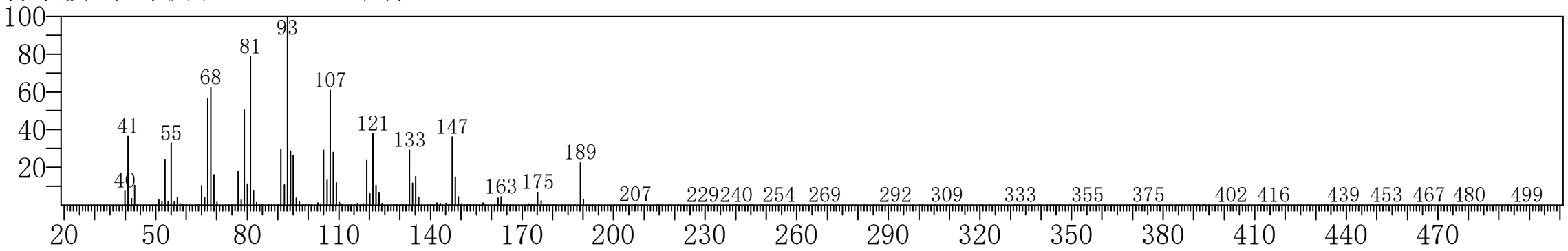

命中#:1　输入:52754　谱库:NIST17-1.lib
SI:94　分子式:C15H24　CAS:515-13-9　摩尔质量:204　保留指数:1398
组分名称:Cyclohexane, 1-ethenyl-1-methyl-2,4-bis(1-methylethenyl)-, [1S-(1.alpha.,2.beta.,4.beta.)]- $$ Cyc

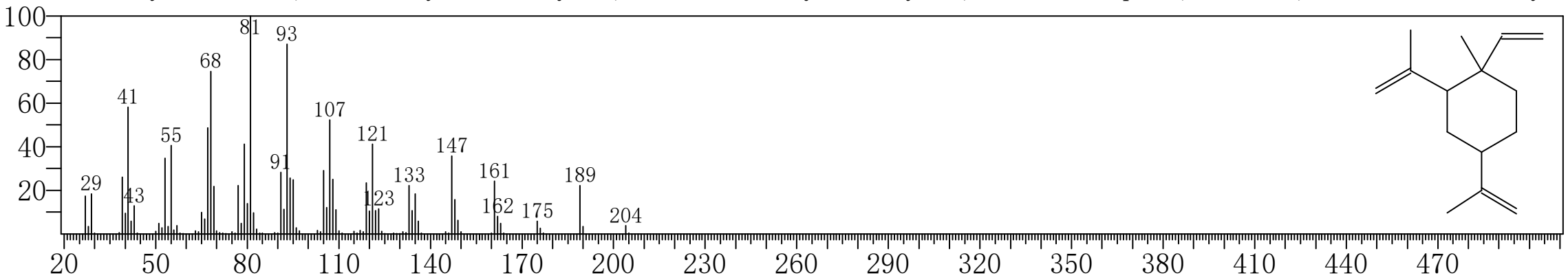

命中#:2　输入:22292　谱库:NIST17s.lib
SI:92　分子式:C15H24　CAS:515-13-9　摩尔质量:204　保留指数:1398
组分名称:Cyclohexane, 1-ethenyl-1-methyl-2,4-bis(1-methylethenyl)-, [1S-(1.alpha.,2.beta.,4.beta.)]- $$ Cyc

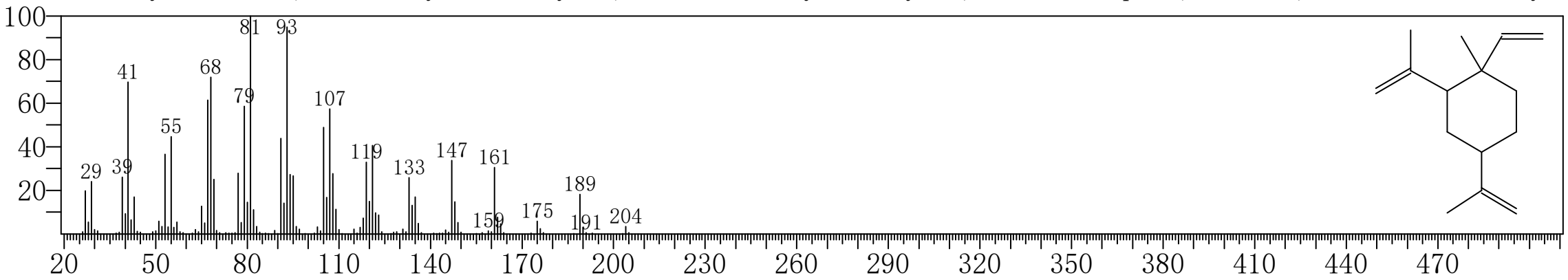

命中#:3　输入:965　谱库:FFNSC1.3.lib
SI:91　分子式:C15 H24　CAS:33880-83-0　摩尔质量:204　保留指数:1390
组分名称:Elemene <beta->

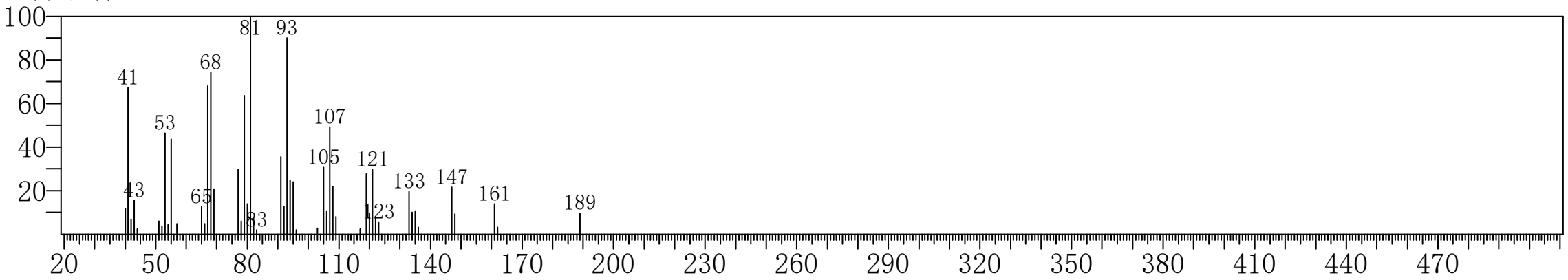

命中#:4　输入:52751　谱库:NIST17-1.lib
SI:90　分子式:C15H24　CAS:33880-83-0　摩尔质量:204　保留指数:1398
组分名称:Cyclohexane, 1-ethenyl-1-methyl-2,4-bis(1-methylethenyl)-, (1.alpha.,2.beta.,4.beta.)- $$ 2,4-Diis

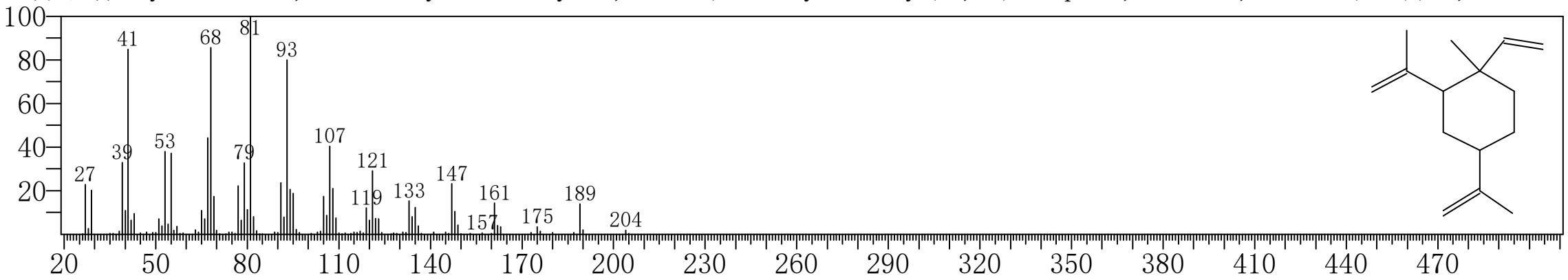

命中#:5　输入:52736　谱库:NIST17-1.lib
SI:90　分子式:C15H24　CAS:75023-40-4　摩尔质量:204　保留指数:1570
组分名称:1,5-Cyclodecadiene, 1,5-dimethyl-8-(1-methylethenyl)-, [S-(Z,E)]- $$ 8-Isopropenyl-1,5-dimethyl-1,

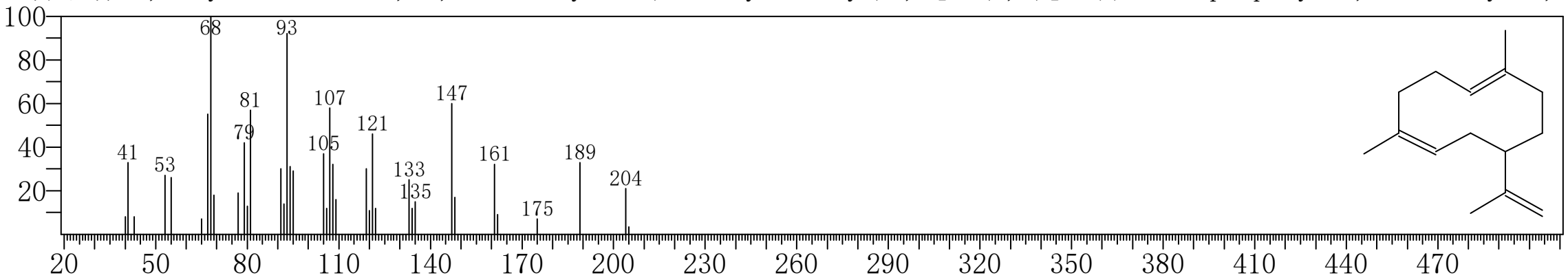

<< 目标组分 >>
行号#:33　保留时间:26.345(扫描数#:4270)　质量峰:269
原始模式:平均 26.340-26.350(4269-4271)　基峰:93.10(4250187)
背景模式:峰校准　组 1 - 事件 1　Scan

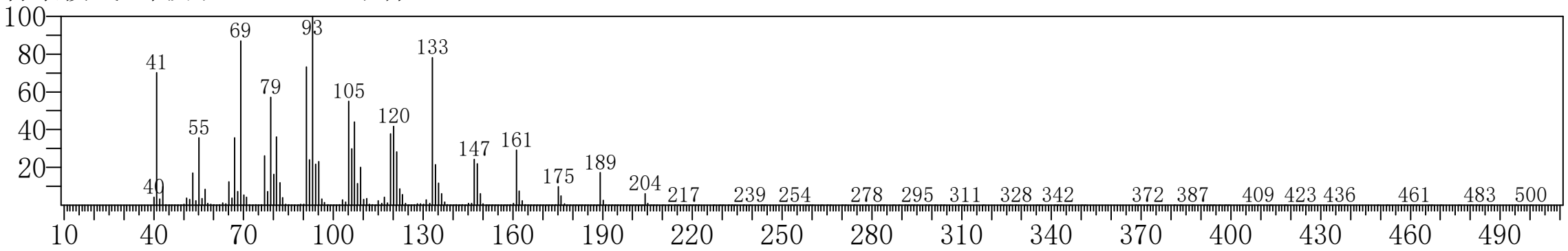

命中#:1　输入:22309　谱库:NIST17s.lib
SI:97　分子式:C15H24　CAS:87-44-5　摩尔质量:204　保留指数:1494
组分名称:Caryophyllene $$ Bicyclo[7.2.0]undec-4-ene, 4,11,11-trimethyl-8-methylene-, [1R-(1R*,4E,9S*)]- $$ I

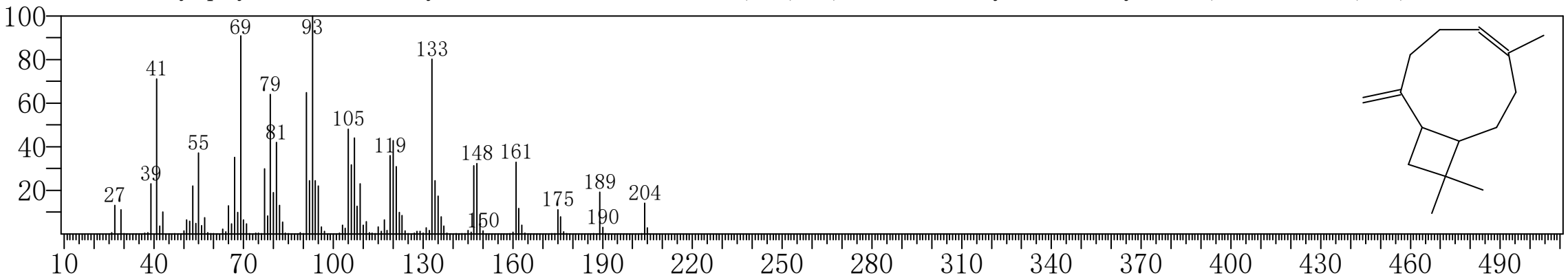

命中#:2　输入:1206　谱库:FFNSC1.3.lib
SI:96　分子式:C15 H24　CAS:13877-93-5　摩尔质量:204　保留指数:1424
组分名称:Caryophyllene <(E)->

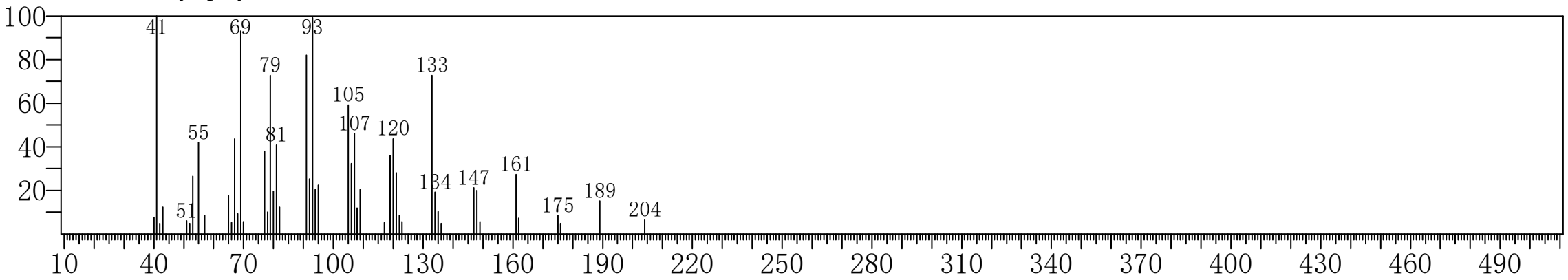

命中#:3　输入:22308　谱库:NIST17s.lib
SI:96　分子式:C15H24　CAS:13877-93-5　摩尔质量:204　保留指数:1494
组分名称:Bicyclo[7.2.0]undec-4-ene, 4,11,11-trimethyl-8-methylene- $$ Bicyclo[7.2.0]undec-4-ene, 4,11,11-tr

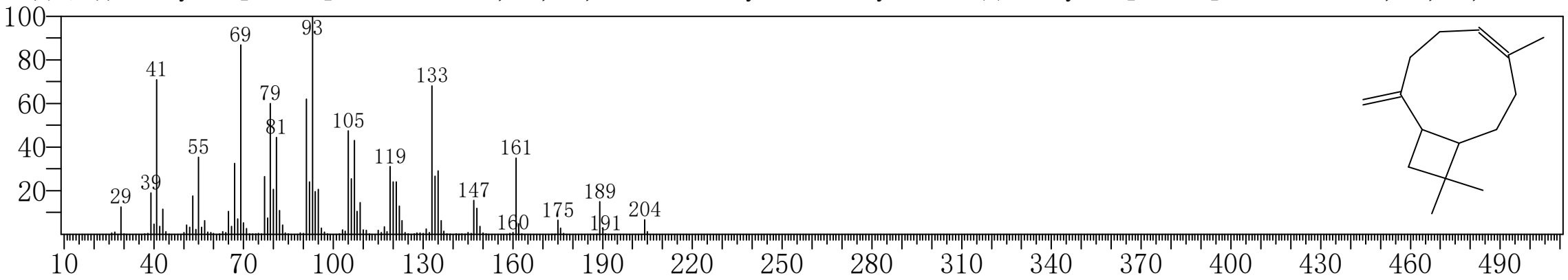

命中#:4　输入:52786　谱库:NIST17-1.lib
SI:95　分子式:C15H24　CAS:87-44-5　摩尔质量:204　保留指数:1494
组分名称:Caryophyllene $$ Bicyclo[7.2.0]undec-4-ene, 4,11,11-trimethyl-8-methylene-, [1R-(1R*,4E,9S*)]- $$ I

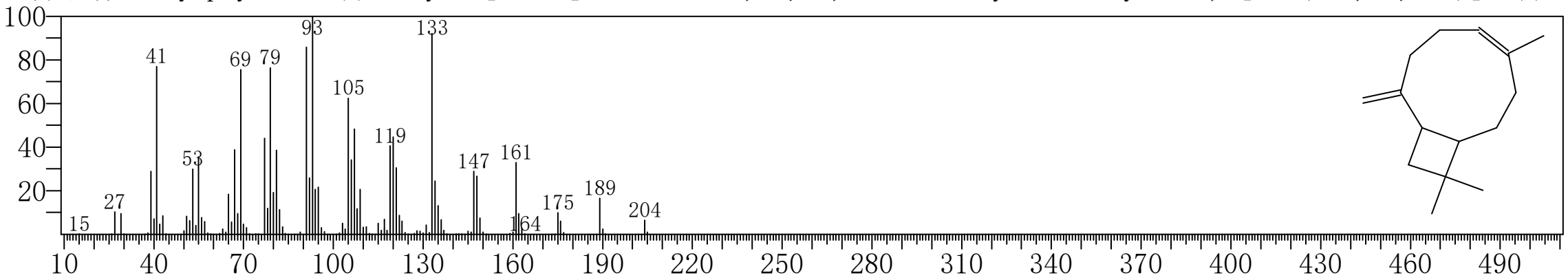

命中#:5　输入:22307　谱库:NIST17s.lib
SI:94　分子式:C15H24　CAS:87-44-5　摩尔质量:204　保留指数:1494
组分名称:Caryophyllene $$ Bicyclo[7.2.0]undec-4-ene, 4,11,11-trimethyl-8-methylene-, [1R-(1R*,4E,9S*)]- $$ I

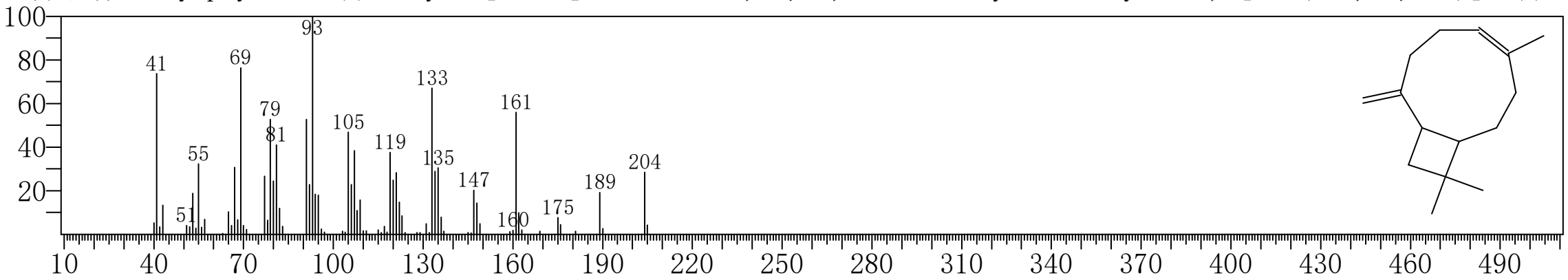

<< 目标组分 >>
行号#:34　保留时间:26.845(扫描数#:4370)　质量峰:282
原始模式:平均 26.840-26.850(4369-4371)　基峰:161.15(343428)
背景模式:峰校准　组 1 - 事件 1　Scan

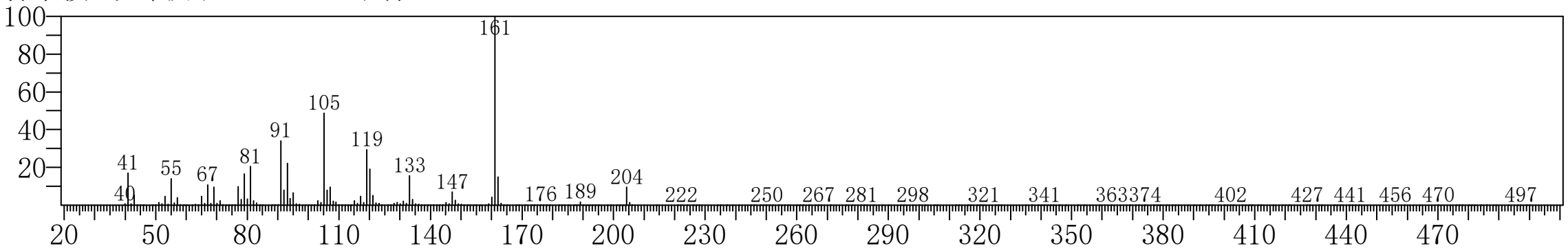

命中#:1　输入:52920　谱库:NIST17-1.lib
SI:96　分子式:C15H24　CAS:18252-44-3　摩尔质量:204　保留指数:1216
组分名称:(1R,2S,6S,7S,8S)-8-Isopropyl-1-methyl-3-methylenetricyclo[4.4.0.02,7]decane-rel- $$ Tricyclo[4.4.0.

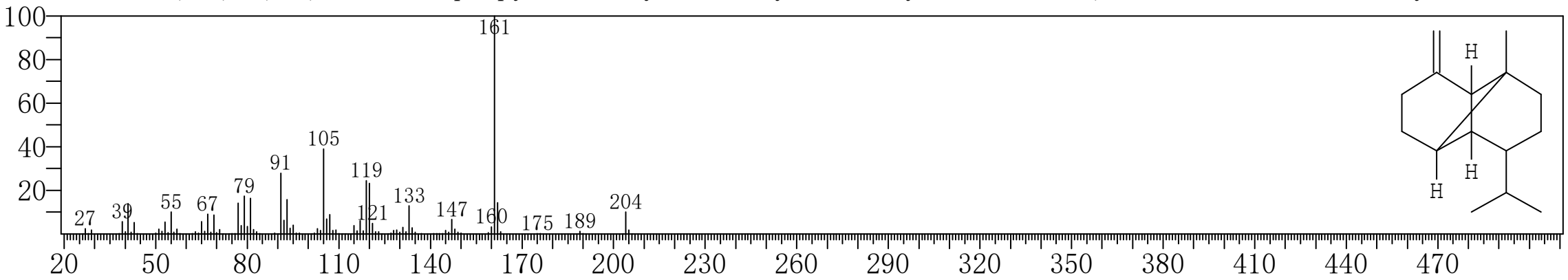

命中#:2　输入:1471　谱库:FFNSC1.3.lib
SI:95　分子式:C15 H24　CAS:13744-15-5　摩尔质量:204　保留指数:1392
组分名称:Cubebene <beta->

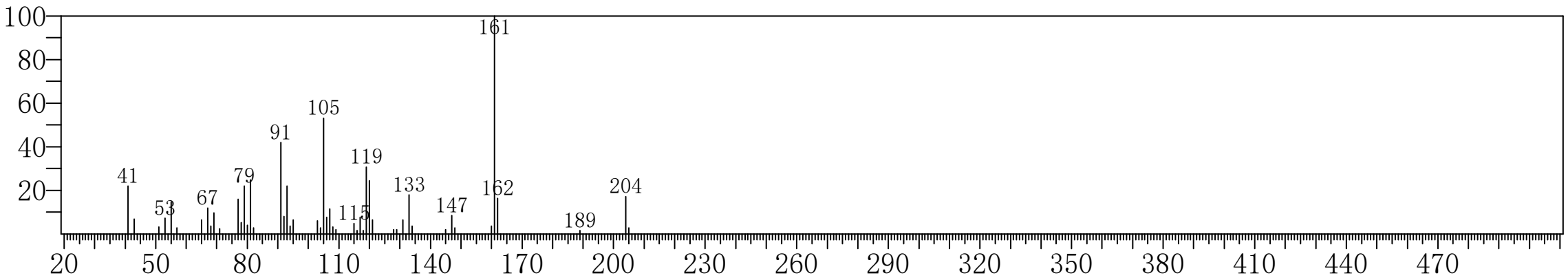

命中#:3　输入:1673　谱库:FFNSC1.3.lib
SI:94　分子式:C15 H24　CAS:6980-46-7　摩尔质量:204　保留指数:1490
组分名称:Amorphene <gamma->

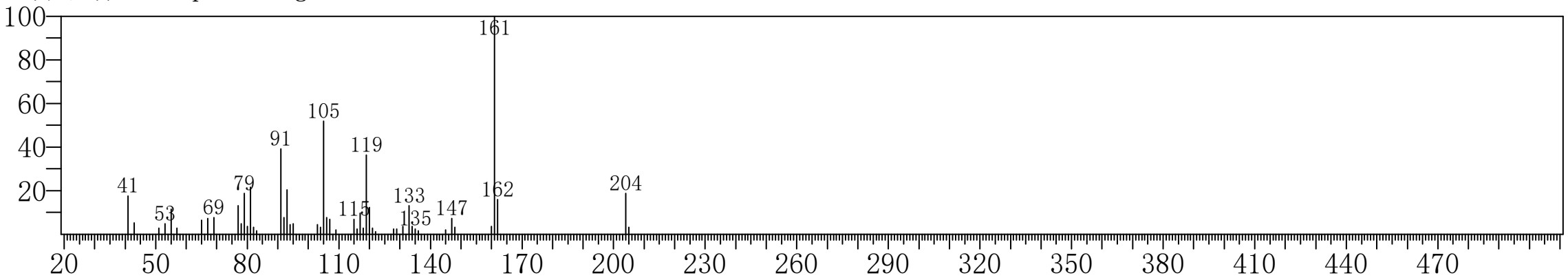

命中#:4　输入:52916　谱库:NIST17-1.lib
SI:94　分子式:C15H24　CAS:13744-15-5　摩尔质量:204　保留指数:1339
组分名称:1H-Cyclopenta[1,3]cyclopropa[1,2]benzene, octahydro-7-methyl-3-methylene-4-(1-methylethyl)-, [3aS-

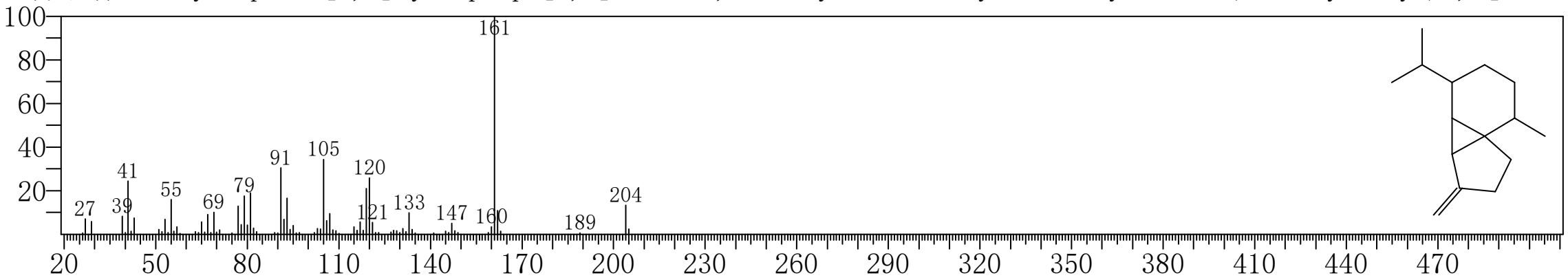

命中#:5　输入:22423　谱库:NIST17s.lib
SI:91　分子式:C15H24　CAS:39029-41-9　摩尔质量:204　保留指数:1435
组分名称:Naphthalene, 1,2,3,4,4a,5,6,8a-octahydro-7-methyl-4-methylene-1-(1-methylethyl)-, (1.alpha.,4a.beta

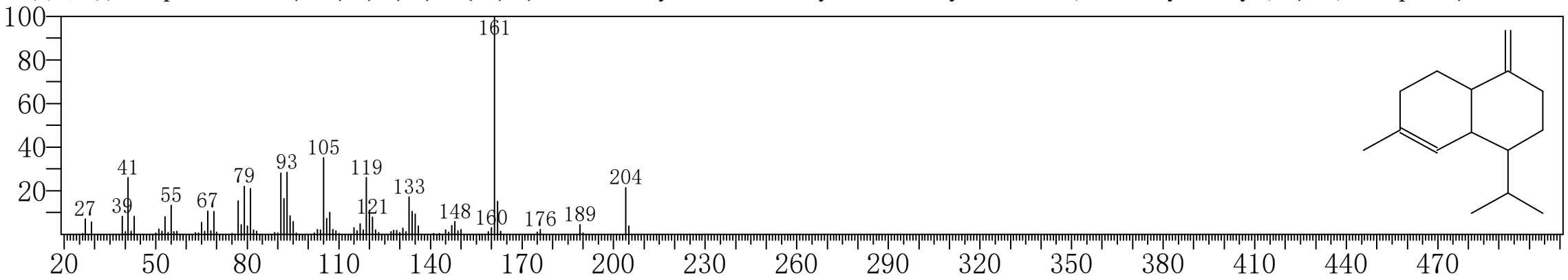

<< 目标组分 >>
行号#:35　 保留时间:27.670(扫描数#:4535)　质量峰:252
原始模式:平均 27.665-27.675(4534-4536)　　基峰:161.15(35543)
背景模式:峰校准　组 1 - 事件 1　Scan

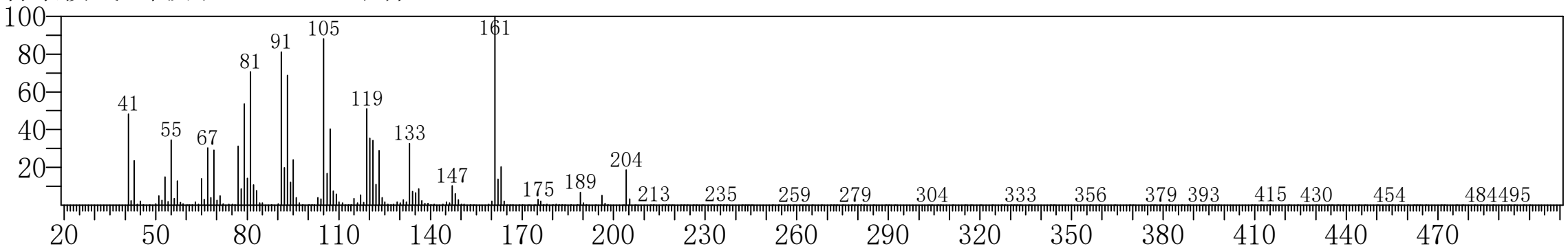

命中#:1　输入:1676　谱库:FFNSC1.3.lib
SI:94　分子式:C15 H24　CAS:317819-80-0　摩尔质量:204　保留指数:1447
组分名称:Isogermacrene D

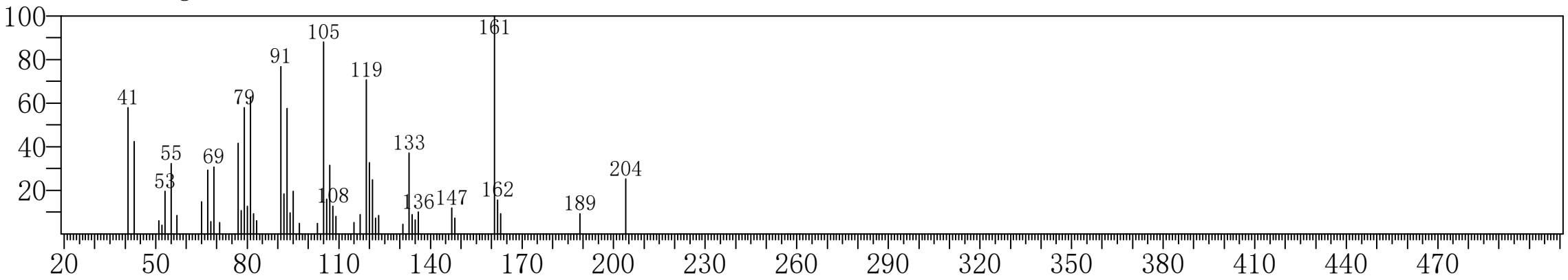

命中#:2　输入:52912　谱库:NIST17-1.lib
SI:93　分子式:C15H24　CAS:317819-80-0　摩尔质量:204　保留指数:1515
组分名称:(S,1Z,6Z)-8-Isopropyl-1-methyl-5-methylenecyclodeca-1,6-diene $$ 1,6-Cyclodecadiene, 1-methyl-5-me

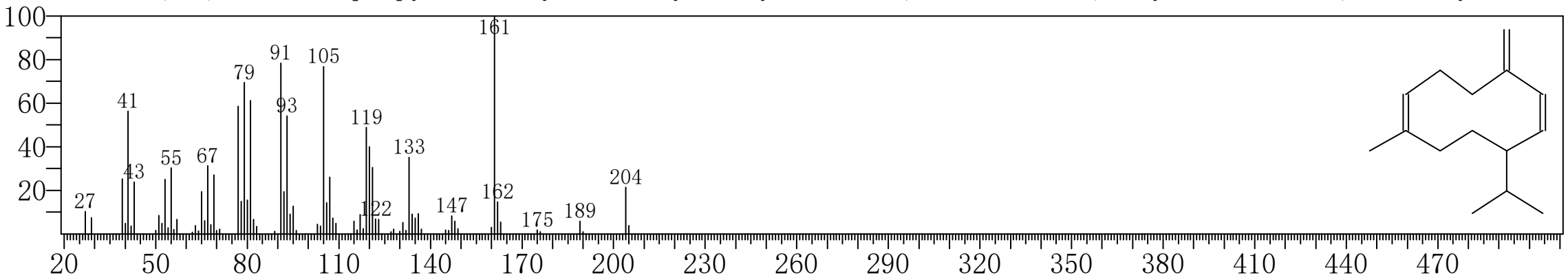

命中#:3　输入:1589　谱库:FFNSC1.3.lib
SI:90　分子式:C15 H24　CAS:29873-99-2　摩尔质量:204　保留指数:1432
组分名称:Elemene <gamma->

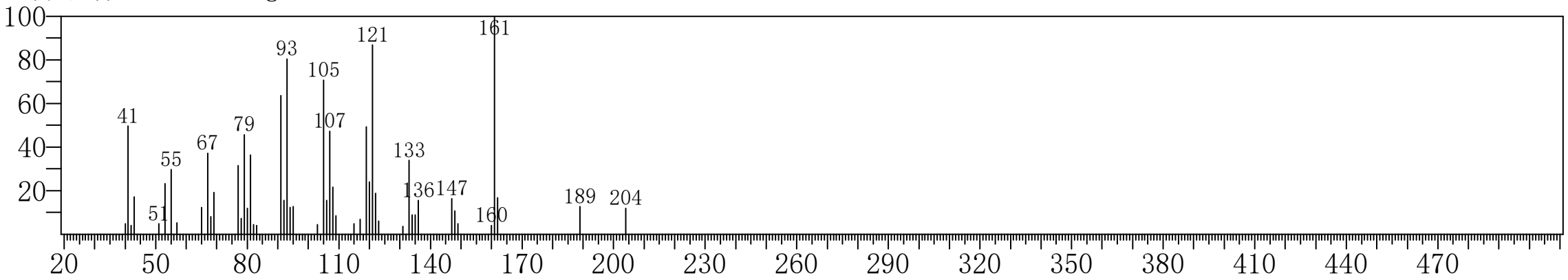

命中#:4　输入:969　谱库:FFNSC1.3.lib
SI:89　分子式:C15 H24　CAS:105453-16-5　摩尔质量:204　保留指数:1480
组分名称:Germacrene D

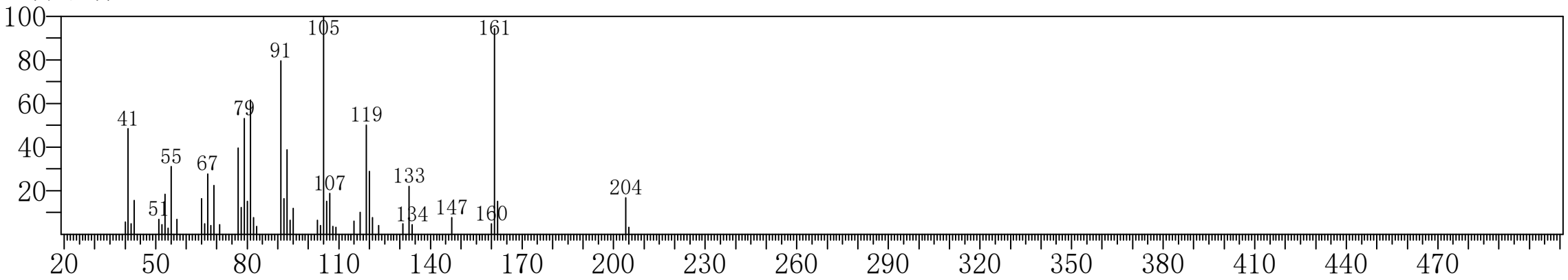

命中#:5　输入:1678　谱库:FFNSC1.3.lib
SI:89　分子式:C15 H24　CAS:54932-90-0　摩尔质量:204　保留指数:1502
组分名称:Amorphene <epsylon->

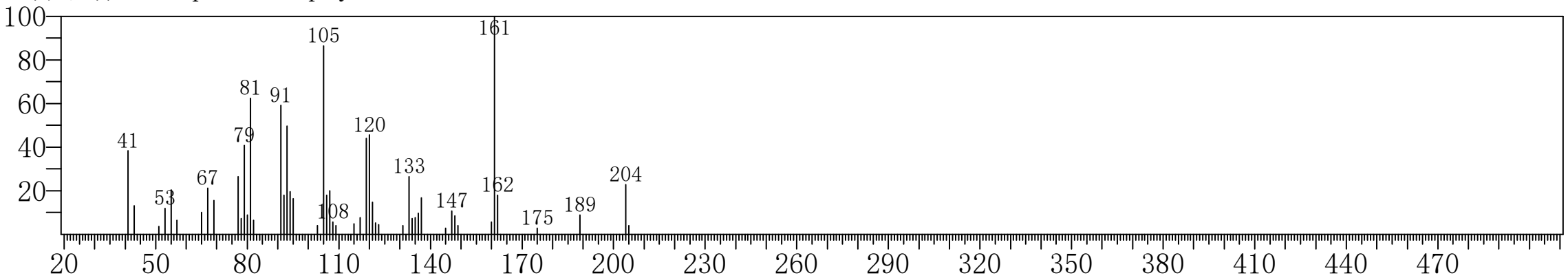

<< 目标组分 >>
行号#:36　保留时间:28.335(扫描数#:4668)　质量峰:296
原始模式:平均 28.330-28.340(4667-4669)　基峰:93.10(2584228)
背景模式:峰校准　组 1 - 事件 1　Scan

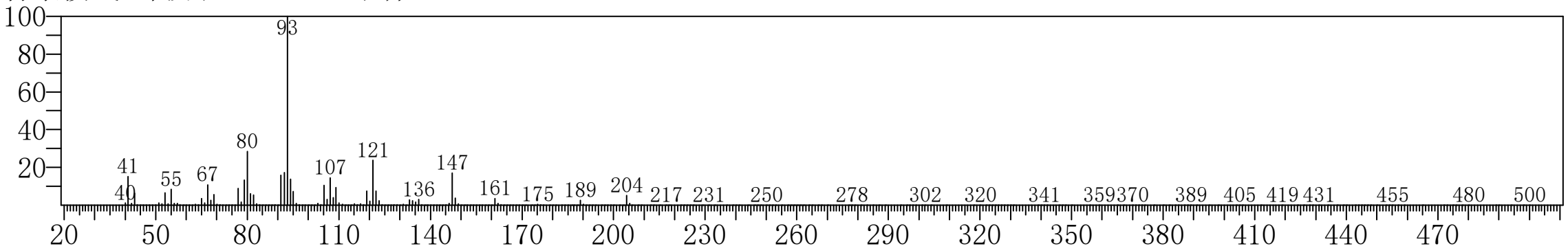

命中#:1　输入:1092　谱库:FFNSC1.3.lib
SI:97　分子式:C15 H24　CAS:6753-98-6　摩尔质量:204　保留指数:1454
组分名称:Humulene <alpha->

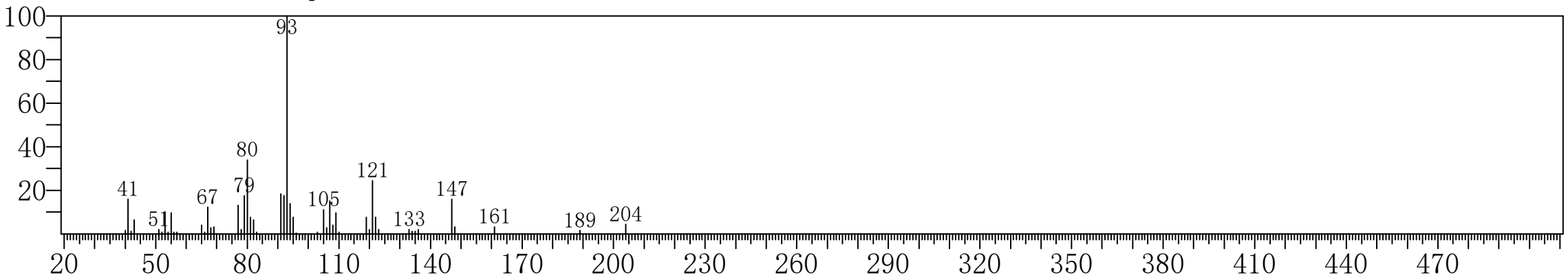

命中#:2　输入:52776　谱库:NIST17-1.lib
SI:96　分子式:C15H24　CAS:0-00-0　摩尔质量:204　保留指数:1579
组分名称:1,4,7,-Cycloundecatriene, 1,5,9,9-tetramethyl-, Z,Z,Z- $$ 1,5,9,9-Tetramethyl-1,4,7-cycloundecatri

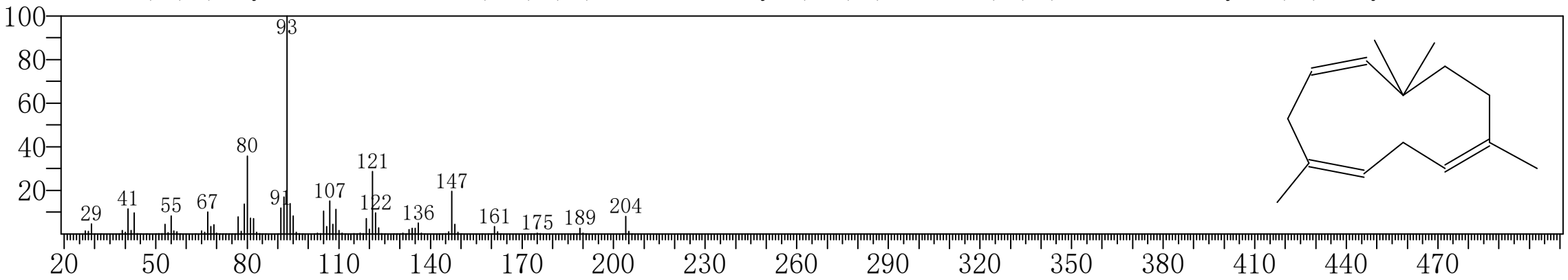

命中#:3　输入:22310　谱库:NIST17s.lib
SI:96　分子式:C15H24　CAS:6753-98-6　摩尔质量:204　保留指数:1579
组分名称:Humulene $$ .alpha.-Caryophyllene $$ 1,4,8-Cycloundecatriene, 2,6,6,9-tetramethyl-, (E,E,E)- $$ .a

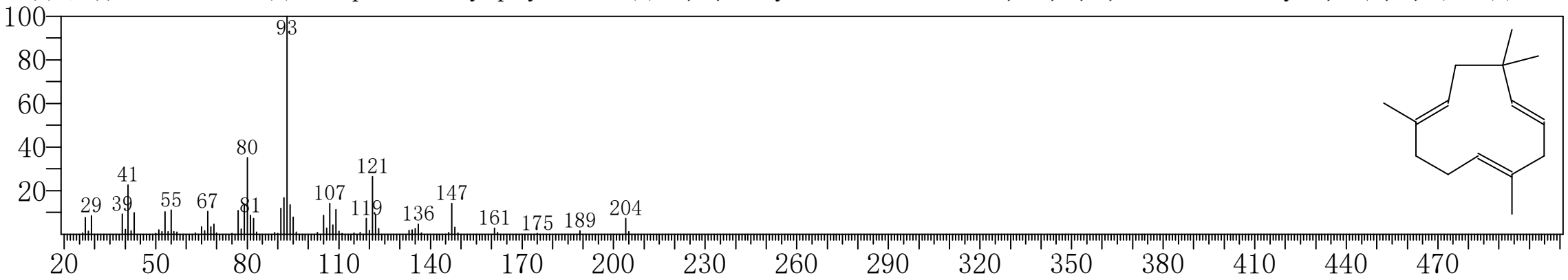

命中#:4　输入:52774　谱库:NIST17-1.lib
SI:95　分子式:C15H24　CAS:6753-98-6　摩尔质量:204　保留指数:1579
组分名称:Humulene $$ .alpha.-Caryophyllene $$ 1,4,8-Cycloundecatriene, 2,6,6,9-tetramethyl-, (E,E,E)- $$ .a

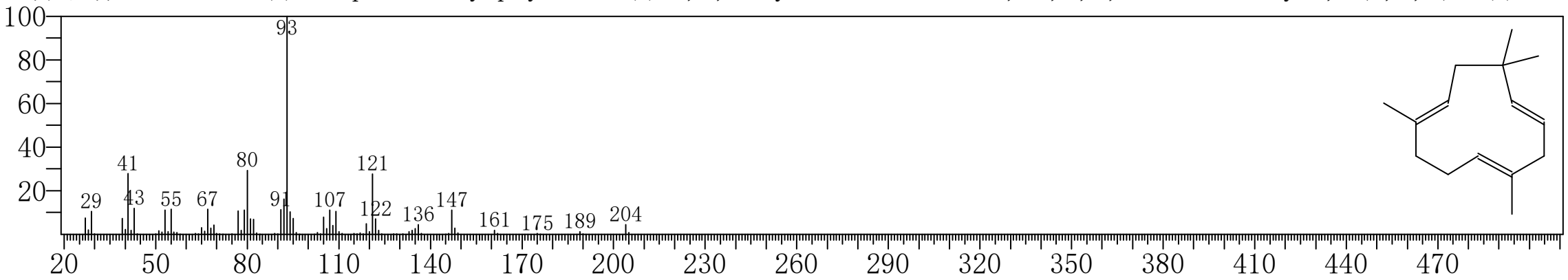

命中#:5　输入:22303　谱库:NIST17s.lib
SI:90　分子式:C15H24　CAS:6753-98-6　摩尔质量:204　保留指数:1579
组分名称:Humulene $$ .alpha.-Caryophyllene $$ 1,4,8-Cycloundecatriene, 2,6,6,9-tetramethyl-, (E,E,E)- $$ .a

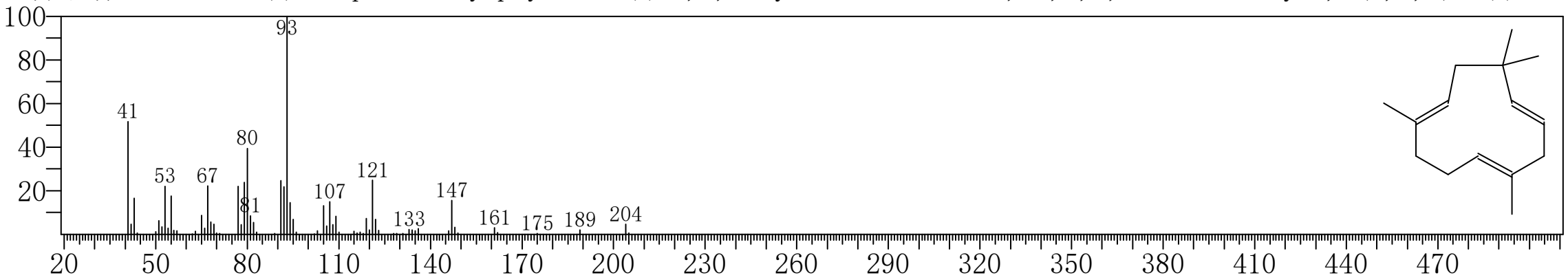

<< 目标组分 >>
行号#:37　保留时间:29.685(扫描数#:4938)　质量峰:221
原始模式:平均 29.680-29.690(4937-4939)　基峰:161.15(702185)
背景模式:峰校准　组 1 - 事件 1　Scan

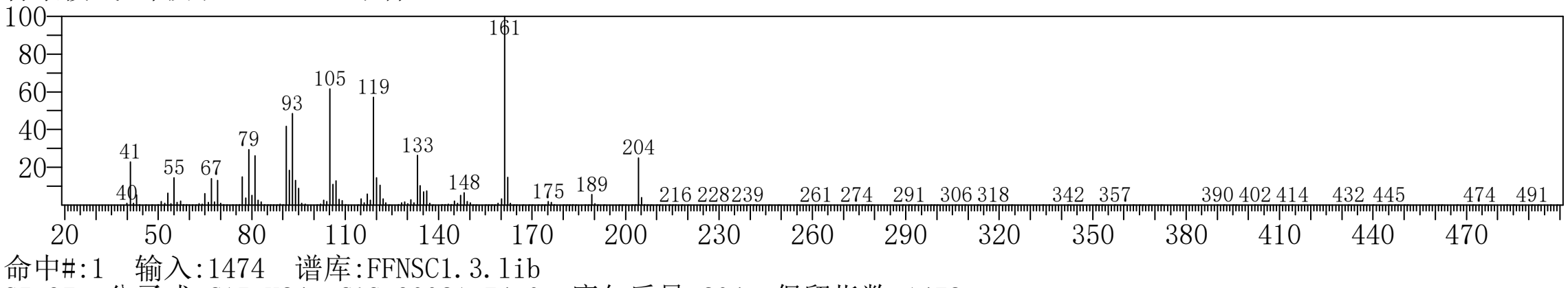

命中#:1　输入:1474　谱库:FFNSC1.3.lib
SI:97　分子式:C15 H24　CAS:30021-74-0　摩尔质量:204　保留指数:1478
组分名称:Muurolene <gamma->

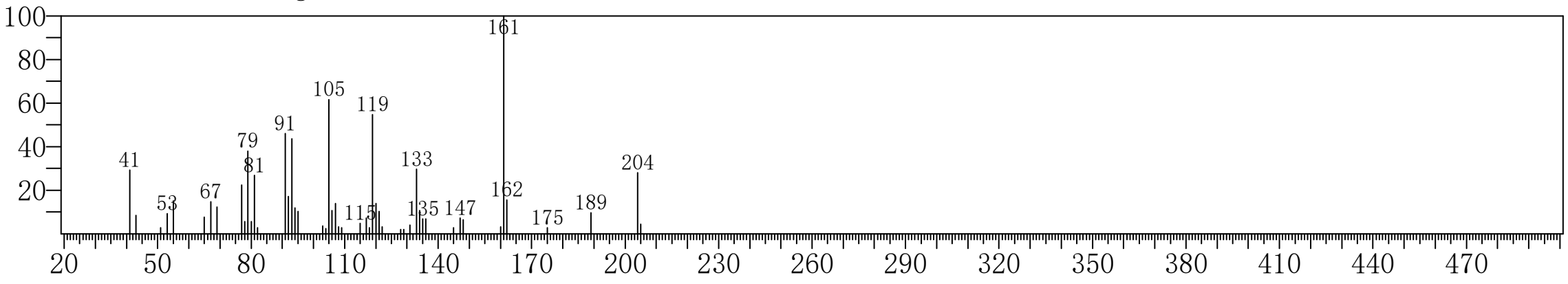

命中#:2　输入:1093　谱库:FFNSC1.3.lib
SI:96　分子式:C15 H24　CAS:38357-83-4　摩尔质量:204　保留指数:1512
组分名称:Cadinene <gamma->

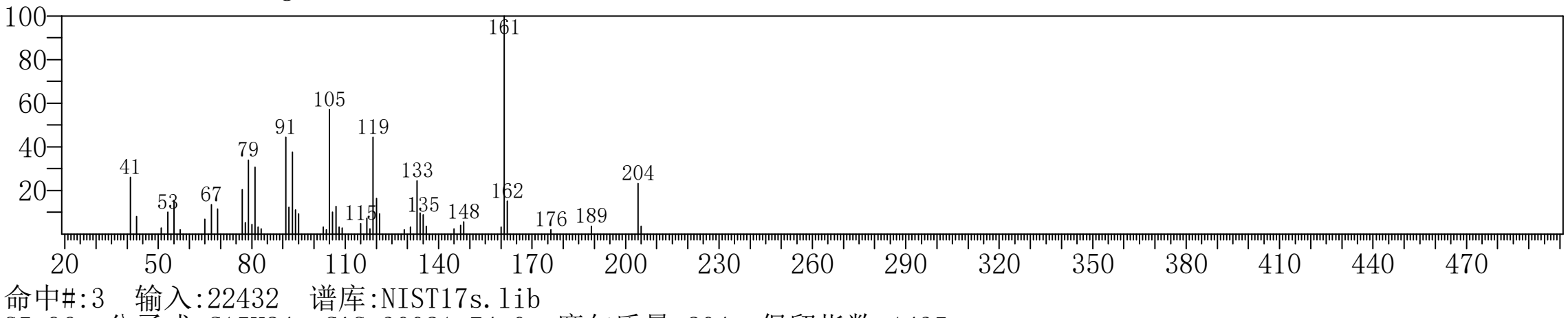

命中#:3　输入:22432　谱库:NIST17s.lib
SI:96　分子式:C15H24　CAS:30021-74-0　摩尔质量:204　保留指数:1435
组分名称:.gamma.-Muurolene $$ Naphthalene, 1,2,3,4,4a,5,6,8a-octahydro-7-methyl-4-methylene-1-(1-methylethyl

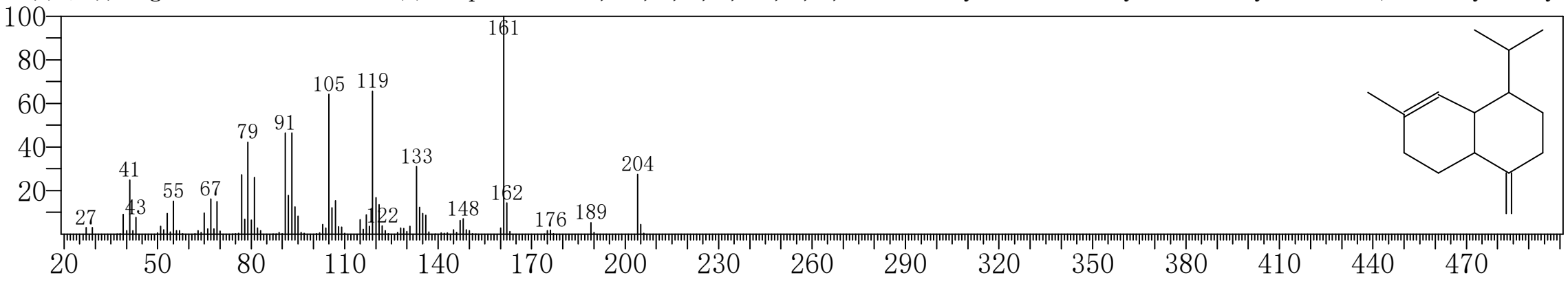

命中#:4　输入:22412　谱库:NIST17s.lib
SI:95　分子式:C15H24　CAS:30021-74-0　摩尔质量:204　保留指数:1435
组分名称:.gamma.-Muurolene $$ Naphthalene, 1,2,3,4,4a,5,6,8a-octahydro-7-methyl-4-methylene-1-(1-methylethyl

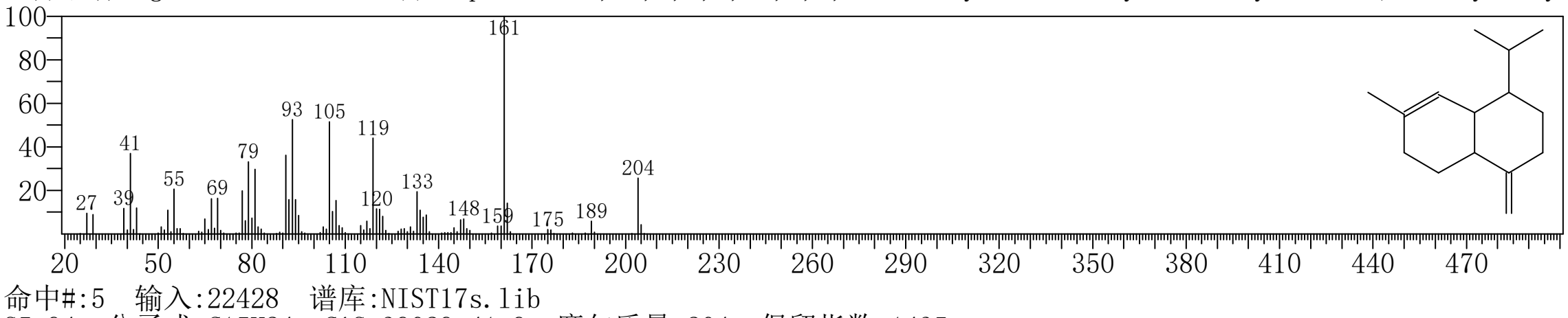

命中#:5　输入:22428　谱库:NIST17s.lib
SI:94　分子式:C15H24　CAS:39029-41-9　摩尔质量:204　保留指数:1435
组分名称:Naphthalene, 1,2,3,4,4a,5,6,8a-octahydro-7-methyl-4-methylene-1-(1-methylethyl)-, (1.alpha.,4a.beta

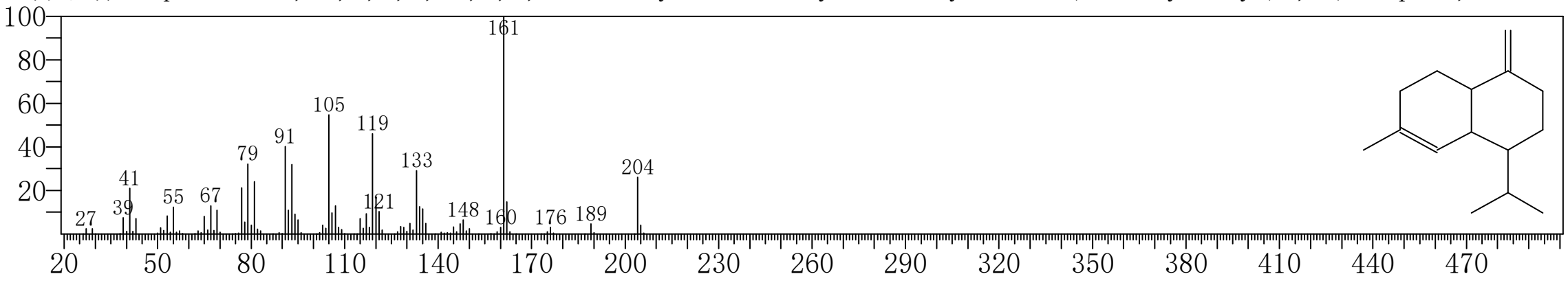

<< 目标组分 >>
行号#:38　保留时间:30.085(扫描数#:5018)　质量峰:274
原始模式:平均 30.080-30.090(5017-5019)　基峰:161.15(7640570)
背景模式:峰校准　组 1 - 事件 1　Scan

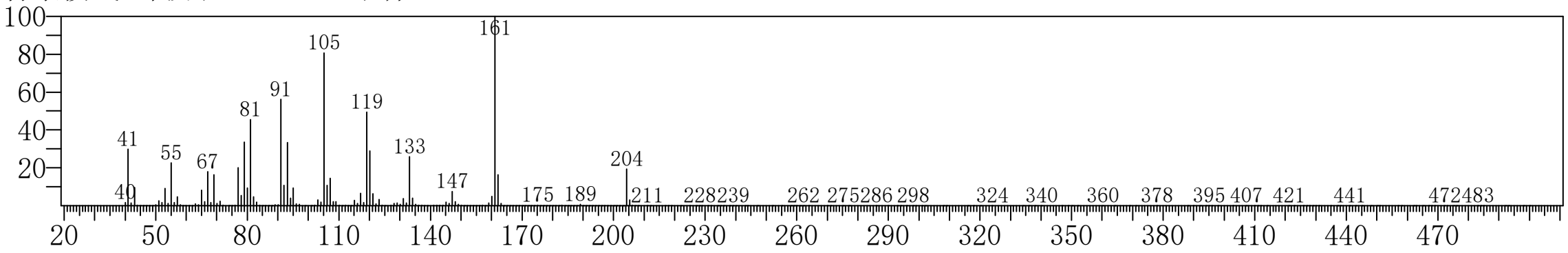

命中#:1　输入:22418　谱库:NIST17s.lib
SI:95　分子式:C15H24　CAS:23986-74-5　摩尔质量:204　保留指数:1515
组分名称:Germacrene D $$ (S,1Z,6Z)-8-Isopropyl-1-methyl-5-methylenecyclodeca-1,6-diene $$ D-Germacrene $$ 1

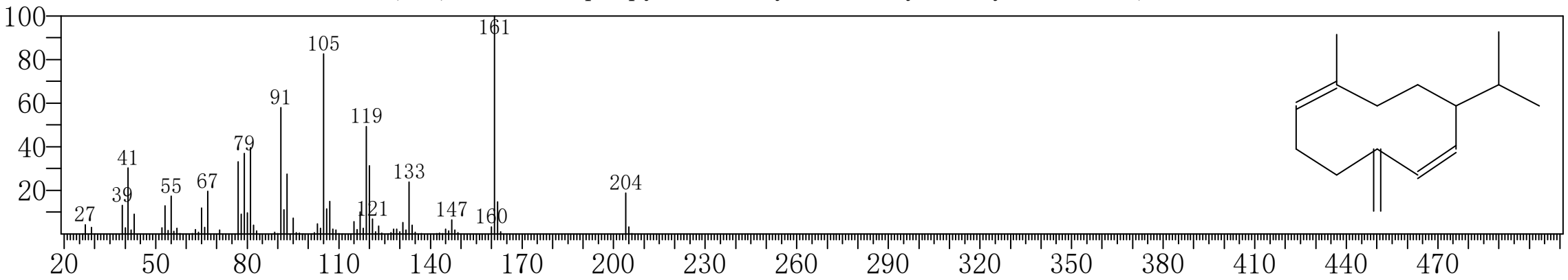

命中#:2　输入:1093　谱库:FFNSC1.3.lib
SI:93　分子式:C15 H24　CAS:38357-83-4　摩尔质量:204　保留指数:1512
组分名称:Cadinene <gamma->

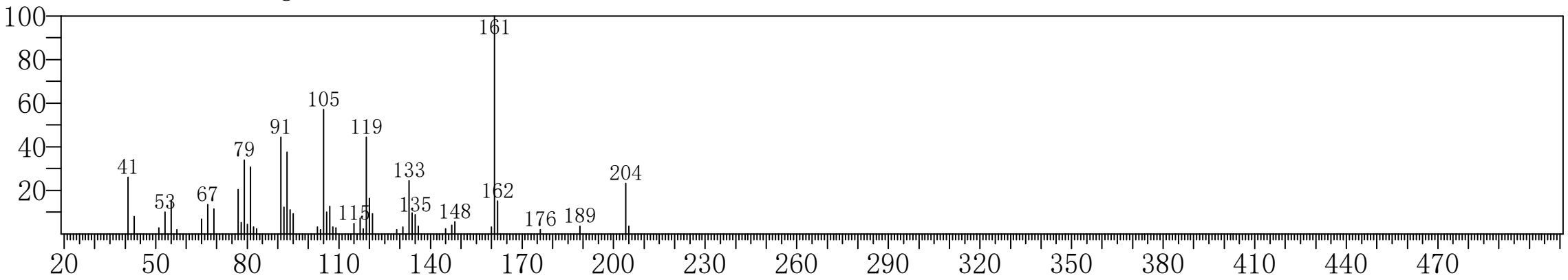

命中#:3　输入:22421　谱库:NIST17s.lib
SI:92　分子式:C15H24　CAS:18252-44-3　摩尔质量:204　保留指数:1216
组分名称:(1R,2S,6S,7S,8S)-8-Isopropyl-1-methyl-3-methylenetricyclo[4.4.0.02,7]decane-rel- $$ Tricyclo[4.4.0.

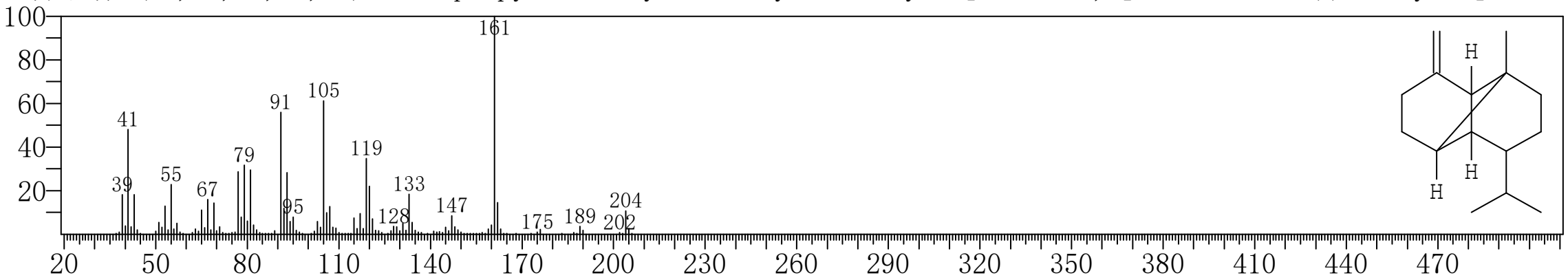

命中#:4　输入:969　谱库:FFNSC1.3.lib
SI:92　分子式:C15 H24　CAS:105453-16-5　摩尔质量:204　保留指数:1480
组分名称:Germacrene D

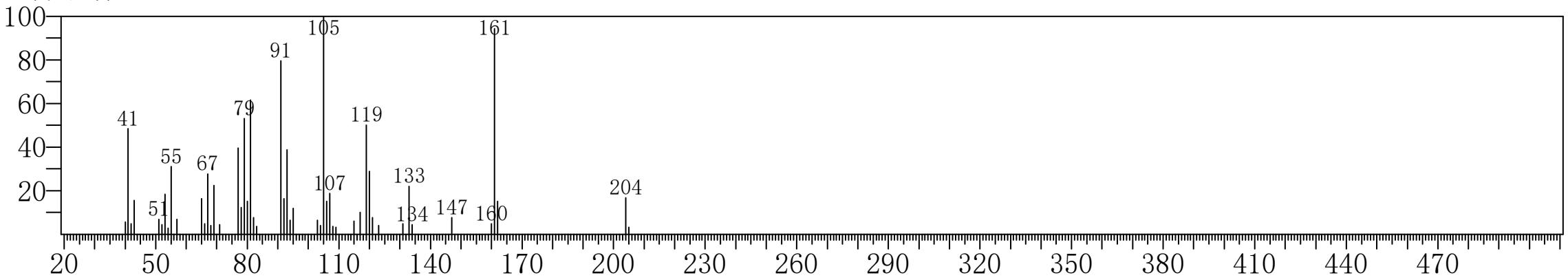

命中#:5　输入:1471　谱库:FFNSC1.3.lib
SI:92　分子式:C15 H24　CAS:13744-15-5　摩尔质量:204　保留指数:1392
组分名称:Cubebene <beta->

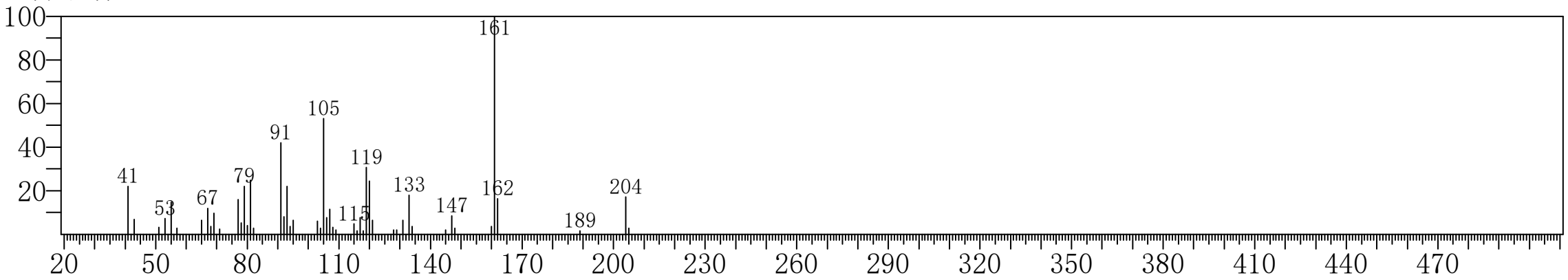

<< 目标组分 >>
行号#:39　保留时间:30.445(扫描数#:5090)　质量峰:192
原始模式:平均 30.440-30.450(5089-5091)　基峰:107.10(13231)
背景模式:峰校准　组 1 - 事件 1　Scan

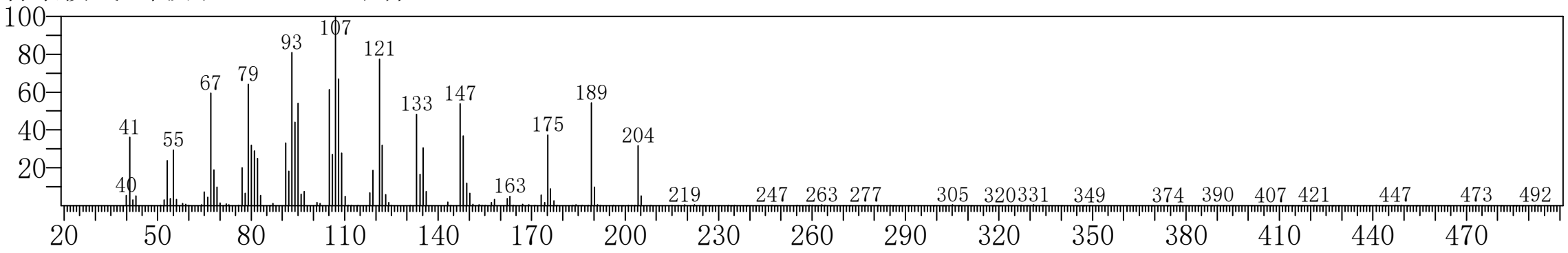

命中#:1　输入:22468　谱库:NIST17s.lib
SI:90　分子式:C15H24　CAS:17066-67-0　摩尔质量:204　保留指数:1469
组分名称:Naphthalene, decahydro-4a-methyl-1-methylene-7-(1-methylethenyl)-, [4aR-(4a.alpha.,7.alpha.,8a.beta

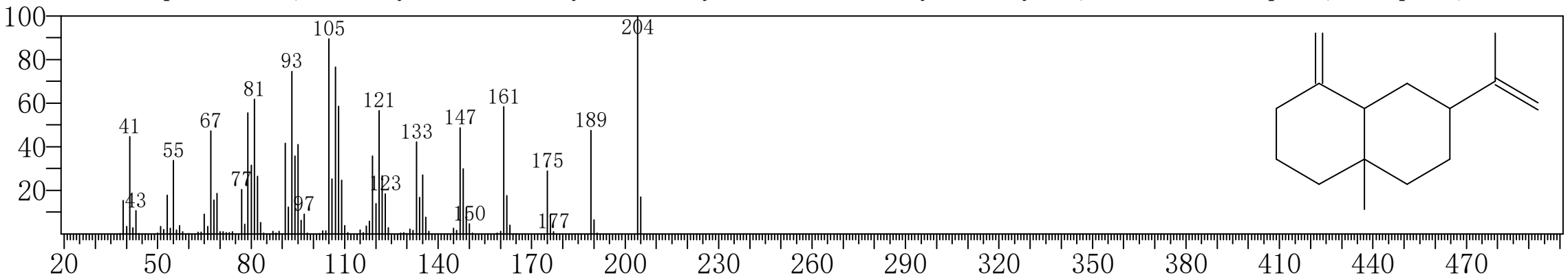

命中#:2　输入:22339　谱库:NIST17s.lib
SI:88　分子式:C15H24　CAS:17066-67-0　摩尔质量:204　保留指数:1469
组分名称:Naphthalene, decahydro-4a-methyl-1-methylene-7-(1-methylethenyl)-, [4aR-(4a.alpha.,7.alpha.,8a.beta

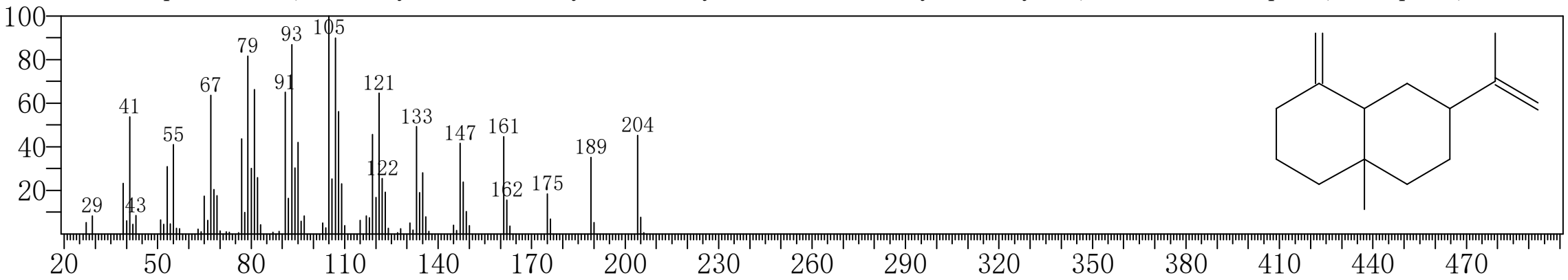

命中#:3　输入:52825　谱库:NIST17-1.lib
SI:88　分子式:C15H24　CAS:116-04-1　摩尔质量:204　保留指数:1574
组分名称:.beta.-Humulene $$ 1,4,4-Trimethyl-8-methylene-1,5-cycloundecadiene # $$

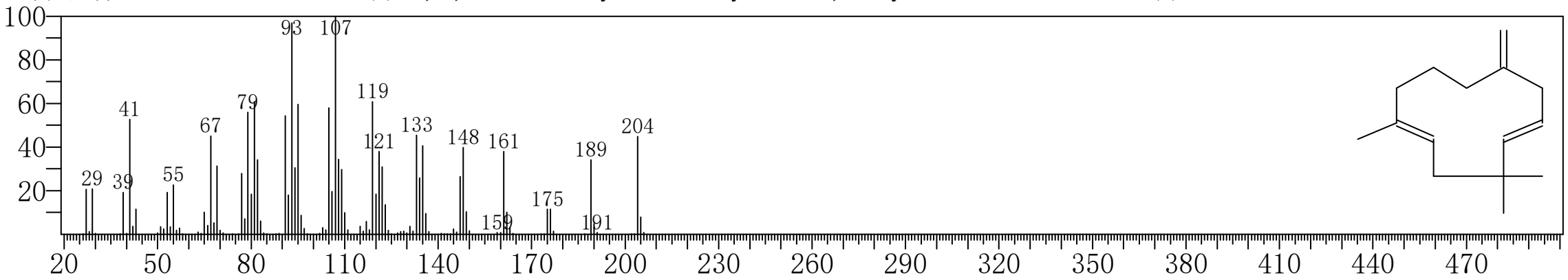

命中#:4　输入:22366　谱库:NIST17s.lib
SI:87　分子式:C15H24　CAS:3691-11-0　摩尔质量:204　保留指数:1490
组分名称:Azulene, 1,2,3,5,6,7,8,8a-octahydro-1,4-dimethyl-7-(1-methylethenyl)-, [1S-(1.alpha.,7.alpha.,8a.be

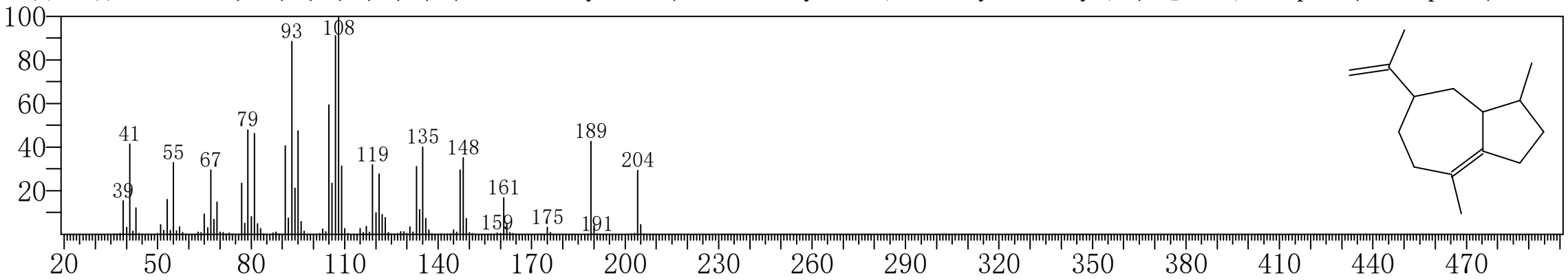

命中#:5　输入:52725　谱库:NIST17-1.lib
SI:87　分子式:C15H24　CAS:17066-67-0　摩尔质量:204　保留指数:1469
组分名称:Naphthalene, decahydro-4a-methyl-1-methylene-7-(1-methylethenyl)-, [4aR-(4a.alpha.,7.alpha.,8a.beta

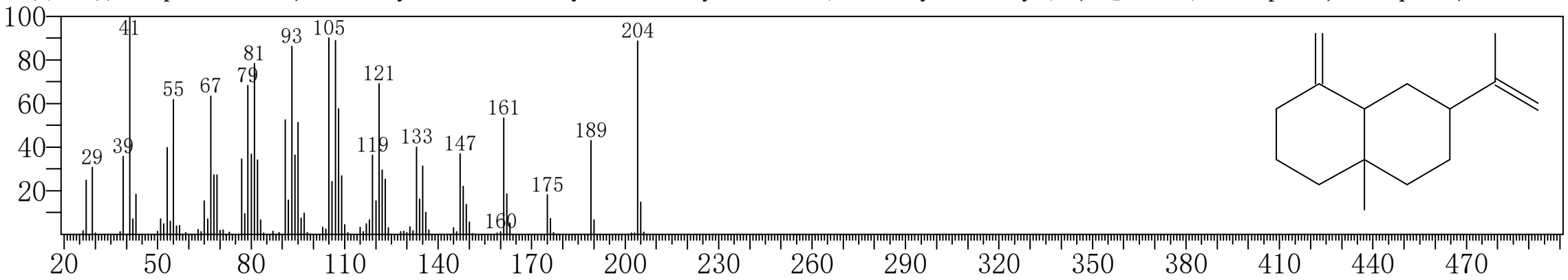

<< 目标组分 >>
行号#:40　保留时间:30.625(扫描数#:5126)　质量峰:254
原始模式:平均 30.620-30.630(5125-5127)　基峰:161.15(67537)
背景模式:峰校准　组 1 - 事件 1　Scan

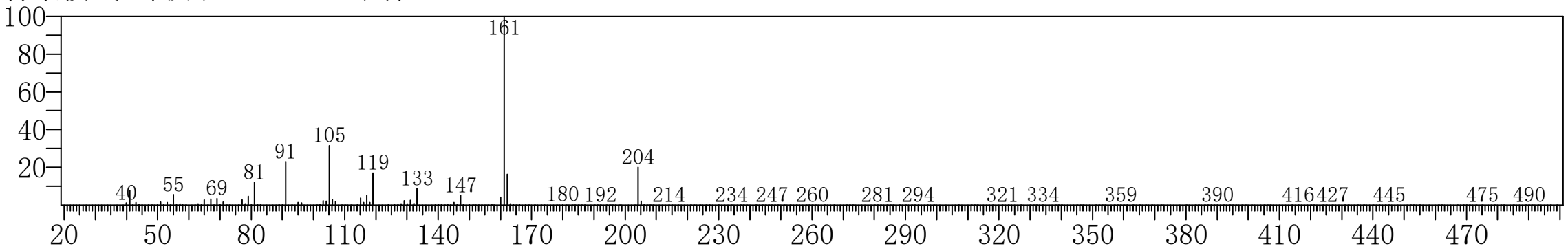

命中#:1　输入:52919　谱库:NIST17-1.lib
SI:91　分子式:C15H24　CAS:157477-72-0　摩尔质量:204　保留指数:1435
组分名称:cis-Muurola-4(15),5-diene $$ (1S,4S,4aR)-1-Isopropyl-4-methyl-7-methylene-1,2,3,4,4a,5,6,7-octahydı

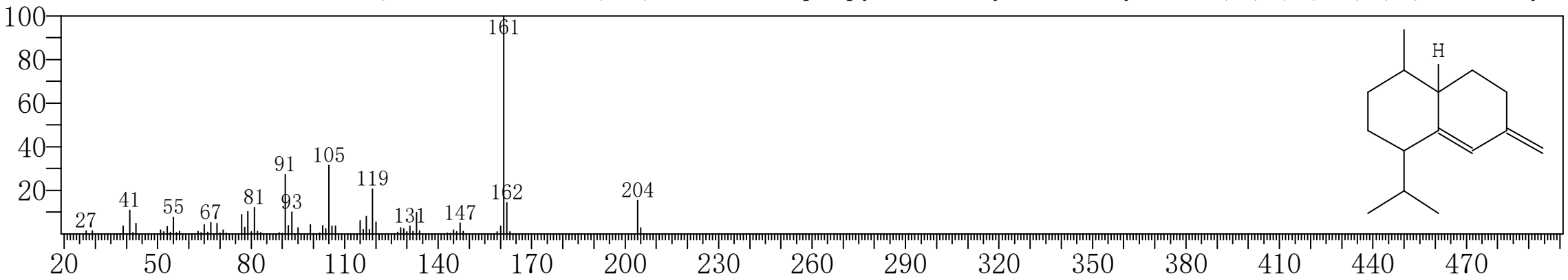

命中#:2　输入:1723　谱库:FFNSC1.3.lib
SI:91　分子式:C15 H24　CAS:157477-72-0　摩尔质量:204　保留指数:1466
组分名称:Muurola-4(14),5-diene <cis->

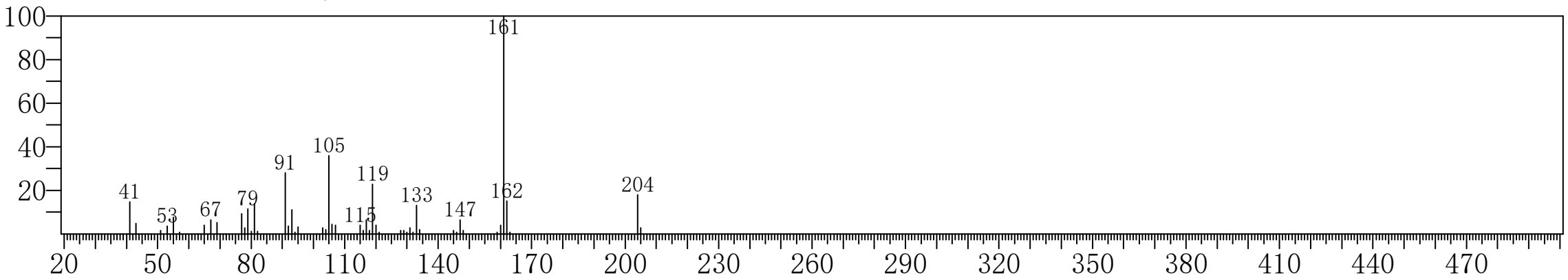

命中#:3　输入:52927　谱库:NIST17-1.lib
SI:90　分子式:C15H24　CAS:54274-73-6　摩尔质量:204　保留指数:1435
组分名称:(+)-epi-Bicyclosesquiphellandrene $$ 1-Isopropyl-4-methyl-7-methylene-1,2,3,4,4a,5,6,7-octahydronaj

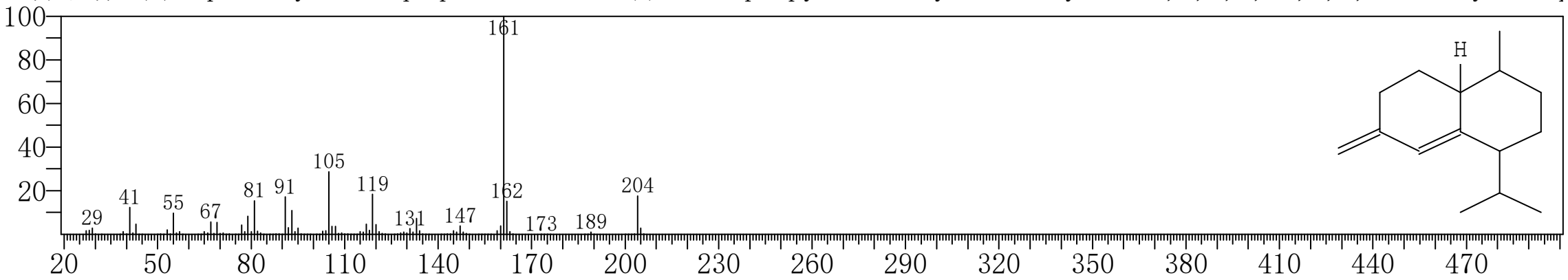

命中#:4　输入:52918　谱库:NIST17-1.lib
SI:90　分子式:C15H24　CAS:54324-03-7　摩尔质量:204　保留指数:1435
组分名称:Bicyclosesquiphellandrene $$ (1S,4R,4aR)-1-Isopropyl-4-methyl-7-methylene-1,2,3,4,4a,5,6,7-octahydı

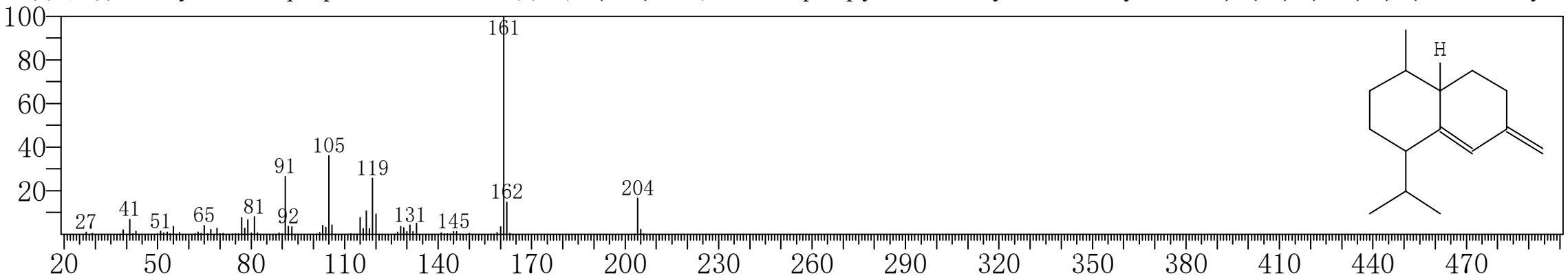

命中#:5　输入:52922　谱库:NIST17-1.lib
SI:87　分子式:C15H24　CAS:267665-20-3　摩尔质量:204　保留指数:1440
组分名称:(1S,4S,4aS)-1-Isopropyl-4,7-dimethyl-1,2,3,4,4a,5-hexahydronaphthalene $$ Naphthalene, 1,2,3,4,4a,!

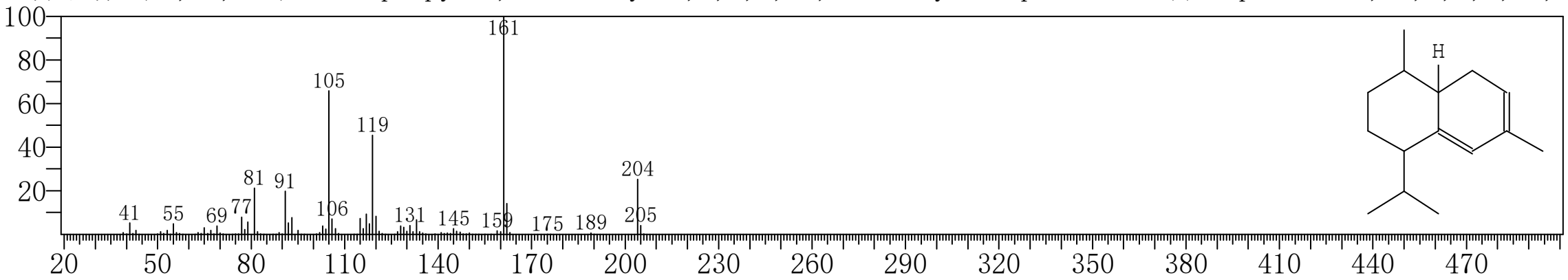

<< 目标组分 >>
行号#:41　保留时间:30.760(扫描数#:5153)　质量峰:251
原始模式:平均 30.755-30.765(5152-5154)　基峰:161.15(107845)
背景模式:峰校准　组 1 - 事件 1　Scan

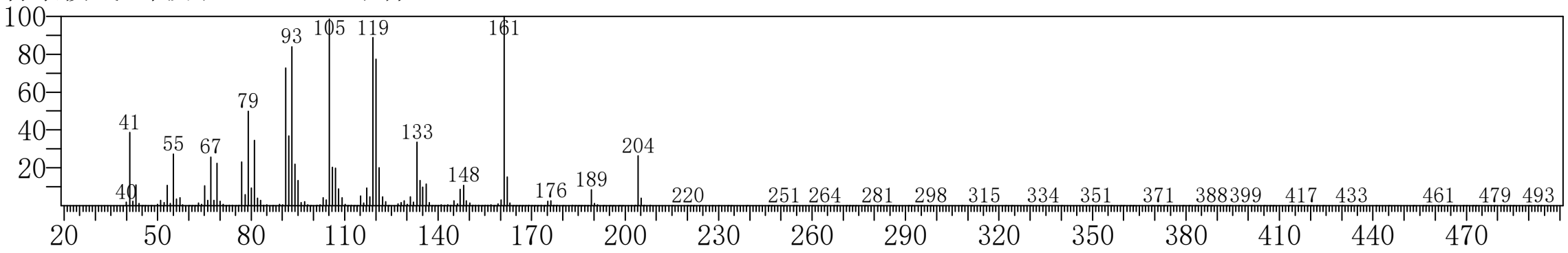

命中#:1　输入:52807　谱库:NIST17-1.lib
SI:92　分子式:C15H24　CAS:14912-44-8　摩尔质量:204　保留指数:1221
组分名称:Ylangene $$ Tricyclo[4.4.0.0(2,7)]dec-3-ene, 8-isopropyl-1,3-dimethyl-, (1S,2R,6R,7R,8S)-(+)- $$ (

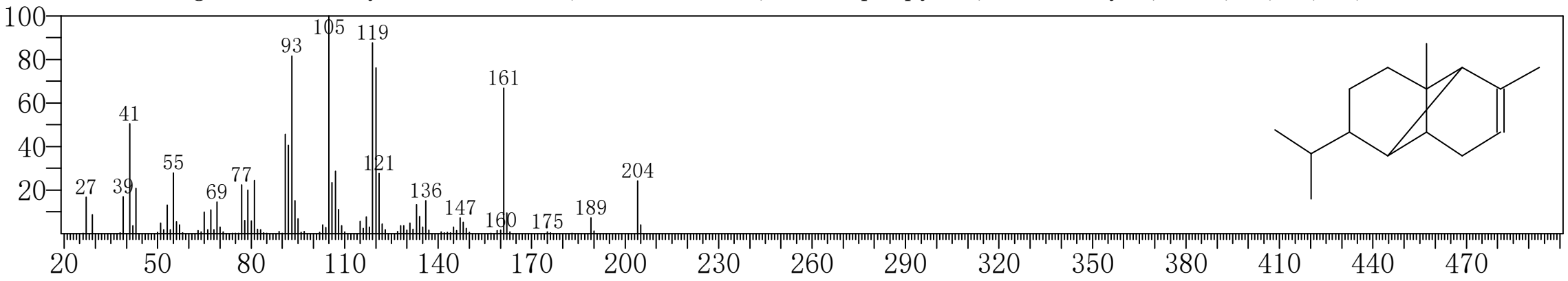

命中#:2　输入:22335　谱库:NIST17s.lib
SI:92　分子式:C15H24　CAS:14912-44-8　摩尔质量:204　保留指数:1221
组分名称:Ylangene $$ Tricyclo[4.4.0.0(2,7)]dec-3-ene, 8-isopropyl-1,3-dimethyl-, (1S,2R,6R,7R,8S)-(+)- $$ (

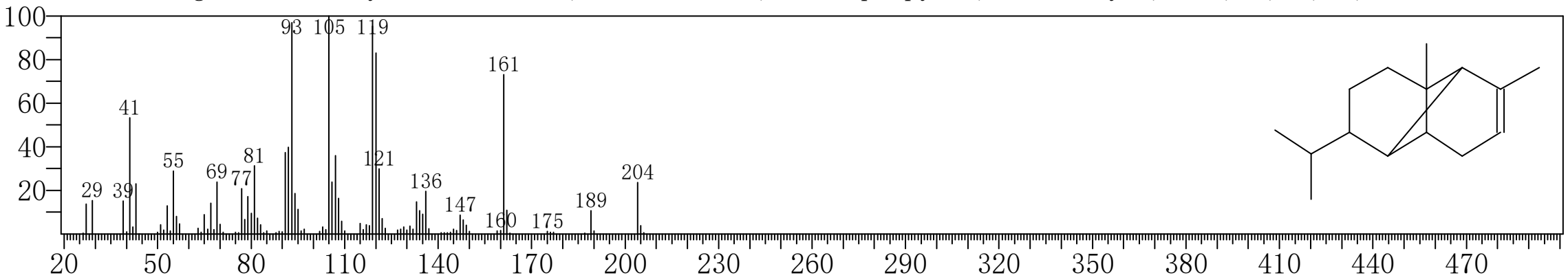

命中#:3　输入:1678　谱库:FFNSC1.3.lib
SI:91　分子式:C15 H24　CAS:54932-90-0　摩尔质量:204　保留指数:1502
组分名称:Amorphene <epsylon->

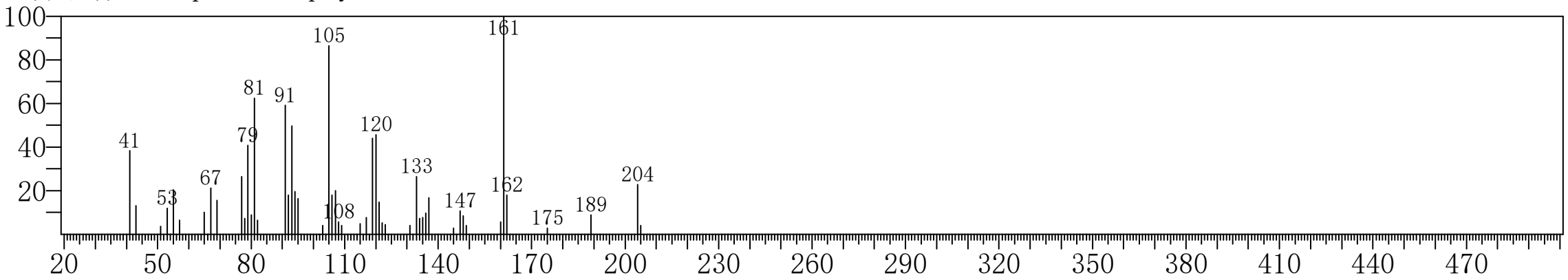

命中#:4　输入:22406　谱库:NIST17s.lib
SI:91　分子式:C15H24　CAS:30021-74-0　摩尔质量:204　保留指数:1435
组分名称:.gamma.-Muurolene $$ Naphthalene, 1,2,3,4,4a,5,6,8a-octahydro-7-methyl-4-methylene-1-(1-methylethy

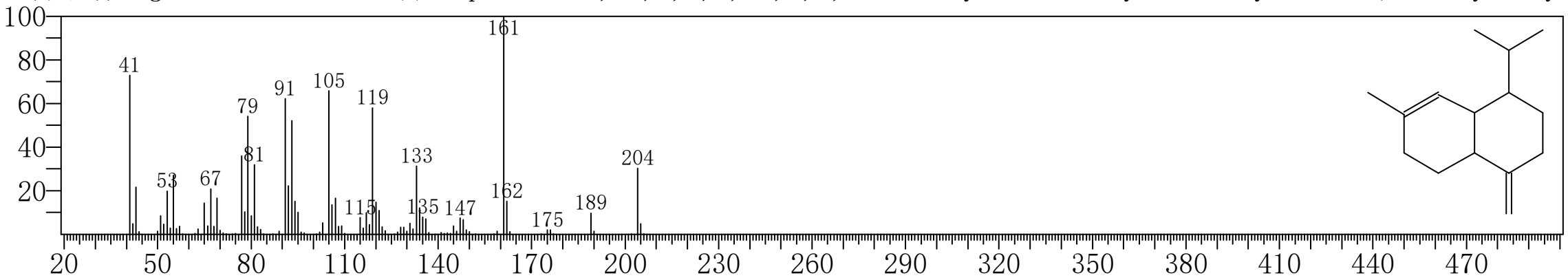

命中#:5　输入:22432　谱库:NIST17s.lib
SI:91　分子式:C15H24　CAS:30021-74-0　摩尔质量:204　保留指数:1435
组分名称:.gamma.-Muurolene $$ Naphthalene, 1,2,3,4,4a,5,6,8a-octahydro-7-methyl-4-methylene-1-(1-methylethy

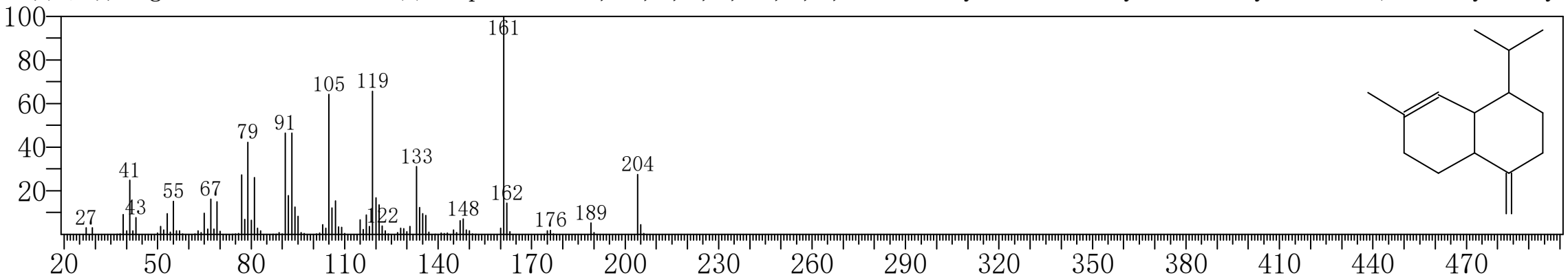

<< 目标组分 >>
行号#:42　保留时间:30.925(扫描数#:5186)　质量峰:253
原始模式:平均 30.920-30.930(5185-5187)　基峰:93.10(26607)
背景模式:峰校准　组 1 - 事件 1　Scan

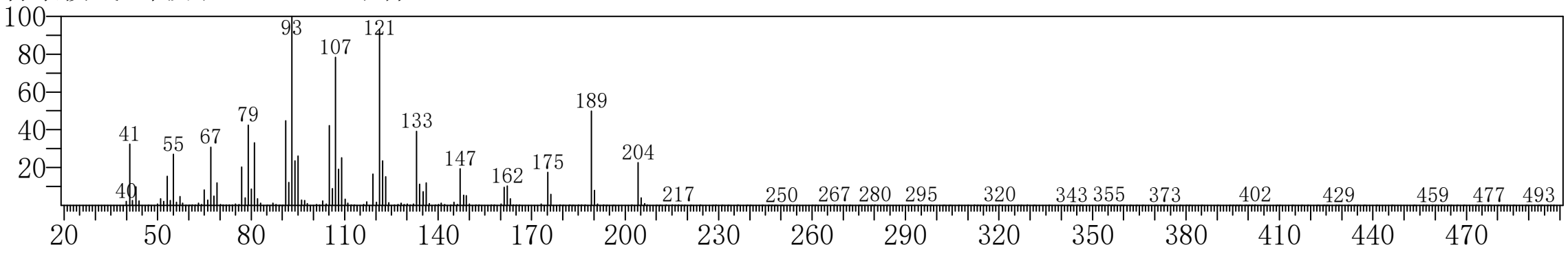

命中#:1　输入:52961　谱库:NIST17-1.lib
SI:88　分子式:C15H24　CAS:207297-57-2　摩尔质量:204　保留指数:1474
组分名称:2-Isopropenyl-4a,8-dimethyl-1,2,3,4,4a,5,6,8a-octahydronaphthalene

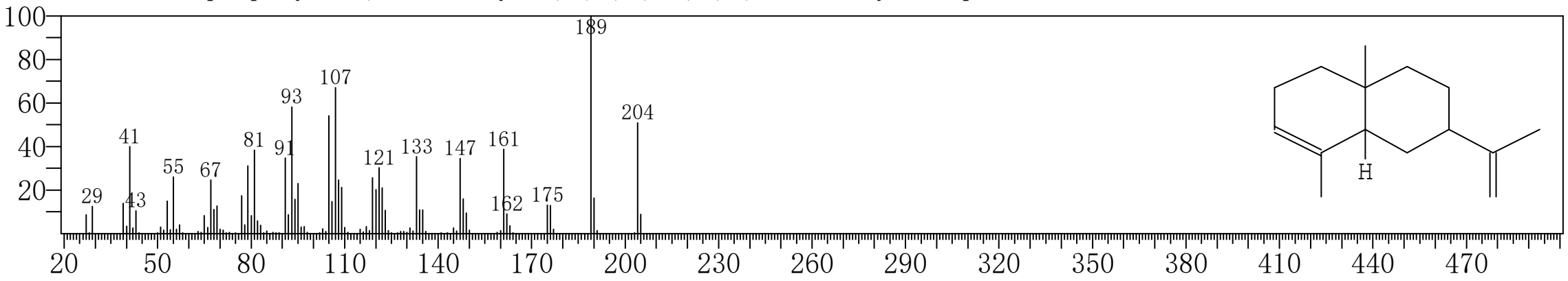

命中#:2　输入:52823　谱库:NIST17-1.lib
SI:88　分子式:C15H24　CAS:0-00-0　摩尔质量:204　保留指数:1480
组分名称:1-Cycloheptene, 1,4-dimethyl-3-(2-methyl-1-propene-1-yl)-4-vinyl- $$ 1,4-Dimethyl-3-(2-methyl-1-pro

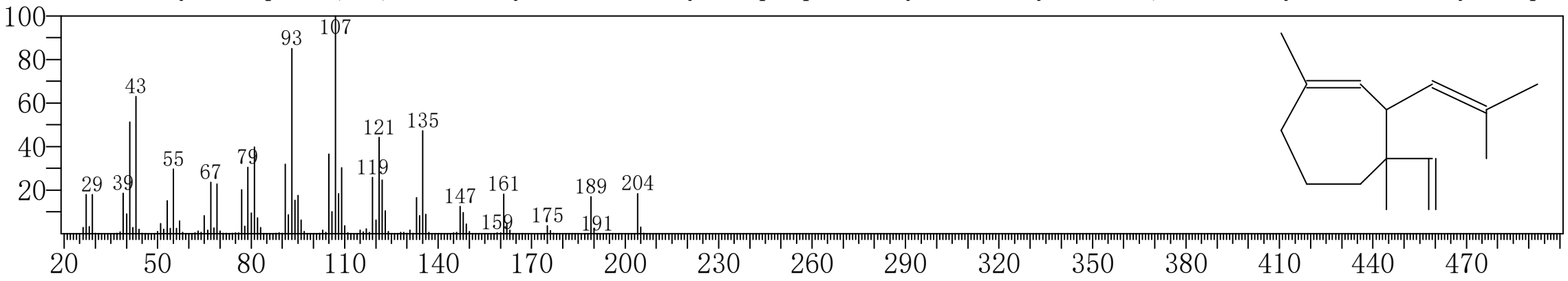

命中#:3　输入:22366　谱库:NIST17s.lib
SI:88　分子式:C15H24　CAS:3691-11-0　摩尔质量:204　保留指数:1490
组分名称:Azulene, 1,2,3,5,6,7,8,8a-octahydro-1,4-dimethyl-7-(1-methylethenyl)-, [1S-(1.alpha.,7.alpha.,8a.be

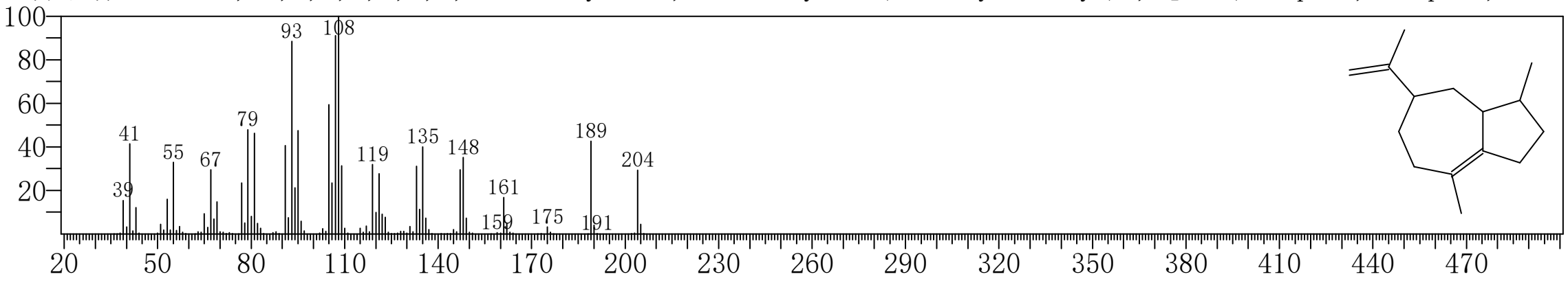

命中#:4　输入:1110　谱库:FFNSC1.3.lib
SI:88　分子式:C15 H24　CAS:67650-90-2　摩尔质量:204　保留指数:1497
组分名称:Bicyclogermacrene

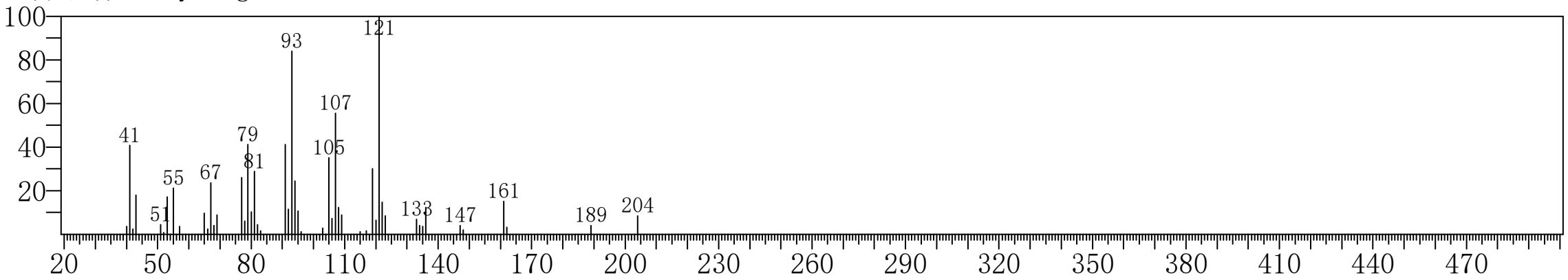

命中#:5　输入:52772　谱库:NIST17-1.lib
SI:88　分子式:C15H24　CAS:142878-08-8　摩尔质量:204　保留指数:1474
组分名称:(3S,3aS,8aR)-6,8a-Dimethyl-3-(prop-1-en-2-yl)-1,2,3,3a,4,5,8,8a-octahydroazulene $$ Azulene, 1,2,3,

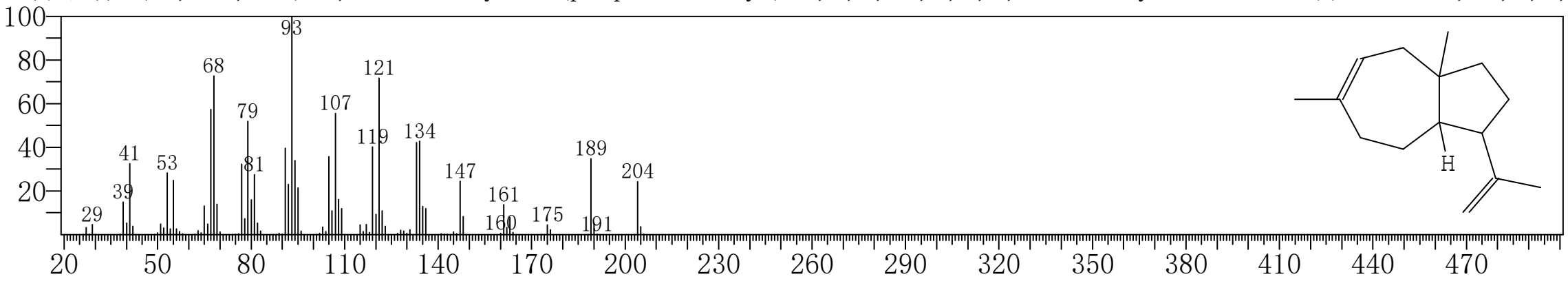

<< 目标组分 >>
行号#:43　 保留时间:31.210(扫描数#:5243)　质量峰:271
原始模式:平均 31.205-31.215(5242-5244)　 基峰:105.10(595302)
背景模式:峰校准　组 1 - 事件 1　Scan

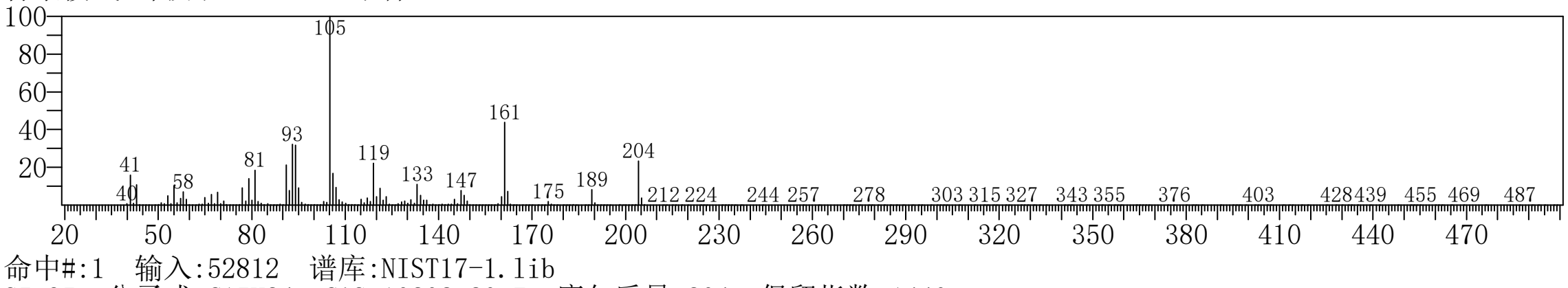

命中#:1　输入:52812　谱库:NIST17-1.lib
SI:95　 分子式:C15H24　CAS:10208-80-7　摩尔质量:204　保留指数:1440
组分名称:.alpha.-Muurolene $$ Naphthalene, 1,2,4a,5,6,8a-hexahydro-4,7-dimethyl-1-(1-methylethyl)-, (1S,4aS,

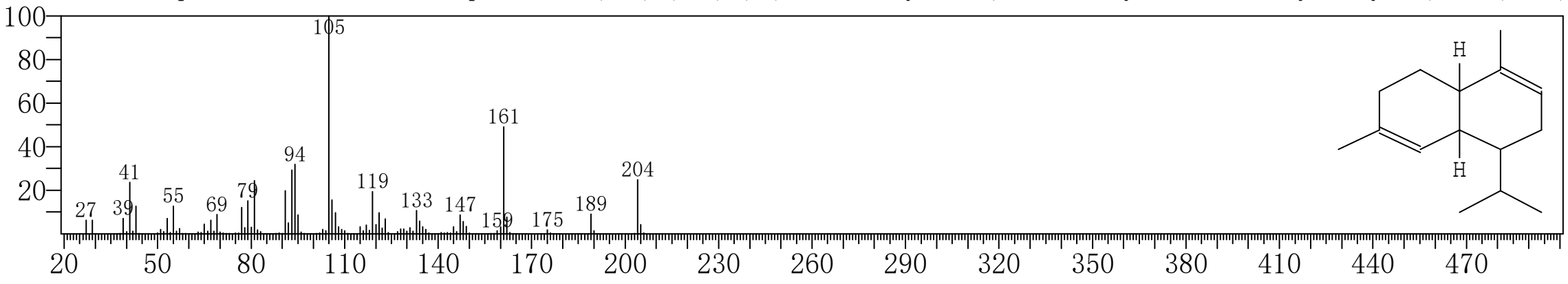

命中#:2　输入:52813　谱库:NIST17-1.lib
SI:95　 分子式:C15H24　CAS:483-75-0　摩尔质量:204　保留指数:1440
组分名称:Naphthalene, 1,2,4a,5,6,8a-hexahydro-4,7-dimethyl-1-(1-methylethyl)- $$ 1-Isopropyl-4,7-dimethyl-1,

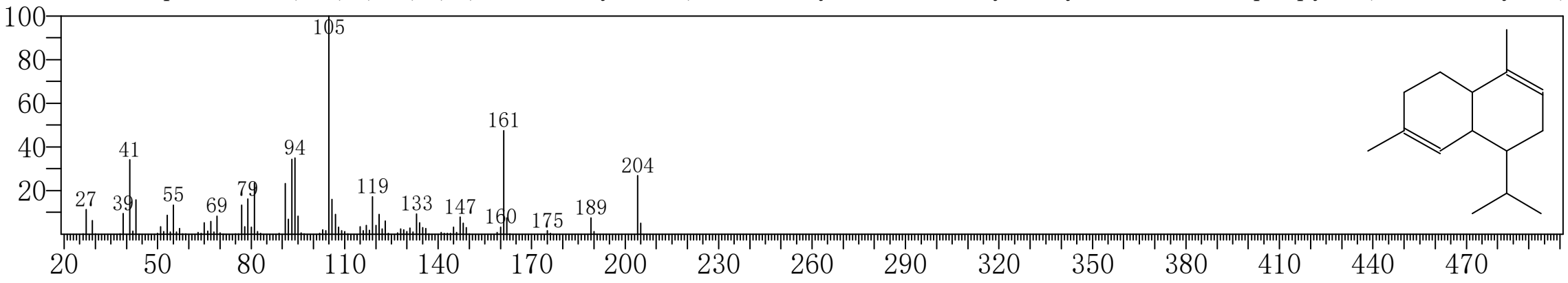

命中#:3　输入:1052　谱库:FFNSC1.3.lib
SI:94　 分子式:C15 H24　CAS:10208-80-7　摩尔质量:204　保留指数:1497
组分名称:Muurolene <alpha->

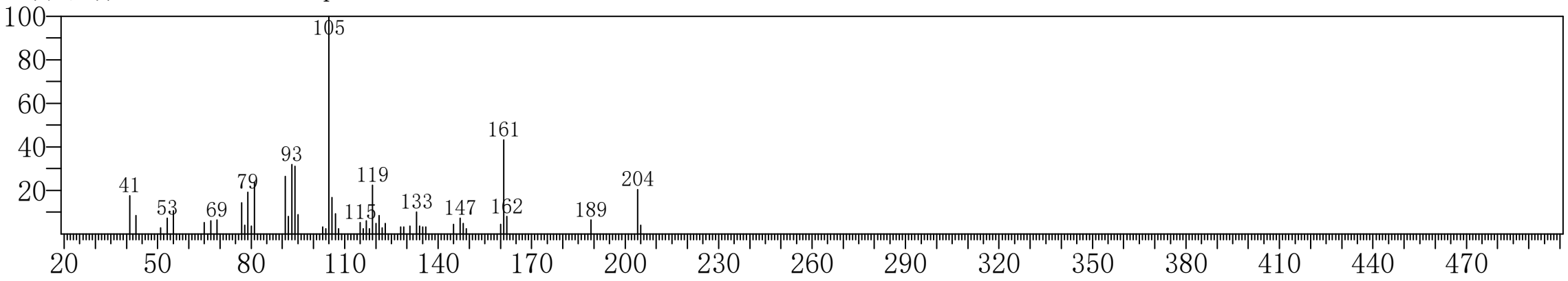

命中#:4　输入:22348　谱库:NIST17s.lib
SI:94　 分子式:C15H24　CAS:31983-22-9　摩尔质量:204　保留指数:1440
组分名称:.alpha.-Muurolene $$ [1.alpha.,4a.alpha.,8a.alpha.]-1,2,4a,5,6,8a-hexahydro-4-7-dimethyl-1-[1-meth

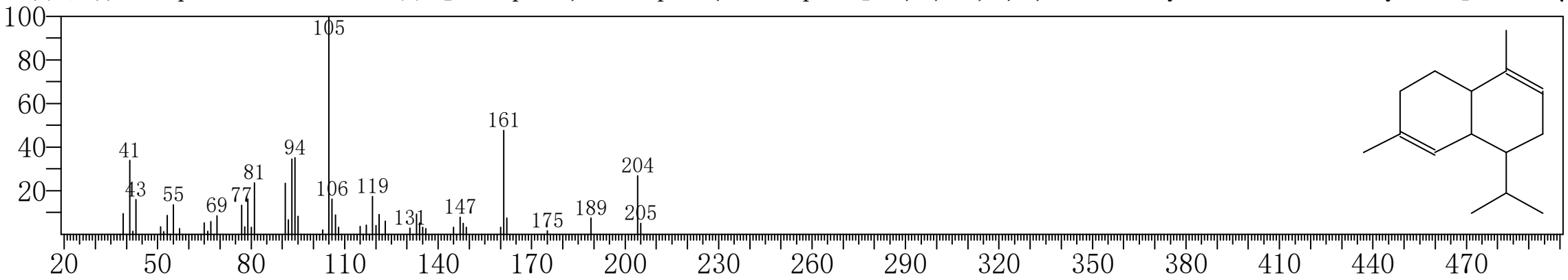

命中#:5　输入:22347　谱库:NIST17s.lib
SI:94　 分子式:C15H24　CAS:10208-80-7　摩尔质量:204　保留指数:1440
组分名称:.alpha.-Muurolene $$ Naphthalene, 1,2,4a,5,6,8a-hexahydro-4,7-dimethyl-1-(1-methylethyl)-, (1S,4aS,

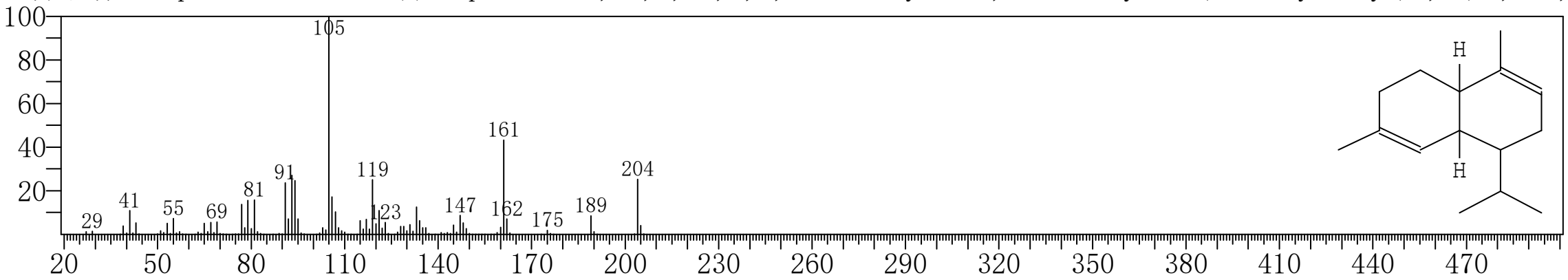

<< 目标组分 >>
行号#:44　 保留时间:31.485(扫描数#:5298)　质量峰:285
原始模式:平均 31.480-31.490(5297-5299)　 基峰:161.15(63567)
背景模式:峰校准　组 1 - 事件 1　Scan

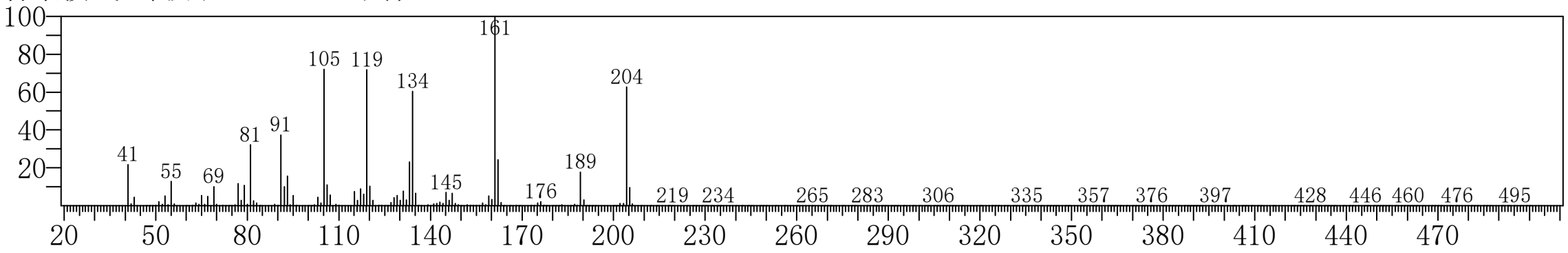

命中#:1　输入:838　谱库:FFNSC1.3.lib
SI:96　分子式:C15 H24　CAS:16729-01-4　摩尔质量:204　保留指数:1518
组分名称:Cadinene <delta->

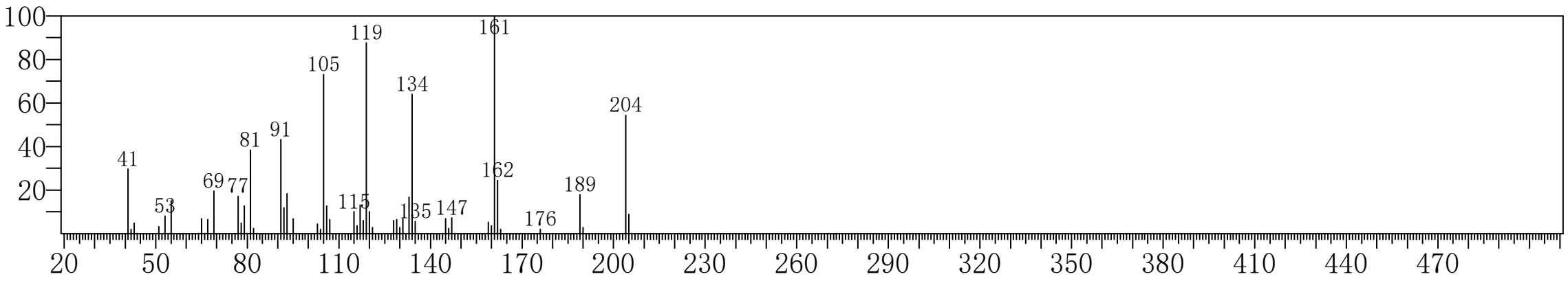

命中#:2　输入:22452　谱库:NIST17s.lib
SI:92　分子式:C15H24　CAS:523-47-7　摩尔质量:204　保留指数:1440
组分名称:Naphthalene, 1,2,4a,5,8,8a-hexahydro-4,7-dimethyl-1-(1-methylethyl)-, [1S-(1.alpha.,4a.beta.,8a.al

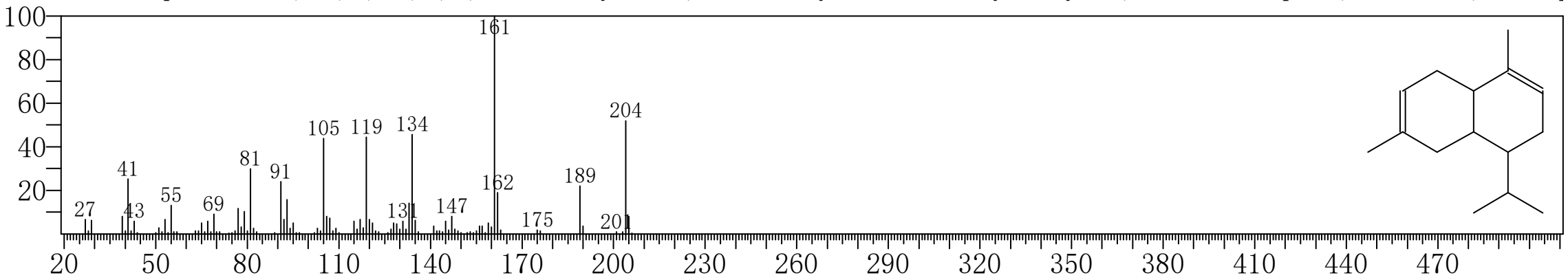

命中#:3　输入:52941　谱库:NIST17-1.lib
SI:92　分子式:C15H24　CAS:483-76-1　摩尔质量:204　保留指数:1469
组分名称:Naphthalene, 1,2,3,5,6,8a-hexahydro-4,7-dimethyl-1-(1-methylethyl)-, (1S-cis)- $$ Cadina-1(10),4-d

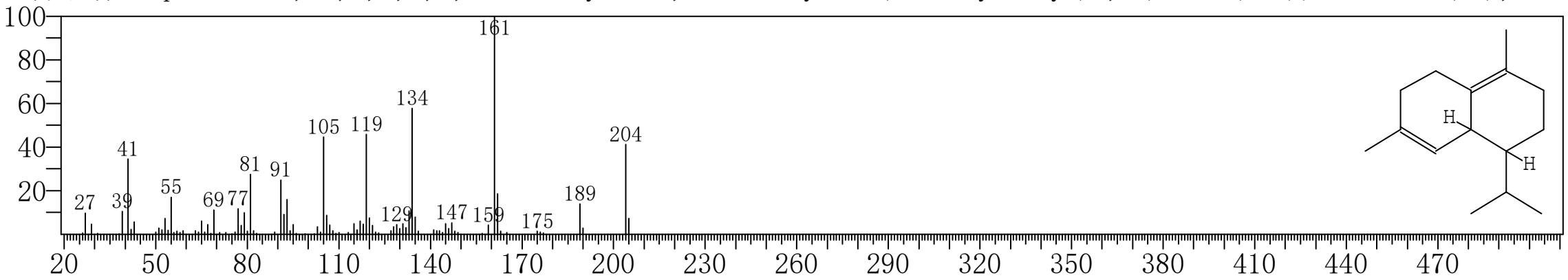

命中#:4　输入:22438　谱库:NIST17s.lib
SI:92　分子式:C15H24　CAS:483-76-1　摩尔质量:204　保留指数:1469
组分名称:Naphthalene, 1,2,3,5,6,8a-hexahydro-4,7-dimethyl-1-(1-methylethyl)-, (1S-cis)- $$ Cadina-1(10),4-d

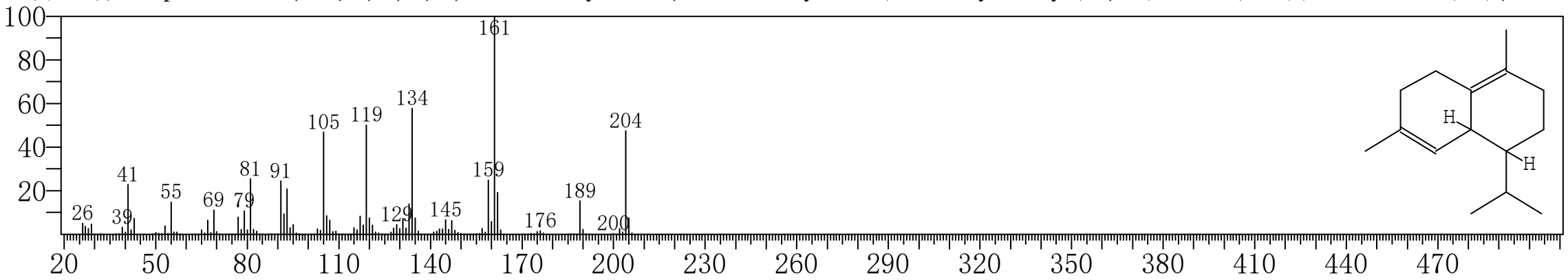

命中#:5　输入:1077　谱库:FFNSC1.3.lib
SI:91　分子式:C15 H24　CAS:16729-00-3　摩尔质量:204　保留指数:1472
组分名称:Cadina-1(6),4-diene

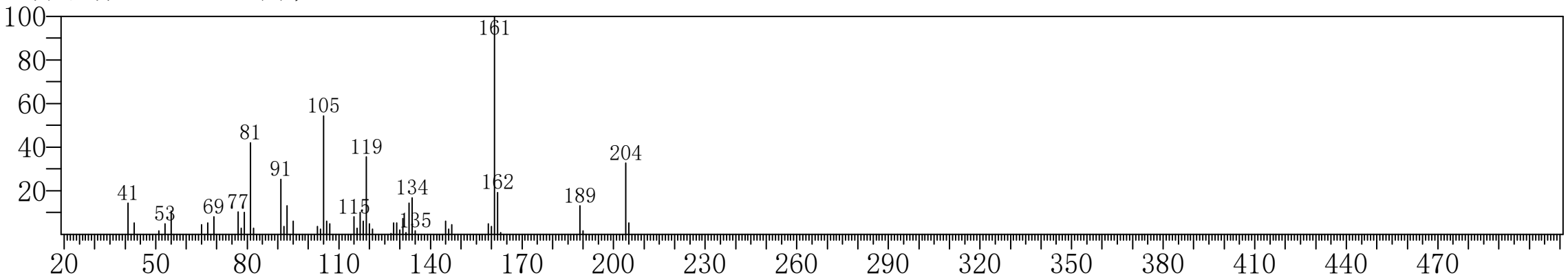

<< 目标组分 >>
行号#:45　 保留时间:32.135(扫描数#:5428)　质量峰:306
原始模式:平均 32.130-32.140(5427-5429)　 基峰:161.15(568392)
背景模式:峰校准　组 1 - 事件 1　Scan

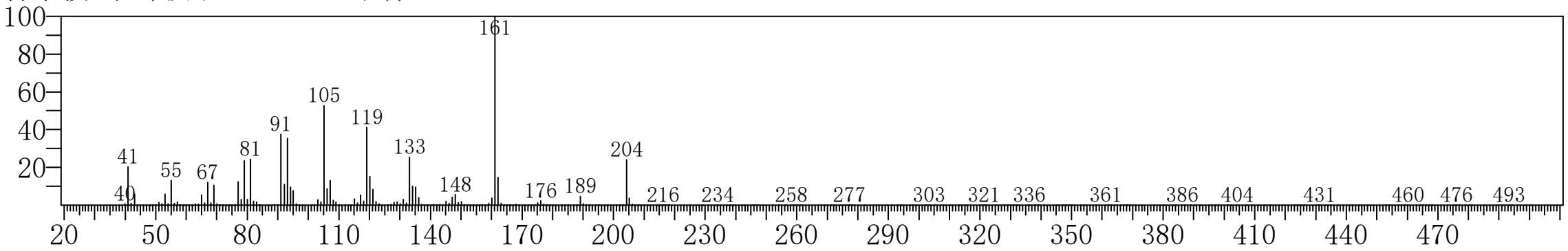

命中#:1　输入:1093　谱库:FFNSC1.3.lib
SI:96　分子式:C15 H24　CAS:38357-83-4　摩尔质量:204　保留指数:1512
组分名称:Cadinene <gamma->

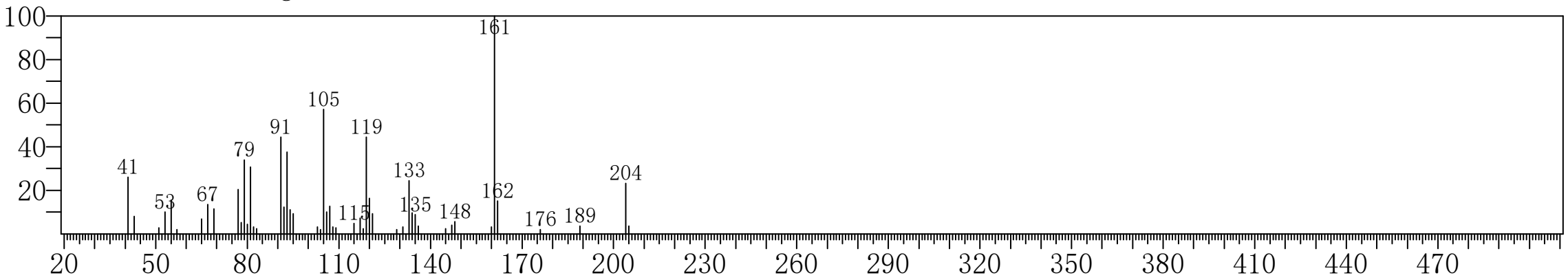

命中#:2　输入:22428　谱库:NIST17s.lib
SI:96　分子式:C15H24　CAS:39029-41-9　摩尔质量:204　保留指数:1435
组分名称:Naphthalene, 1,2,3,4,4a,5,6,8a-octahydro-7-methyl-4-methylene-1-(1-methylethyl)-, (1.alpha.,4a.beta

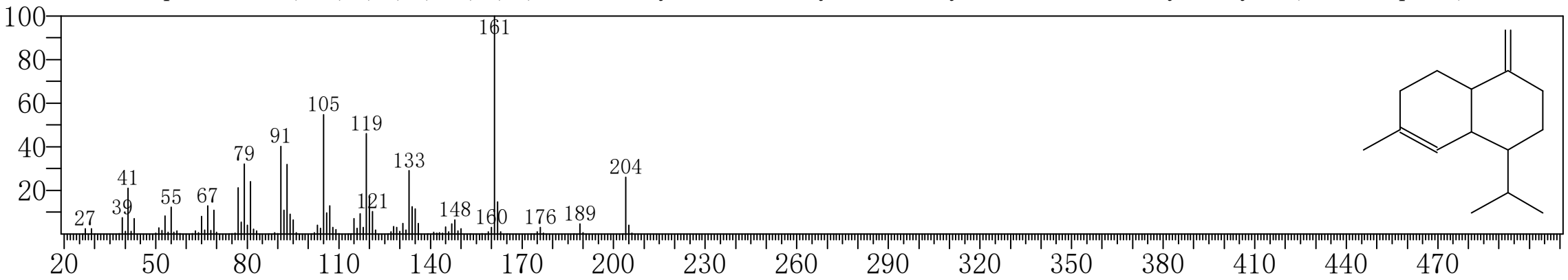

命中#:3　输入:22423　谱库:NIST17s.lib
SI:95　分子式:C15H24　CAS:39029-41-9　摩尔质量:204　保留指数:1435
组分名称:Naphthalene, 1,2,3,4,4a,5,6,8a-octahydro-7-methyl-4-methylene-1-(1-methylethyl)-, (1.alpha.,4a.beta

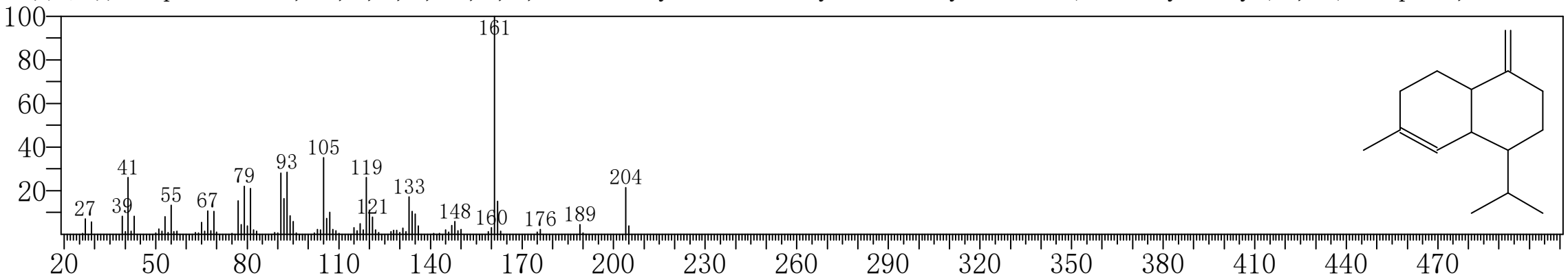

命中#:4　输入:52924　谱库:NIST17-1.lib
SI:94　分子式:C15H24　CAS:30021-74-0　摩尔质量:204　保留指数:1435
组分名称:.gamma.-Muurolene $$ Naphthalene, 1,2,3,4,4a,5,6,8a-octahydro-7-methyl-4-methylene-1-(1-methylethyl

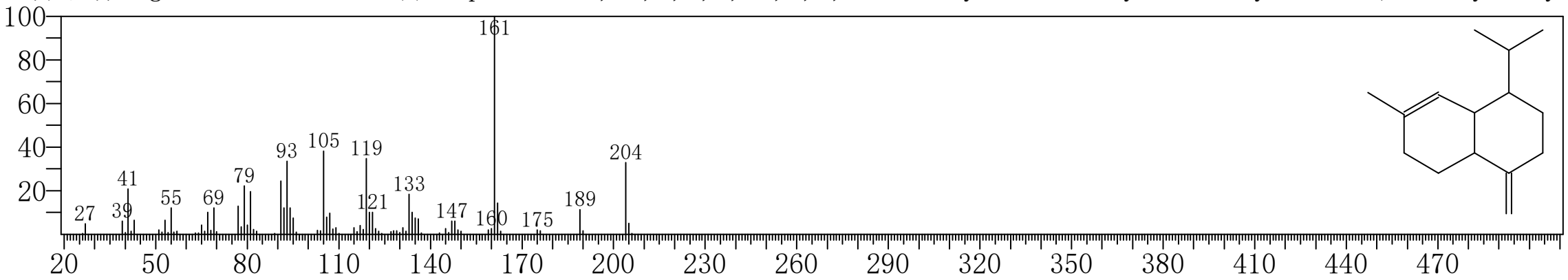

命中#:5　输入:1474　谱库:FFNSC1.3.lib
SI:94　分子式:C15 H24　CAS:30021-74-0　摩尔质量:204　保留指数:1478
组分名称:Muurolene <gamma->

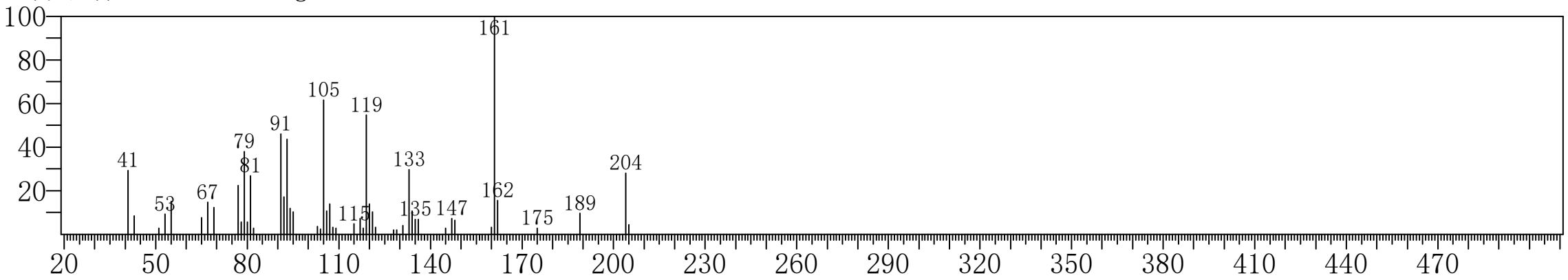

<< 目标组分 >>
行号#:46　保留时间:32.590(扫描数#:5519)　质量峰:318
原始模式:平均 32.585-32.595(5518-5520)　基峰:161.15(1331348)
背景模式:峰校准　组 1 - 事件 1　Scan

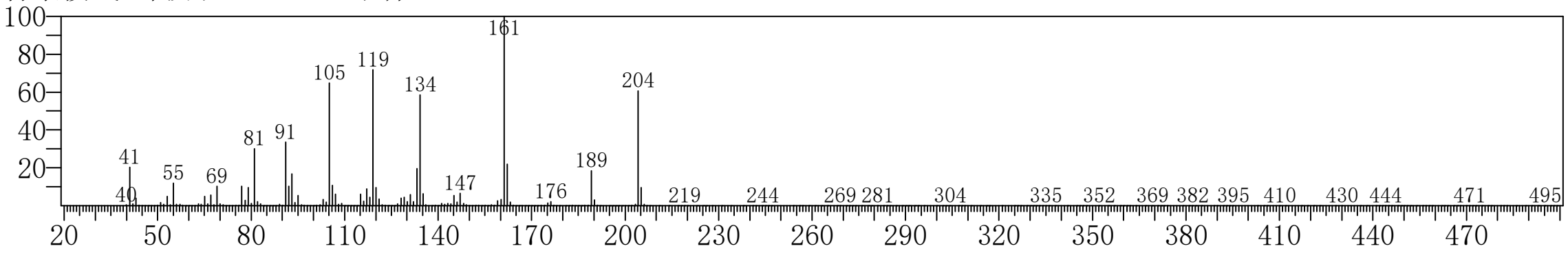

命中#:1　输入:838　谱库:FFNSC1.3.lib
SI:95　分子式:C15 H24　CAS:16729-01-4　摩尔质量:204　保留指数:1518
组分名称:Cadinene <delta->

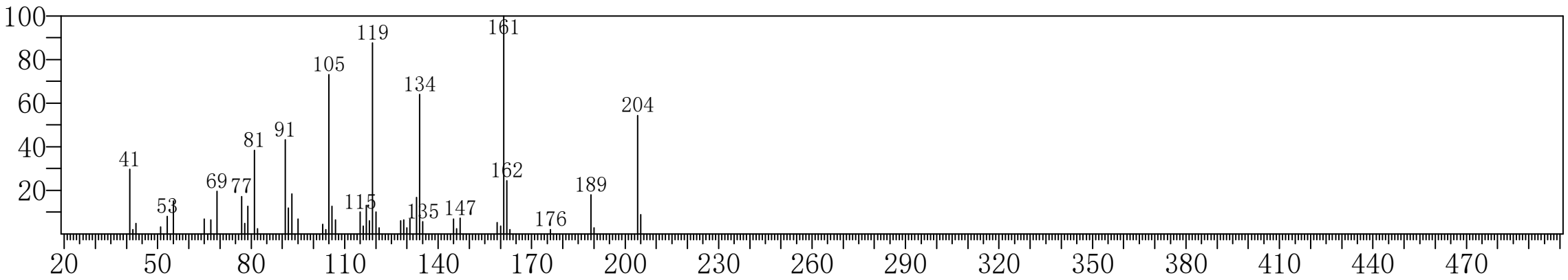

命中#:2　输入:52941　谱库:NIST17-1.lib
SI:93　分子式:C15H24　CAS:483-76-1　摩尔质量:204　保留指数:1469
组分名称:Naphthalene, 1,2,3,5,6,8a-hexahydro-4,7-dimethyl-1-(1-methylethyl)-, (1S-cis)- $$ Cadina-1(10),4-di

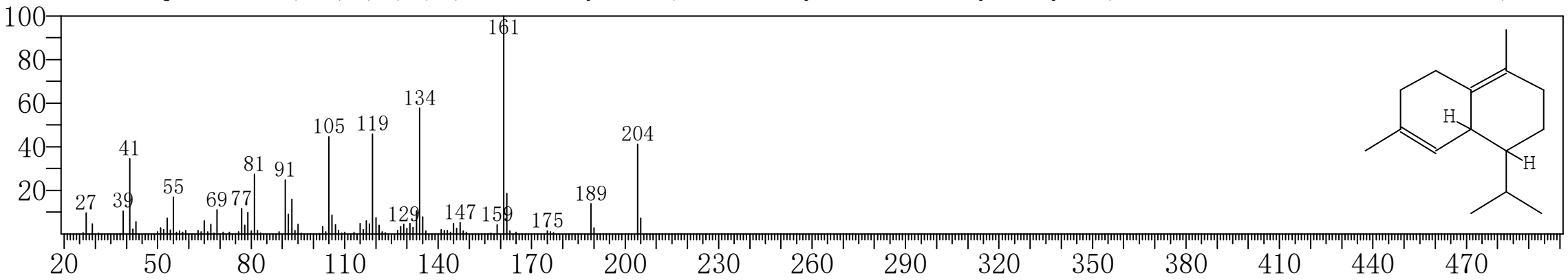

命中#:3　输入:22452　谱库:NIST17s.lib
SI:93　分子式:C15H24　CAS:523-47-7　摩尔质量:204　保留指数:1440
组分名称:Naphthalene, 1,2,4a,5,8,8a-hexahydro-4,7-dimethyl-1-(1-methylethyl)-, [1S-(1.alpha.,4a.beta.,8a.al

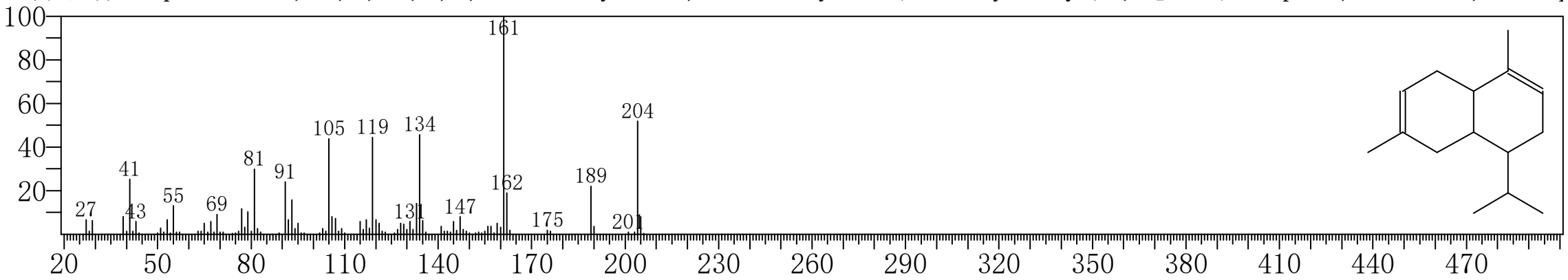

命中#:4　输入:22438　谱库:NIST17s.lib
SI:92　分子式:C15H24　CAS:483-76-1　摩尔质量:204　保留指数:1469
组分名称:Naphthalene, 1,2,3,5,6,8a-hexahydro-4,7-dimethyl-1-(1-methylethyl)-, (1S-cis)- $$ Cadina-1(10),4-di

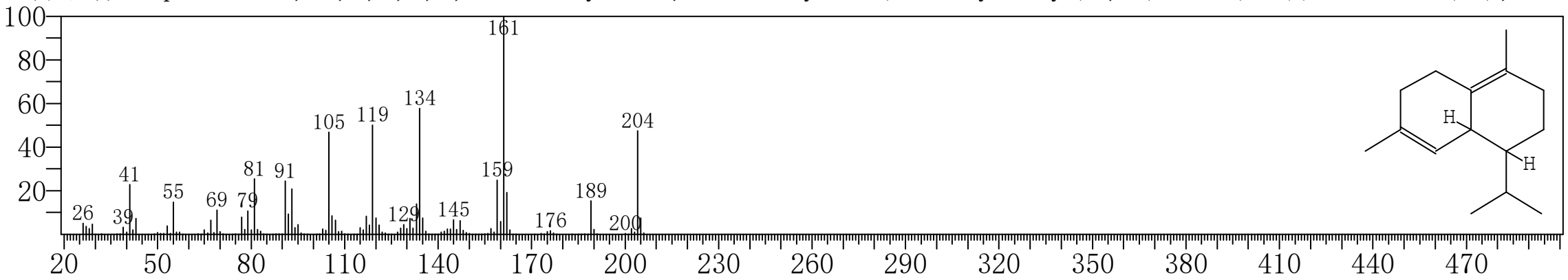

命中#:5　输入:1077　谱库:FFNSC1.3.lib
SI:91　分子式:C15 H24　CAS:16729-00-3　摩尔质量:204　保留指数:1472
组分名称:Cadina-1(6),4-diene

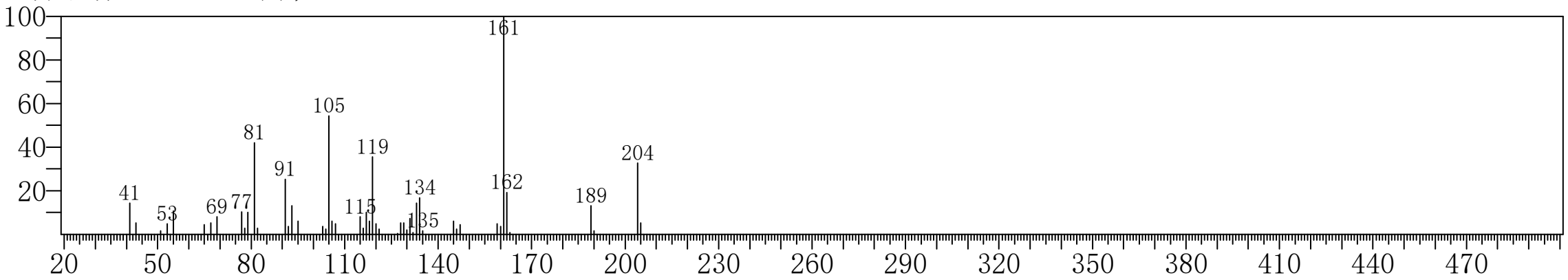

<< 目标组分 >>
行号#:47　 保留时间:33.495(扫描数#:5700)　质量峰:252
原始模式:平均 33.490-33.500(5699-5701)　 基峰:119.10(63594)
背景模式:峰校准　组 1 - 事件 1　Scan

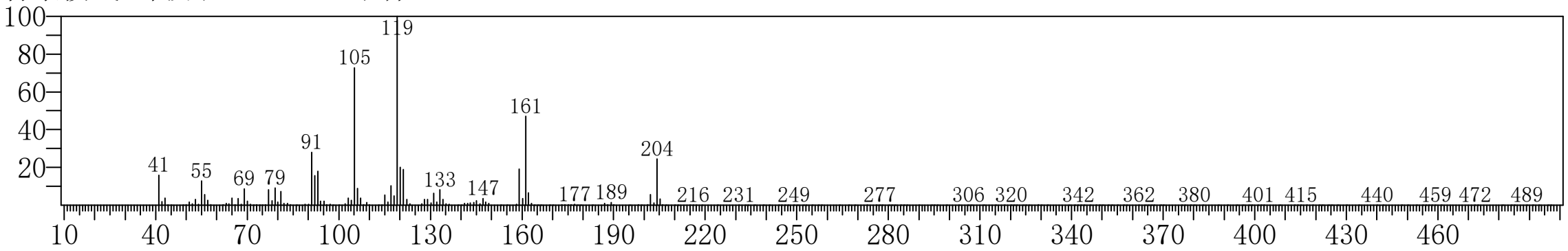

命中#:1　输入:1475　谱库:FFNSC1.3.lib
SI:96　分子式:C15 H24　CAS:38758-02-0　摩尔质量:204　保留指数:1536
组分名称:Naphthalene <1,2,3,4,4a,7-hexahydro-, 1,6-dimethyl-, 4-(1-methylethyl)-, (1alpha,4beta,4abeta)-(?-

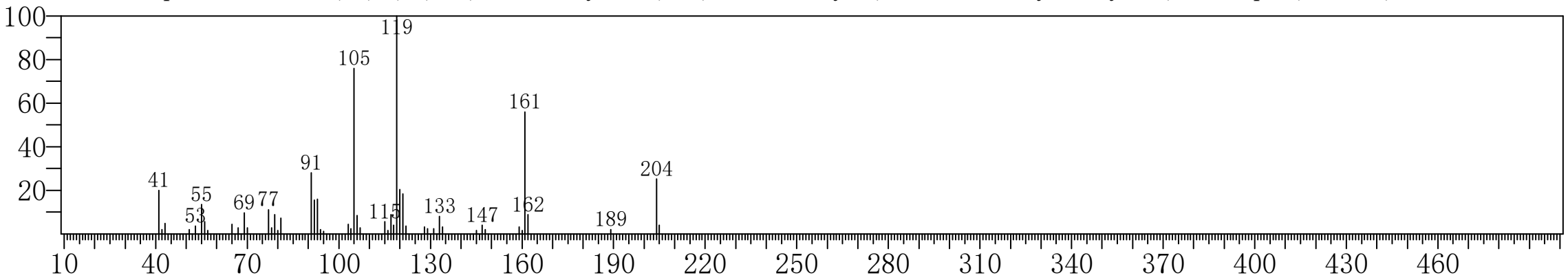

命中#:2　输入:22380　谱库:NIST17s.lib
SI:94　分子式:C15H24　CAS:16728-99-7　摩尔质量:204　保留指数:1440
组分名称:Naphthalene, 1,2,3,4,4a,7-hexahydro-1,6-dimethyl-4-(1-methylethyl)- $$ Naphthalene, 1,2,3,4,6,8a-he

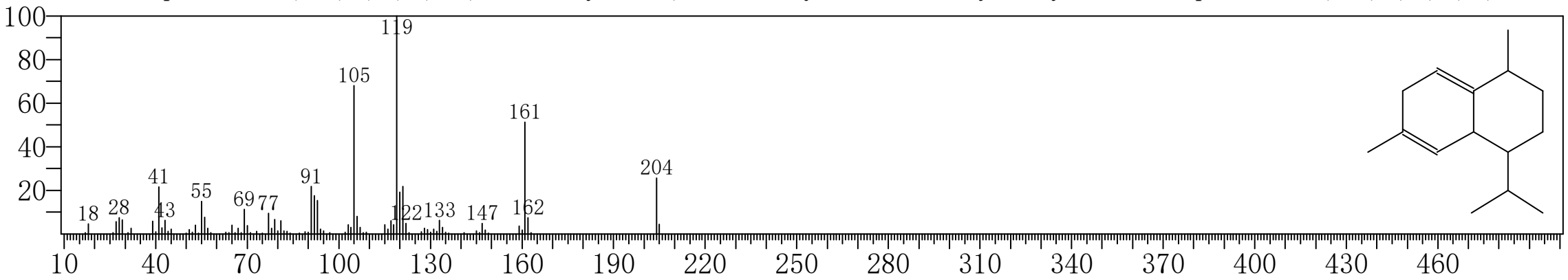

命中#:3　输入:52853　谱库:NIST17-1.lib
SI:92　分子式:C15H24　CAS:29837-12-5　摩尔质量:204　保留指数:1440
组分名称:Cubenene $$ Cadina-1,4-diene, cis $$ Naphthalene, 1,2,3,4,4a,7-hexahydro-1,6-dimethyl-4-(1-methyle

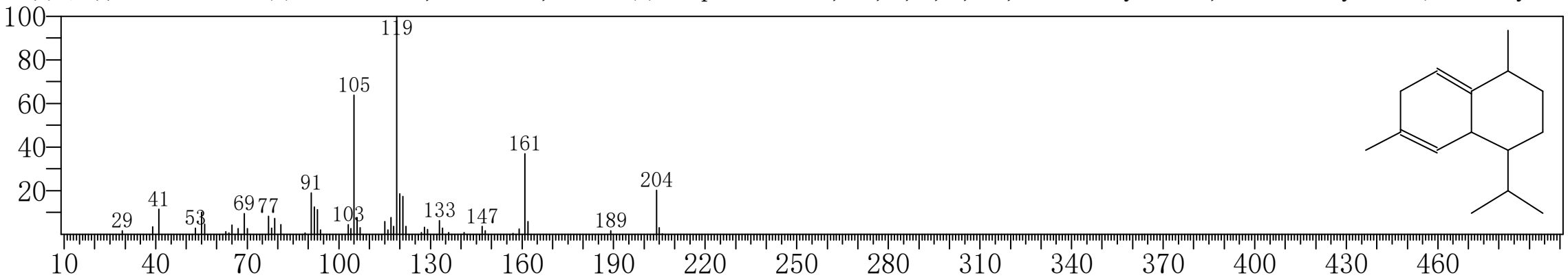

命中#:4　输入:52850　谱库:NIST17-1.lib
SI:89　分子式:C15H24　CAS:16728-99-7　摩尔质量:204　保留指数:1440
组分名称:Naphthalene, 1,2,3,4,4a,7-hexahydro-1,6-dimethyl-4-(1-methylethyl)- $$ Naphthalene, 1,2,3,4,6,8a-he

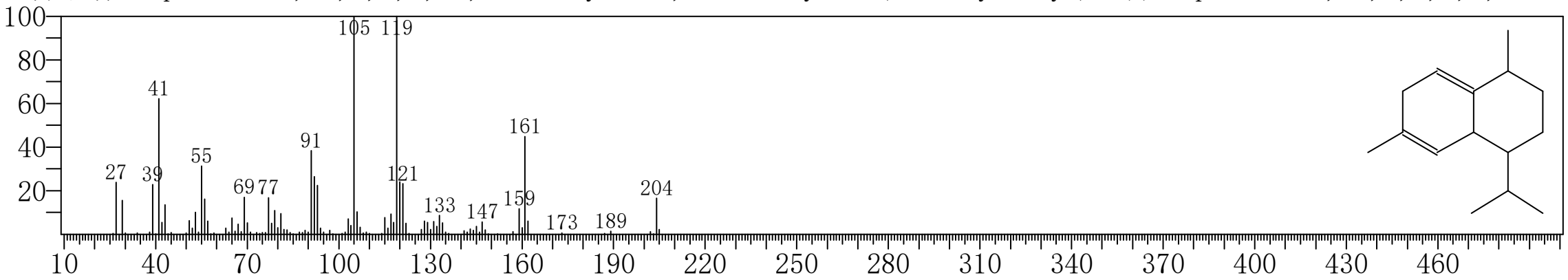

命中#:5　输入:1095　谱库:FFNSC1.3.lib
SI:89　分子式:C15 H24　CAS:31141-66-9　摩尔质量:204　保留指数:1349
组分名称:Cubebene <alpha->

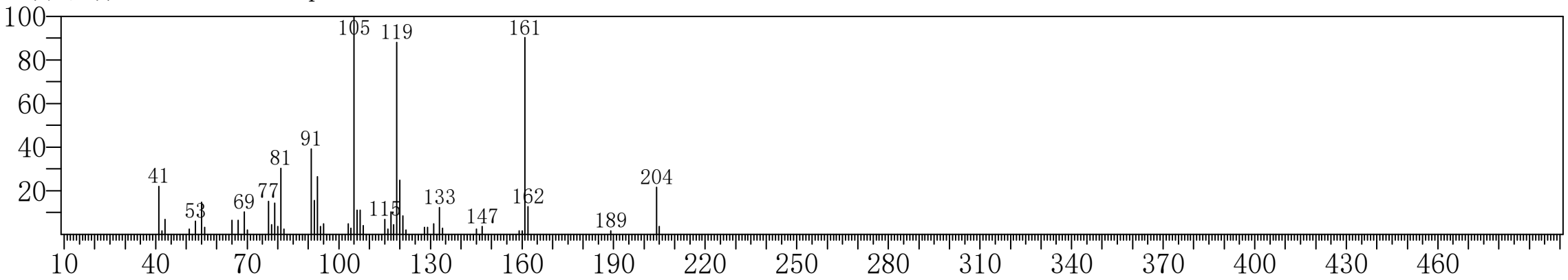

<< 目标组分 >>
行号#:48　保留时间:33.805(扫描数#:5762)　质量峰:268
原始模式:平均 33.800-33.810(5761-5763)　基峰:105.05(115268)
背景模式:峰校准　组 1 - 事件 1　Scan

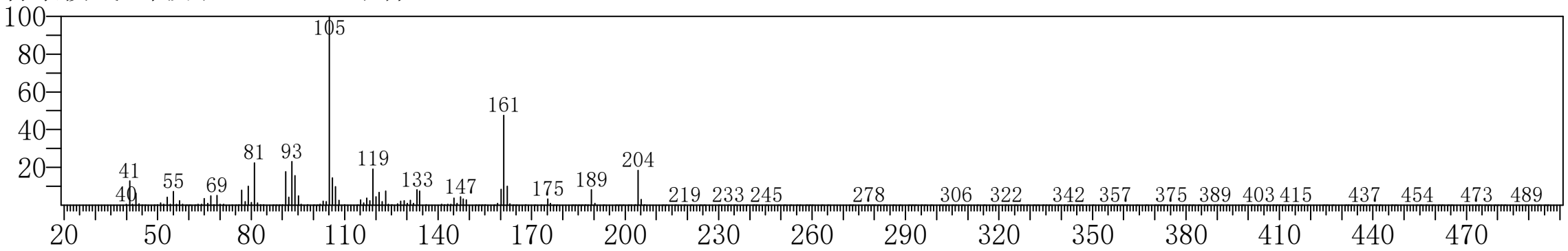

命中#:1　输入:1763　谱库:FFNSC1.3.lib
SI:95　分子式:C15 H24　CAS:82468-90-4　摩尔质量:204　保留指数:1538
组分名称:Cadinene <alpha->

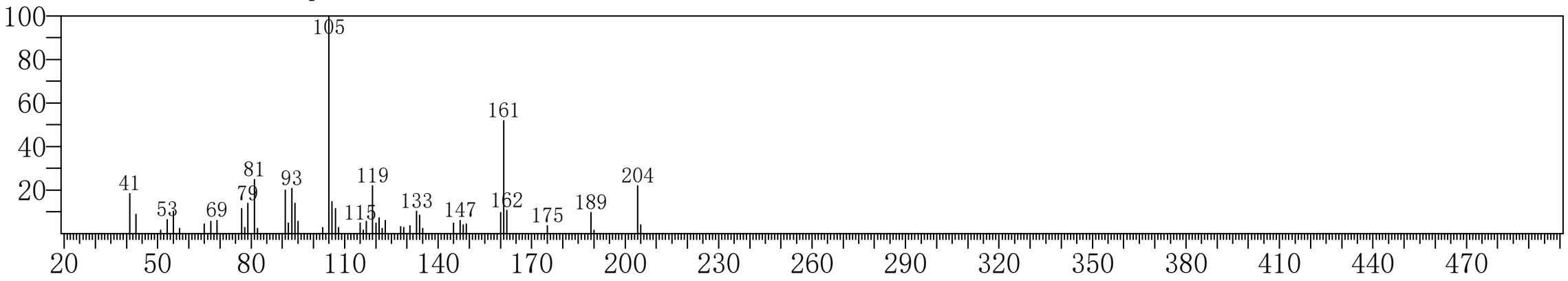

命中#:2　输入:22346　谱库:NIST17s.lib
SI:95　分子式:C15H24　CAS:24406-05-1　摩尔质量:204　保留指数:1440
组分名称:Naphthalene, 1,2,4a,5,6,8a-hexahydro-4,7-dimethyl-1-(1-methylethyl)-, [1S-(1.alpha.,4a.beta.,8a.al

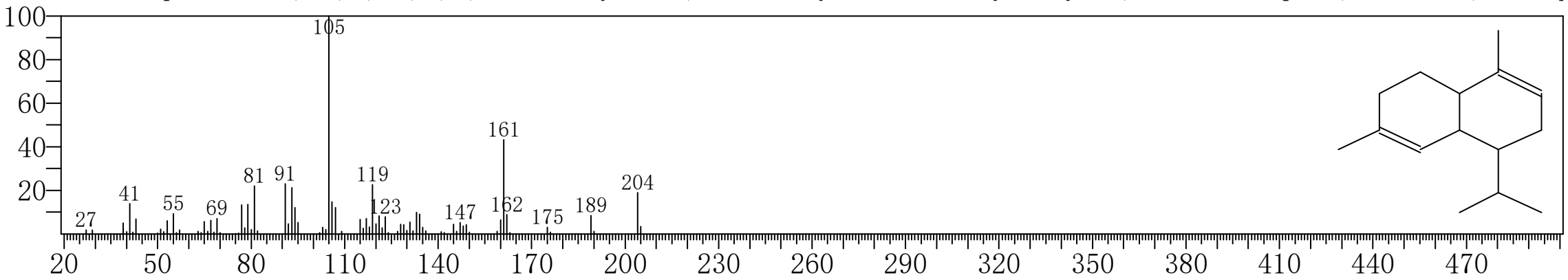

命中#:3　输入:52815　谱库:NIST17-1.lib
SI:93　分子式:C15H24　CAS:24406-05-1　摩尔质量:204　保留指数:1440
组分名称:Naphthalene, 1,2,4a,5,6,8a-hexahydro-4,7-dimethyl-1-(1-methylethyl)-, [1S-(1.alpha.,4a.beta.,8a.al

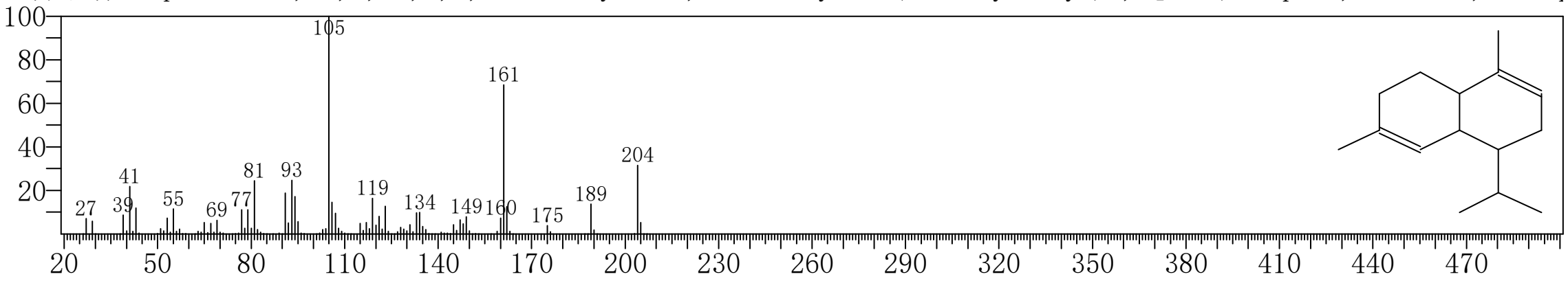

命中#:4　输入:22347　谱库:NIST17s.lib
SI:93　分子式:C15H24　CAS:10208-80-7　摩尔质量:204　保留指数:1440
组分名称:.alpha.-Muurolene $$ Naphthalene, 1,2,4a,5,6,8a-hexahydro-4,7-dimethyl-1-(1-methylethyl)-, (1S,4aS,

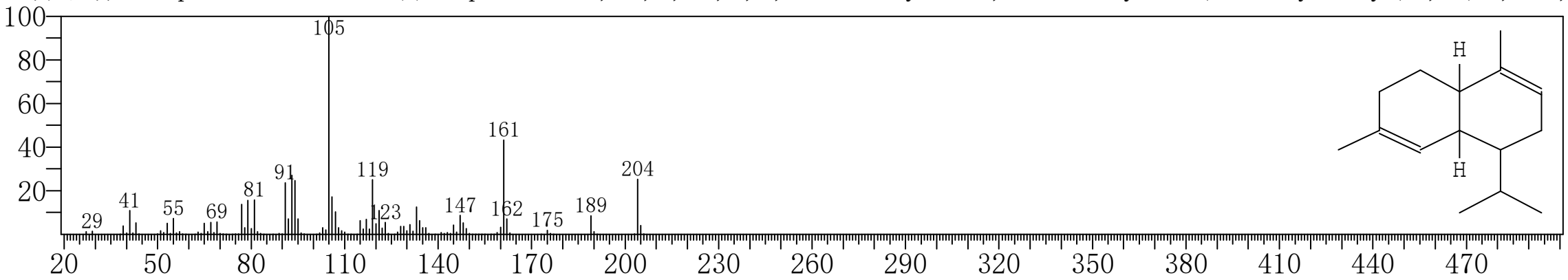

命中#:5　输入:52812　谱库:NIST17-1.lib
SI:93　分子式:C15H24　CAS:10208-80-7　摩尔质量:204　保留指数:1440
组分名称:.alpha.-Muurolene $$ Naphthalene, 1,2,4a,5,6,8a-hexahydro-4,7-dimethyl-1-(1-methylethyl)-, (1S,4aS,

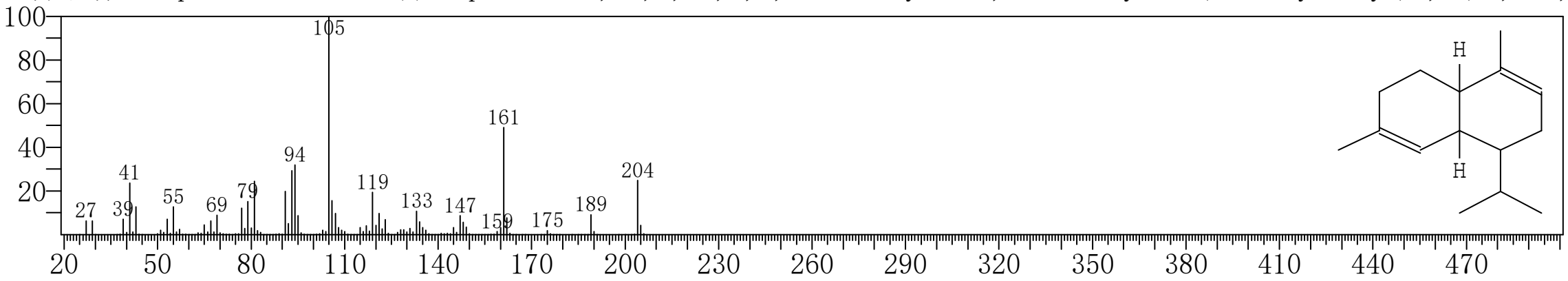

<< 目标组分 >>
行号#:49　 保留时间:35.195(扫描数#:6040)　质量峰:301
原始模式:平均 35.190-35.200(6039-6041)　 基峰:123.10(40796)
背景模式:峰校准　组 1 - 事件 1　Scan

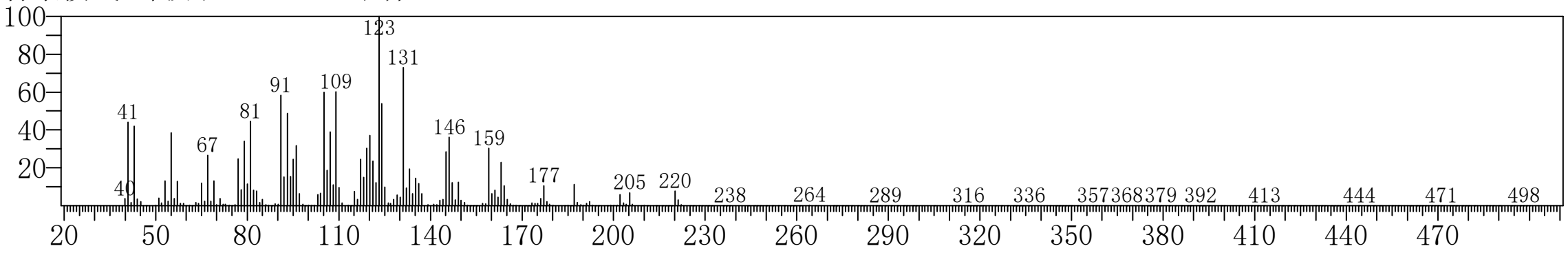

命中#:1　输入:66705　谱库:NIST17-1.lib
SI:80　分子式:C15H24O　CAS:0-00-0　摩尔质量:220　保留指数:1745
组分名称:2-(4a,8-Dimethyl-1,2,3,4,4a,5,6,7-octahydro-naphthalen-2-yl)-prop-2-en-1-ol $$ 2-(4a,8-Dimethyl-1,2

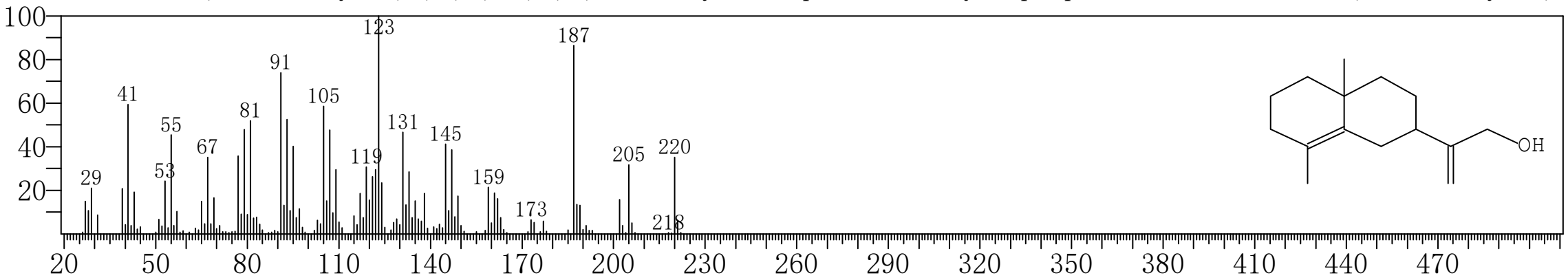

命中#:2　输入:66676　谱库:NIST17-1.lib
SI:80　分子式:C15H24O　CAS:81968-62-9　摩尔质量:220　保留指数:1699
组分名称:(1R,7S,E)-7-Isopropyl-4,10-dimethylenecyclodec-5-enol $$ 5-Cyclodecen-1-ol, 4,10-bis(methylene)-7-

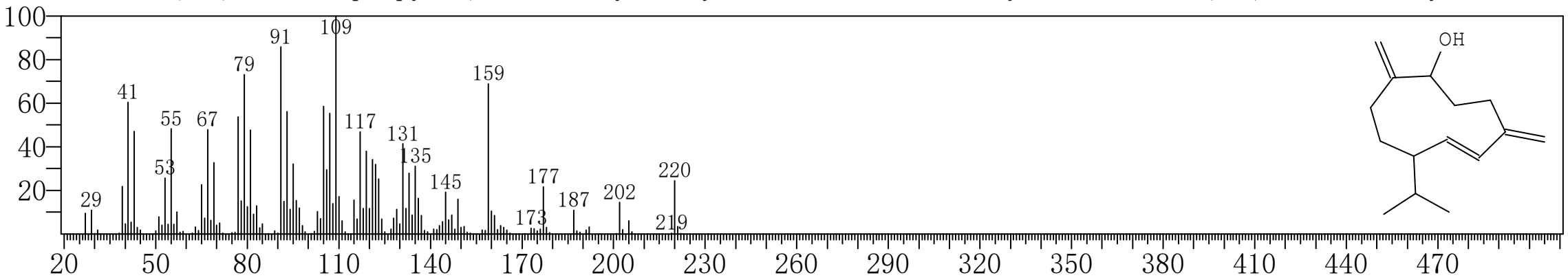

命中#:3　输入:1714　谱库:FFNSC1.3.lib
SI:79　分子式:C15 H24 O　CAS:79580-01-1　摩尔质量:220　保留指数:1636
组分名称:Bicyclo[7.2.0]undecan-3-ol <11,11-dimethyl-, 4,8-bis(methylene)->

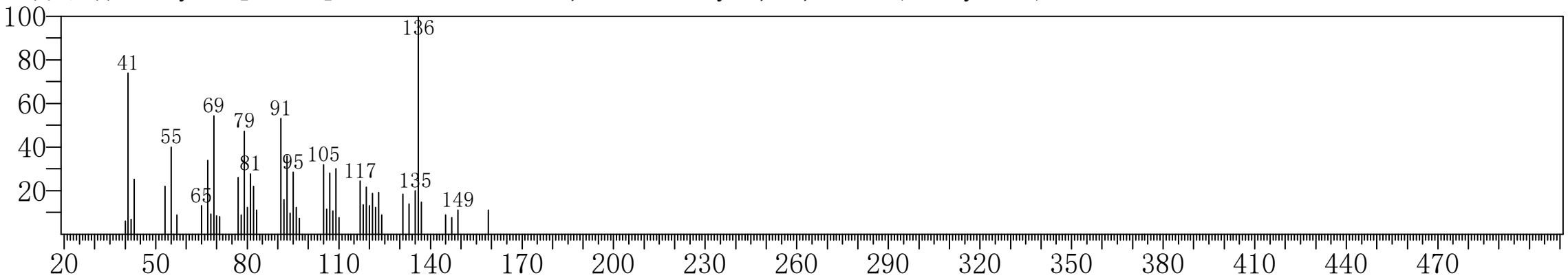

命中#:4　输入:903　谱库:FFNSC1.3.lib
SI:78　分子式:C15 H24 O　CAS:72203-24-8　摩尔质量:220　保留指数:1576
组分名称:Spathulenol

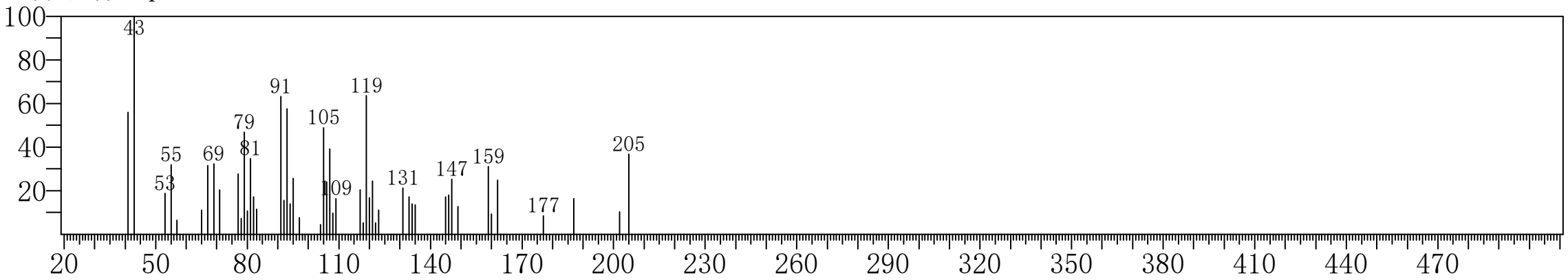

命中#:5　输入:1159　谱库:FFNSC1.3.lib
SI:78　分子式:C14 H22 O　CAS:3155-71-3　摩尔质量:206　保留指数:1581
组分名称:But-2-enal <2-methyl-, 4-(2,6,6-trimethyl-1-cyclohexen-1-yl)->

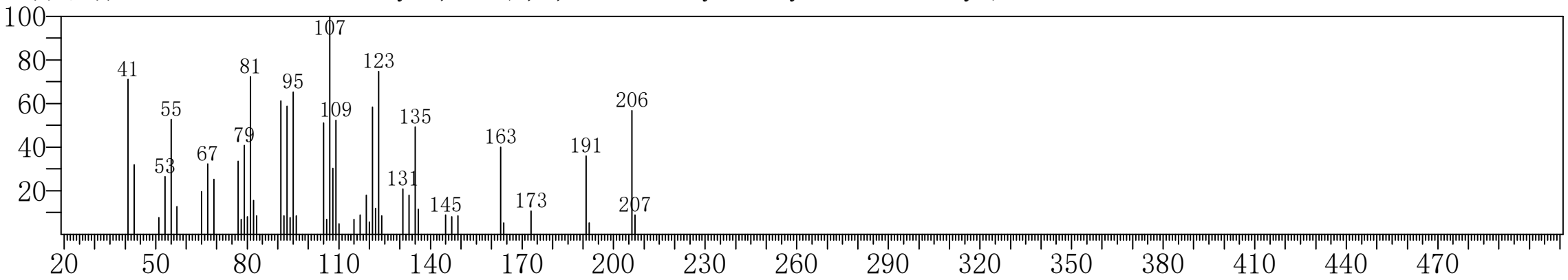

<< 目标组分 >>
行号#:50　保留时间:36.950(扫描数#:6391)　质量峰:335
原始模式:平均 36.945-36.955(6390-6392)　基峰:43.00(150660)
背景模式:峰校准　组 1 - 事件 1　Scan

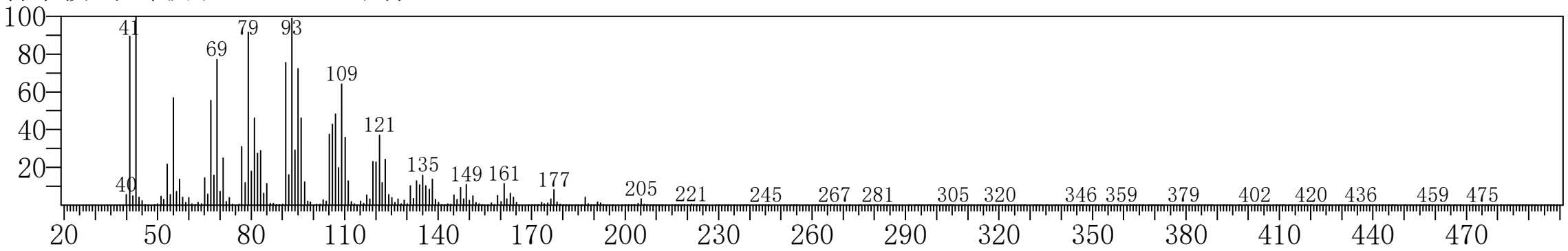

命中#:1　输入:145　谱库:FFNSC1.3.lib
SI:94　分子式:C15 H24 O　CAS:17627-43-9　摩尔质量:220　保留指数:1587
组分名称:Caryophyllene oxide

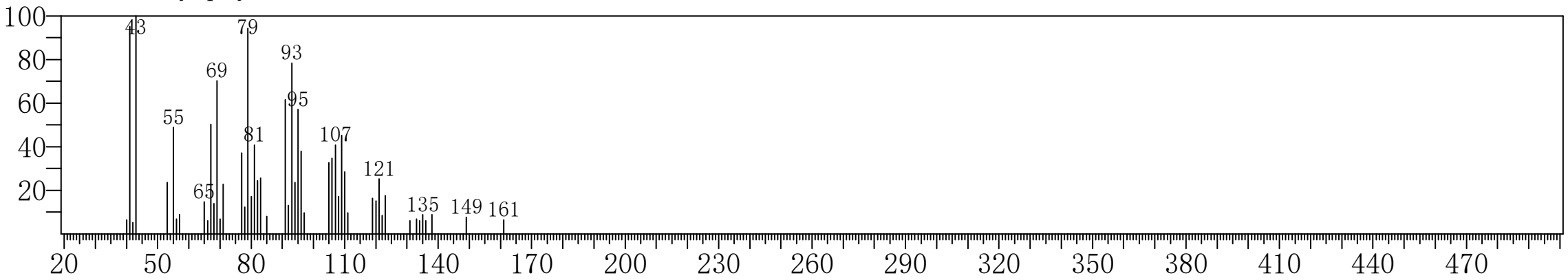

命中#:2　输入:24969　谱库:NIST17s.lib
SI:92　分子式:C15H24O　CAS:1139-30-6　摩尔质量:220　保留指数:1507
组分名称:Caryophyllene oxide $$ 5-Oxatricyclo[8.2.0.0(4,6)-]dodecane, 4,12,12-trimethyl-9-methylene-, [1R-(

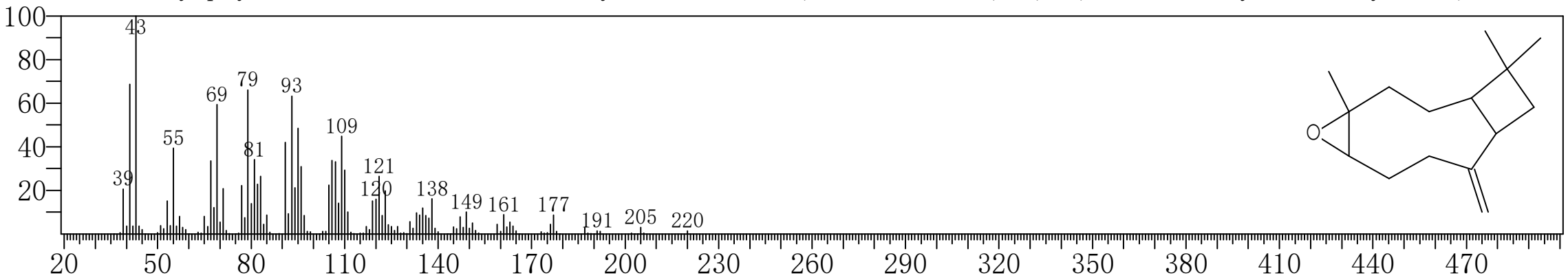

命中#:3　输入:66606　谱库:NIST17-1.lib
SI:91　分子式:C15H24O　CAS:1139-30-6　摩尔质量:220　保留指数:1507
组分名称:Caryophyllene oxide $$ 5-Oxatricyclo[8.2.0.0(4,6)-]dodecane, 4,12,12-trimethyl-9-methylene-, [1R-(

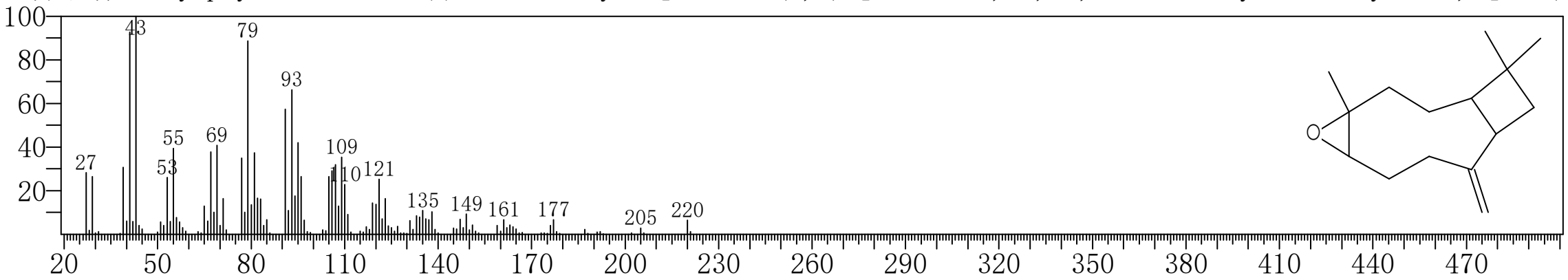

命中#:4　输入:66617　谱库:NIST17-1.lib
SI:86　分子式:C15H24O　CAS:19888-00-7　摩尔质量:220　保留指数:1762
组分名称:Humulenol-II $$ 4,8-Cycloundecadien-1-ol, 6,6,9-trimethyl-2-methylene-, (1R,4E,8E)- $$ (R,4E,8E)-6,

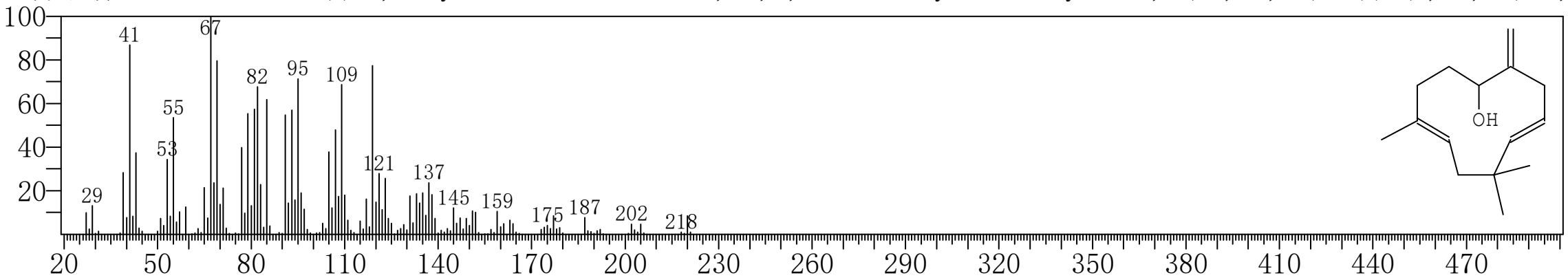

命中#:5　输入:1643　谱库:FFNSC1.3.lib
SI:85　分子式:C15 H24 O　CAS:19888-34-7　摩尔质量:220　保留指数:1613
组分名称:Humulene epoxide II

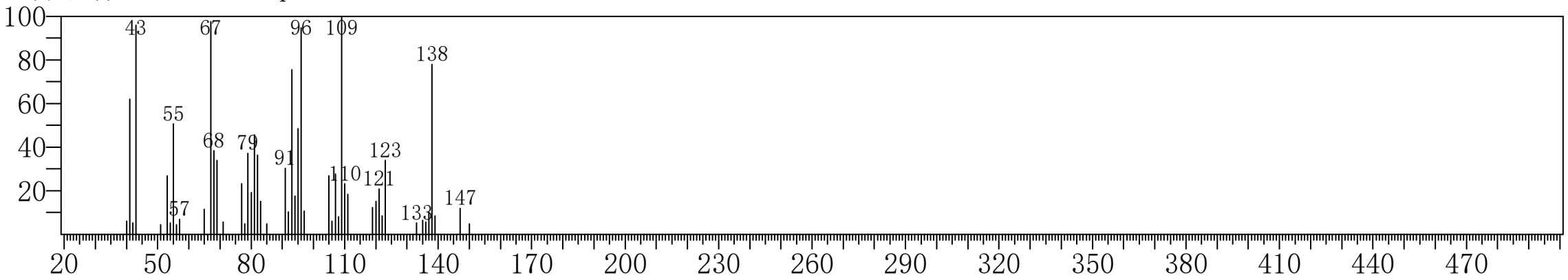

<< 目标组分 >>
行号#:51　保留时间:37.595(扫描数#:6520)　质量峰:312
原始模式:平均 37.590-37.600(6519-6521)　基峰:135.15(34764)
背景模式:峰校准　组 1 - 事件 1　Scan

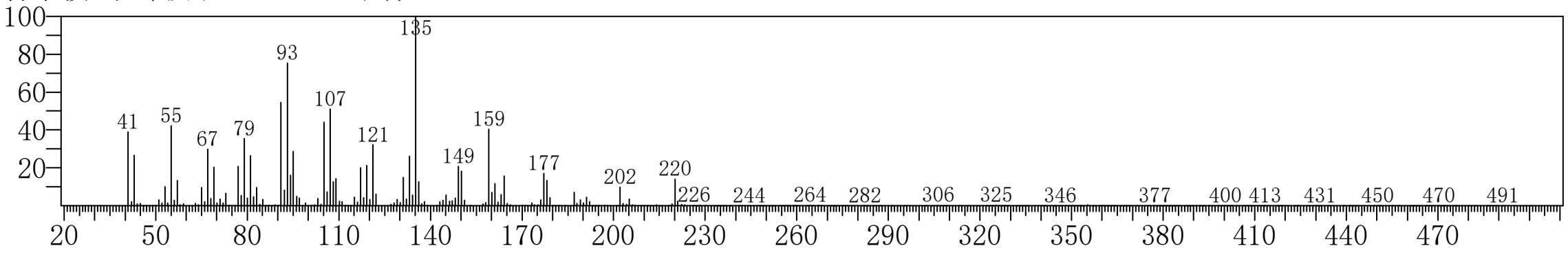

命中#:1　输入:66710　谱库:NIST17-1.lib
SI:80　分子式:C15H24O　CAS:41610-69-9　摩尔质量:220　保留指数:1464
组分名称:Ylangenol $$ (8-Isopropyl-1-methyltricyclo[4.4.0.02,7]dec-3-en-3-yl)methanol $$ Tricyclo[4.4.0.02,7

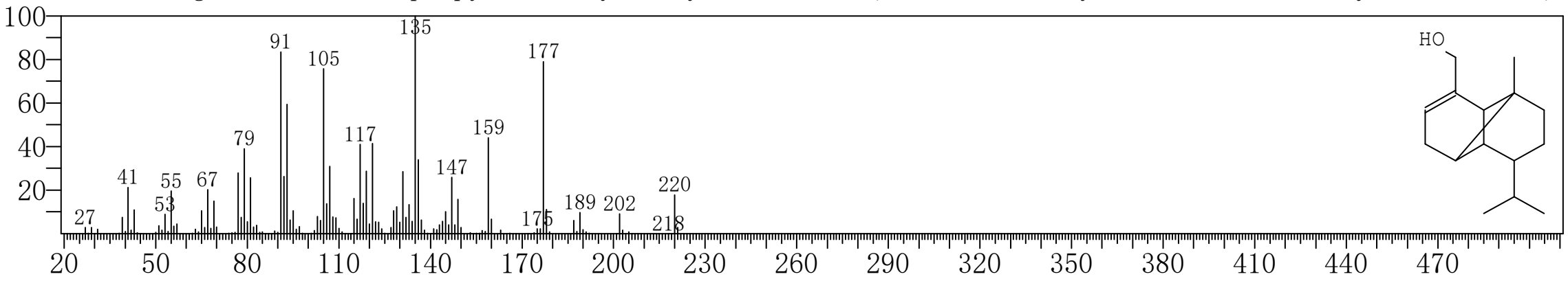

命中#:2　输入:66598　谱库:NIST17-1.lib
SI:79　分子式:C15H24O　CAS:0-00-0　摩尔质量:220　保留指数:1462
组分名称:Alloaromadendrene oxide-(2)

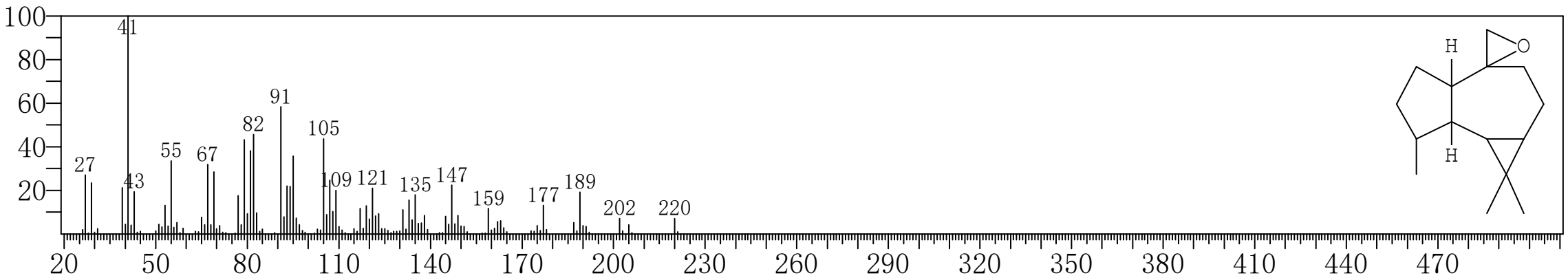

命中#:3　输入:66708　谱库:NIST17-1.lib
SI:79　分子式:C15H24O　CAS:149496-35-5　摩尔质量:220　保留指数:1716
组分名称:((1R,4S,5R)-1-Methyl-4-(prop-1-en-2-yl)spiro[4.5]dec-7-en-8-yl)methanol $$ Spiro[4.5]dec-7-ene-8-me

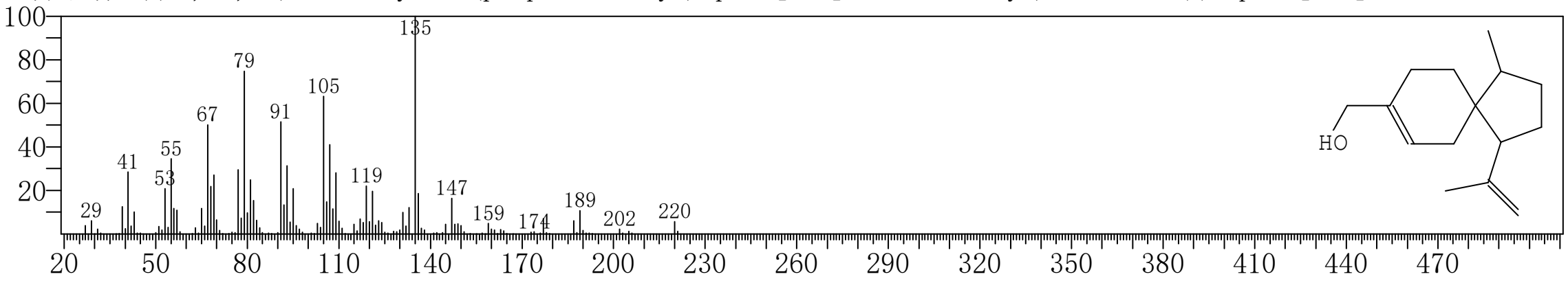

命中#:4　输入:66676　谱库:NIST17-1.lib
SI:79　分子式:C15H24O　CAS:81968-62-9　摩尔质量:220　保留指数:1699
组分名称:(1R,7S,E)-7-Isopropyl-4,10-dimethylenecyclodec-5-enol $$ 5-Cyclodecen-1-ol, 4,10-bis(methylene)-7-

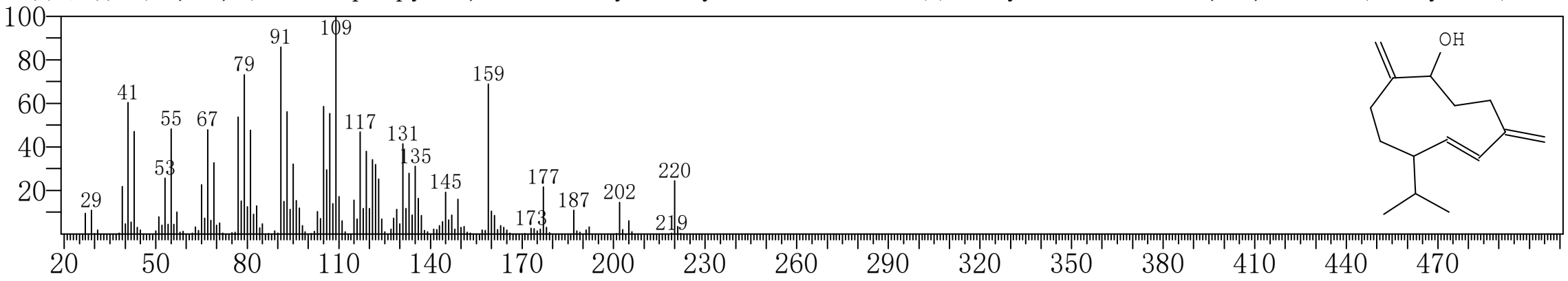

命中#:5　输入:66739　谱库:NIST17-1.lib
SI:79　分子式:C15H24O　CAS:0-00-0　摩尔质量:220　保留指数:1690
组分名称:6-Isopropenyl-4,8a-dimethyl-1,2,3,5,6,7,8,8a-octahydro-naphthalen-2-ol $$ 6-Isopropenyl-4,8a-dimetl

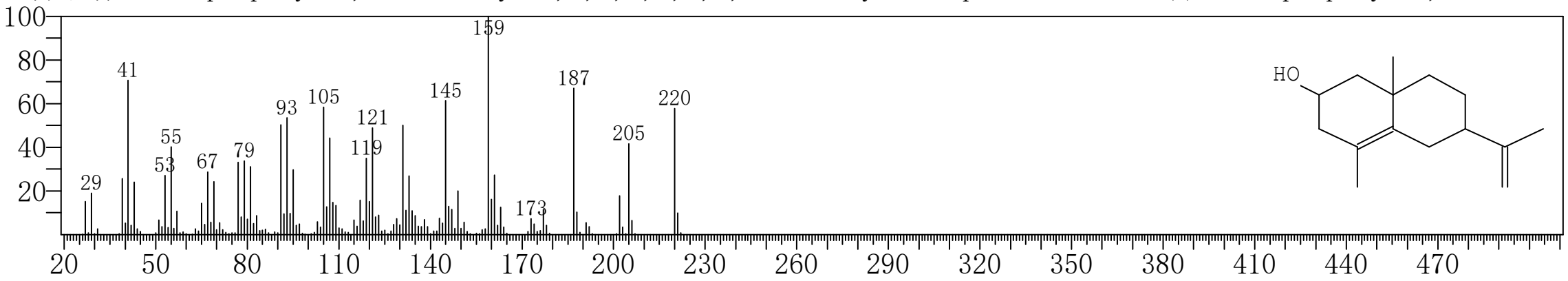

<< 目标组分 >>
行号#:52　保留时间:38.780(扫描数#:6757)　质量峰:304
原始模式:平均 38.775-38.785(6756-6758)　基峰:88.05(161427)
背景模式:峰校准　组 1 - 事件 1　Scan

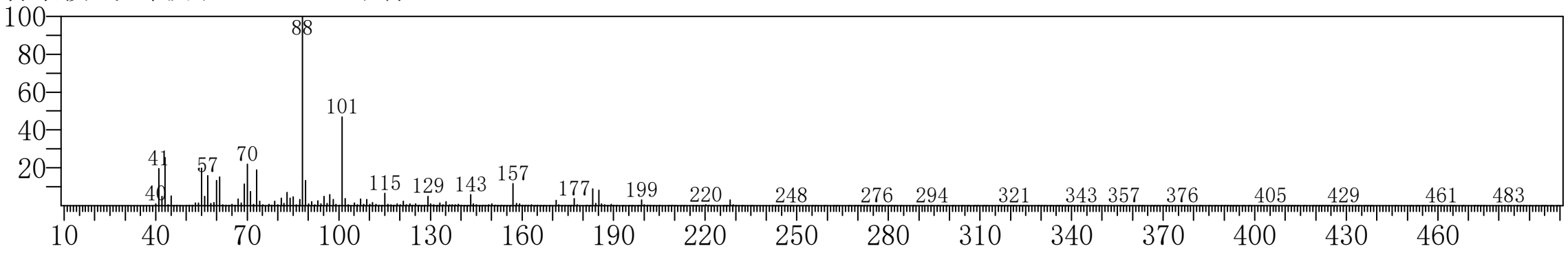

命中#:1　输入:162　谱库:FFNSC1.3.lib
SI:94　分子式:C14 H28 O2　CAS:106-33-2　摩尔质量:228　保留指数:1598
组分名称:Dodecanoate <ethyl->

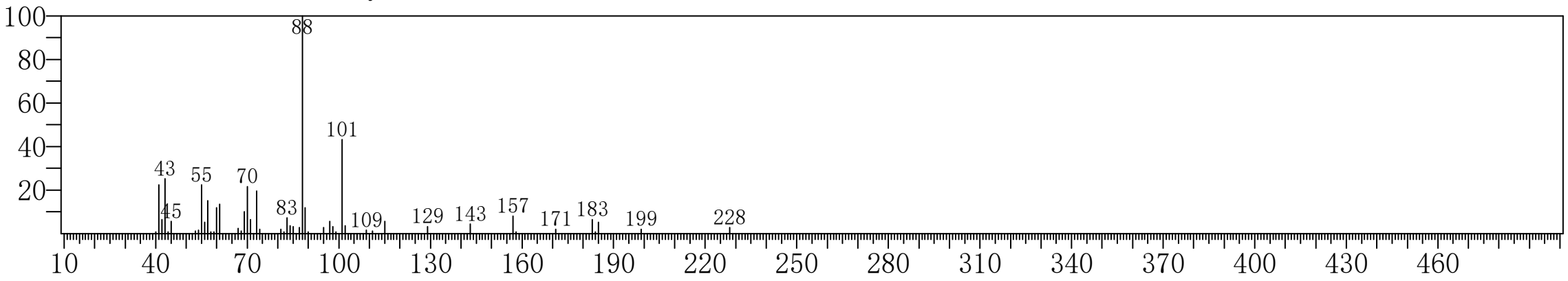

命中#:2　输入:26239　谱库:NIST17s.lib
SI:93　分子式:C14H28O2　CAS:106-33-2　摩尔质量:228　保留指数:1580
组分名称:Dodecanoic acid, ethyl ester $$ Lauric acid, ethyl ester $$ Ethyl dodecanoate $$ Ethyl dodecylate $

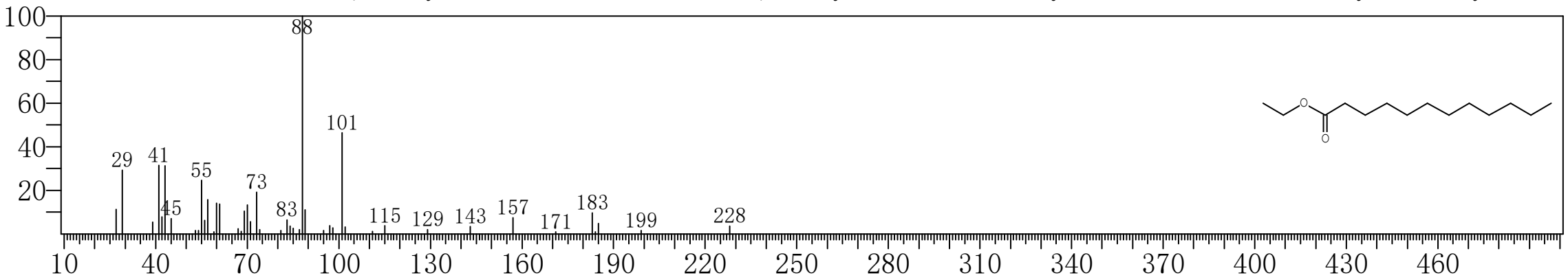

命中#:3　输入:74188　谱库:NIST17-1.lib
SI:92　分子式:C14H28O2　CAS:106-33-2　摩尔质量:228　保留指数:1580
组分名称:Dodecanoic acid, ethyl ester $$ Lauric acid, ethyl ester $$ Ethyl dodecanoate $$ Ethyl dodecylate $

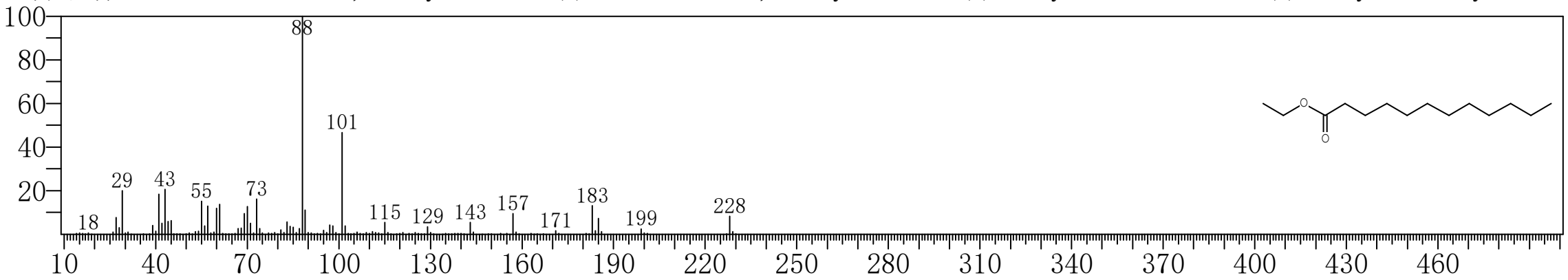

命中#:4　输入:95　谱库:FFNSC1.3.lib
SI:91　分子式:C13 H26 O2　CAS:627-90-7　摩尔质量:214　保留指数:1498
组分名称:Undecanoate <ethyl->

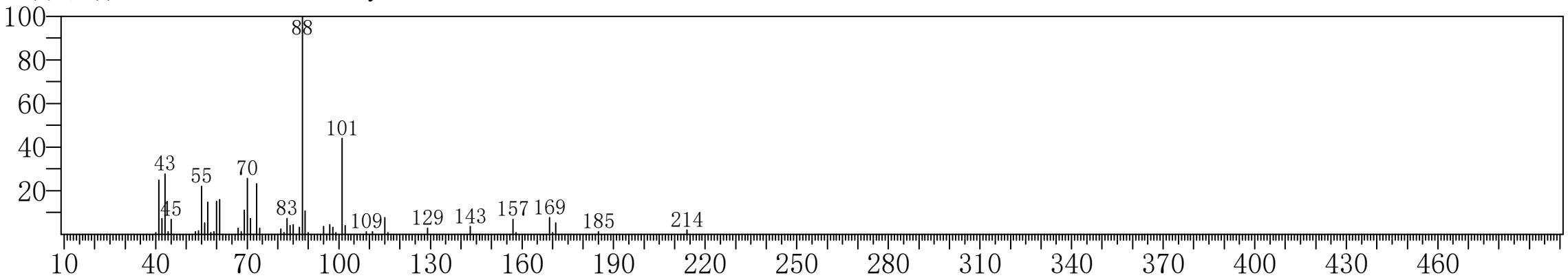

命中#:5　输入:26242　谱库:NIST17s.lib
SI:91　分子式:C14H28O2　CAS:106-33-2　摩尔质量:228　保留指数:1580
组分名称:Dodecanoic acid, ethyl ester $$ Lauric acid, ethyl ester $$ Ethyl dodecanoate $$ Ethyl dodecylate $

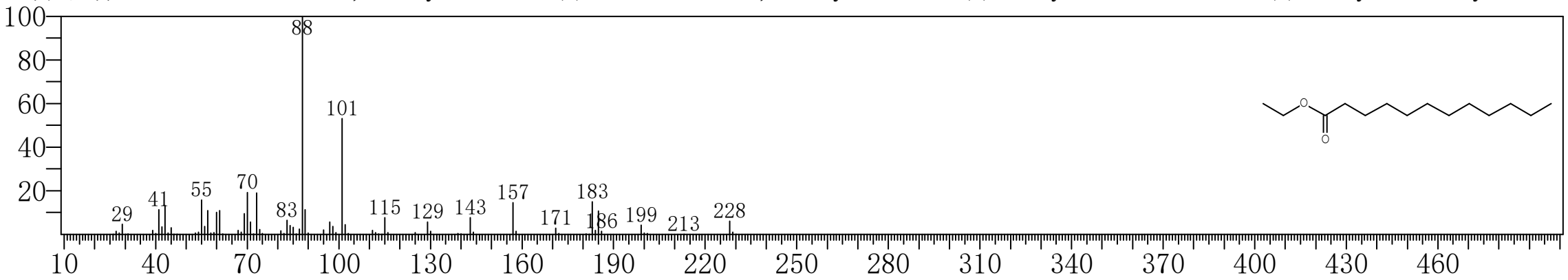

<< 目标组分 >>
行号#:53　保留时间:39.095(扫描数#:6820)　质量峰:282
原始模式:平均 39.090-39.100(6819-6821)　基峰:96.10(24058)
背景模式:峰校准　组 1 - 事件 1　Scan

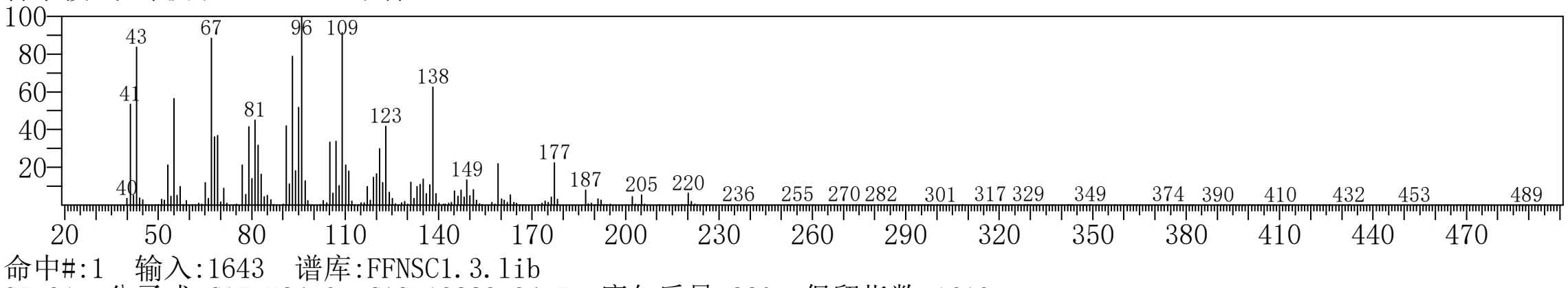

命中#:1　输入:1643　谱库:FFNSC1.3.lib
SI:91　分子式:C15 H24 O　CAS:19888-34-7　摩尔质量:220　保留指数:1613
组分名称:Humulene epoxide II

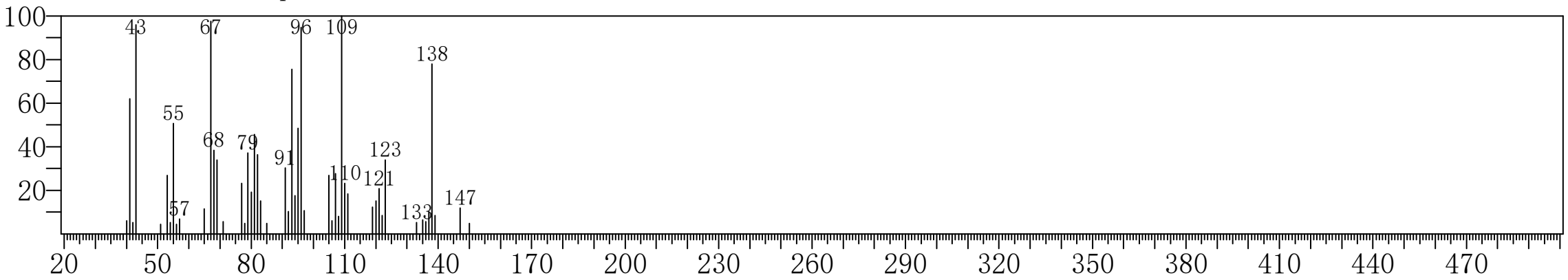

命中#:2　输入:66675　谱库:NIST17-1.lib
SI:87　分子式:C15H24O　CAS:19888-34-7　摩尔质量:220　保留指数:1592
组分名称:(1R,3E,7E,11R)-1,5,5,8-Tetramethyl-12-oxabicyclo[9.1.0]dodeca-3,7-diene $$ 12-Oxabicyclo[9.1.0]dode

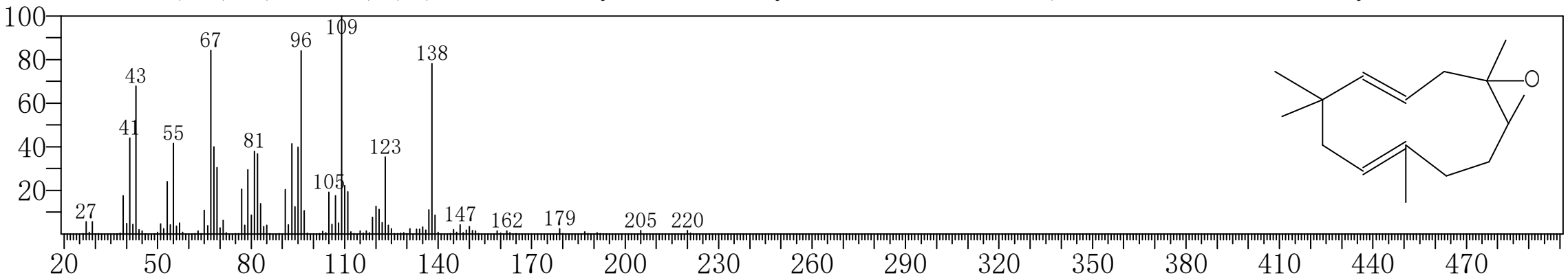

命中#:3　输入:24969　谱库:NIST17s.lib
SI:86　分子式:C15H24O　CAS:1139-30-6　摩尔质量:220　保留指数:1507
组分名称:Caryophyllene oxide $$ 5-Oxatricyclo[8.2.0.0(4,6)-]dodecane, 4,12,12-trimethyl-9-methylene-, [1R-(

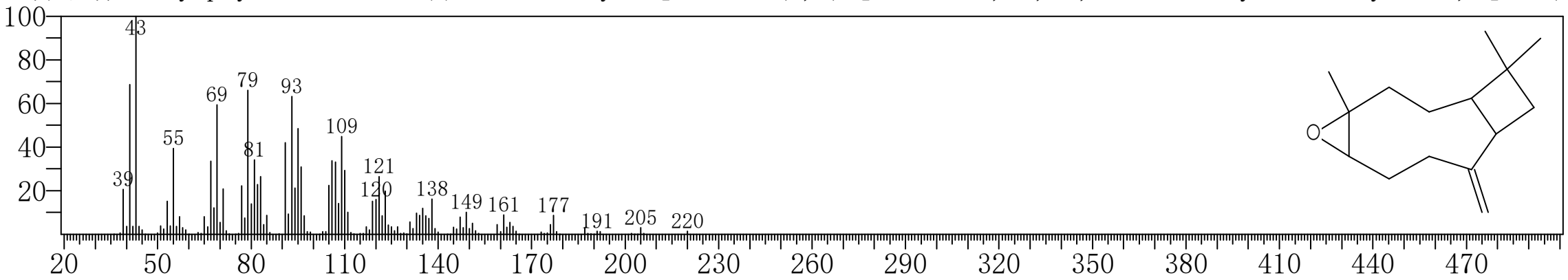

命中#:4　输入:1334　谱库:FFNSC1.3.lib
SI:84　分子式:C15 H24 O　CAS:71735-79-0　摩尔质量:220　保留指数:1716
组分名称:Bicyclo[10.1.0]trideca-4,8-diene <13-oxa-, trimethyl->

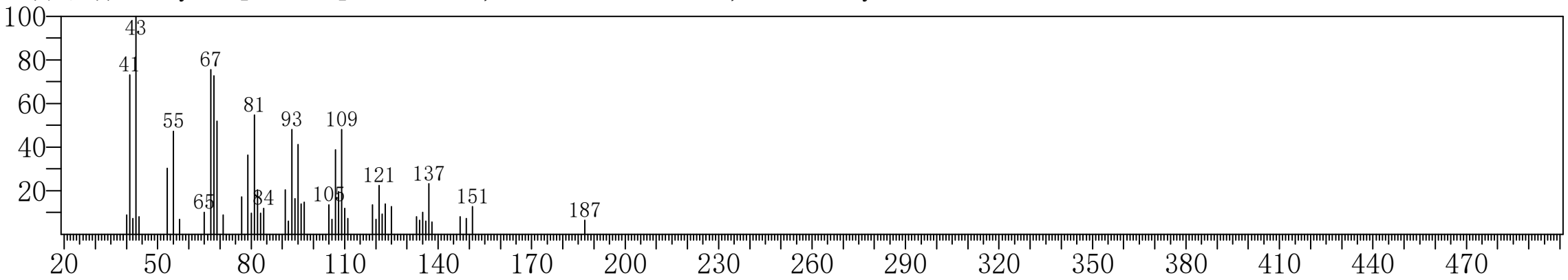

命中#:5　输入:66606　谱库:NIST17-1.lib
SI:84　分子式:C15H24O　CAS:1139-30-6　摩尔质量:220　保留指数:1507
组分名称:Caryophyllene oxide $$ 5-Oxatricyclo[8.2.0.0(4,6)-]dodecane, 4,12,12-trimethyl-9-methylene-, [1R-(

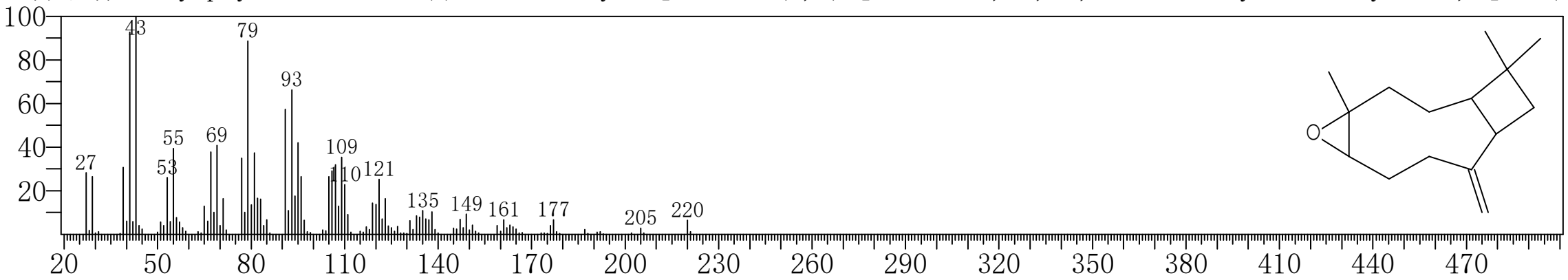

<< 目标组分 >>
行号#:54　保留时间:39.835(扫描数#:6968)　质量峰:276
原始模式:平均 39.830-39.840(6967-6969)　基峰:131.10(26027)
背景模式:峰校准　组 1 - 事件 1　Scan

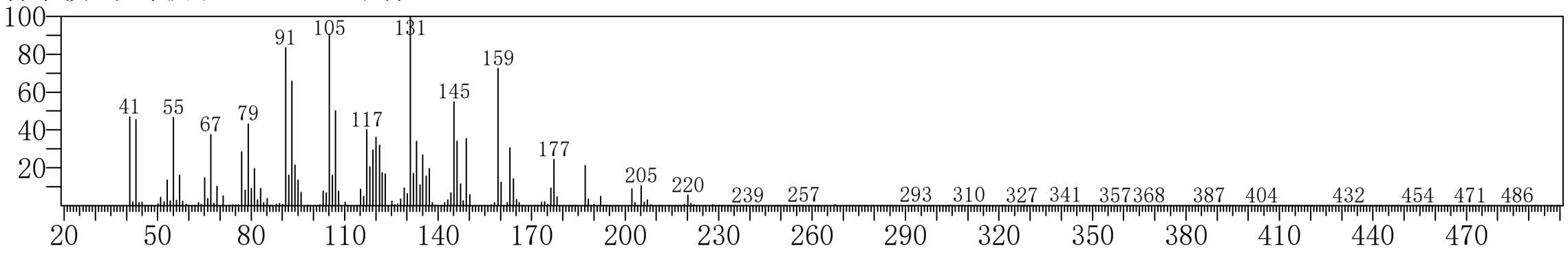

命中#:1　输入:24989　谱库:NIST17s.lib
SI:82　分子式:C15H24O　CAS:6750-60-3　摩尔质量:220　保留指数:1536
组分名称:1H-Cycloprop[e]azulen-7-ol, decahydro-1,1,7-trimethyl-4-methylene-, [1ar-(1a.alpha.,4a.alpha.,7.be

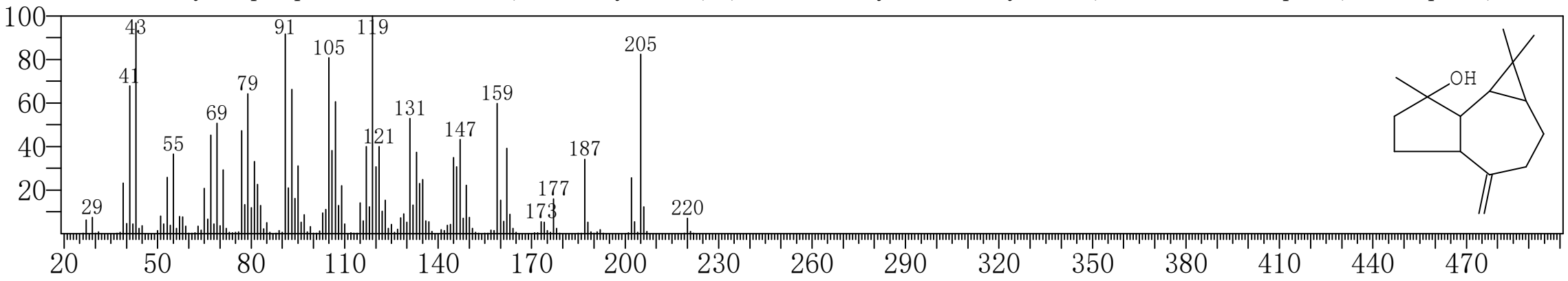

命中#:2　输入:66739　谱库:NIST17-1.lib
SI:81　分子式:C15H24O　CAS:0-00-0　摩尔质量:220　保留指数:1690
组分名称:6-Isopropenyl-4,8a-dimethyl-1,2,3,5,6,7,8,8a-octahydro-naphthalen-2-ol $$ 6-Isopropenyl-4,8a-dimetl

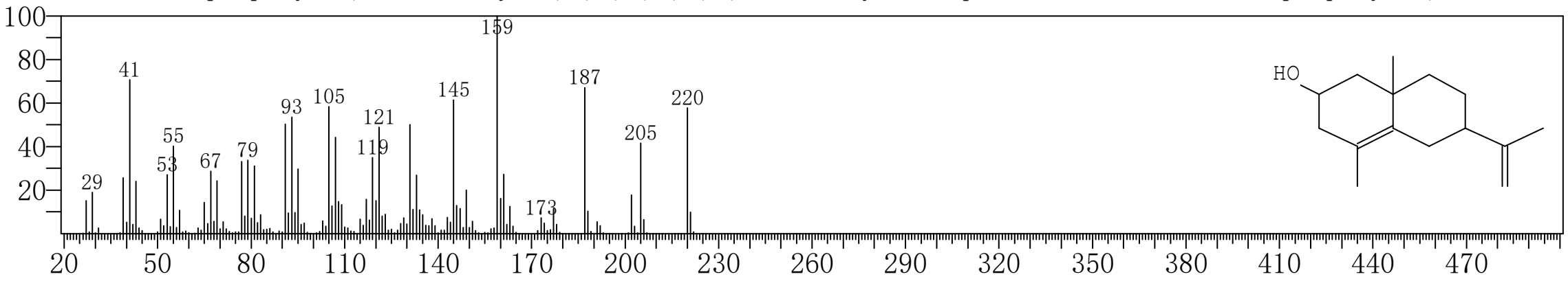

命中#:3　输入:903　谱库:FFNSC1.3.lib
SI:79　分子式:C15 H24 O　CAS:72203-24-8　摩尔质量:220　保留指数:1576
组分名称:Spathulenol

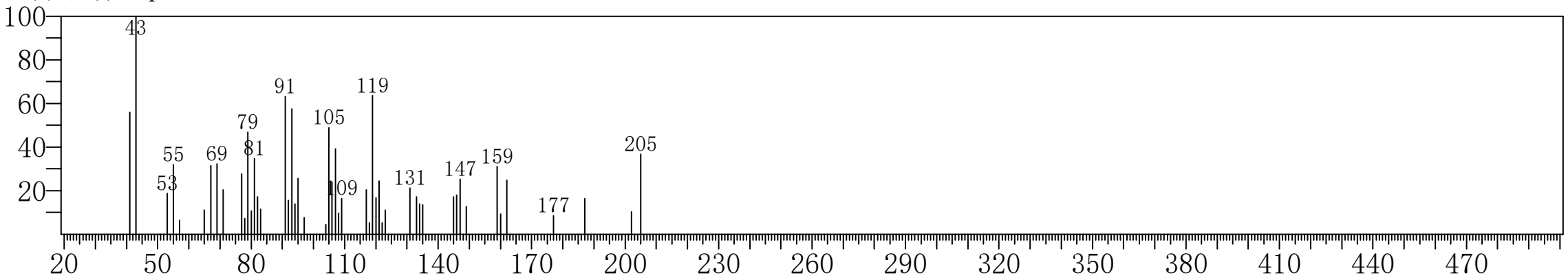

命中#:4　输入:66688　谱库:NIST17-1.lib
SI:79　分子式:C15H24O　CAS:38142-56-2　摩尔质量:220　保留指数:1663
组分名称:6-Methyl-2-(4-methylcyclohex-3-en-1-yl)hepta-1,5-dien-4-ol $$ 3-Cyclohexene-1-propanol, 4-methyl-.

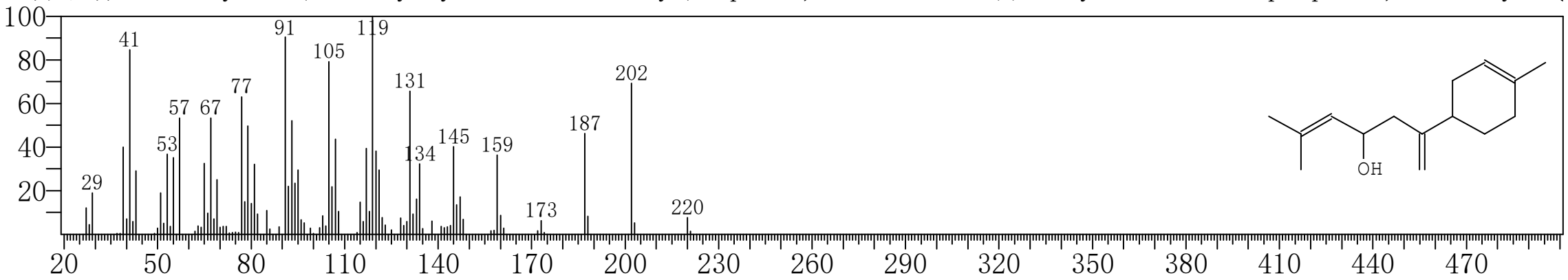

命中#:5　输入:66603　谱库:NIST17-1.lib
SI:79　分子式:C15H24O　CAS:0-00-0　摩尔质量:220　保留指数:1599
组分名称:Tricyclo[5.2.2.0(1,6)]undecan-3-ol, 2-methylene-6,8,8-trimethyl-

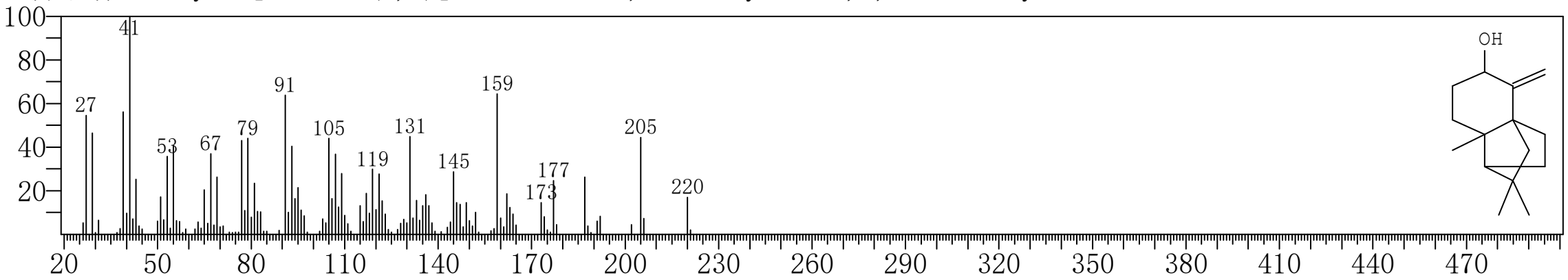

<< 目标组分 >>
行号#:55　保留时间:40.035(扫描数#:7008)　质量峰:273
原始模式:平均 40.030-40.040(7007-7009)　基峰:109.10(77522)
背景模式:峰校准　组 1 - 事件 1　Scan

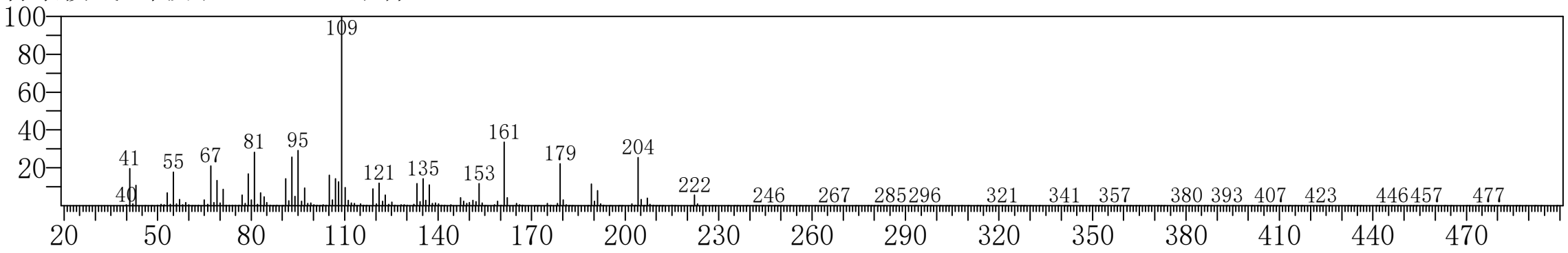

命中#:1　输入:68724　谱库:NIST17-1.lib
SI:90　分子式:C15H26O　CAS:472-07-1　摩尔质量:222　保留指数:1625
组分名称:Junenol $$ (1S,2S,4aR,8aS)-2-Isopropyl-4a-methyl-8-methylenedecahydronaphthalen-1-ol $$ 1-Naphthale

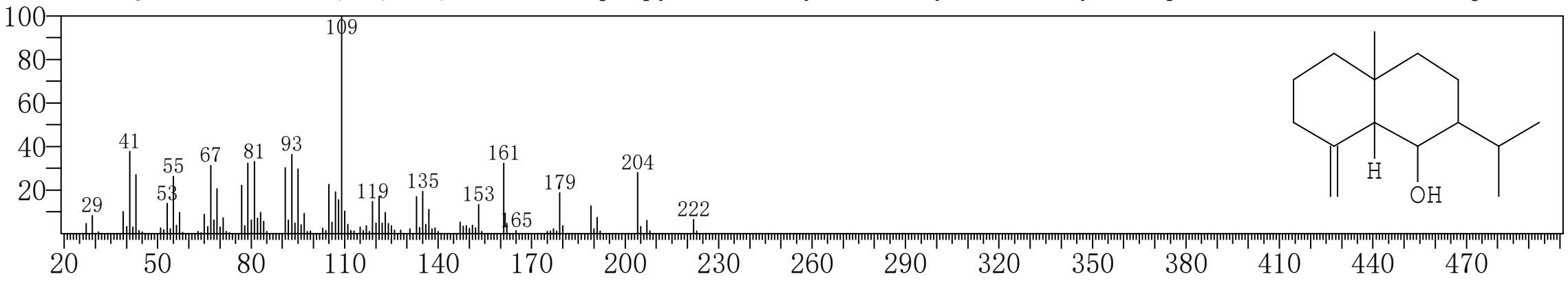

命中#:2　输入:68727　谱库:NIST17-1.lib
SI:80　分子式:C15H26O　CAS:0-00-0　摩尔质量:222　保留指数:1757
组分名称:Humulane-1,6-dien-3-ol $$ 3,7,7,10-Tetramethyl-2,8-cycloundecadien-1-ol # $$

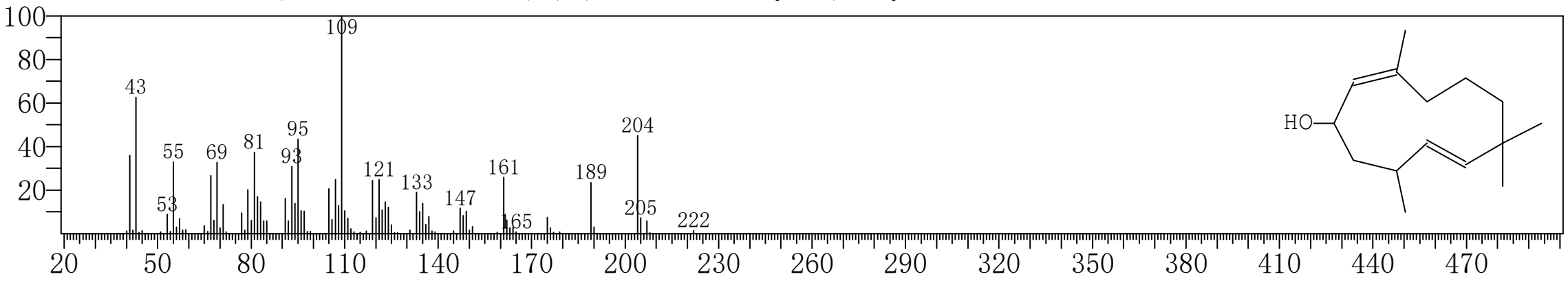

命中#:3　输入:68730　谱库:NIST17-1.lib
SI:79　分子式:C15H26O　CAS:19078-37-6　摩尔质量:222　保留指数:1685
组分名称:1-Naphthalenemethanol, 1,4,4a,5,6,7,8,8a-octahydro-2,5,5,8a-tetramethyl- $$ Drimenol $$ (2,5,5,8a-

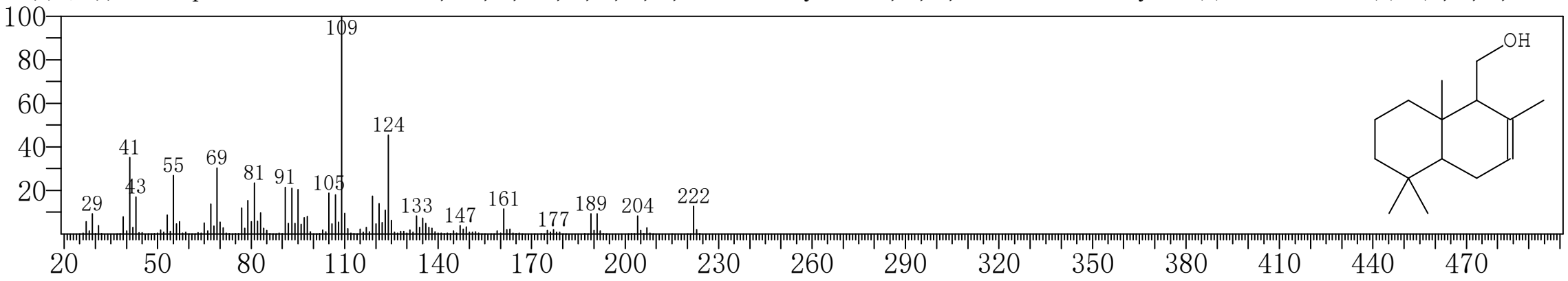

命中#:4　输入:68729　谱库:NIST17-1.lib
SI:79　分子式:C15H26O　CAS:21966-93-8　摩尔质量:222　保留指数:1534
组分名称:(1S,3aS,4S,5S,7aR,8R)-5-Isopropyl-1,7a-dimethyloctahydro-1H-1,4-methanoinden-8-ol $$ Copaborneol $

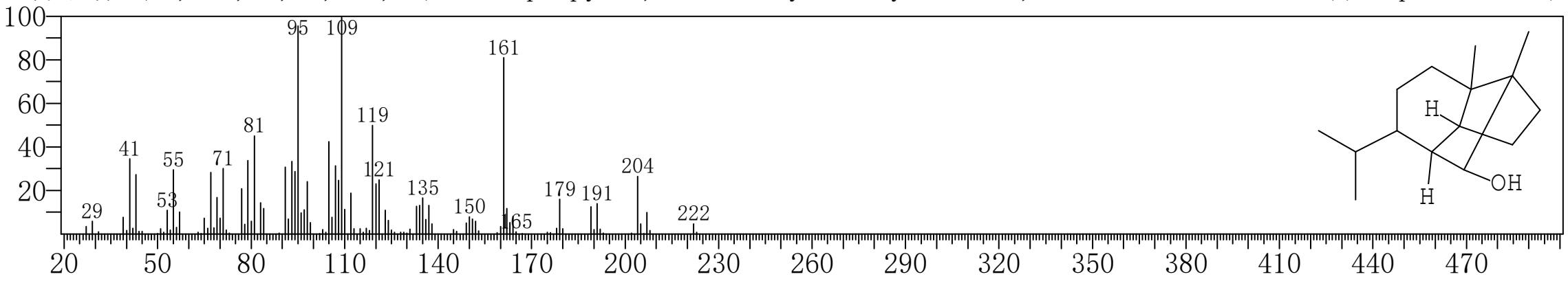

命中#:5　输入:68746　谱库:NIST17-1.lib
SI:77　分子式:C15H26O　CAS:19912-67-5　摩尔质量:222　保留指数:1580
组分名称:4a(2H)-Naphthalenol, 1,3,4,5,6,8a-hexahydro-4,7-dimethyl-1-(1-methylethyl)-, (1S,4R,4aS,8aR)- $$ E

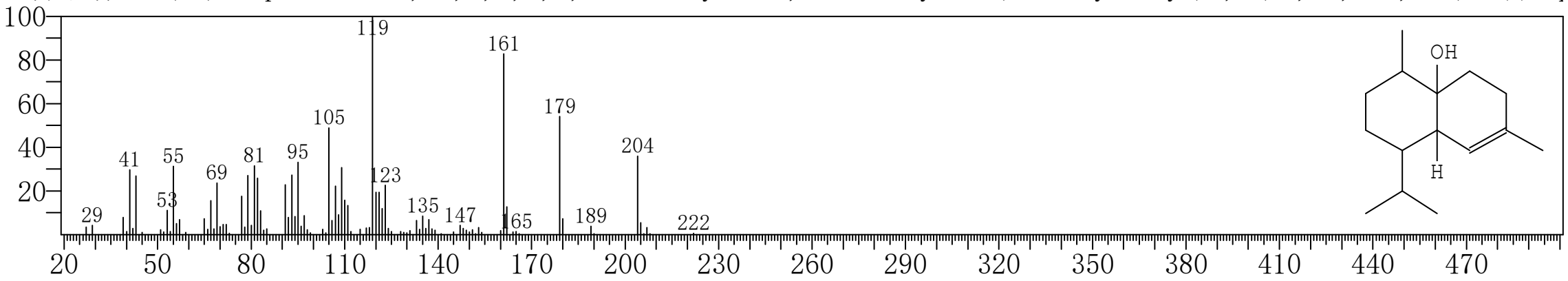

<< 目标组分 >>
行号#:56　保留时间:41.975(扫描数#:7396)　质量峰:295
原始模式:平均 41.970-41.980(7395-7397)　基峰:161.15(120471)
背景模式:峰校准　组 1 - 事件 1　Scan

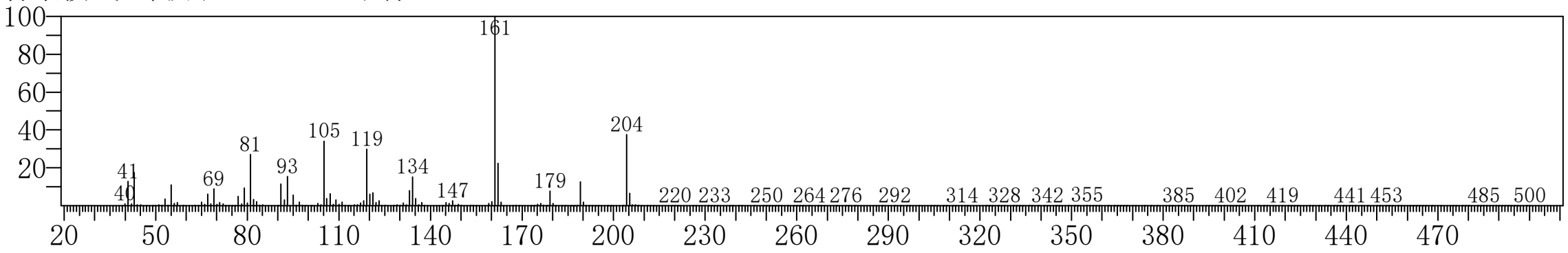

命中#:1　输入:25400　谱库:NIST17s.lib
SI:91　分子式:C15H26O　CAS:5937-11-1　摩尔质量:222　保留指数:1580
组分名称:.tau.-Cadinol $$ 4-Isopropyl-1,6-dimethyl-1,2,3,4,4a,7,8,8a-octahydro-1-naphthalenol-, (1S-(1alpha,

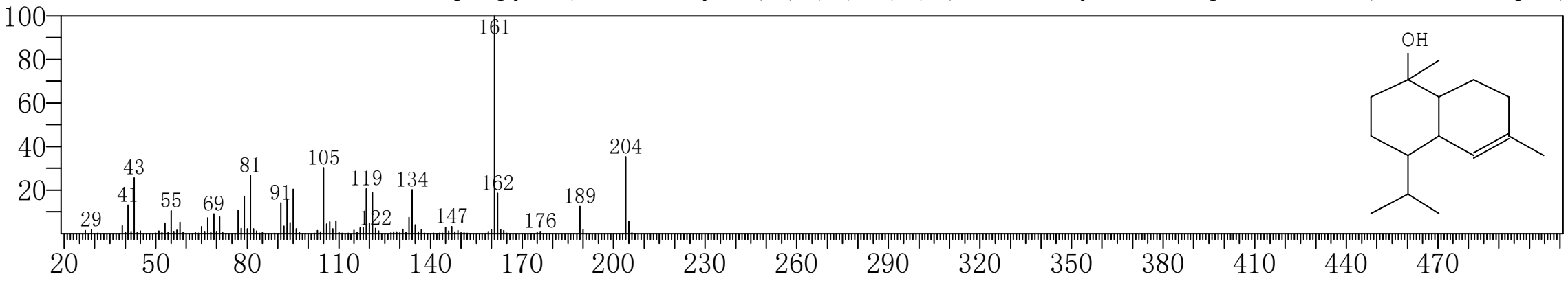

命中#:2　输入:52931　谱库:NIST17-1.lib
SI:88　分子式:C15H24　CAS:150320-52-8　摩尔质量:204　保留指数:1464
组分名称:Bicyclo[4.4.0]dec-1-ene, 2-isopropyl-5-methyl-9-methylene- $$ 8-Isopropyl-5-methyl-2-methylene-1,2,

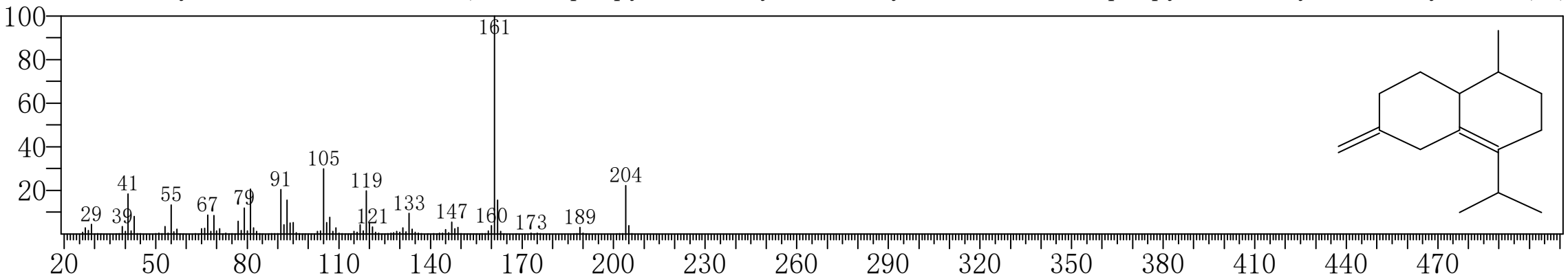

命中#:3　输入:107617　谱库:NIST17-1.lib
SI:87　分子式:C17H28O2　CAS:134-28-1　摩尔质量:264　保留指数:1804
组分名称:5-Azulenemethanol, 1,2,3,4,5,6,7,8-octahydro-.alpha.,.alpha.,3,8-tetramethyl-, acetate, [3S-(3.alpl

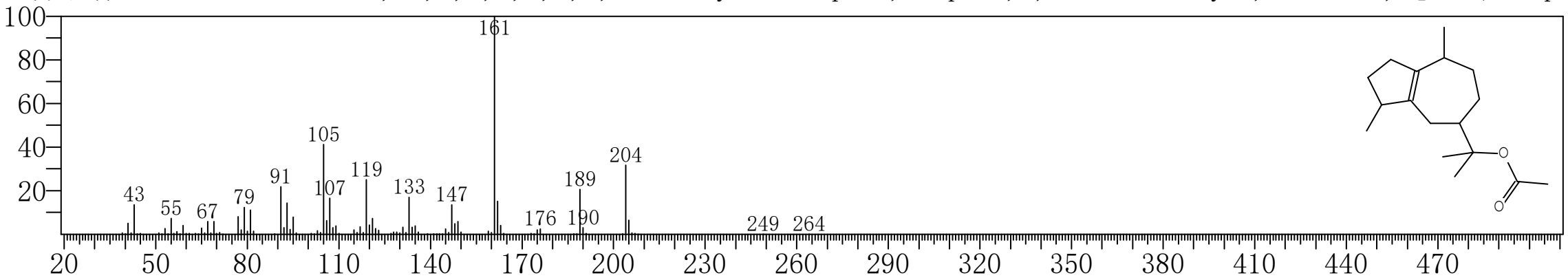

命中#:4　输入:1077　谱库:FFNSC1.3.lib
SI:87　分子式:C15 H24　CAS:16729-00-3　摩尔质量:204　保留指数:1472
组分名称:Cadina-1(6),4-diene

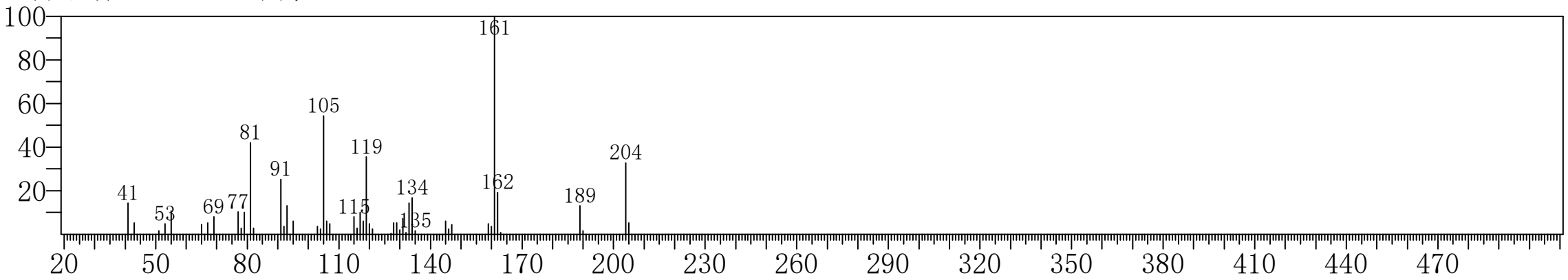

命中#:5　输入:52927　谱库:NIST17-1.lib
SI:86　分子式:C15H24　CAS:54274-73-6　摩尔质量:204　保留指数:1435
组分名称:(+)-epi-Bicyclosesquiphellandrene $$ 1-Isopropyl-4-methyl-7-methylene-1,2,3,4,4a,5,6,7-octahydronaj

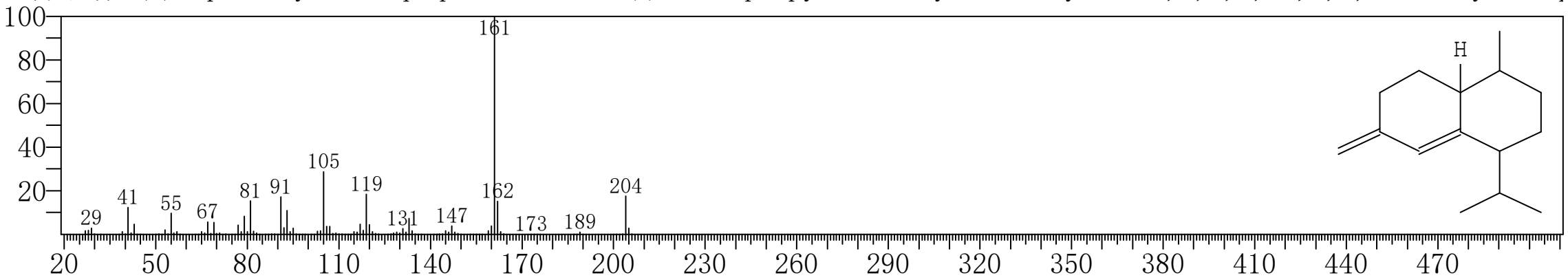

<< 目标组分 >>
行号#:57　保留时间:42.155(扫描数#:7432)　质量峰:314
原始模式:平均 42.150-42.160(7431-7433)　基峰:95.10(134945)
背景模式:峰校准　组 1 - 事件 1　Scan

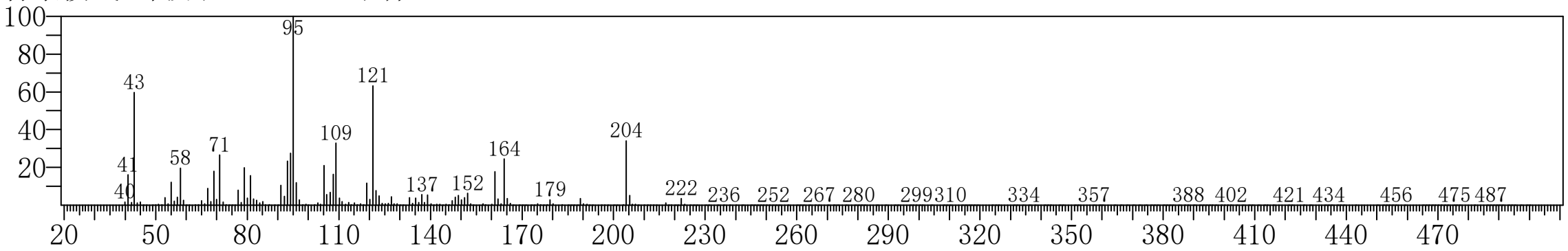

命中#:1　输入:68717　谱库:NIST17-1.lib
SI:89　分子式:C15H260　CAS:481-34-5　摩尔质量:222　保留指数:1580
组分名称:.alpha.-Cadinol $$ 4-Isopropyl-1,6-dimethyl-1,2,3,4,4a,7,8,8a-octahydro-1-naphthalenol # $$ (1R,4S,

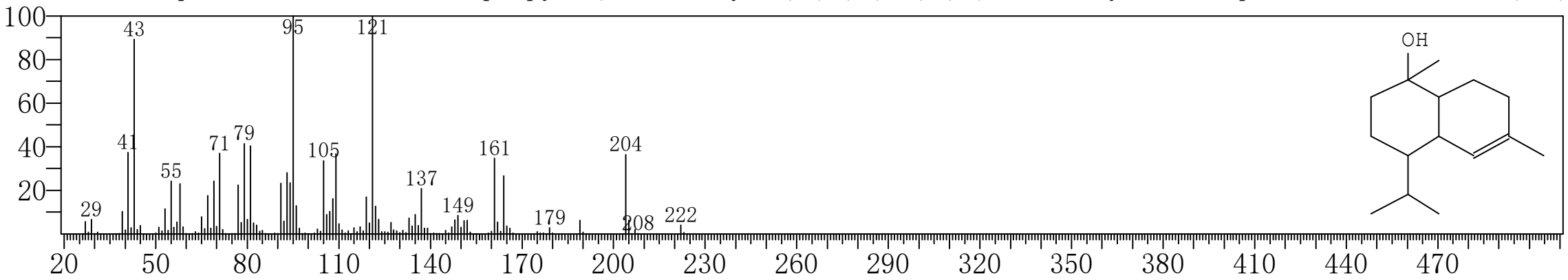

命中#:2　输入:1496　谱库:FFNSC1.3.lib
SI:89　分子式:C15 H26 0　CAS:481-34-5　摩尔质量:222　保留指数:1659
组分名称:Cadin-4-en-10-ol

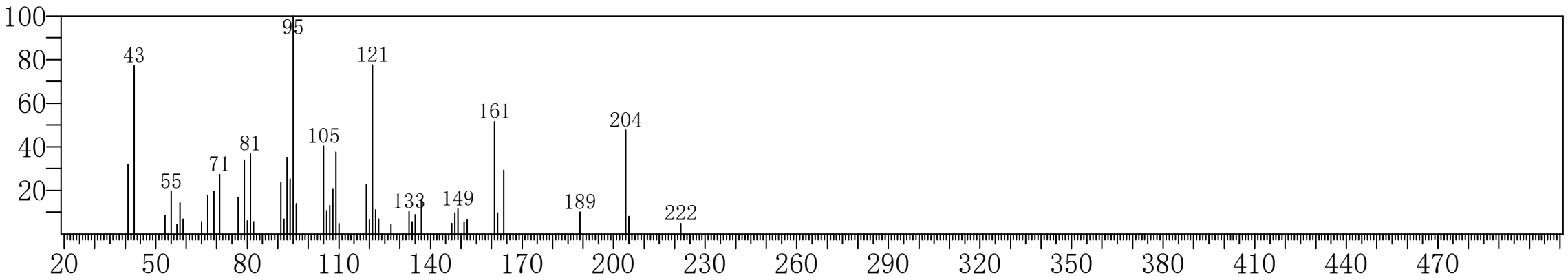

命中#:3　输入:25331　谱库:NIST17s.lib
SI:89　分子式:C15H260　CAS:481-34-5　摩尔质量:222　保留指数:1580
组分名称:.alpha.-Cadinol $$ 4-Isopropyl-1,6-dimethyl-1,2,3,4,4a,7,8,8a-octahydro-1-naphthalenol # $$ (1R,4S,

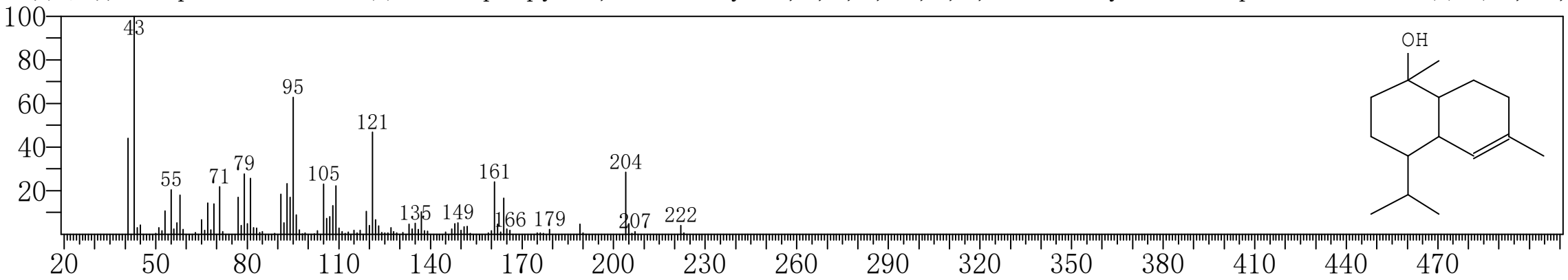

命中#:4　输入:68718　谱库:NIST17-1.lib
SI:85　分子式:C15H260　CAS:19912-62-0　摩尔质量:222　保留指数:1580
组分名称:.tau.-Muurolol $$ 4-Isopropyl-1,6-dimethyl-1,2,3,4,4a,7,8,8a-octahydro-1-naphthalenol-, [1S-(1.alpl

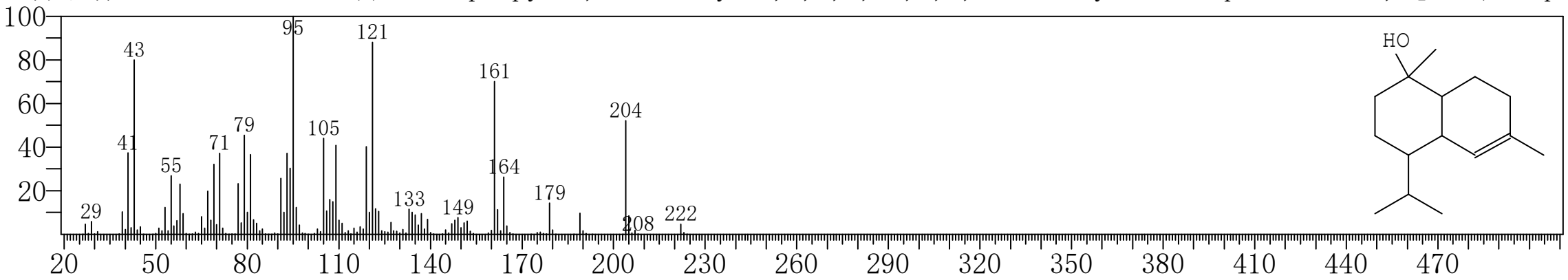

命中#:5　输入:25335　谱库:NIST17s.lib
SI:84　分子式:C15H260　CAS:19912-62-0　摩尔质量:222　保留指数:1580
组分名称:.tau.-Muurolol $$ 4-Isopropyl-1,6-dimethyl-1,2,3,4,4a,7,8,8a-octahydro-1-naphthalenol-, [1S-(1.alpl

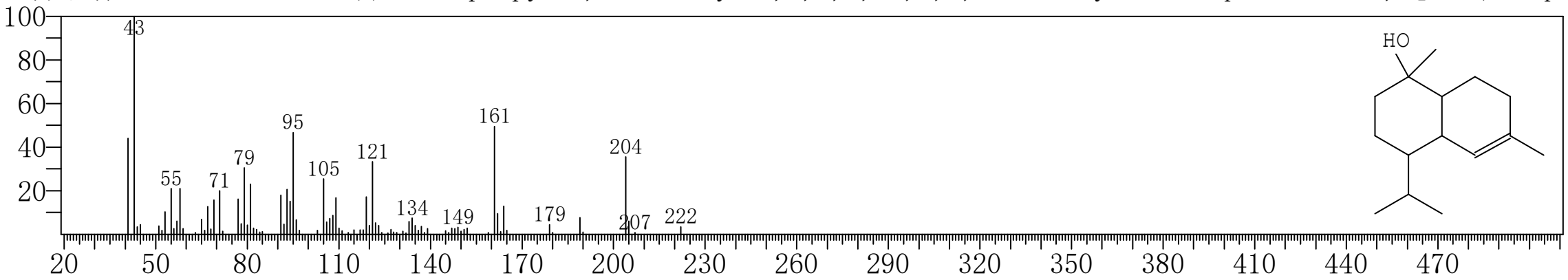

<< 目标组分 >>
行号#:58　保留时间:42.390(扫描数#:7479)　质量峰:259
原始模式:平均 42.385-42.395(7478-7480)　基峰:161.15(49967)
背景模式:峰校准　组 1 - 事件 1　Scan

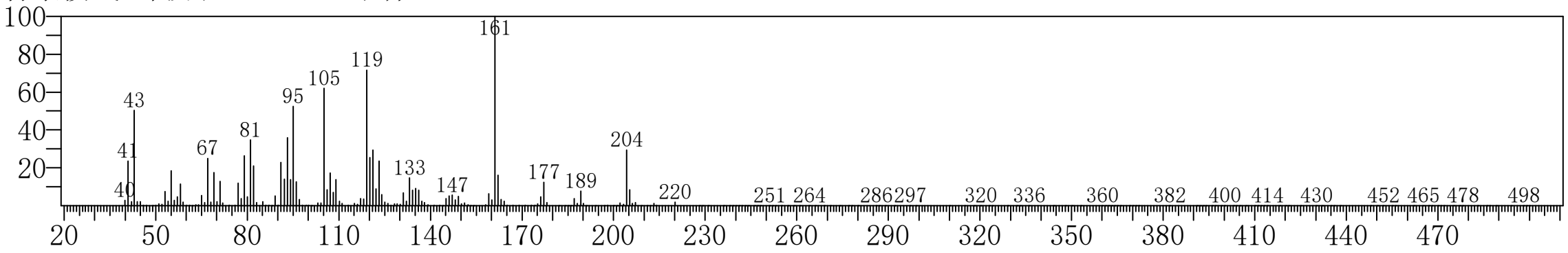

命中#:1　输入:25397　谱库:NIST17s.lib
SI:90　分子式:C15H26O　CAS:19435-97-3　摩尔质量:222　保留指数:1580
组分名称:1-Naphthalenol, 1,2,3,4,4a,7,8,8a-octahydro-1,6-dimethyl-4-(1-methylethyl)-, [1R-(1.alpha.,4.beta.,

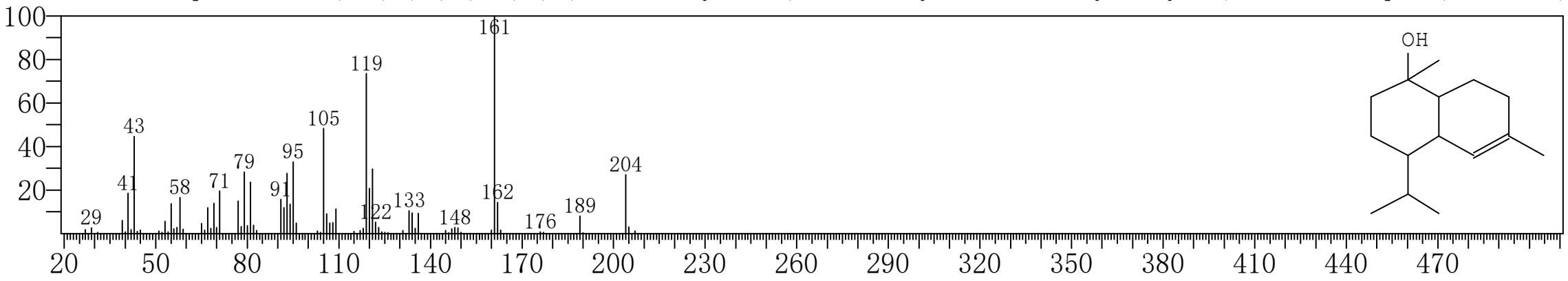

命中#:2　输入:68767　谱库:NIST17-1.lib
SI:89　分子式:C15H26O　CAS:19435-97-3　摩尔质量:222　保留指数:1580
组分名称:1-Naphthalenol, 1,2,3,4,4a,7,8,8a-octahydro-1,6-dimethyl-4-(1-methylethyl)-, [1R-(1.alpha.,4.beta.,

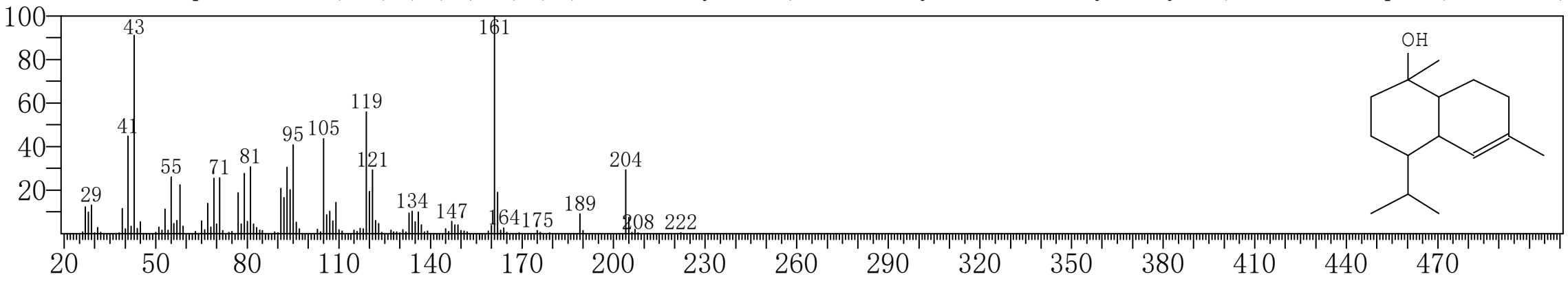

命中#:3　输入:1187　谱库:FFNSC1.3.lib
SI:89　分子式:C15 H26 O　CAS:19435-97-3　摩尔质量:222　保留指数:1651
组分名称:Muurolol <alpha->

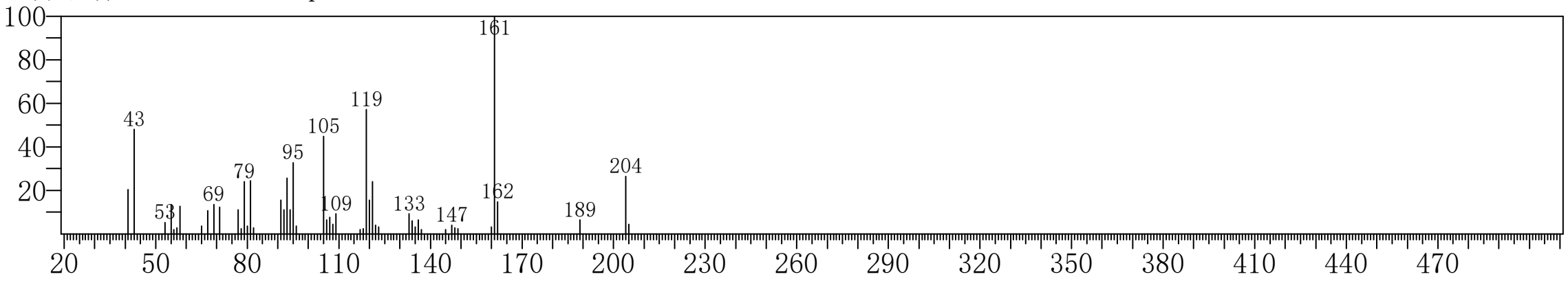

命中#:4　输入:25388　谱库:NIST17s.lib
SI:88　分子式:C15H26O　CAS:19435-97-3　摩尔质量:222　保留指数:1580
组分名称:1-Naphthalenol, 1,2,3,4,4a,7,8,8a-octahydro-1,6-dimethyl-4-(1-methylethyl)-, [1R-(1.alpha.,4.beta.,

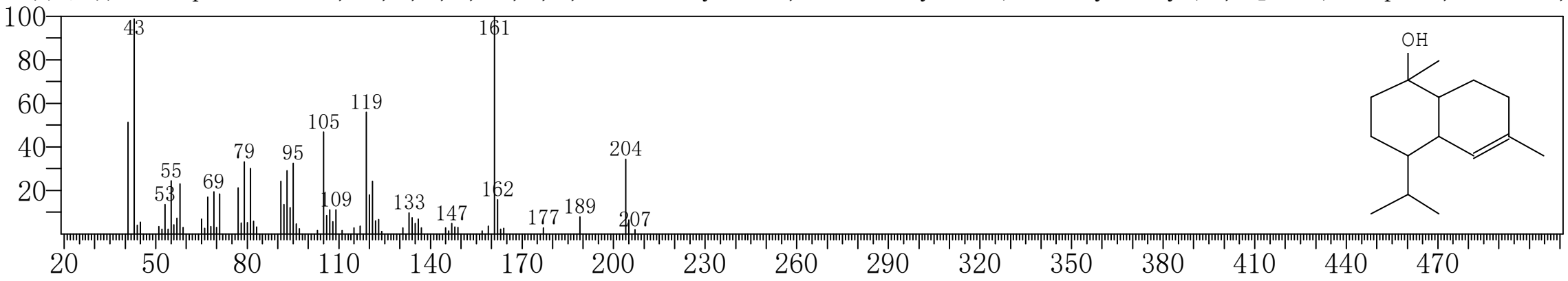

命中#:5　输入:25389　谱库:NIST17s.lib
SI:88　分子式:C15H26O　CAS:19435-97-3　摩尔质量:222　保留指数:1580
组分名称:1-Naphthalenol, 1,2,3,4,4a,7,8,8a-octahydro-1,6-dimethyl-4-(1-methylethyl)-, [1R-(1.alpha.,4.beta.,

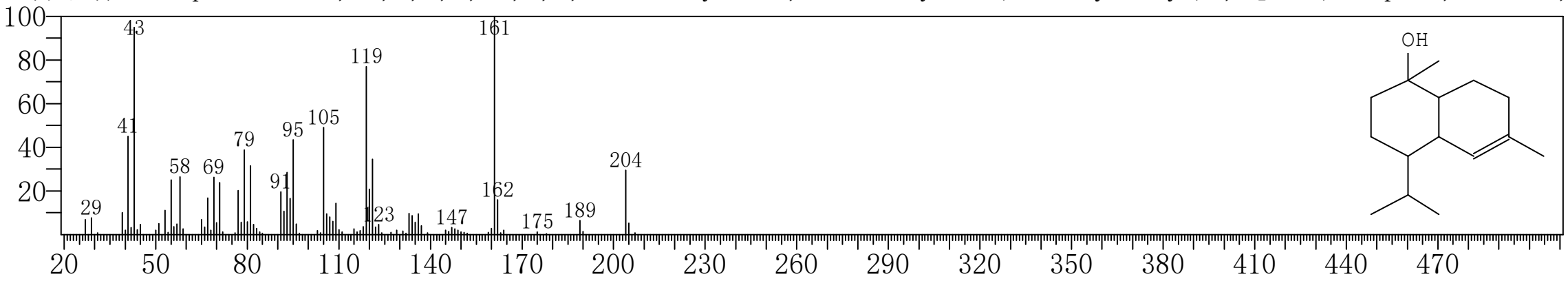

<< 目标组分 >>
行号#:59　保留时间:43.080(扫描数#:7617)　质量峰:287
原始模式:平均 43.075-43.085(7616-7618)　基峰:95.10(236675)
背景模式:峰校准　组 1 - 事件 1　Scan

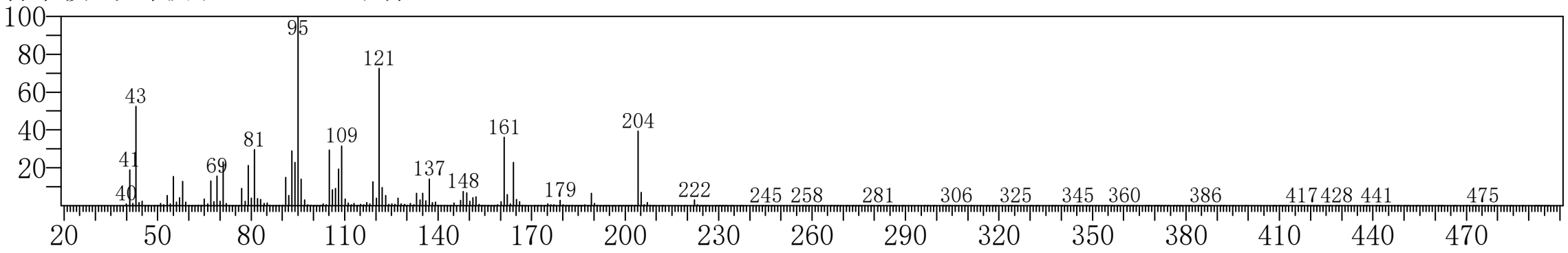

命中#:1　输入:1496　谱库:FFNSC1.3.lib
SI:93　分子式:C15 H26 O　CAS:481-34-5　摩尔质量:222　保留指数:1659
组分名称:Cadin-4-en-10-ol

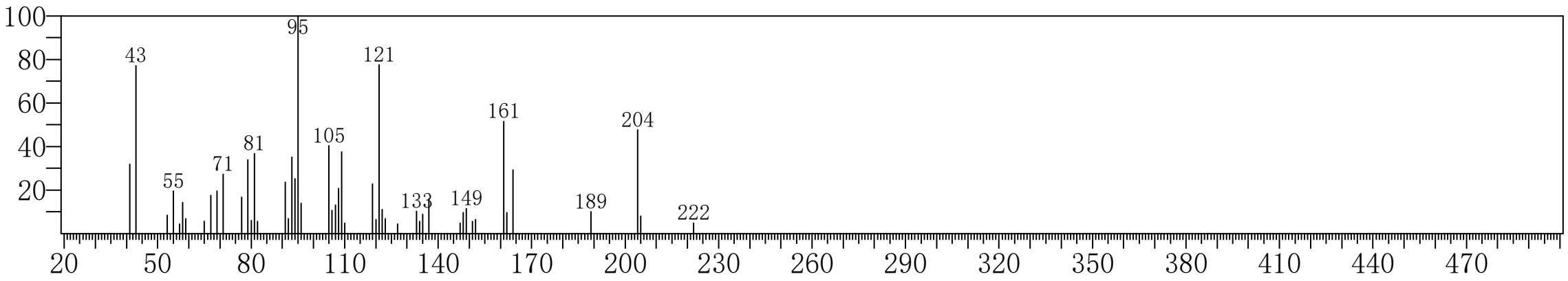

命中#:2　输入:68717　谱库:NIST17-1.lib
SI:92　分子式:C15H26O　CAS:481-34-5　摩尔质量:222　保留指数:1580
组分名称:.alpha.-Cadinol $$ 4-Isopropyl-1,6-dimethyl-1,2,3,4,4a,7,8,8a-octahydro-1-naphthalenol # $$ (1R,4S,

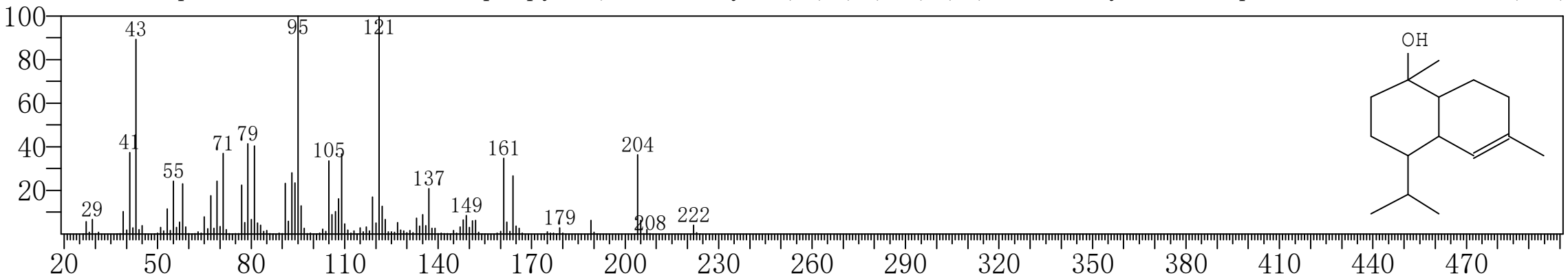

命中#:3　输入:25331　谱库:NIST17s.lib
SI:89　分子式:C15H26O　CAS:481-34-5　摩尔质量:222　保留指数:1580
组分名称:.alpha.-Cadinol $$ 4-Isopropyl-1,6-dimethyl-1,2,3,4,4a,7,8,8a-octahydro-1-naphthalenol # $$ (1R,4S,

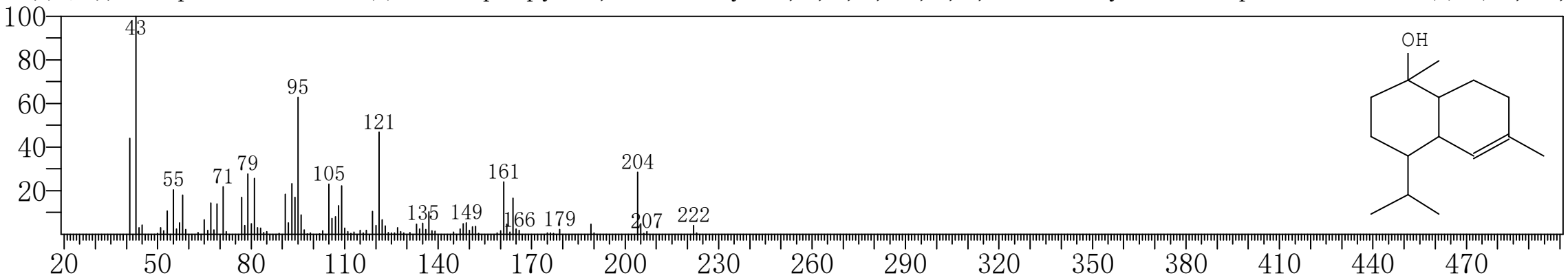

命中#:4　输入:68718　谱库:NIST17-1.lib
SI:87　分子式:C15H26O　CAS:19912-62-0　摩尔质量:222　保留指数:1580
组分名称:.tau.-Muurolol $$ 4-Isopropyl-1,6-dimethyl-1,2,3,4,4a,7,8,8a-octahydro-1-naphthalenol-, [1S-(1.alpl

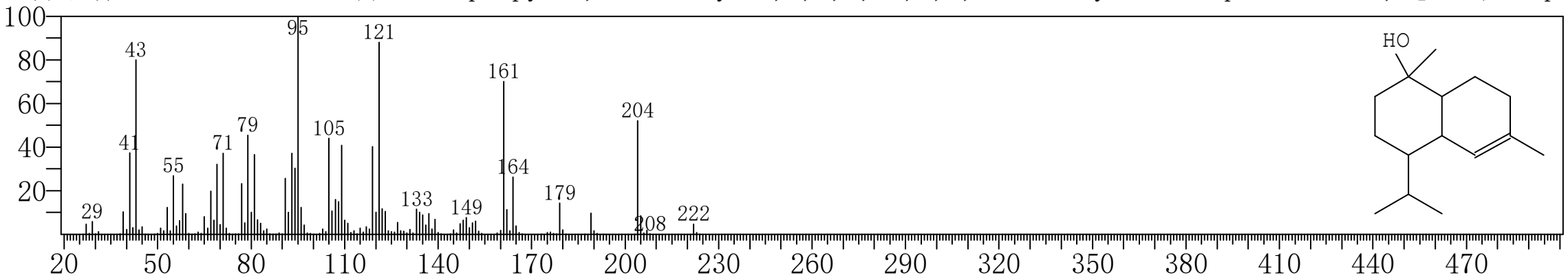

命中#:5　输入:25335　谱库:NIST17s.lib
SI:85　分子式:C15H26O　CAS:19912-62-0　摩尔质量:222　保留指数:1580
组分名称:.tau.-Muurolol $$ 4-Isopropyl-1,6-dimethyl-1,2,3,4,4a,7,8,8a-octahydro-1-naphthalenol-, [1S-(1.alpl

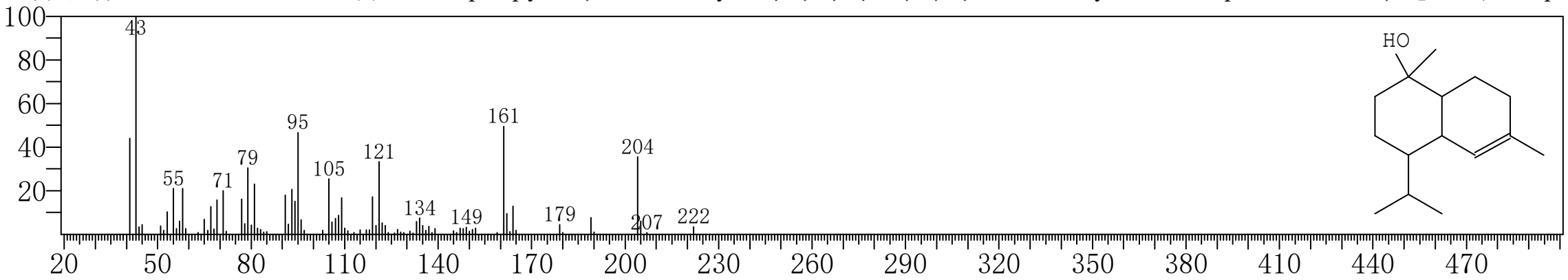

<< 目标组分 >>
行号#:60　保留时间:45.420(扫描数#:8085)　质量峰:300
原始模式:平均 45.415-45.425(8084-8086)　基峰:109.05(71362)
背景模式:峰校准　组 1 - 事件 1　Scan

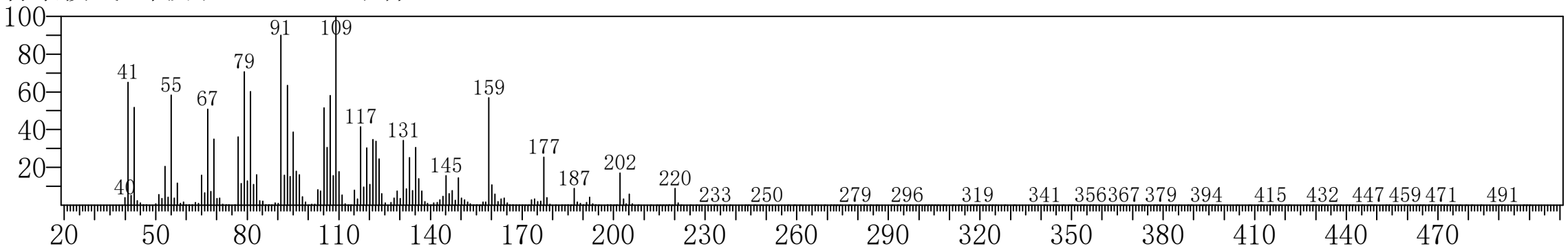

命中#:1　输入:66676　谱库:NIST17-1.lib
SI:95　分子式:C15H24O　CAS:81968-62-9　摩尔质量:220　保留指数:1699
组分名称:(1R,7S,E)-7-Isopropyl-4,10-dimethylenecyclodec-5-enol $$ 5-Cyclodecen-1-ol, 4,10-bis(methylene)-7-

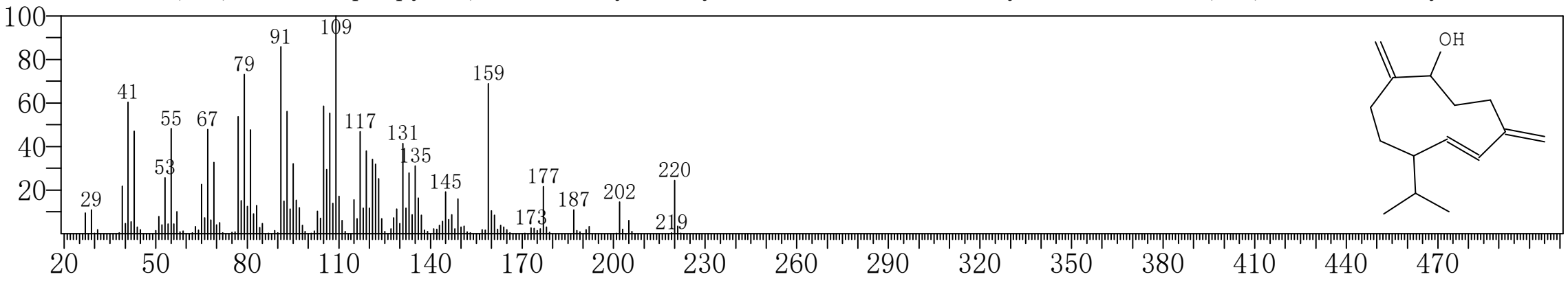

命中#:2　输入:66741　谱库:NIST17-1.lib
SI:87　分子式:C15H24O　CAS:119120-23-9　摩尔质量:220　保留指数:1636
组分名称:Eudesma-4(15),7-dien-1.beta. -ol $$ (1R,4aS,8aR)-6-Isopropyl-8a-methyl-4-methylene-1,2,3,4,4a,5,8,8

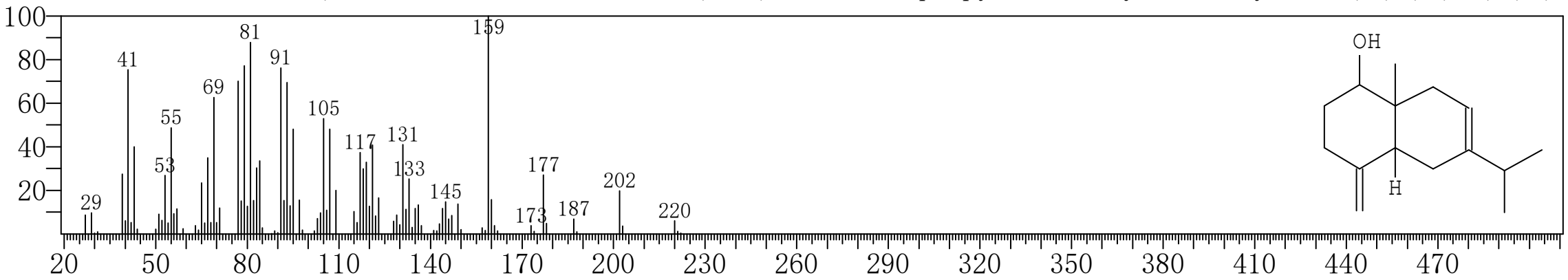

命中#:3　输入:66712　谱库:NIST17-1.lib
SI:83　分子式:C15H24O　CAS:115728-41-1　摩尔质量:220　保留指数:1464
组分名称:Tricyclo[4.4.0.0(2,7)]dec-3-ene-3-methanol, 1-methyl-8-(1-methylethyl)-

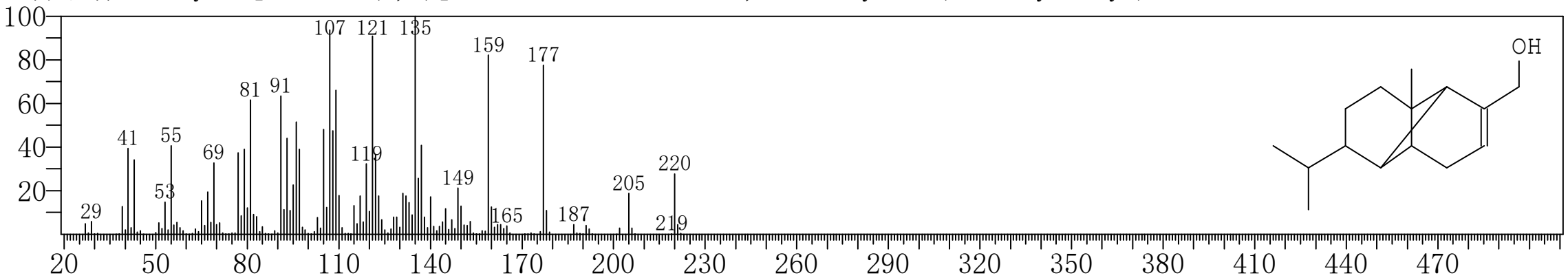

命中#:4　输入:192706　谱库:NIST17-1.lib
SI:82　分子式:C20H30O5　CAS:5508-58-7　摩尔质量:350　保留指数:2944
组分名称:Andrographolide $$ 2(3H)-Furanone, 3-[2-[decahydro-6-hydroxy-5-(hydroxymethyl)-5,8a-dimethyl-2-metl

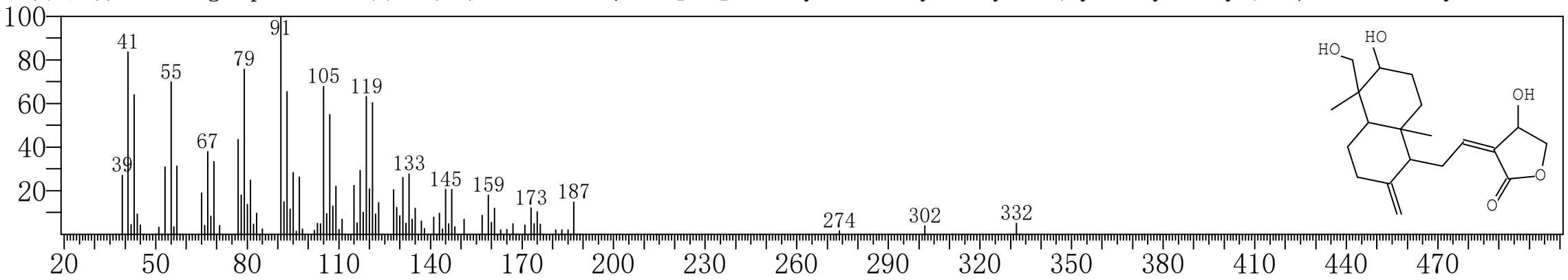

命中#:5　输入:903　谱库:FFNSC1.3.lib
SI:81　分子式:C15 H24 O　CAS:72203-24-8　摩尔质量:220　保留指数:1576
组分名称:Spathulenol

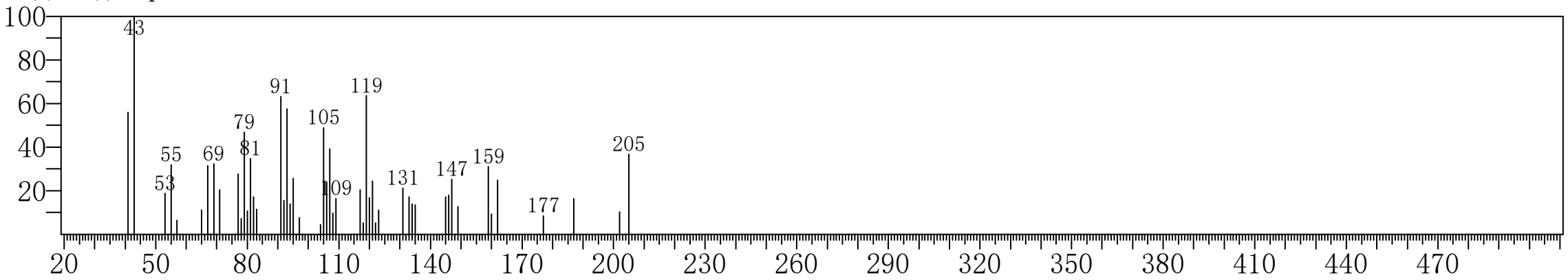

<< 目标组分 >>
行号#:61　保留时间:45.895(扫描数#:8180)　质量峰:286
原始模式:平均 45.890-45.900(8179-8181)　基峰:159.10(27808)
背景模式:峰校准　组 1 - 事件 1　Scan

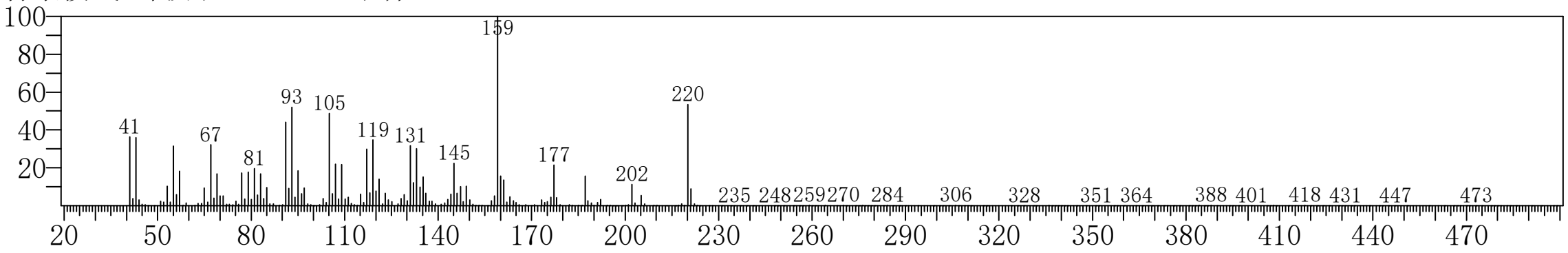

命中#:1　输入:66773　谱库:NIST17-1.lib
SI:86　分子式:C15H24O　CAS:161362-94-3　摩尔质量:220　保留指数:1754
组分名称:7R,8R-8-Hydroxy-4-isopropylidene-7-methylbicyclo[5.3.1]undec-1-ene $$ 7-Methyl-4-(1-methylethyliden

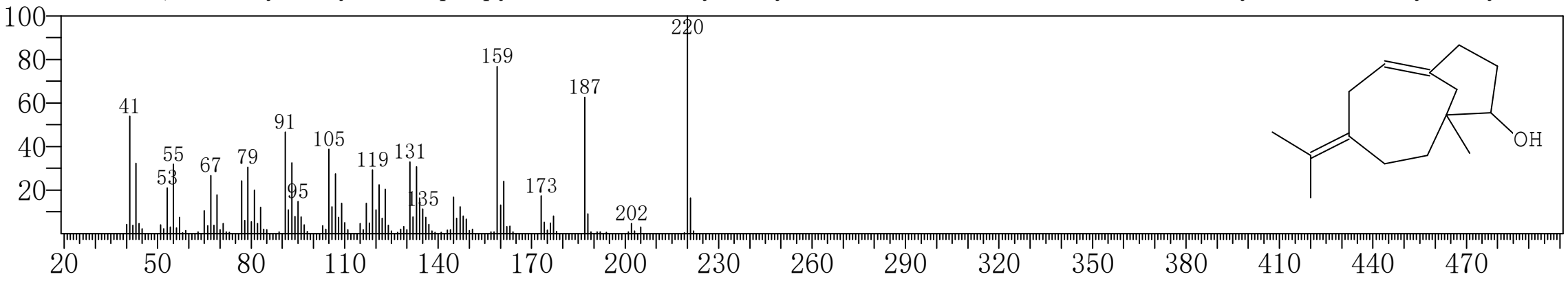

命中#:2　输入:66739　谱库:NIST17-1.lib
SI:85　分子式:C15H24O　CAS:0-00-0　摩尔质量:220　保留指数:1690
组分名称:6-Isopropenyl-4,8a-dimethyl-1,2,3,5,6,7,8,8a-octahydro-naphthalen-2-ol $$ 6-Isopropenyl-4,8a-dimetl

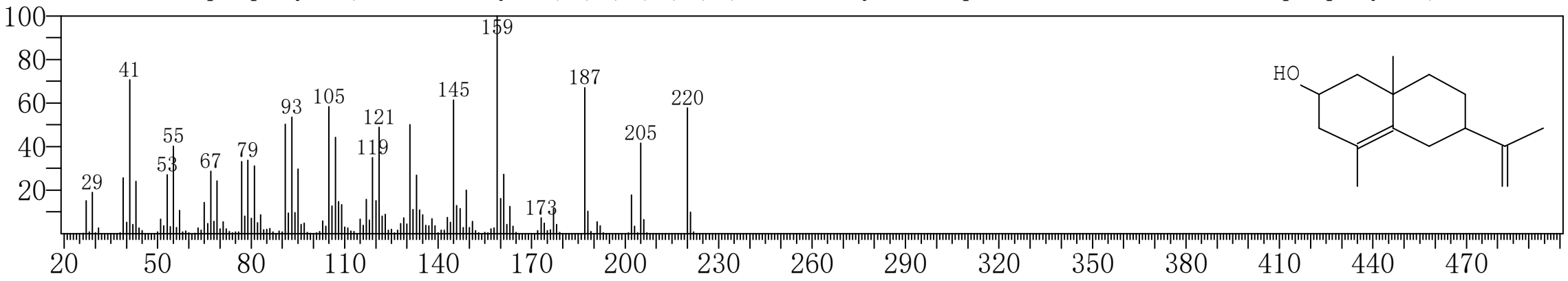

命中#:3　输入:66743　谱库:NIST17-1.lib
SI:81　分子式:C15H24O　CAS:135118-51-3　摩尔质量:220　保留指数:1683
组分名称:((4aS,8S,8aR)-8-Isopropyl-5-methyl-3,4,4a,7,8,8a-hexahydronaphthalen-2-yl)methanol $$ 2-Naphthalen

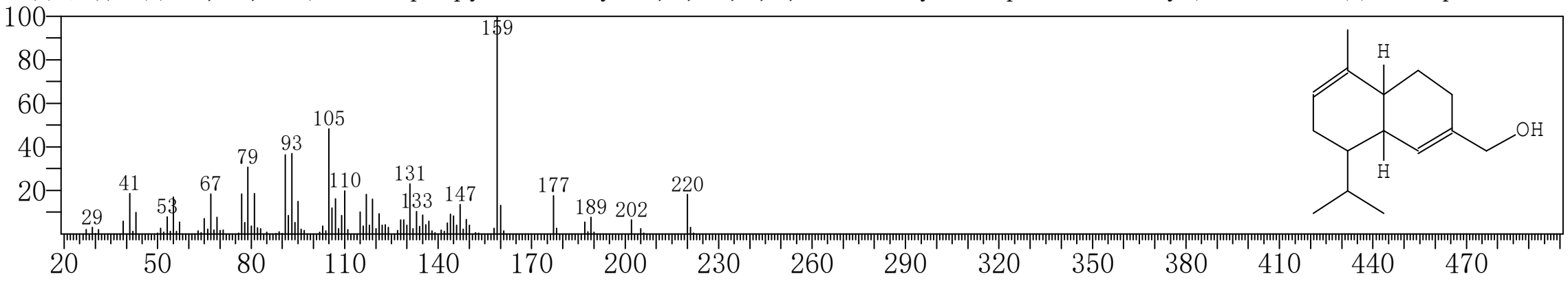

命中#:4　输入:66744　谱库:NIST17-1.lib
SI:81　分子式:C15H24O　CAS:124753-76-0　摩尔质量:220　保留指数:1405
组分名称:(1R,2R,4S,6S,7S,8S)-8-Isopropyl-1-methyl-3-methylenetricyclo[4.4.0.02,7]decan-4-ol $$ Tricyclo[4.4.

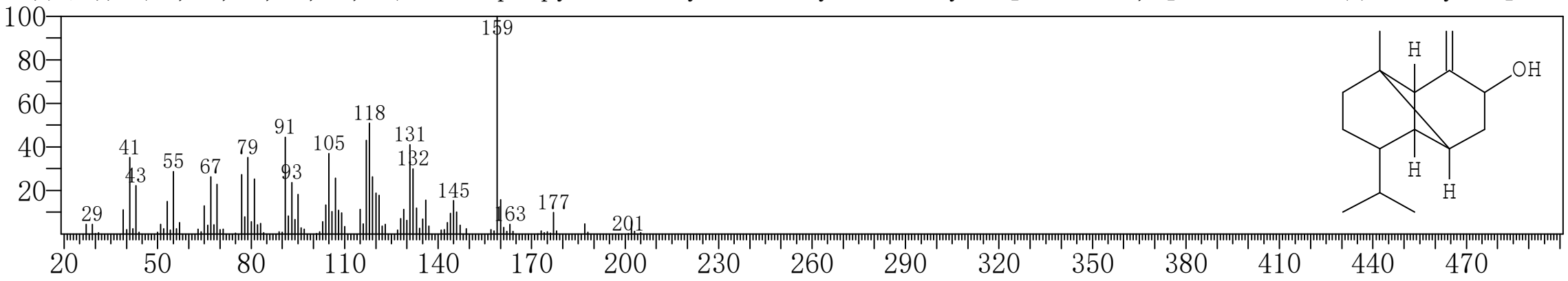

命中#:5　输入:66676　谱库:NIST17-1.lib
SI:80　分子式:C15H24O　CAS:81968-62-9　摩尔质量:220　保留指数:1699
组分名称:(1R,7S,E)-7-Isopropyl-4,10-dimethylenecyclodec-5-enol $$ 5-Cyclodecen-1-ol, 4,10-bis(methylene)-7-

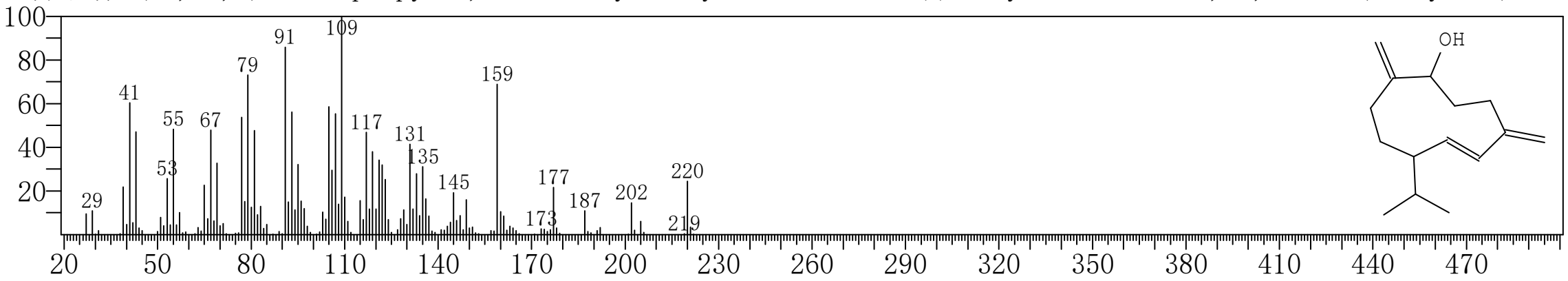

<< 目标组分 >>
行号#:62　 保留时间:49.020(扫描数#:8805)　质量峰:253
原始模式:平均 49.015-49.025(8804-8806)　 基峰:57.05(61792)
背景模式:峰校准　组 1 - 事件 1　Scan

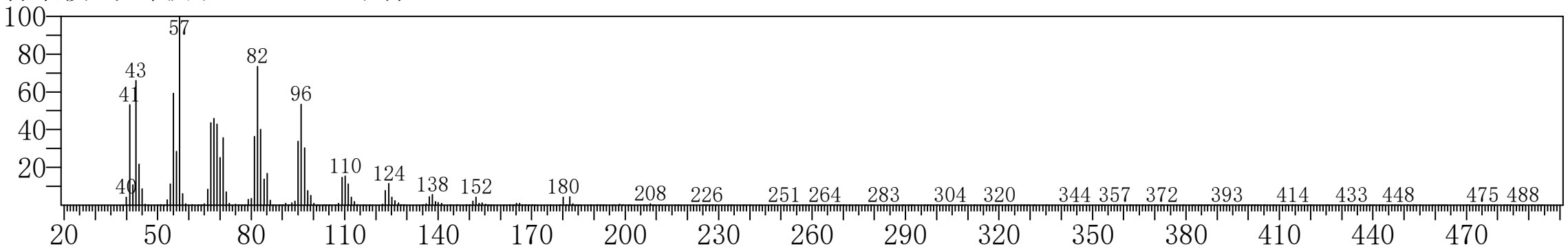

命中#:1　输入:25968　谱库:NIST17s.lib
SI:95　 分子式:C15H30O　CAS:2765-11-9　摩尔质量:226　保留指数:1701
组分名称:Pentadecanal- $$ 1-Pentadecanal $$ n-Pentadecanal $$

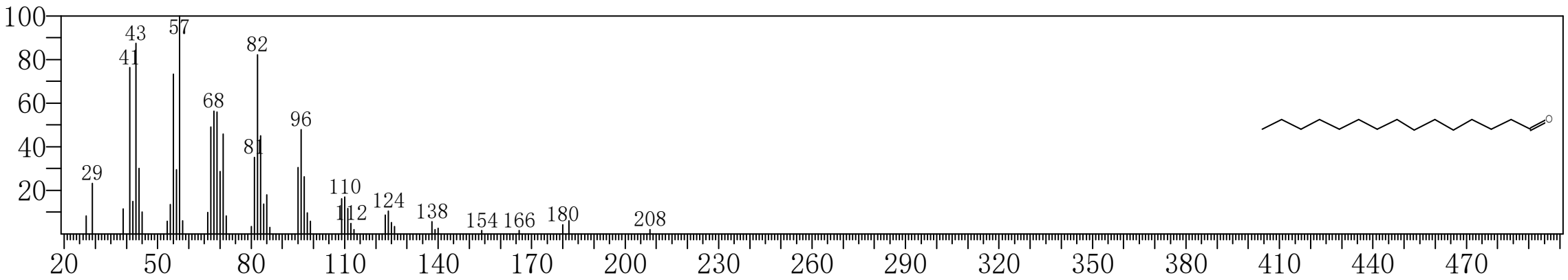

命中#:2　输入:98019　谱库:NIST17-1.lib
SI:95　 分子式:C17H34O　CAS:0-00-0　摩尔质量:254　保留指数:1899
组分名称:Heptadecanal

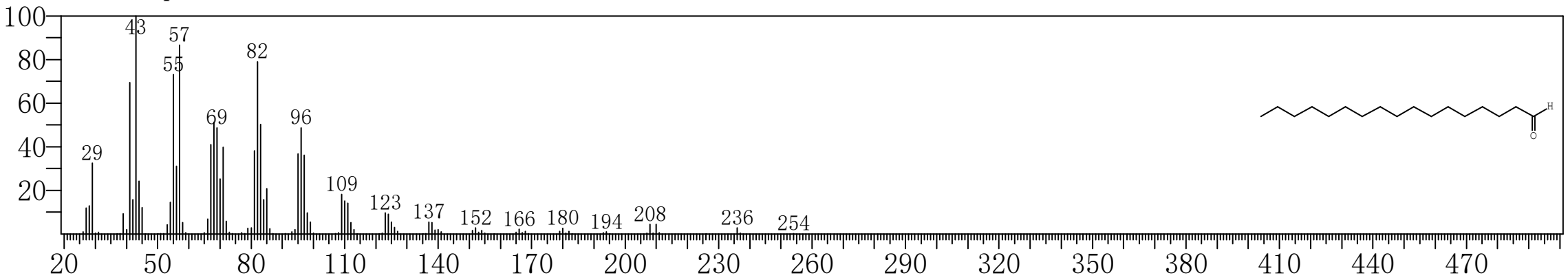

命中#:3　输入:23827　谱库:NIST17s.lib
SI:94　 分子式:C14H28O　CAS:124-25-4　摩尔质量:212　保留指数:1601
组分名称:Tetradecanal $$ Myristaldehyde $$ Myristylaldehyde $$ Tetradecylaldehyde $$ n-Tetradecanal $$ Aldel

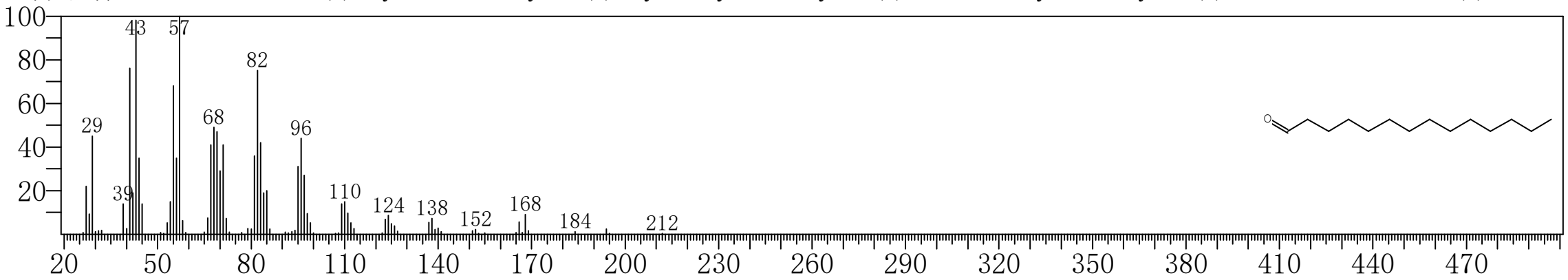

命中#:4　输入:21286　谱库:NIST17s.lib
SI:94　 分子式:C13H26O　CAS:10486-19-8　摩尔质量:198　保留指数:1502
组分名称:Tridecanal $$ n-Tridecylaldehyde $$ Tridecanaldehyde $$ Tridecyl aldehyde $$ 1-Tridecanal $$ Tridec

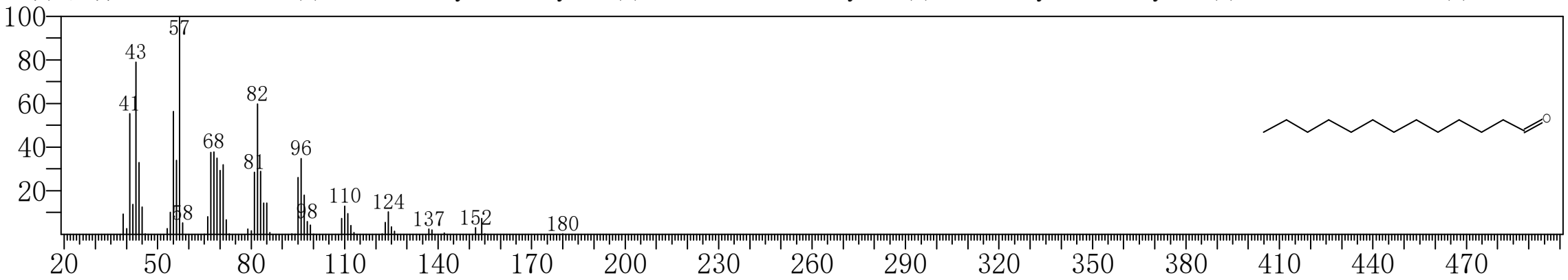

命中#:5　输入:25974　谱库:NIST17s.lib
SI:94　 分子式:C15H30O　CAS:2765-11-9　摩尔质量:226　保留指数:1701
组分名称:Pentadecanal- $$ 1-Pentadecanal $$ n-Pentadecanal $$

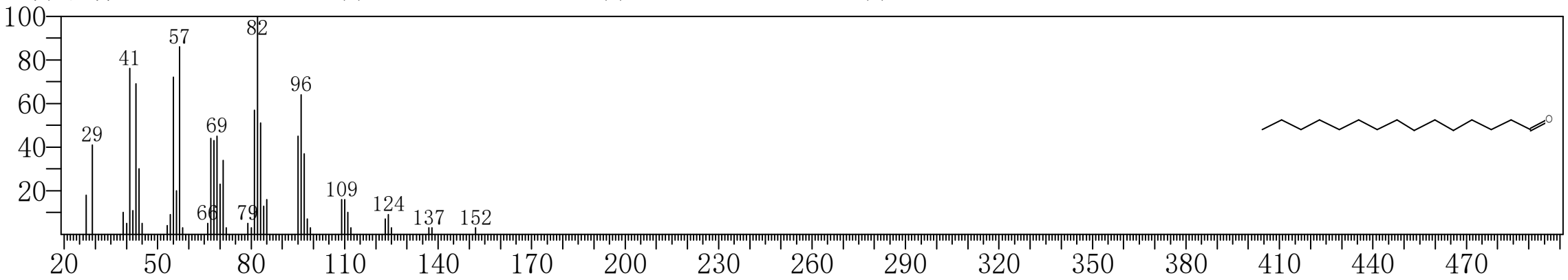

<< 目标组分 >>
行号#:63　保留时间:88.330(扫描数#:16667)　质量峰:327
原始模式:平均 88.325-88.335(16666-16668)　基峰:81.05(33739)
背景模式:峰校准　组 1 - 事件 1　Scan

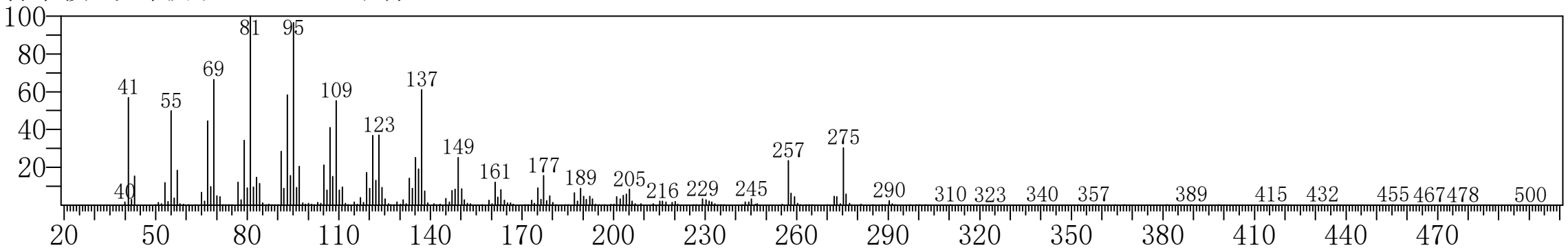

命中#:1　输入:133781　谱库:NIST17-1.lib
SI:91　分子式:C20H34O　CAS:21738-29-4　摩尔质量:290　保留指数:2162
组分名称:(E)-3-Methyl-5-((1R,4aR,8aR)-5,5,8a-trimethyl-2-methylenedecahydronaphthalen-1-yl)pent-2-en-1-ol $$

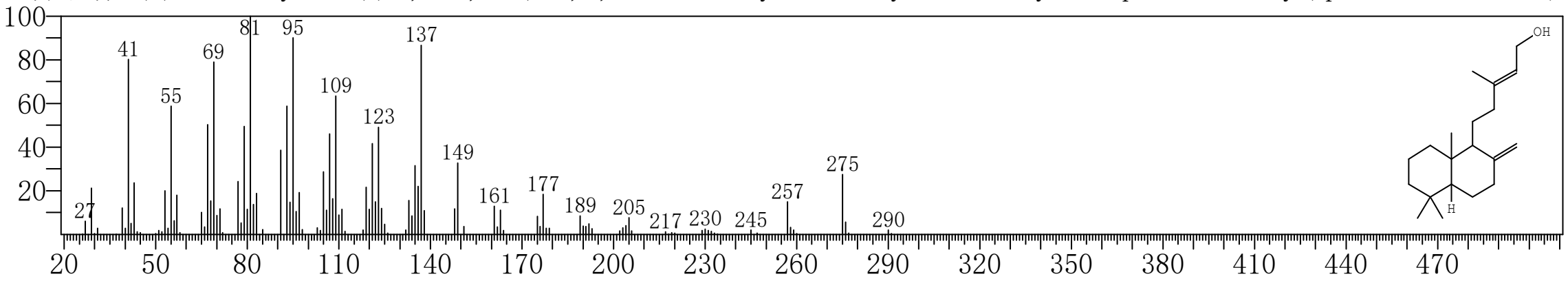

命中#:2　输入:949　谱库:FFNSC1.3.lib
SI:89　分子式:C20 H34 O　CAS:106631-38-3　摩尔质量:290　保留指数:2062
组分名称:Manool

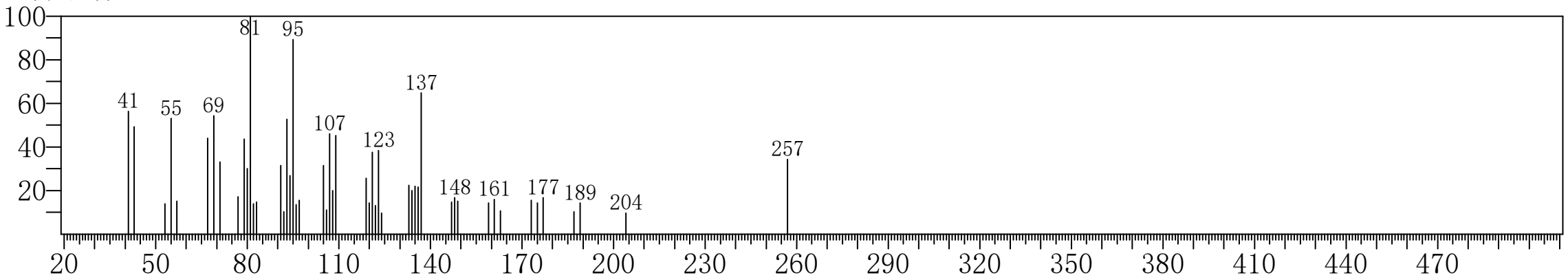

命中#:3　输入:32335　谱库:NIST17s.lib
SI:85　分子式:C20H34O　CAS:1438-62-6　摩尔质量:290　保留指数:2016
组分名称:1-Naphthalenepropanol, .alpha.-ethenyldecahydro-.alpha.,5,5,8a-tetramethyl-2-methylene-, [1S-[1.al

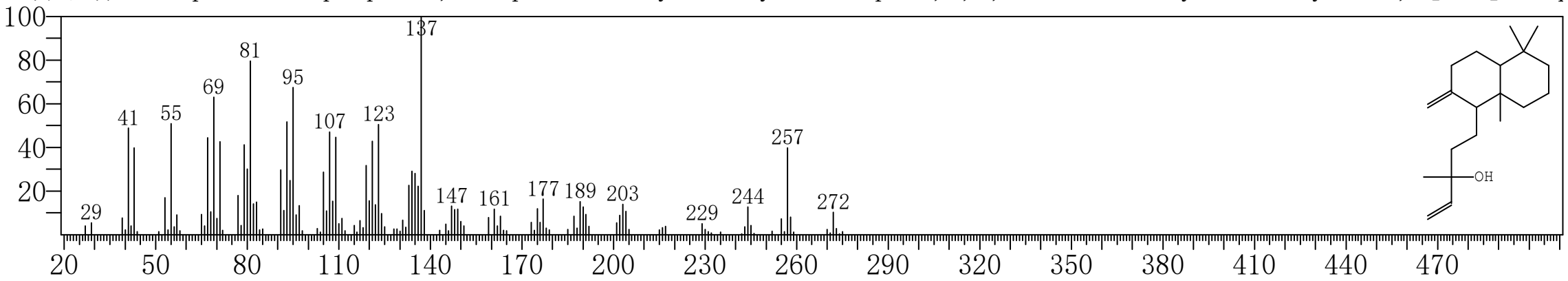

命中#:4　输入:148058　谱库:NIST17-1.lib
SI:85　分子式:C20H32O2　CAS:20257-75-4　摩尔质量:304　保留指数:2276
组分名称:(E)-3-Methyl-5-((1R,4aR,8aR)-5,5,8a-trimethyl-2-methylenedecahydronaphthalen-1-yl)pent-2-enoic acid

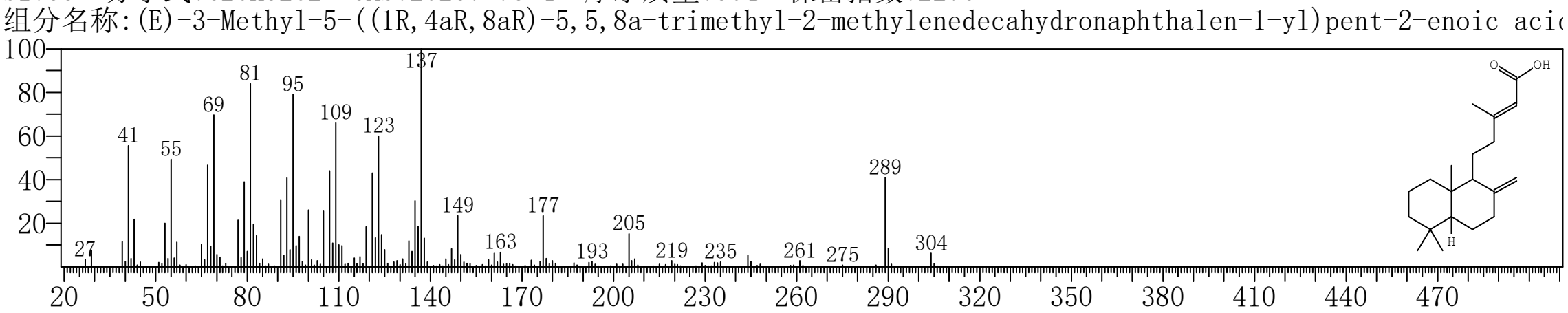

命中#:5　输入:247717　谱库:NIST17-1.lib
SI:83　分子式:C33H56　CAS:0-00-0　摩尔质量:452　保留指数:2885
组分名称:1,1,6-trimethyl-3-methylene-2-(3,6,9,13-tetramethyl-6-ethenye-10,14-dimethylene-pentadec-4-enyl)cyc

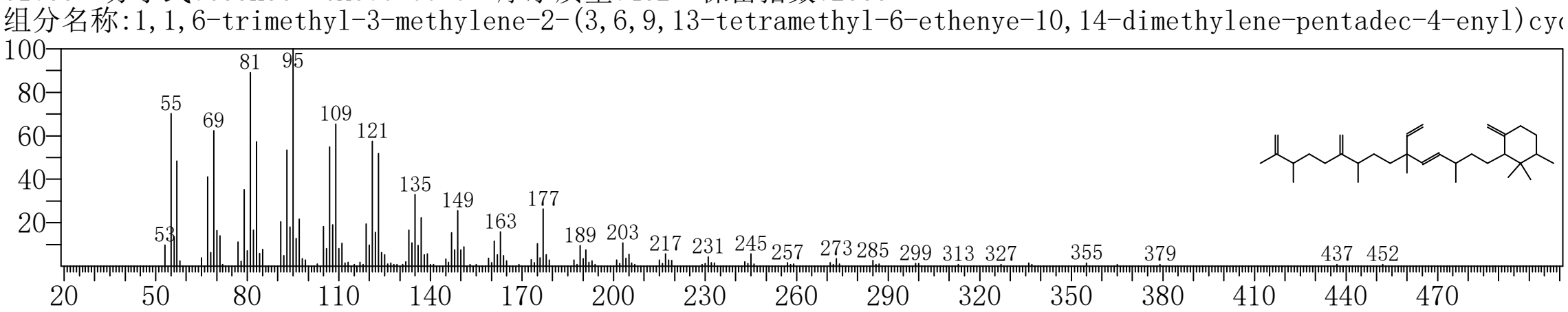

Supplement: Supplementary file 5 [file DataSheet5.pdf]
